# Emergence of antimicrobial resistance in New Caledonia: 20-year trends from laboratory-based surveillance (2005–2024)

**DOI:** 10.1016/j.lanwpc.2026.101913

**Published:** 2026-07-03

**Authors:** Andrea Antoniolli, Tiffany Ruge, Antoine Biron, Praveen Rahi, Claude Flamand, Alexandre Bourles, Cyrille Goarant, Julien Colot

**Affiliations:** a Medical and Environmental Bacteriology Group, Institut Pasteur de Nouvelle-Calédonie, Nouméa, New Caledonia; b Epidemiology and Public Health Unit, Institut Pasteur du Cambodge, Phnom Penh, Cambodia; c Université Paris-Cité, Paris, France; d Medical Biology Laboratory, Centre Hospitalier Territorial Gaston-Bourret, Nouméa, New Caledonia; e Bacteriology and Antibiotic Resistance, Medical Biology Laboratory, Institut Pasteur du Cambodge, Phnom Penh, Cambodia; f Mathematical Modelling of Infectious Diseases Unit, Institut Pasteur, Université Paris-Cité, U1332 INSERM, UMR2000 CNRS, Paris, France; g Public Health Division, The Pacific Community, Nouméa, New Caledonia

**Keywords:** Antimicrobial resistance, Surveillance, Time trends, New Caledonia

## Abstract

**Background:**

Antimicrobial resistance (AMR) issues have emerged in New Caledonia, particularly involving methicillin-resistant *Staphylococcus aureus* (MRSA), and carbapenem-resistant *Acinetobacter baumannii* (CR-AB). However, a comprehensive overall picture is still required to guide an antimicrobial stewardship.

**Methods:**

A prospective collection of antimicrobial susceptibility testing results was performed over a 20-year period (2005–2024) in the Territorial Hospital Center, the main hospital in New Caledonia. This surveillance encompassed 22 different pathogens and 28 antimicrobial agents, covering 157 pathogen-drug combinations. Resistance rates over time were analyzed using logistic models. A focus was placed on high-priority pathogens, including MRSA, CR-AB, vancomycin-resistant *Enterococcus faecium*, ceftazidime-resistant *Pseudomonas aeruginosa*, extended-spectrum β-lactamase Enterobacterales (ESBL-E), and carbapenemase-producing Enterobacterales (CPE). Multidrug-resistant (MDR) and possible extensively drug-resistant (pXDR) isolates were also identified and characterized.

**Findings:**

Across 111,022 included isolates, emergences of resistance were observed, notably to penicillins, third-generation cephalosporins, tetracycline, and fusidic acid. Temporal resistance trends were described, along with outbreaks of MRSA and CR-AB, and the emergence of ESBL-E and CPE, representing 3.7% [95% CI: 3.5–3.8] (N = 2208), and 0.1% [95% CI: 0.1–0.2] (N = 85) of the 59,954 Enterobacterales isolates, respectively. Overall, 11% [95% CI: 10.8–11.1] were MDR and 0.6% [95% CI: 0.5–0.6] were pXDR.

**Interpretation:**

Through trend analyses, this surveillance highlighted both the emergence and decline of AMR across diverse bacterial pathogens, helping inform which antibiotics may remain appropriate as first-line options and addressing the lack of data from Pacific Island settings.

**Funding:**

This work received financial support from the Pierre Ledoux Jeunesse Internationale scholarship (Fondation de France) and the Institut Pasteur, Paris.

**Research in context**

**Evidence before this study**

Antimicrobial resistance (AMR) is a major and growing global public health threat. In several regions, the World Health Organization (WHO) has highlighted gaps in surveillance and data availability. Oceania, and more specifically Pacific Island Countries and Territories (PICTs), remain settings where AMR evidence is limited. Search terms were used combining two domains, in PubMed up to January 2026: (1) AMR outcomes (eg, “antimicrobial resistance”, “drug resistance”, “antimicrobial susceptibility testing”, “antibiogram”), and (2) Western Pacific/Pacific Islands country and territory identifiers. Surveillance studies in PICTs were typically short in duration (generally up to 5 years) and covered a limited number of bacterial species and antimicrobials.

Across the PICTs, AMR resistance trends were described in 75 articles. Papua New Guinea was the most frequently investigated setting (34 articles), but all of these reports focused on a single pathogen. Fiji accounted for 14 studies, including four multi-pathogen reports: one covering 15 pathogens over four years (2019–2022), two focusing on Enterobacterales (2 and 3 years), and one investigating Enterobacterales, *Acinetobacter baumannii*, and *Pseudomonas aeruginosa* (2 years). In New Caledonia, eight studies reported AMR rate, each limited to a single bacterial species. A five-year surveillance report from the Cook Islands, Kiribati, Samoa, and Tonga investigated four bacterial species (*Staphylococcus aureus*, *Escherichia coli*, *Klebsiella pneumoniae*, and *P*. *aeruginosa*). Other territories contributed only one or two studies, generally describing resistance trends for a single species. In addition, the World Health Organization published four multi-country surveillance reports on *Neisseria gonorrhoeae*, each spanning one to two years. Overall, the lack of AMR data in insular Oceania persists. No long-term surveillance covering both a broad range of bacterial species and a broad range of antibiotics was identified in Pacific Island Countries and Territories (PICTs). Moreover, only two PICTs, Papua New Guinea and Fiji, reported AMR data to the WHO Global Antimicrobial Resistance and Use Surveillance System (GLASS) for 2023, and New Caledonia was not among them.

**Added value of this study**

This work analyses 157 pathogen–drug combinations across 22 bacterial species and 28 antimicrobials over 20 years (2005–2024). It is the first study in PICTs to combine such duration with such breadth of bacterial pathogens, providing a long-term, laboratory-based surveillance to identify key AMR threats. Antimicrobial susceptibility testing (AST), using European Committee on Antimicrobial Susceptibility Testing (EUCAST) guidelines, was performed at the Territorial Hospital Centre, the main hospital in New Caledonia. The surveillance included 111,022 clinical isolates. Resistance trends were described and temporal changes were analyzed using logistic models. Major regional threats and events were documented, including Methicillin Resistant *S*. *aureus* (MRSA) and carbapenem-resistant *A. baumannii* outbreaks caused by clonal spread in the Pacific region, and the emergence of extended-spectrum β-lactamase-producing Enterobacterales (ESBL-E) from 2007, and carbapenemase-producing Enterobacterales from 2013. Overall, multidrug resistance (MDR) accounted for 11% of isolates, and possible extensively drug-resistant phenotypes accounted for 0.6% among isolates that could be classified according to international definitions.

**Implications of all the available evidence**

Long-term, multi-pathogen surveillance provides actionable evidence to inform antimicrobial stewardship. It can capture both the emergence and decline of resistance, as well as clonal spread. Because Pacific territories are closely connected, resistant clones detected in New Caledonia also circulate in other settings and patient transfers with Australia can facilitate cross-border dissemination. Comprehensive local surveillance and the reporting of AMR trends across PICTs are therefore essential. Sustained, standardized surveillance across the region is needed to strengthen evidence, enable regional comparisons, and anticipate the introduction and spread of MDR pathogens, particularly in hospitals.

## Introduction

Antimicrobial resistance (AMR) is an increasingly pressing issue in the healthcare landscape. According to estimations, by 2050, 1.91 million [95% CI: 1.56–2.26] people could die worldwide directly due to AMR, within a total of 8.22 million [95% CI: 6.85–9.65] deaths that could be associated with AMR.^1^ In 2021, AMR was already associated with 4.71 million [95% CI: 4.23–5.19] deaths, including 1.14 million [95% CI: 1.00–1.28] directly attributable.^1^ Among the deadliest pathogen-drug combinations globally, Methicillin-resistant *S. aureus* (MRSA) and, carbapenem-resistant *A. baumannii* accounted for the highest number of deaths in 2021. These same combinations ranked among the top three causes of AMR-attributable deaths in Oceania with 12.4 [95% CI: 10.8–13.9] deaths per 100,000 people attributable to AMR in 2021, projected to rise to 20.3 [95% CI: 16.4–24.5] per 100,000 by 2050.^1^

These deaths result from the emergence of therapeutic dead ends that make some bacterial infections difficult, or even impossible, to treat. The World Health Organization (WHO) has identified several pathogen–drug resistance combinations, most involved in these therapeutic dead ends, that substantially increase the AMR burden and are therefore priorities for study within the strategy for AMR surveillance and control.^2^ In the critical priority group are carbapenem-resistant Enterobacterales, third-generation cephalosporin-resistant Enterobacterales, and carbapenem-resistant *A. baumannii*.^2^

The global AMR crisis is largely the consequence of decades of antibiotic use, misuse and overuse, leading to the strong selective pressure and spread of antibiotic-resistant bacterial pathogens, further aggravated by the transfer of resistance determinants between pathogens.^3^ Despite efforts such as raising awareness to promote more measured and responsible use of antibiotics in human and animal health or banning their use in Europe as growth promoters in livestock, resistant strains have nevertheless emerged and caused significant public health damage.^3^

Effective AMR surveillance requires the timely detection of emerging resistance and the assessment of its impact on antibiotic efficacy within specific geographic and epidemiological contexts. Microbiology laboratories and diagnostic centers can serve as sentinel sites for tracking the emergence, spread, and evolution of AMR, generating high-value, near–real-time data at the interface of pathogens, patients, and antimicrobial use. When compiled longitudinally, routine laboratory records enable robust detection of temporal trends and shifts in resistance patterns. However, the Western Pacific region, especially the Pacific Island Countries and Territories (PICTs), remains among the least monitored according to WHO Global Antimicrobial Resistance and Use Surveillance System (GLASS).^4^ New Caledonia does not currently participate in, or submit data to, the GLASS. However, data are reported to ConsoRes, a French national surveillance network in which the Territorial Hospital Center (CHT) has participated since 2017, allowing benchmarking of antimicrobial resistance and antibiotic consumption with other healthcare facilities in mainland France and the French overseas territories. In New Caledonia, published data in human health are fragmented and largely focused on specific pathogens, including *A. baumannii*,^5^ *S. aureus*,^6^^,^^7^ *N. gonorrhoeae*,^8^ and Enterobacterales.^9^, ^10^, ^11^ A comprehensive overview of antibiotic resistance and its time trend is still lacking.

This work aims to summarize the evolution of antibiotic resistance in New Caledonia from 2005 to 2024, to describe past and ongoing outbreaks of resistant bacteria, and to better prepare for future threats. Such an overview is essential to inform empirical treatment strategies, optimize antibiotic use, and support effective responses to bacterial infections within the medical community.

## Methods

### Study design and population

This long-term laboratory-based sentinel surveillance system is based on a prospective collection of antimicrobial susceptibility testing, performed according to European Committee on Antimicrobial Susceptibility Testing (EUCAST) standards,^12^ at the Territorial Hospital Center (CHT), the referral hospital in New Caledonia, a French territory within the Pacific region. Over a 20-year period, from January 1, 2005 to December 31, 2024, antimicrobial susceptibility was assessed from diagnostic clinical samples, collected from patients. A total of 22 bacterial pathogens, and 28 antimicrobial agents were included based on clinical relevance, resulting in 157 pathogen-drug combinations (Table S1). The surveillance design allowed repeated sampling over time, meaning that one patient could contribute multiple samples within the cohort, and each sample could contain multiple bacterial isolates.

### Data management

Data were extracted from the INLOG Laboserveur® laboratory information system and included nature and date of sampling, hospital admission date (for CHT inpatients), sex, age, AST details, and anonymized patient and sample IDs. Results of AST were reported as resistant, susceptible, high-dose susceptible, and intermediate.

Screening isolates collected for colonization surveillance were excluded, following international recommendations, to ensure conservative estimation and avoid overestimation of antimicrobial resistance rates.^4^^,^^13^ Specimens not subjected to antimicrobial susceptibility testing (AST), as well as duplicate isolates, were also excluded. Duplicates were defined as isolates from the same patient, of the same pathogen with no major differences in resistance profiles, collected within a maximum of 365 days. A major difference was defined as a change between resistant and susceptible results, while changes involving intermediate results or missing values were considered minor differences. In cases of duplicates, the most recent isolate was removed.^13^^,^^14^

The study included samples from all inpatient wards within CHT, grouped into six main departments: medicine, surgery, gynecology, intensive care, pediatrics, and geriatrics. A seventh category “Outpatients” grouped samples collected outside CHT or from patients who were not admitted. The samples were categorized into eleven sample types: blood culture, deep (abscess, surgical site), invasive (deep sterile site), skin, gynecological, ophthalmic, respiratory/ear-nose-throat, urinary, stool, neonatal fluid, and other.

The community- or hospital-acquired status was assigned to hospitalized patients based on the timing of sample collection relative to admission at the CHT. Isolates originating from samples collected more than two days after admission were classified as hospital-acquired. All other, including outpatients samples were considered community-acquired.^15^ Previous hospitalization in another facility was not accounted for. This operational definition was used consistently throughout the study to ensure comparability over time.

Susceptibility and non-susceptibility imputation followed EUCAST guidelines and are detailed in Table S2. Certain drugs were grouped together under a common reference if they were considered to be clinically equivalent.

High-priority pathogens (HPP) relevant to the epidemiological context of New Caledonia were characterized using specific resistance for some pathogen-drug combinations: Extended-spectrum β-lactamase Enterobacterales (ESBL-E), methicillin-resistant *S. aureus* (MRSA), carbapenem-resistant *A. baumannii* (CR-AB), ceftazidime-resistant *P. aeruginosa* (CAZR-PA), vancomycin-resistant *Enterococcus faecium* (VRE), and carbapenemase-producing Enterobacterales (CPE) (Table S3). Synergy tests were performed using amoxicillin/clavulanate and third- and fourth-generation cephalosporin discs to differentiate ESBLs and hyperproduced cephalosporinases. The presence of CPE was detected using ertapenem, temocillin and ceftolozane-tazobactam discs and confirmed using NG-CARBA-5® immunochromatographic tests (NG Biotech Laboratories, Guipry, France).

Isolates with resistance to at least one drug from each of three different antimicrobial classes, considering the 157 combinations (Table S4), were identified as multidrug resistant (MDR).^16^ Possible extensive drug-resistance (pXDR) was identified as susceptibility to a maximum of two antimicrobial classes, considering five, six or twelve classes relevant for each pathogen (Table S5).^16^ An alternative estimate of MDR and pXDR proportions was also performed using a non-susceptibility-based definition, in which intermediate isolates were grouped with resistant isolates, in accordance with the approach proposed by Magiorakos et al.^16^

### Calculation of antibiotic resistance percentage by pathogen

High-dose susceptible, and intermediate testing results were considered as susceptible,^17^ yielding a binary outcome (susceptible versus resistant). This approach follows EUCAST recommendations for surveillance analyses and ensures temporal consistency despite breakpoint updates over the study period. Antibiotic resistance rates were calculated as the proportion of resistant over susceptible plus resistant.

Intrinsic resistances were excluded from resistance percentage calculations, as well as for MDR and pXDR identification (Table S6).^18^^,^^19^

### Data analysis

Resistance trends were analyzed using two logistic regression approaches, both adjusted for age and sex. First, a logistic regression model with time as a continuous variable was used to estimate overall trends in antibiotic resistance over the 20-year period. Time was computed in years from the date of the first sample recorded for each pathogen providing an odds ratio (OR) representing the average yearly change in resistance over the study period. Second, a logistic regression model using sampling year as a categorical variable was applied to detect year-to-year variations in resistance. In this approach, each year was compared to the immediately preceding year using the function contrast (object, method = “consec”, adjust = “fdr”) from the *emmeans* package. False discovery rate (FDR) correction was systematically applied to account for multiple testing across numerous pathogen-drug combinations, yelding OR for annual changes in resistance. Contrasts that were uninterpretable due to small sample sizes were not considered.

Analyses combining all isolates, including those exploring MDR and pXDR, were performed using generalized linear mixed models (GLMMs), incorporating a random effect for bacterial pathogens to account for between-pathogen heterogeneity.

Additional stratified analyses focused on Enterobacterales (*E. coli, K. pneumoniae, Citrobacter freundii, Citrobacter koseri, Enterobacter cloacae complex, Proteus mirabilis, Morganella morgannii, Salmonella spp., Shigella spp., Serratia marcescens*). Analyses were also stratified by antibiotic class:

o Fluoroquinolones (ciprofloxacin, moxifloxacin, levofloxacin).
o Third-generation cephalosporins (3GCs) (cefotaxime, ceftazidime).

For class-level analyses, resistance was defined conservatively at the isolate level: an isolate was classified as resistant to an antibiotic class if at least one representative antibiotic within that class was reported as resistant.

Missing values were handled by complete-case analysis for each variable. Confidence intervals were calculated using the Wilson method. Differences were considered statistically significant at p < 0.05 (∗), p < 0.001 (∗∗) and p < 0.0001 (∗∗∗). All analyses were performed using R version 4.3.1.^20^

### Ethics approval

Clinical data were routinely collected and the analysis was conducted under the CNIL reference methodology MR-004 (Deliberation No. 2018-155, 3 May 2018); therefore, it did not require review by a French Research Ethics Committee (CPP). Patients are informed that data collected during routine care may be used for research, and they can withdraw at any time by exercising their right to object via the CHT data transparency portal: https://www.cht.nc/recherche-medicale/portail-transparence-donnees/. However, the analysis was approved by the Comité Consultatif d’Éthique de Nouvelle-Calédonie pour les Sciences de la Vie et de la Santé (New Caledonia Advisory Ethics Committee for Life and Health Sciences) on 24 November 2025.

### Role of the funding source

The funders had no role in the study or manuscript preparation.

## Results

### Study inclusion and dataset description

**Fig. 1:**
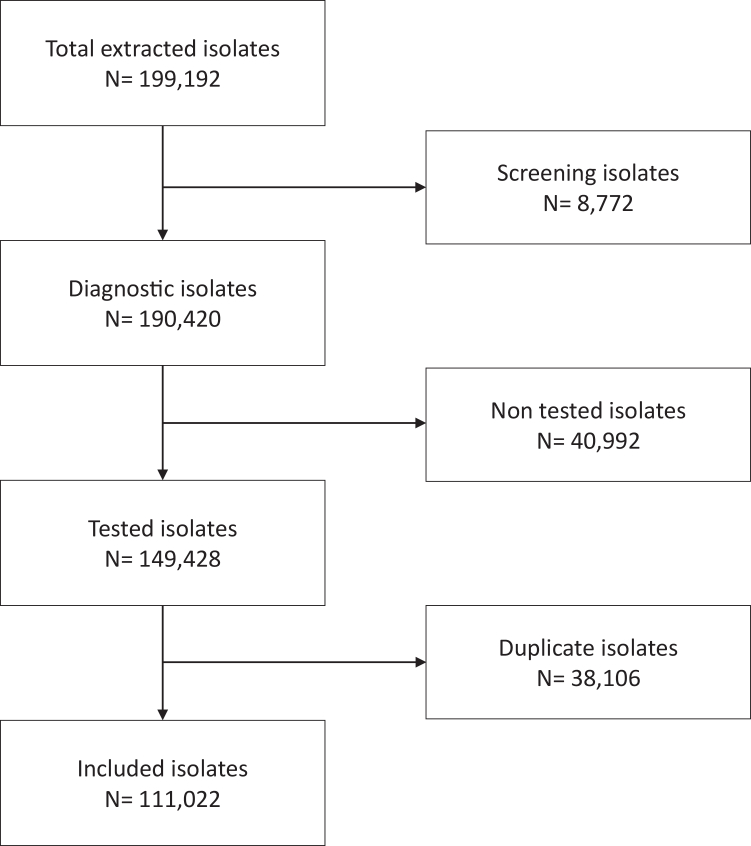
**Flowchart showing the inclusion of isolates**.

After excluding screening isolates, untested isolates, and duplicates, a total of 111,022 isolates were included in the analysis (Fig. 1, details by pathogen in Table S7), originating from 102,419 samples from 65,046 patients. The median patient age was 30 years [IQR: 15–57] with 54.6% [95% CI: 54.2–55.0] females and 45.4% [95% CI: 45.0–45.8] males. Over the 20-year period, 34.1% [95% CI: 33.7–34.4] of patients contributed more than one sample, reflecting repeated healthcare contacts or follow-up sampling. The CHT performs more than half of all microbiological antimicrobial susceptibility testing conducted in the territory, although no population-wide denominator is available.

### Included isolates

The annual number of included isolates ranged from 4814 in 2024 to 6219 in 2009 (Table S8). *E*. *coli* (N = 35,940; 32.4% [95% CI: 32.1–32.6]) and *S. aureus* (N = 24,406; 22% [95% CI: [21.7–22.2]) (Table S9) were the most frequently isolated pathogens. Overall, Enterobacterales accounted for more than half of all isolates (54% [95% CI: 53.7–54.3] while non-fermenters represented 7.3% [95% CI: 7.2–7.5] and streptococci 2.8% [95% CI: 2.7–2.9]. Most isolates were community-acquired (82.5% [95% CI: 82.3–82.8]) and urine samples were the most frequent specimen type (33.3% [95% CI: 33.1–33.6]) (Table 1). Three fifths of isolates (60.5% [95% CI: 60.2–60.8]) were from CHT patients. Approximately half of outpatients were managed through the CHT emergency department (N = 23,762).

**Table 1:** Characteristics of isolates (statistical unit).

	Median [IQR]	Frequency	Percentage [95% CI]
Age	39 [20–64]		
Sex			
Female		59,866	54.2% [53.9–54.5]
Male		50,608	45.8% [45.5–46.1]
NA		548	
Acquisition status			
Community-acquired		89,853	82.5% [82.3–82.8]
Hospital-acquired		19,010	17.5% [17.2–17.7]
NA		2159	
Sample type			
Urinary		36,714	33.3% [33.1–33.6]
Deep		19,547	17.7% [17.5–18.0]
Respiratory/ear-nose-throat		14,646	13.3% [13.1–13.5]
Gynecological		11,172	10.1% [10.0–10.3]
Skin		10,561	9.6% [9.4–9.8]
Hemoculture		10,503	9.5% [9.4–9.7]
Neonate fluid		3932	3.6% [3.5–3.7]
Stool		1211	1.1% [1.0–1.2]
Ophthalmic		1021	0.9% [0.9–1.0]
Invasive		768	0.7% [0.6–0.7]
Other		77	0.1% [0.1–0.1]
NA		870	
Department			
Outpatients		43,876	39.2% [39.2–39.8]
Medicine		24,332	21.9% [21.7–22.2]
Surgery		14,637	13.2% [13.0–13.4]
Intensive Care Unit		10,093	9.1% [8.9–9.3]
Pediatrics		9391	8.5% [8.3–8.6]
Gynecology		7263	6.5% [6.4–6.7]
Geriatrics		1430	1.3% [1.2–1.4]

### Enterobacterales and key antimicrobial agents: amikacin, fluoroquinolones, 3GCs, imipenem, piperacillin/tazobactam, and cotrimoxazole

**Fig. 2:**
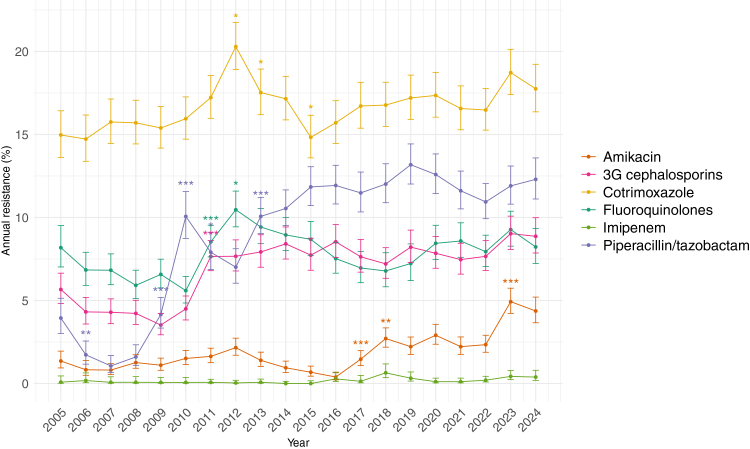
**Annual resistance percentages among the subset of 59,954 Enterobacterales isolates for key antimicrobial agents, from 2005 to 2024**. Year-to-year differences were assessed using contrasts from a logistic model: significance refers to comparisons with the immediately preceding year. Differences were considered statistically significant at p < 0.05 (∗), p < 0.001 (∗∗) and p < 0.0001 (∗∗∗).

Among Enterobacterales, resistance to amikacin and imipenem did not exceed 5% (Fig. 2) (Tables S10–S16). Resistance to 3 GC increased significantly in 2011 (OR = 1.7, p-value <0.001), reaching a in 2023 of 9% [95% CI: 8.1–10.1]. Resistance to cotrimoxazole peaked at 20.3% [95% CI: 18.9–21.8] in 2012 (OR = 1.23, p-value = 0.030). Fluoroquinolones resistance among Enterobacterales increased significantly in 2011 to 8.6% [95% CI: 7.7–9.6] (OR = 1.51, p-value = 0.001) and again in 2012 to 10.5% [95% CI: 9.4–11.6] (OR = 1.28, p-value = 0.044), before stabilized around 8% until 2024. Resistance to piperacillin/tazobactam showed three significant stepwise increases: in 2009 (OR = 2.7, p-value <0.001), 2010 (OR = 2.49, p-value <0.001), and in 2013 (OR = 1.5, p-value <0.001), before stabilizing at 12%.

### Pathogen-drug combinations

**Fig. 3:**
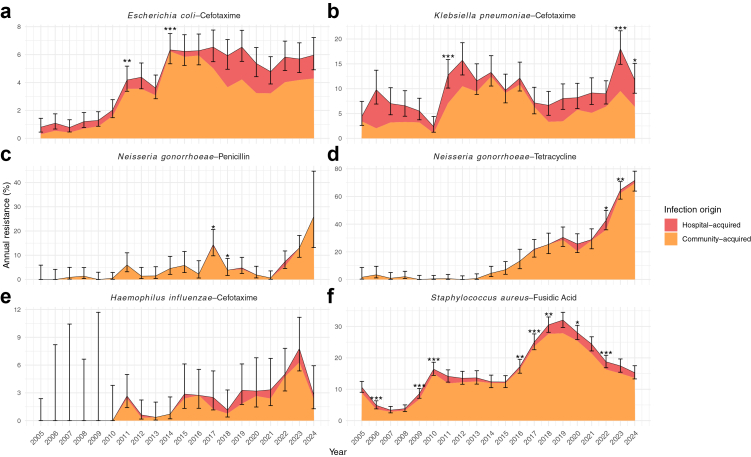
**Annual percentage of resistance for six pathogen-drug combinations: (a) *E. coli*-cefotaxime, (b) *K. pneumoniae*-cefotaxime, (c) *N. gonorrhoeae*-penicillin, (d) *N. gonorrhoeae*-tetracycline, (e) *H. influenzae*-cefotaxime, (f) *S. aureus*-fusidic acid, with annual segregations between hospital-acquired and community-acquired infections, from 2005 to 2024**. Year-to-year differences were assessed using contrasts from a logistic model: significance refers to comparisons with the immediately preceding year. Differences were considered statistically significant at p < 0.05 (∗), p < 0.001 (∗∗) and p < 0.0001 (∗∗∗).

All 157 pathogen-drug combinations are detailed in Tables S17–S195 and Figures S1–S22 with temporal effects modeled both continuously, and categorically using rolling years. Six pathogen-drug combinations suspected to be problematic in New Caledonia are highlighted in Fig. 3. A significant increase in *E*. *coli* resistance to cefotaxime was observed in 2011 versus 2010 (OR = 2.03, p = 0.003) and 2014 versus 2013 (OR = 1.86, p = 0.001). Over the 20-year study period, the continuous analysis revealed a significant overall upward trend in *N*. *gonorrhoeae* resistance to penicillin (OR = 1.14, p < 0.001) and tetracycline (OR = 1.42, p < 0.001), reaching 25.9% [95% CI: 13.2–44.7] and 71.6% [95% CI: 63.9–78.3], respectively at the end of the data collection in 2024. An increase was also observed in *Haemophilus influenzae* resistance to cefotaxime (OR = 1.17, p-value <0.001). Furthermore, *S. aureus* resistance to fusidic acid increased significantly in two phases, between 2009–2010 and 2016–2018, peaking at 32.1% [95% CI: 29.7–34.5] in 2019, and declined thereafter.

Throughout the study period, hospital-acquired *E. coli* and *K. pneumoniae* isolates showed higher rates of resistance to cefotaxime than community-acquired isolates (Figure S23). For *H. influenzae* resistance to cefotaxime and *S. aureus* resistance to fusidic acid, resistance rates were approximately similar between hospital- and community-acquired isolates.

### High priority pathogens in New Caledonia

Among all isolates, 8483 (7.6% [95% CI: 7.5–7.8]) were classified as HPP. Whereas 7.3% [95% CI: 7.1–7.5] of community-acquired infections were HPP, 9.3% [95% CI: 8.9–9.7] of hospital-acquired infections were HPP (χ^2^ test, p < 0.001).

**Fig. 4:**
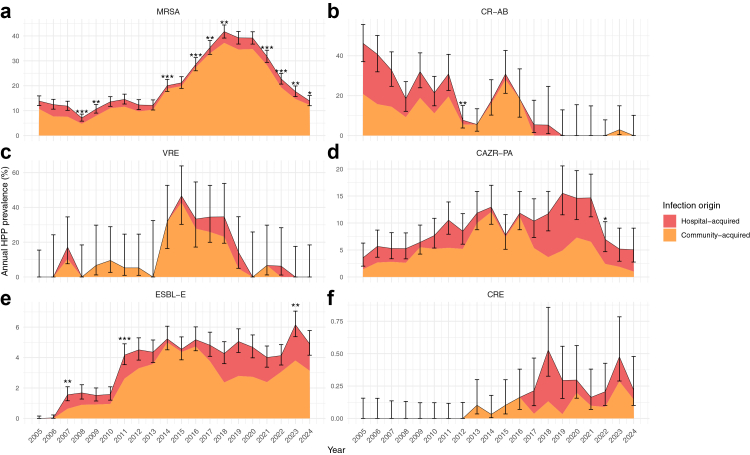
**Annual High Priority Pathogens prevalence: (a) methicillin-resistant *S. aureus*, (b) carbapenem-resistant *A. baumannii*, (c) vancomycin-resistant *E. faecium*, (d) ceftazidime-resistant *P. aeruginosa*, (e) ESBL-Enterobacterales, (f) carbapenemase-producing Enterobacterales, within each relevant bacterial species or group, with annual segregations between hospital-acquired and community-acquired, from 2005 to 2024**. Year-to-year differences were assessed using contrasts from a logistic model: significance refers to comparisons with the immediately preceding year. Differences were considered statistically significant at p < 0.05 (∗), p < 0.001 (∗∗) and p < 0.0001 (∗∗∗).

MRSA was the most prevalent HPP, representing 62.2% [95% CI: 61.2–63.3] (Table S196). Among *S. aureus* isolates, 21.6% [95% CI: 21.1–22.2] were MRSA (Table S197). This prevalence increased from 2014 to 2018, at 41.7% [95% CI: 39.1–44.4], and declined from 2021 to 2024 (Fig. 4).

Across the study period, *A. baumannii* isolates exhibited 21.8% [95% CI: 19.6–24.1] of CR-AB. CR-AB prevalence significantly dropped in 2012 versus 2011 (OR = 0.17, p = 0.001), with 17% decrease in the odds per year (OR = 0.83, p-value <0.001), resulting in a cumulative 97% reduction from 2005 to 2024. Hospital-acquired infections among CR-AB isolates also decreased, from 55.4% [95% CI: 47.4–63.2] in [2005–2008], to 40.6% [95% CI: 31.3–50.6] in [2009–2012], and 10.5% [95% CI: 4.9–21.1] for [2013–2024].

Over 20 years, 16% [95% CI: 12.7–20] of *E*. *faecium* isolates were VRE. Prevalence peaked in 2015 at 46.7% [95% CI: 30.2–63.9]. Hospital-acquisition was involved in 21.1% [95% CI: 12.5–33.3] of VRE.

Among *P. aeruginosa* isolates, 9% [95% CI: 8.3–9.7] were CAZR-PA, and 41.0% [95% CI: 37.0–45.2] of these were hospital-acquired.

Across the 59,954 Enterobacterales isolates, 3.7% [95% CI: 3.5–3.8] (N = 2208) were ESBL. The highest ESBL-E prevalences were observed in *K. pneumoniae* (8.4% [95% CI: 7.9–9]), followed by the *E*. *cloacae complex* (6% [95% CI: 5.4–6.8]), *C*. *freundii* (5.2% [95% CI: 3.8–7.1]), and *S*. *marcescens* (3.8% [95% CI: 2.8–5]) (Table S198). No ESBL-E was detected in 2005 among 2451 Enterobacterales isolates. The first ESBL-E detected was an *E. coli* from a sample collected in December 2006, among 2469 Enterobacterales isolates tested that year. In 2007, 44 ESBL-E isolates were identified (1.6%, [95% CI: 1.2–2.1]) among 2829 isolates, including 22 *K. pneumoniae* and 12 *E. coli*. Since then, ESBL-producing isolates have been identified each year (Table S199). Two phases of increasing prevalence were observed: in 2011, reaching 4.2% [95% CI: 3.5–4.9] (OR = 2.59, p < 0.001), and in 2023, reaching 6.2% [95% CI: 5.4–7.1] (OR = 1.49, p = 0.001). Overall, 29.7% [95% CI: 27.8–31.7] of ESBL-E were hospital-acquired. However, ESBL-E prevalence was higher among hospital-acquired isolates than among community-acquired isolates (χ^2^ test, p < 0.001) (Figure S24).

A very low prevalence of CPE was observed in Enterobacterales (0.1% [95% CI: 0.1–0.2] (N = 85)). *C*. *freundii* had the highest prevalence (1.9% [95% CI: 1.1–3.1]), followed by *S*. *marcescens* (1% [95% CI: 0.6–1.8]), and the *E*. *cloacae complex* (0.7% [95% CI: 0.5–1]) (Table S200, Table S201). CPE showed the highest hospital-acquired proportion among HPP types with 49.4% [95% CI: 39.0–59.8], and CPE prevalence was higher among hospital-acquired isolates than among community-acquired isolates (χ^2^ test, p < 0.001).

Being male was a significant risk factor for five of the six HPP (CR-AB, MRSA, CAZR-PA, ESBL-E, CPE), as was older age (CR-AB, MRSA, VRE, ESBL-E, CPE) (Table S202). Isolation from blood culture was positively associated with ESBL-E (OR = 1.53 [95% CI: 1.35–1.73]), and CPE (OR = 1.78 [95% CI: 1.05–3.02]) but negatively associated with CR-AB (OR = 0.37 [95% CI: 0.24–0.58]) and MRSA (OR = 0.82 [95% CI: 0.73–0.91]). Hospital acquisition was significantly associated with CR-AB, CAZR-PA, ESBL-E, and CPE, whereas community acquisition was significantly associated with VRE.

### Multidrug-resistance

Across 110,205 isolates with at least three different antibiotic classes tested, 12,080 (11% [95% CI: 10.8–11.1] were MDR, comprising 10.4% [95% CI: 10.5–11] in Enterobacterales. According to the more conservative Magiorakos definition, based on non-susceptibility, this proportion reached 14.1% [95% CI: 13.8–14.3].

The penicillins class was the class most involved in MDR, being present in 63.1% [95% CI: 62.3–64.0] of the 12,080 isolates. This was followed by sulfamides (48.2% [95% CI: 47.3–49.0]), aminoglycosides (40.0% [95% CI: 38.8–41.0]), and fluoroquinolones (39.0% [95% CI: 38.1–39.8]). 3 GC represented 30.2% [95% CI: 29.4–31.1] and anti-pseudomonal 3 GC 29.3% [95% CI: 28.4–30.1], so 3 GC as a whole were involved in almost two thirds of MDR isolates. The most frequent combination of classes involved in MDR was penicillins–anti-staphylococcal agents– fusidic acid class, representing 14.2% [95% CI: 13.6–14.8], followed by aminoglycosides–macrolides–lincosamides (5.9% [95% CI: 5.5–6.3]), penicillins–fluoroquinolones–sulfamides (4.9% [95% CI: 4.6–5.3]), and aminoglycosides–penicillins–sulfamides (4.5% [95% CI: 4.1–4.8]).

Among HPP isolates, 67.1% [95% CI: 66.1–68.1] were MDR: from 90.3% to 100% of ESBL-E, VRE, CR-AB, and CPE, 66.5% [95% CI: 62.4–70.3] of CAZR-PA, and 54.8% [95% CI: 53.5–56.2] of MRSA (Table S203).

Averaged over the 20-year study period, the following pathogens had the highest proportion of MDR isolates: *C*. *freundii* (27.4% [95% CI: 24.3–30.7]), *A. baumannii* (25.4% [95% CI: 23.1–27.8]), *E*. *faecium* (24.3% [95% CI: 20.3–28.8]), and *E*. *cloacae complex* (24.1% [95% CI: 22.9–25.4]) (Table S204).

Whereas 9.8% [95% CI: 9.6–10] of community-acquired infections were MDR, 16.4% [95% CI: 15.9–17] of hospital-acquired infections were MDR (χ^2^ test, p < 0.001), reaching a peak of 22.3% [95% CI: 19.7–25.1] in hospital-acquired infections in 2011 (Table S205).

Over time, MDR prevalence varied between 7.4% [95% CI: 6.8–8.1] in 2008 and 14.5% [95% CI: 13.7–15.6] in 2020, with an ongoing decline starting in 2021 (OR = 0.8, p-value = 0.001) (Figure S25, Table S206).

For males, the odds of carrying an MDR isolate were 29% higher than for females (OR = 1.29 [95% CI: 1.24–1.34, p-value <0.001). Each one-year age increase was associated with a 41% increase in the odds of MDR (OR = 1.41 [95% CI: 1.38–1.44], p-value <0.001). Isolates obtained from blood cultures had 15% lower odds of being MDR (OR = 0.85 [95% CI: 0.79–0.91], p-value <0.001), whereas hospital-acquired isolates had 44% higher odds (OR = 1.44 [95% CI: 1.37–1.51], p-value <0.001) (Table S207).

Among isolates from CHT, compared to surgery (the chosen reference), isolates from the geriatrics service had more than twice the odds of being MDR (OR = 2.3 [95% CI: 2.01–2.62], p < 0.001). Isolates from medicine (32% higher odds; OR = 1.32 [95% CI: 1.24–1.4], p < 0.001), pediatrics (32% higher odds; OR = 1.32 [95% CI: 1.18–1.45], p < 0.001), and intensive care (47% higher odds; OR = 1.47 [95% CI: 1.36–1.58], p < 0.001) also showed significantly increased odds of MDR compared to the reference. In contrast, isolates from gynecology had 37% lower odds of being MDR (OR = 0.63 [95% CI: 0.55–0.71], p < 0.001) (Table S08).

### Extensive drug-resistance

Of the 33,175 isolates across 16 pathogens with a sufficient number of different classes tested, 188 were pXDR (0.6% [95% CI: 0.5–0.6]) (Table S209), all were MDR and 35.8% [95% CI: 29.3–42.9] were hospital-acquired. According to the more conservative Magiorakos definition, based on non-susceptibility, this proportion reached 1% [95% CI: 0.9–1.1].

Pathogens with highest pXDR prevalence were *E*. *cloacae* complex (5.1% [95% CI: 3.6–7.2]), *Stenotrophomonas maltophilia* (2.6% [95% CI: 1.6–4.2]), and *K*. *pneumoniae* (2.1% [95% CI: 1.5–3]) (Table S210).

## Discussion

This 20-year laboratory-based sentinel surveillance provides a comprehensive overview of AMR dynamics in New Caledonia, documenting both the emergence and decline of resistance across 111,022 clinical isolates. Notable emergences were observed for resistance to β-lactams (penicillins, 3 GC), tetracycline, and fusidic acid. Past outbreaks of MRSA, and CR-AB have been identified, alongside the emergence of ESBL-E and CPE. Overall, MDR affected 11% of isolates and was consistently associated with male sex, older age, and hospital-acquired infections, in line with global epidemiological patterns.

Resistance to penicillin and tetracycline progressively increased from initial full susceptibility for *N. gonorrhoeae*, reaching 26% and 72%, respectively, by 2024. High levels of resistance have also been reported in Australia, with 40% of gonococcal isolates resistant to penicillin in 2022 and 45% resistant to tetracycline.^21^ Resistance to other penicillins also emerged, with MRSA prevalence increasing from 2014 to 2018 and subsequently declining after 2021. This temporal pattern is consistent with the introduction and subsequent decline of the CC6-MRSA-[IV + fus] (PVL+) clone, which also drove increased fusidic acid resistance in *S. aureus*.^6^

Similarly, the CR-AB outbreak observed in New Caledonia was driven by the OXA-23 clone, previously identified locally from 2004^5^ and in Tahiti,^22^ another French territory in the Pacific Ocean. A related strain was isolated in Montpellier in 1999 from a patient directly transferred from Papeete, suggesting that this clone, or a closely related one, was already circulating in French Polynesia several years before the 2004 outbreak. CR-AB prevalence declined throughout the study period, reaching zero or near-zero from 2019 onward, whereas the global proportion increased between 2018 and 2023 to an estimated 54% resistant to imipenem in 2023.^4^ CR-AB ranked first on the 2017 WHO priority pathogens list but third in 2024.

Resistance to 3GCs increased among Enterobacterales isolates, mainly due to rising cefotaxime resistance in *E. coli* from 2010, stabilizing around 6%. This remains lower than in Australia (12%), and comparable to New Zealand (8%), in 2017.^23^ This 3 GC resistance trend paralleled the emergence of ESBL-E and CPE. Although only 3% of *E. coli* were ESBL, this proportion reached nearly 20% in Australia in 2018–2019, 10% in New Zealand,^24^ and 18% in Vanuatu.^25^ In one hospital in France during 2013–2016, 41% of *E. coli* and 36% of *K. pneumoniae* were ESBL producers, whereas in New Caledonia only 8% of *K. pneumoniae* were ESBL.^26^ In Fiji, during 2011–2012, a hospital detected 22% of *K. pneumoniae* as ESBL producers.^27^ In New Caledonia, CPE undetected until 2013, have since represented 0.25–0.5% of isolates. This pattern is consistent with the emergence, detected in 2013, and the persistent spread of an IMP-4 IncM2 plasmid, with evidence of cross-border exchange with Australia, notably through medevac patient transfers.^11^ Although this prevalence remains low,^11^ during 2017–2019, 1% of *E. coli* globally were CPE, and 3% in the Asia–Pacific region.^28^

In the published literature from PICTs, the earliest identifiable ESBL-E report appears to come from New Caledonia and involved *K. pneumoniae* from 2008 onward. By contrast, our data suggest an earlier occurrence, with an ESBL-*E. coli* isolate detected in December 2006, although its provenance could not be determined.^29^

Additionally, 90% of ESBL-E and 100% of CPE were MDR, but only 10% of Enterobacterales overall. MDR was strongly associated with emergence of ESBL-E and CPE, as these pathogens frequently carry combinations of β-lactamases and additional mechanisms such as porin loss or efflux pumps, resulting in MDR phenotypes.^30^ Similar patterns have been identified for CAZR-PA,^31^ CR-AB,^32^ and VRE,^33^ all of which are strongly associated with MDR. While MDR was observed in 11% of isolates overall, it was only 1% for *N. gonorrhoeae* and the XDR rate was zero, whereas higher levels were reported in some Asia–Pacific countries, such as Cambodia in 2022–2023, where 6% of isolates were XDR.^34^ Consistent with previous studies, MDR has been associated with hospital-acquired infections.^26^^,^^35^^,^^36^ Indeed, hospitals with diverse bacterial populations can serve as hotspots of horizontal gene transfer and act as incubators for MDR,^37^ through transmission between patients as well as via contaminated surfaces such as sinks and drains.^38^ Older age has previously been identified as a risk factor for MDR carriage, likely reflecting immunosenescence, multimorbidity, repeated healthcare exposure, and cumulative antibiotic use.^39^ Male sex has also been associated with MDR in some settings, although this association should be interpreted more cautiously, as it may reflect both biological sex-related immune differences and differences in healthcare exposure or other unmeasured factors.^40^^,^^41^

Despite documented episodes of resistance emergence and clonal spread, AMR prevalence in New Caledonia generally remains lower than in well-connected territories such as Australia, with extensive business and tourist exchanges and patient transfers, and mainland France. Geographic remoteness and relative insularity may prevent or delay the introduction of resistant clones, including MDR strains. Nevertheless, clonal invasions have already been documented,^6^^,^^22^ and continuous surveillance remains essential.

The long 20-year collection period enabled a large number of isolates to be gathered, thus providing a comprehensive picture of the past and current situation of AMR in New Caledonia. De-duplication, recognized as a challenge in AMR epidemiological studies,^42^ minimized overestimation of resistance rates. However, annual resistance rates can be affected by the modifiable temporal unit problem, since events at the end of one year and the beginning of the next may be closely related.^43^

The main limitation of the study is an unknown denominator for all antibiograms performed in New Caledonia, constraining extrapolation, and a sampling frame that predominantly covers the southern part of the country. However, this area is by far the most densely populated, and the CHT performed more than half of all antibiograms conducted in New Caledonia. In addition, the most severe cases treated at or referred to the CHT may have led to an overestimation of resistance rates, as these patients are more likely to have complex clinical histories and previous healthcare exposure. Additionally, in the absence of consensus across all bacterial species on the antibiotics to include, and with some agents untested, XDR status could be assessed only as possible. Classification as MDR, defined as resistance to at least three antimicrobial classes, does not necessarily imply resistance to first-line agents only, but rather reflects the accumulation of acquired resistance across multiple antimicrobial classes.

In addition, EUCAST guideline changes may cause apparent year-to-year fluctuations despite unchanged susceptibility. The apparent increase in piperacillin–tazobactam resistance in 2013 could be related to the reduction of the breakpoint from 64 to 16 mg/L in 2011,^44^ while the rise in 3 GC resistance reported in 2009 may be attributable to the 2008 CASFM update,^45^ which lowered the breakpoint from 32 to 2 mg/L. Methodological factors might also have contributed to these observations; for instance, the use of mini-API *Pseudomonas* panels (ATB PSE EU08) for *Stenotrophomonas* until 2020, despite their limited reliability, may have led to an apparent overestimation of *S. maltophilia* resistance to some antibiotics. Accordingly, phenotypic identification of ESBL-E and CPE also changed slightly over the study period, following successive updates in EUCAST recommendations.

Furthermore, as EUCAST is a European standard, comparisons may be biased by differences in breakpoints, particularly with CDS used in Australia and New Zealand and CLSI widely adopted worldwide.^46^ Indeed, EUCAST is often more stringent, thereby tending to classify a higher proportion of isolates as resistant.^47^

Additional limitations include the fact that the rule classifying infections as hospital-acquired when the sample is taken more than two days after hospital admission may have introduced misclassification bias. An overestimation was observed for gonococcal infections, a small minority of which were incorrectly classified as nosocomial despite their sexually transmitted nature. Conversely, hospital-acquired infections may have been underestimated for a minority of inpatients managed outside CHT when the admission date was unknown.

This work has identified key areas for vigilance, notably Enterobacterales, especially ESBL-E, together with *N. gonorrhoeae* and its acquisition of resistance to penicillin and tetracyclines, even though it is not yet significantly affected by MDR. Ongoing risks include the introduction of new clones and the spread of MDR, especially in hospitals.

Surveillance of resistance patterns at CHT has already led to adaptations of empiric treatment guidelines in New Caledonia. For example, penicillin G was historically used to treat gonococcal infections, but the emergence of resistance and associated clinical failures led to a switch to third-generation cephalosporins. Similarly, for severe *S. aureus* infections, the perceived burden of MRSA between 2020 and 2025 supported the upfront use of vancomycin in first line sepsis treatment. These changes are documented in successive local antibiotic guidelines issued in New Caledonia in 2016, 2020, and 2024.^48^

In conclusion, this 20-year surveillance study provides a unique and policy-relevant overview of AMR dynamics in New Caledonia. While resistance, MDR emergence and clonal spread have undeniably occurred, overall prevalence remains lower than in comparable regions, and effective control have been achieved for major threats such as CR-AB and MRSA. These findings emphasize the need for continued and strengthened surveillance, particularly in hospital settings where MDR pathogens tend to emerge and spread most readily. Maintaining long-term monitoring is essential to anticipate new resistance threats, to guide local and regional antimicrobial stewardship policies, and to update recommendations for first-line treatments.

## Contributors

JC and CG conceptualized the study. AA and JC curated the data. AA performed the formal statistical analysis and prepared the visualizations. JC, TR, AnBi, AlBo, and AA contributed to the investigation. JC, CG, and AA developed the methodology. JC and CG provided resources and supervised the study. Funding was acquired by JC, CG, and CF. JC validated the analyses. AA wrote the original draft. All authors critically reviewed and edited the manuscript and approved the final version. AA and JC accessed and verified the data. JC and CG were responsible for the decision to submit the manuscript.

## Data sharing statement

Data sharing may be possible upon consultation with the corresponding author, subject to approval by all authors.

## Editor note

This translated Abstract in French was submitted by the authors and we reproduce it as supplied. It has not been peer reviewed. Our editorial processes have only been applied to the original abstract in English, which should serve as reference for this manuscript.

## Declaration of interests

We have no conflicts of interest to declare.

## Appendix A Supplementary data

Translated Abstract
