# Supplementary material for: Emergence of antimicrobial resistance in New Caledonia: 20-year trends from laboratory-based surveillance (2005–2024)
Source: Lancet Reg Health West Pac. 2026 Jul 3;72:101913. doi: 10.1016/j.lanwpc.2026.101913 (PMC13351301; doi:10.1016/j.lanwpc.2026.101913)
Supplement: Translated Abstract [file mmc1.docx]

**Résumé**

Contexte

Des problématiques de résistance aux antimicrobiens ont émergé en Nouvelle-Calédonie, notamment avec une augmentation des infections à *Staphylococcus aureus* résistant à la méticilline (SARM) et à *Acinetobacter baumannii* résistant à l’imipénème (ABRI). Toutefois, un panorama global reste nécessaire pour guider le bon usage des antimicrobiens.

Méthodes

Une collecte prospective des résultats d’antibiogrammes a été menée sur 20 ans (2005–2024) au Centre Hospitalier Territorial de Nouvelle-Calédonie. Cette surveillance portait sur 22 pathogènes et 28 agents antimicrobiens, couvrant 157 combinaisons pathogène-antibiotique. Les tendances de résistance ont été analysées à l’aide de modèles logistiques. Une attention particulière a été portée aux pathogènes résistants hautement prioritaires, incluant SARM, ABRI, *Enterococcus faecium* résistant à la vancomycine, *Pseudomonas aeruginosa* résistant à la ceftazidime, les entérobactéries productrices de β-lactamases à spectre étendu (E-BLSE) et les entérobactéries productrices de carbapénèmases (EPC). Les isolats multirésistants (MDR) et possiblement à résistance étendue (pXDR) ont également été identifiés et caractérisés.

Résultats

Parmi les 111 022 isolats inclus, des émergences de résistances ont été observées, notamment aux pénicillines, aux céphalosporines de troisième génération, à la tétracycline et à l’acide fusidique. Les tendances temporelles de la résistance ont été décrites, ainsi que les épidémies de SARM et d’ABRI, et l’émergence des E-BLSE et des EPC, qui représentaient respectivement 3,7% [IC95% : 3,5–3,8] (N=2 208) et 0,1% [IC95% : 0,1–0,2] (N=85) des 59 800 isolats d’entérobactéries. Au total, 11% [IC95% : 10,8–11,1] des isolats étaient MDR et 0,6% [IC95% : 0,5–0,6] étaient pXDR.

Interprétation

L’analyse des tendances temporelles a mis en évidence des dynamiques variables de résistance aux antimicrobiens selon les pathogènes bactériens, incluant des augmentations comme des diminutions. Ces résultats contribuent à identifier les antibiotiques pouvant conserver leur place parmi les options de première ligne, tout en répondant au manque de données disponibles dans les territoires insulaires du Pacifique.

Financement

Ce travail a bénéficié du soutien financier de la bourse Pierre Ledoux Jeunesse Internationale (Fondation de France) et de l’Institut Pasteur, Paris.
