# Supplementary material for: Emergence of antimicrobial resistance in New Caledonia: 20-year trends from laboratory-based surveillance (2005–2024)
Source: Lancet Reg Health West Pac. 2026 Jul 3;72:101913. doi: 10.1016/j.lanwpc.2026.101913 (PMC13351301; doi:10.1016/j.lanwpc.2026.101913)
Supplement: Supplementary Files [file mmc2.docx]

Table of Contents

[A) Description 1](#_Toc228977387)

[Table S1: List of the 157 pathogen-drug combinations. 1](#_Toc228977388)

[Table S2: Susceptibility and resistance imputation rules. 4](#_Toc228977389)

[Table S3: Imputation rules for High-Priority Pathogen (HPP) in New Caledonia. 5](#_Toc228977390)

[Table S4: Antibiotic classes used for multidrug (MDR) and possible extensively-drug resistance (pXDR). 5](#_Toc228977391)

[Table S5: Major classes used to identify XDR. 7](#_Toc228977392)

[Table S6: Intrinsic resistance. 9](#_Toc228977393)

[Table S7: Data management by species or genera. 13](#_Toc228977394)

[Table S8: Number of patients, samples, and isolates per year. 14](#_Toc228977395)

[Table S9: Prevalence of each bacterial species or genera among the total number of isolates. 15](#_Toc228977396)

[B) Enterobacterales and key antimicrobial agents 16](#_Toc228977397)

[Table S10: Logistic regression analysis of the effect of continuous time on antibiotic resistance of Enterobacterales from 2005 to 2024 16](#_Toc228977398)

[Table S11: Annual percentage of Enterobacterales resistance to Aminoglycosides from 2005 to 2024. 16](#_Toc228977399)

[Table S12: Annual percentage of Enterobacterales resistance to Fluoroquinolones from 2005 to 2024. 17](#_Toc228977400)

[Table S13: Annual percentage of Enterobacterales resistance to Third-generation cephalosporins from 2005 to 2024. 18](#_Toc228977401)

[Table S14: Annual percentage of Enterobacterales resistance to Imipenem from 2005 to 2024. 18](#_Toc228977402)

[Table S15: Annual percentage of Enterobacterales resistance to Cotrimoxazole from 2005 to 2024. 19](#_Toc228977403)

[Table S16: Annual percentage of Enterobacterales resistance to Piperacillin/Tazobactam from 2005 to 2024. 20](#_Toc228977404)

[C) 157 pathogen-drug combinations 21](#_Toc228977405)

[Enterobacterales 21](#_Toc228977406)

[Figure S1: Annual percentage of *Citrobacter freundii* resistance from 2005 to 2024 21](#_Toc228977407)

[Table S17: Logistic regression analysis of the effect of continuous time on the antibiotic resistance of *Citrobacter freundii* from 2005 to 2024. 21](#_Toc228977408)

[Table S18: Annual percentage of *Citrobacter freundii* resistance to Amikacin from 2005 to 2024. 22](#_Toc228977409)

[Table S19: Annual percentage of *Citrobacter freundii* resistance to Ceftazidime from 2005 to 2024. 23](#_Toc228977410)

[Table S20: Annual percentage of *Citrobacter freundii* resistance to Ciprofloxacin from 2005 to 2024. 24](#_Toc228977411)

[Table S21: Annual percentage of *Citrobacter freundii* resistance to Cefotaxime from 2005 to 2024. 25](#_Toc228977412)

[Table S22: Annual percentage of *Citrobacter freundii* resistance to Gentamicin from 2005 to 2024. 26](#_Toc228977413)

[Table S23: Annual percentage of *Citrobacter freundii* resistance to Imipenem from 2005 to 2024. 27](#_Toc228977414)

[Table S24: Annual percentage of *Citrobacter freundii* resistance to Cotrimoxazole from 2005 to 2024. 28](#_Toc228977415)

[Table S25: Annual percentage of *Citrobacter freundii* resistance to Piperacillin/Tazobactam from 2005 to 2024. 29](#_Toc228977416)

[Figure S2: Annual percentage of *Citrobacter kosseri* resistance from 2005 to 2024 30](#_Toc228977417)

[Table S26: Logistic regression analysis of the effect of continuous time on the antibiotic resistance of *Citrobacter kosseri* from 2005 to 2024. 30](#_Toc228977418)

[Table S27: Annual percentage of *Citrobacter kosseri* resistance to Amikacin from 2005 to 2024. 31](#_Toc228977419)

[Table S28: Annual percentage of *Citrobacter kosseri* resistance to Amoxicillin/Clavulanic Acid from 2005 to 2024. 31](#_Toc228977420)

[Table S29: Annual percentage of *Citrobacter kosseri* resistance to Ceftazidime from 2005 to 2024. 32](#_Toc228977421)

[Table S30: Annual percentage of *Citrobacter kosseri* resistance to Ciprofloxacin from 2005 to 2024. 33](#_Toc228977422)

[Table S31: Annual percentage of *Citrobacter kosseri* resistance to Cefotaxime from 2005 to 2024. 34](#_Toc228977423)

[Table S32: Annual percentage of *Citrobacter kosseri* resistance to Gentamicin from 2005 to 2024. 35](#_Toc228977424)

[Table S33: Annual percentage of *Citrobacter kosseri* resistance to Imipenem from 2005 to 2024. 36](#_Toc228977425)

[Table S34: Annual percentage of *Citrobacter kosseri* resistance to Cotrimoxazole from 2005 to 2024. 36](#_Toc228977426)

[Table S35: Annual percentage of *Citrobacter kosseri* resistance to Piperacillin/Tazobactam from 2005 to 2024. 37](#_Toc228977427)

[Figure S3: Annual percentage of *Escherichia coli* resistance from 2005 to 2024 39](#_Toc228977428)

[Table S36: Logistic regression analysis of the effect of continuous time on the antibiotic resistance of *Escherichia coli* from 2005 to 2024. 39](#_Toc228977429)

[Table S37: Annual percentage of *Escherichia coli* resistance to Amikacin from 2005 to 2024. 40](#_Toc228977430)

[Table S38: Annual percentage of *Escherichia coli* resistance to Amoxicillin/Clavulanic Acid from 2005 to 2024. 41](#_Toc228977431)

[Table S39: Annual percentage of *Escherichia coli* resistance to Aminopenicillin from 2005 to 2024. 41](#_Toc228977432)

[Table S40: Annual percentage of *Escherichia coli* resistance to Ceftazidime from 2005 to 2024. 42](#_Toc228977433)

[Table S41: Annual percentage of *Escherichia coli* resistance to Ciprofloxacin from 2005 to 2024. 43](#_Toc228977434)

[Table S42: Annual percentage of *Escherichia coli* resistance to Cefotaxime from 2005 to 2024. 44](#_Toc228977435)

[Table S43: Annual percentage of *Escherichia coli* resistance to Gentamicin from 2005 to 2024. 45](#_Toc228977436)

[Table S44: Annual percentage of *Escherichia coli* resistance to Imipenem from 2005 to 2024. 45](#_Toc228977437)

[Table S45: Annual percentage of *Escherichia coli* resistance to Cotrimoxazole from 2005 to 2024. 46](#_Toc228977438)

[Table S46: Annual percentage of *Escherichia coli* resistance to Piperacillin/Tazobactam from 2005 to 2024. 47](#_Toc228977439)

[Figure S4: Annual percentage of *Enterococcus cloacae* complex resistance from 2005 to 2024 48](#_Toc228977440)

[Table S47: Logistic regression analysis of the effect of continuous time on the antibiotic resistance of *Enterococcus cloacae* complex from 2005 to 2024. 49](#_Toc228977441)

[Table S48: Annual percentage of *Enterococcus cloacae* complex resistance to Amikacin from 2005 to 2024. 49](#_Toc228977442)

[Table S49: Annual percentage of *Enterococcus cloacae* complex resistance to Ceftazidime from 2005 to 2024. 50](#_Toc228977443)

[Table S50: Annual percentage of *Enterococcus cloacae* complex resistance to Ciprofloxacin from 2005 to 2024. 51](#_Toc228977444)

[Table S51: Annual percentage of *Enterococcus cloacae* complex resistance to Cefotaxime from 2005 to 2024. 51](#_Toc228977445)

[Table S52: Annual percentage of *Enterococcus cloacae* complex resistance to Gentamicin from 2005 to 2024. 52](#_Toc228977446)

[Table S53: Annual percentage of *Enterococcus cloacae* complex resistance to Imipenem from 2005 to 2024. 53](#_Toc228977447)

[Table S54: Annual percentage of *Enterococcus cloacae* complex resistance to Cotrimoxazole from 2005 to 2024. 54](#_Toc228977448)

[Table S55: Annual percentage of *Enterococcus cloacae* complex resistance to Piperacillin/Tazobactam from 2005 to 2024. 55](#_Toc228977449)

[Figure S5: Annual percentage of *Klebsiella pneumoniae* resistance from 2005 to 2024 56](#_Toc228977450)

[Table S56: Logistic regression analysis of the effect of continuous time on the antibiotic resistance of *Klebsiella pneumoniae* from 2005 to 2024. 56](#_Toc228977451)

[Table S57: Annual percentage of *Klebsiella pneumoniae* resistance to Amoxicillin/Clavulanic Acid from 2005 to 2024. 57](#_Toc228977452)

[Table S58: Annual percentage of *Klebsiella pneumoniae* resistance to Amikacin from 2005 to 2024. 57](#_Toc228977453)

[Table S59: Annual percentage of *Klebsiella pneumoniae* resistance to Ceftazidime from 2005 to 2024. 58](#_Toc228977454)

[Table S60: Annual percentage of *Klebsiella pneumoniae* resistance to Ciprofloxacin from 2005 to 2024. 59](#_Toc228977455)

[Table S61: Annual percentage of *Klebsiella pneumoniae* resistance to Cefotaxime from 2005 to 2024. 60](#_Toc228977456)

[Table S62: Annual percentage of *Klebsiella pneumoniae* resistance to Gentamicin from 2005 to 2024. 61](#_Toc228977457)

[Table S63: Annual percentage of *Klebsiella pneumoniae* resistance to Imipenem from 2005 to 2024. 62](#_Toc228977458)

[Table S64: Annual percentage of *Klebsiella pneumoniae* resistance to Cotrimoxazole from 2005 to 2024. 62](#_Toc228977459)

[Table S65: Annual percentage of *Klebsiella pneumoniae* resistance to Piperacillin/Tazobactam from 2005 to 2024. 63](#_Toc228977460)

[Table S66: Logistic regression analysis of the effect of continuous time on the antibiotic resistance of *Morganella morganii* from 2005 to 2024. 65](#_Toc228977461)

[Table S67: Annual percentage of *Morganella morganii* resistance to Amikacin from 2005 to 2024. 66](#_Toc228977462)

[Table S68: Annual percentage of *Morganella morganii* resistance to Ceftazidime from 2005 to 2024. 66](#_Toc228977463)

[Table S69: Annual percentage of *Morganella morganii* resistance to Ciprofloxacin from 2005 to 2024. 67](#_Toc228977464)

[Table S70: Annual percentage of *Morganella morganii* resistance to Cefotaxime from 2005 to 2024. 68](#_Toc228977465)

[Table S71: Annual percentage of *Morganella morganii* resistance to Gentamicin from 2005 to 2024. 69](#_Toc228977466)

[Table S72: Annual percentage of *Morganella morganii* resistance to Imipenem from 2005 to 2024. 70](#_Toc228977467)

[Table S73: Annual percentage of *Morganella morganii* resistance to Cotrimoxazole from 2005 to 2024. 70](#_Toc228977468)

[Table S74: Annual percentage of *Morganella morganii* resistance to Piperacillin/Tazobactam from 2005 to 2024. 71](#_Toc228977469)

[Figure S7: Annual percentage of *Proteus mirabilis* resistance from 2005 to 2024 73](#_Toc228977470)

[Table S75: Logistic regression analysis of the effect of continuous time on the antibiotic resistance of *Proteus mirabilis* from 2005 to 2024. 73](#_Toc228977471)

[Table S76: Annual percentage of *Proteus mirabilis* resistance to Amikacin from 2005 to 2024. 74](#_Toc228977472)

[Table S77: Annual percentage of *Proteus mirabilis* resistance to Aminopenicillin from 2005 to 2024. 75](#_Toc228977473)

[Table S78: Annual percentage of *Proteus mirabilis* resistance to Amoxicillin/Clavulanic Acid from 2005 to 2024. 75](#_Toc228977474)

[Table S79: Annual percentage of *Proteus mirabilis* resistance to Ceftazidime from 2005 to 2024. 76](#_Toc228977475)

[Table S80: Annual percentage of *Proteus mirabilis* resistance to Ciprofloxacin from 2005 to 2024. 77](#_Toc228977476)

[Table S81: Annual percentage of *Proteus mirabilis* resistance to Cefotaxime from 2005 to 2024. 78](#_Toc228977477)

[Table S82: Annual percentage of *Proteus mirabilis* resistance to Gentamicin from 2005 to 2024. 79](#_Toc228977478)

[Table S83: Annual percentage of *Proteus mirabilis* resistance to Cotrimoxazole from 2005 to 2024. 79](#_Toc228977479)

[Table S84: Annual percentage of *Proteus mirabilis* resistance to Piperacillin/Tazobactam from 2005 to 2024. 80](#_Toc228977480)

[Figure S8: Annual percentage of *Salmonella spp.* resistance from 2005 to 2024. 82](#_Toc228977481)

[Table S85: Logistic regression analysis of the effect of continuous time on the antibiotic resistance of *Salmonella spp.* from 2005 to 2024. 82](#_Toc228977482)

[Table S86: Annual percentage of *Salmonella spp.* resistance to Aminopenicillin from 2005 to 2024. 83](#_Toc228977483)

[Table S87: Annual percentage of *Salmonella spp.* resistance to Amoxicillin/Clavulanic Acid from 2005 to 2024. 83](#_Toc228977484)

[Table S88: Annual percentage of *Salmonella spp.* resistance to Ceftazidime from 2005 to 2024. 84](#_Toc228977485)

[Table S89: Annual percentage of *Salmonella spp.* resistance to Ciprofloxacin from 2005 to 2024. 85](#_Toc228977486)

[Table S90: Annual percentage of *Salmonella spp.* resistance to Cefotaxime from 2005 to 2024. 86](#_Toc228977487)

[Table S91: Annual percentage of *Salmonella spp.* resistance to Imipenem from 2005 to 2024. 87](#_Toc228977488)

[Table S92: Annual percentage of *Salmonella spp.* resistance to Cotrimoxazole from 2005 to 2024. 87](#_Toc228977489)

[Figure S9: Annual percentage of *Shigella spp.* resistance from 2005 to 2024. 89](#_Toc228977490)

[Table S93: Logistic regression analysis of the effect of continuous time on the antibiotic resistance of *Shigella spp.* from 2005 to 2024. 89](#_Toc228977491)

[Table S94: Annual percentage of *Shigella spp.* resistance to Amikacin from 2005 to 2024. 90](#_Toc228977492)

[Table S95: Annual percentage of *Shigella spp.* resistance to Aminopenicillin from 2005 to 2024. 90](#_Toc228977493)

[Table S96: Annual percentage of *Shigella spp.* resistance to Amoxicillin/Clavulanic Acid from 2005 to 2024. 91](#_Toc228977494)

[Table S97: Annual percentage of *Shigella spp.* resistance to Ceftazidime from 2005 to 2024. 92](#_Toc228977495)

[Table S98: Annual percentage of *Shigella spp.* resistance to Ciprofloxacin from 2005 to 2024. 92](#_Toc228977496)

[Table S99: Annual percentage of *Shigella spp.* resistance to Cefotaxime from 2005 to 2024. 93](#_Toc228977497)

[Table S100: Annual percentage of *Shigella spp.* resistance to Gentamicin from 2005 to 2024. 94](#_Toc228977498)

[Table S101: Annual percentage of *Shigella spp.* resistance to Imipenem from 2005 to 2024. 94](#_Toc228977499)

[Table S102: Annual percentage of *Shigella spp.* resistance to Imipenem from 2005 to 2024. 95](#_Toc228977500)

[Table S103: Annual percentage of *Shigella spp.* resistance to Cotrimoxazole from 2005 to 2024. 96](#_Toc228977501)

[Figure S10: Annual percentage of *Serratia marcescens* resistance from 2005 to 2024. 97](#_Toc228977502)

[Table S104: Logistic regression analysis of the effect of continuous time on the antibiotic resistance of *Serratia marcescens* from 2005 to 2024. 97](#_Toc228977503)

[Table S105: Annual percentage of *Serratia marcescens* resistance to Ceftazidime from 2005 to 2024. 98](#_Toc228977504)

[Table S106: Annual percentage of *Serratia marcescens* resistance to Ciprofloxacin from 2005 to 2024. 98](#_Toc228977505)

[Table S107: Annual percentage of *Serratia marcescens* resistance to Cefotaxime from 2005 to 2024. 99](#_Toc228977506)

[Table S108: Annual percentage of *Serratia marcescens* resistance to Gentamicin from 2005 to 2024. 100](#_Toc228977507)

[Table S109: Annual percentage of *Serratia marcescens* resistance to Imipenem from 2005 to 2024. 101](#_Toc228977508)

[Table S110: Annual percentage of *Serratia marcescens* resistance to Cotrimoxazole from 2005 to 2024. 102](#_Toc228977509)

[Table 111: Annual percentage of *Serratia marcescens* resistance to Piperacillin/Tazobactam from 2005 to 2024. 103](#_Toc228977510)

[Enterococci 104](#_Toc228977511)

[Figure S11: Annual percentage of *Enterococcus faecium* resistance from 2005 to 2024. 104](#_Toc228977512)

[Table S112: Logistic regression analysis of the effect of continuous time on the antibiotic resistance of *Enterococcus faecium* from 2005 to 2024. 104](#_Toc228977513)

[Table S113: Annual percentage of *Enterococcus faecium* resistance to Aminopenicillin from 2005 to 2024. 105](#_Toc228977514)

[Table S114: Annual percentage of *Enterococcus faecium* resistance to Erythromycin from 2005 to 2024. 105](#_Toc228977515)

[Table S115: Annual percentage of *Enterococcus faecium* resistance to Gentamicin from 2005 to 2024. 106](#_Toc228977516)

[Table S116: Annual percentage of *Enterococcus faecium* resistance to Linezolid from 2005 to 2024. 107](#_Toc228977517)

[Table S117: Annual percentage of *Enterococcus faecium* resistance to Teicoplanin from 2005 to 2024. 108](#_Toc228977518)

[Table S118: Annual percentage of *Enterococcus faecium* resistance to Vancomycin from 2005 to 2024. 109](#_Toc228977519)

[Figure S12: Annual percentage of *Enterococcus faecalis* resistance from 2005 to 2024. 110](#_Toc228977520)

[Table S119: Logistic regression analysis of the effect of continuous time on the antibiotic resistance of *Enterococcus faecalis* from 2005 to 2024. 110](#_Toc228977521)

[Table S120: Annual percentage of *Enterococcus faecalis* resistance to Erythromycin from 2005 to 2024. 111](#_Toc228977522)

[Table S121: Annual percentage of *Enterococcus faecalis* resistance to Gentamicin from 2005 to 2024. 111](#_Toc228977523)

[Table S122: Annual percentage of *Enterococcus faecalis* resistance to Linezolid from 2005 to 2024. 112](#_Toc228977524)

[Table S123: Annual percentage of *Enterococcus faecalis* resistance to Teicoplanin from 2005 to 2024. 113](#_Toc228977525)

[Table S124: Annual percentage of *Enterococcus faecalis* resistance to Vancomycin from 2005 to 2024. 114](#_Toc228977526)

[Mycobacteria 115](#_Toc228977527)

[Figure S13: Annual percentage of *Mycobacterium tuberculosis* resistance from 2005 to 2024. 115](#_Toc228977528)

[Table S125: Logistic regression analysis of the effect of continuous time on the antibiotic resistance of *Mycobacterium tuberculosis* from 2005 to 2024. 116](#_Toc228977529)

[Table S126: Annual percentage of *Mycobacterium tuberculosis* resistance to Ethambutol from 2005 to 2024. 116](#_Toc228977530)

[Table S127: Annual percentage of *Mycobacterium tuberculosis* resistance to Isoniazid from 2005 to 2024. 117](#_Toc228977531)

[Table S128: Annual percentage of *Mycobacterium tuberculosis* resistance to Rifampicin from 2005 to 2024. 117](#_Toc228977532)

[Table S129: Annual percentage of *Mycobacterium tuberculosis* resistance to Streptomycin from 2005 to 2024. 118](#_Toc228977533)

[Non-fermenters 120](#_Toc228977534)

[Figure S14: Annual percentage of *Acinetobacter baumannii* resistance from 2005 to 2024. 120](#_Toc228977535)

[Table S130: Logistic regression analysis of the effect of continuous time on the antibiotic resistance of *Acinetobacter baumannii* from 2005 to 2024. 120](#_Toc228977536)

[Table S131: Annual percentage of *Acinetobacter baumannii* resistance to Ceftazidime from 2005 to 2024. 121](#_Toc228977537)

[Table S132: Annual percentage of *Acinetobacter baumannii* resistance to Ciprofloxacin from 2005 to 2024. 121](#_Toc228977538)

[Table S133: Annual percentage of *Acinetobacter baumannii* resistance to Gentamycin from 2005 to 2024. 122](#_Toc228977539)

[Table S134: Annual percentage of *Acinetobacter baumannii* resistance to Imipenem from 2005 to 2024. 123](#_Toc228977540)

[Table S135: Annual percentage of *Acinetobacter baumannii* resistance to Cotrimoxazole from 2005 to 2024. 124](#_Toc228977541)

[Table S136: Annual percentage of *Acinetobacter baumannii* resistance to Piperacillin/Tazobactam from 2005 to 2024. 125](#_Toc228977542)

[Figure S15: Annual percentage of *Stenotrophomonas maltophilia* resistance from 2005 to 2024. 126](#_Toc228977543)

[Table S137: Logistic regression analysis of the effect of continuous time on the antibiotic resistance of *Stenotrophomonas maltophilia* from 2005 to 2024. 126](#_Toc228977544)

[Table S138: Annual percentage of *Stenotrophomonas maltophilia* resistance to Ceftazidime from 2005 to 2024. 127](#_Toc228977545)

[Table 139: Annual percentage of *Stenotrophomonas maltophilia* resistance to Levofloxacin from 2005 to 2024. 127](#_Toc228977546)

[Table S140: Annual percentage of *Stenotrophomonas maltophilia* resistance to Ticarcillin/Clavulanic Acid from 2005 to 2024. 128](#_Toc228977547)

[Table S141: Annual percentage of *Stenotrophomonas maltophilia* resistance to Cotrimoxazole from 2005 to 2024. 129](#_Toc228977548)

[Figure S16: Annual percentage of *Pseudomonas aeruginosa* resistance from 2005 to 2024 130](#_Toc228977549)

[Table S142: Logistic regression analysis of the effect of continuous time on the antibiotic resistance of *Pseudomonas aeruginosa* from 2005 to 2024. 131](#_Toc228977550)

[Table S143: Annual percentage of *Pseudomonas aeruginosa* resistance to Amikacin from 2005 to 2024. 131](#_Toc228977551)

[Table S144: Annual percentage of *Pseudomonas aeruginosa* resistance to Ceftazidime from 2005 to 2024. 132](#_Toc228977552)

[Table S145: Annual percentage of *Pseudomonas aeruginosa* resistance to Ciprofloxacin from 2005 to 2024. 133](#_Toc228977553)

[Table S146: Annual percentage of *Pseudomonas aeruginosa* resistance to Gentamicin from 2005 to 2024. 133](#_Toc228977554)

[Table S147: Annual percentage of *Pseudomonas aeruginosa* resistance to Imipenem from 2005 to 2024. 134](#_Toc228977555)

[Table S148: Annual percentage of *Pseudomonas aeruginosa* resistance to Piperacillin/Tazobactam from 2005 to 2024. 135](#_Toc228977556)

[Other Gram-Negatives 136](#_Toc228977557)

[Figure S17: Annual percentage of *Haemophilus influenzae* resistance from 2005 to 2024. 136](#_Toc228977558)

[Table S149: Logistic regression analysis of the effect of continuous time on the antibiotic resistance of *Haemophilus influenzae* from 2005 to 2024. 137](#_Toc228977559)

[Table S150: Annual percentage of *Haemophilus influenzae* resistance to Aminopenicillin from 2005 to 2024. 137](#_Toc228977560)

[Table S151: Annual percentage of *Haemophilus influenzae* resistance to Amoxicillin/Clavulanic Acid from 2005 to 2024. 138](#_Toc228977561)

[Table S152: Annual percentage of *Haemophilus influenzae* resistance to Cefotaxime from 2005 to 2024. 139](#_Toc228977562)

[Table S153: Annual percentage of *Haemophilus influenzae* resistance to Levofloxacin from 2005 to 2024. 139](#_Toc228977563)

[Table S154: Annual percentage of *Haemophilus influenzae* resistance to Tetracycline from 2005 to 2024. 140](#_Toc228977564)

[Table S155: Annual percentage of *Haemophilus influenzae* resistance to Cotrimoxazole from 2005 to 2024. 141](#_Toc228977565)

[Figure S18: Annual percentage of *Neisseria gonorrhoeae* resistance from 2005 to 2024. 142](#_Toc228977566)

[Table S156: Logistic regression analysis of the effect of continuous time on the antibiotic resistance of *Neisseria gonorrhoeae* from 2005 to 2024. 143](#_Toc228977567)

[Table S157: Annual percentage of *Neisseria gonorrhoeae* resistance to Ciprofloxacin from 2005 to 2024. 143](#_Toc228977568)

[Table S158: Annual percentage of *Neisseria gonorrhoeae* resistance to Ceftriaxone from 2005 to 2024. 144](#_Toc228977569)

[Table S159: Annual percentage of *Neisseria gonorrhoeae* resistance to Penicillin from 2005 to 2024. 145](#_Toc228977570)

[Table S160: Annual percentage of *Neisseria gonorrhoeae* resistance to Spectinomycin from 2005 to 2024. 145](#_Toc228977571)

[Table S161: Annual percentage of *Neisseria gonorrhoeae* resistance to Tetracycline from 2005 to 2024. 146](#_Toc228977572)

[Staphylococci 147](#_Toc228977573)

[Figure S19: Annual percentage of *Staphylococcus aureus* resistance from 2005 to 2024. 148](#_Toc228977574)

[Table S162: Logistic regression analysis of the effect of continuous time on the antibiotic resistance of *Staphylococcus aureus* from 2005 to 2024. 148](#_Toc228977575)

[Table S163: Annual percentage of *Staphylococcus aureus* resistance to Clindamycin from 2005 to 2024. 149](#_Toc228977576)

[Table S164: Annual percentage of *Staphylococcus aureus* resistance to Fusidic acid from 2005 to 2024. 149](#_Toc228977577)

[Table S165: Annual percentage of *Staphylococcus aureus* resistance to Gentamicin from 2005 to 2024. 150](#_Toc228977578)

[Table S166: Annual percentage of *Staphylococcus aureus* resistance to Levofloxacin from 2005 to 2024. 151](#_Toc228977579)

[Table S167: Annual percentage of *Staphylococcus aureus* resistance to Linezolid from 2005 to 2024. 152](#_Toc228977580)

[Table S168: Annual percentage of *Staphylococcus aureus* resistance to Oxacillin from 2005 to 2024. 153](#_Toc228977581)

[Table S169: Annual percentage of *Staphylococcus aureus* resistance to Pristinamycin from 2005 to 2024. 154](#_Toc228977582)

[Table S170: Annual percentage of *Staphylococcus aureus* resistance to Rifampicin from 2005 to 2024. 154](#_Toc228977583)

[Table S171: Annual percentage of *Staphylococcus aureus* resistance to Tetracycline from 2005 to 2024. 155](#_Toc228977584)

[Table S172: Annual percentage of *Staphylococcus aureus* resistance to Cotrimoxazole from 2005 to 2024. 156](#_Toc228977585)

[Streptococci 158](#_Toc228977586)

[Figure S20: Annual percentage of *Streptococcus pneumoniae* resistance from 2005 to 2024. 158](#_Toc228977587)

[Table S173: Logistic regression analysis of the effect of continuous time on the antibiotic resistance of *Streptococcus pneumoniae* from 2005 to 2024. 158](#_Toc228977588)

[Table S174: Annual percentage of *Streptococcus pneumoniae* resistance to Aminopenicillin from 2005 to 2024. 159](#_Toc228977589)

[Table S175: Annual percentage of *Streptococcus pneumoniae* resistance to Cefoxtaxime from 2005 to 2024. 159](#_Toc228977590)

[Table S176: Annual percentage of *Streptococcus pneumoniae* resistance to Clindamycin from 2005 to 2024. 160](#_Toc228977591)

[Table S177: Annual percentage of *Streptococcus pneumoniae* resistance to Mixofloxacin from 2005 to 2024. 161](#_Toc228977592)

[Table S178: Annual percentage of *Streptococcus pneumoniae* resistance to Penicillin from 2005 to 2024. 162](#_Toc228977593)

[Table S179: Annual percentage of *Streptococcus pneumoniae* resistance to Pristinamycin from 2005 to 2024. 162](#_Toc228977594)

[Table S180: Annual percentage of *Streptococcus pneumoniae* resistance to Tetracycline from 2005 to 2024. 163](#_Toc228977595)

[Table S181: Annual percentage of *Streptococcus pneumoniae* resistance to Cotrimoxazole from 2005 to 2024. 164](#_Toc228977596)

[Figure S11: Annual percentage of *Streptococcus pyogenes* resistance from 2005 to 2024. 165](#_Toc228977597)

[Table S182: Logistic regression analysis of the effect of continuous time on the antibiotic resistance of *Streptococcus pyogenes* from 2005 to 2024. 165](#_Toc228977598)

[Table S183: Annual percentage of *Streptococcus pyogenes* resistance to Erythromycin from 2005 to 2024. 166](#_Toc228977599)

[Table S184: Annual percentage of *Streptococcus pyogenes* resistance to Levofloxacin from 2005 to 2024. 166](#_Toc228977600)

[Table S185: Annual percentage of *Streptococcus pyogenes* resistance to Cefotaxime from 2005 to 2024. 167](#_Toc228977601)

[Table S186: Annual percentage of *Streptococcus pyogenes* resistance to Gentamycin from 2005 to 2024. 168](#_Toc228977602)

[Table S187: Annual percentage of *Streptococcus pyogenes* resistance to Tetracycline from 2005 to 2024. 169](#_Toc228977603)

[Table S188: Annual percentage of *Streptococcus pyogenes* resistance to Cotrimoxazole from 2005 to 2024. 170](#_Toc228977604)

[Figure S22: Annual percentage of *Streptococcus agalactiae* resistance from 2005 to 2024. 171](#_Toc228977605)

[Table S189: Logistic regression analysis of the effect of continuous time on the antibiotic resistance of *Streptococcus agalactiae* from 2005 to 2024. 171](#_Toc228977606)

[Table S190: Annual percentage of *Streptococcus agalactiae* resistance to Erythromycin from 2005 to 2024. 172](#_Toc228977607)

[Table S191: Annual percentage of *Streptococcus agalactiae* resistance to Cefotaxime from 2005 to 2024. 172](#_Toc228977608)

[Table S192: Annual percentage of *Streptococcus agalactiae* resistance to Gentamicin from 2005 to 2024. 173](#_Toc228977609)

[Table S193: Annual percentage of *Streptococcus agalactiae* resistance to Levofloxacin from 2005 to 2024. 174](#_Toc228977610)

[Table S194: Annual percentage of *Streptococcus agalactiae* resistance to Tetracycline from 2005 to 2024. 175](#_Toc228977611)

[Table S195: Annual percentage of *Streptococcus agalactiae* resistance to Cotrimoxazole from 2005 to 2024. 176](#_Toc228977612)

[Figure S23: Annual percentage of resistance for six pathogen-drug combinations among community-acquired isolates versus among hospital-acquired isolates from 2005 to 2024. 177](#_Toc228977613)

[D) High Priority Pathogens 177](#_Toc228977614)

[Table S196: Distribution of HPP types. 178](#_Toc228977615)

[Table S197: HPP prevalence within each bacterial species or genera. 178](#_Toc228977616)

[Table S198: ESBL prevalence by bacterial species or genera. 178](#_Toc228977617)

[Table S199: Annual percentage of ESBL-E among Enterobacterales from 2005 to 2024. 179](#_Toc228977618)

[Table S200: CPE prevalence by bacterial species or genera. 179](#_Toc228977619)

[Table S201: Annual percentage of CPE among Enterobacterales from 2005 to 2024. 180](#_Toc228977620)

[Figure S24: Annual percentage of resistance for HPP isolates among community-acquired isolates versus among hospital-acquired isolates from 2005 to 2024. 181](#_Toc228977621)

[Table S202: Logistic regression analyses for each of the six HPP types–from 2005 to 2024. 181](#_Toc228977622)

[E) MDR and pXDR 182](#_Toc228977623)

[Table S203: MDR prevalence within each HPP type. 183](#_Toc228977624)

[Table S204: MDR prevalence within each bacterial pathogen. 183](#_Toc228977625)

[Table S205: Hospital-acquired prevalence among MDR isolates per year. 183](#_Toc228977626)

[Figure S25: Annual MDR prevalence with annual proportions of hospital-acquired and community-acquired, from 2005 to 2024. 184](#_Toc228977627)

[Table S206: Annual MDR percentage among 110,205 isolates. 185](#_Toc228977628)

[Table S207: Mixed-Effect logistic regression analysis of the effect continuous time–blood infection and hospital-acquired infection on the presence of MDR isolates across all isolates–from 2005 to 2024. 185](#_Toc228977629)

[Table S208: Mixed-Effect logistic regression analysis of the effect service on the presence of MDR isolates across CHT isolates–from 2005 to 2024. 186](#_Toc228977630)

[Table S209: Annual pXDR percentage among 33,75 isolates. 186](#_Toc228977631)

[Table S210: pXDR prevalence within each bacterial pathogen. 187](#_Toc228977632)

# A) Description

### Table S1: List of the 157 pathogen-drug combinations.

| Bacteria | Antibiotic |
| --- | --- |
| *Acinetobacter baumannii* | Ceftazidime |
|  | Ciprofloxacin |
|  | Gentamycin |
|  | Imipenem |
|  | Cotrimoxazole |
|  | Piperacillin/Tazobactam |
| *Citrobacter freundii* | Amikacin |
|  | Ceftazidime |
|  | Ciprofloxacin |
|  | Cefotaxime |
|  | Gentamycin |
|  | Imipenem |
|  | Cotrimoxazole |
|  | Piperacillin/Tazobactam |
| *Citrobacter koseri* | Amikacin |
|  | Amoxicillin/Clavulanic Acid |
|  | Ceftazidime |
|  | Ciprofloxacin |
|  | Cefotaxime |
|  | Gentamicin |
|  | Imipenem |
|  | Cotrimoxazole |
|  | Piperacillin/Tazobactam |
| *Escherichia coli* | Amikacin |
|  | Amoxicillin/Clavulanic Acid |
|  | Aminopenicillin |
|  | Ceftazidime |
|  | Ciprofloxacin |
|  | Cefotaxime |
|  | Gentamicin |
|  | Imipenem |
|  | Cotrimoxazole |
|  | Piperacillin/Tazobactam |
| *Enterobacter cloacae complex* | Amikacin |
|  | Ceftazidime |
|  | Ciprofloxacin |
|  | Cefotaxime |
|  | Gentamicin |
|  | Imipenem |
|  | Cotrimoxazole |
|  | Piperacillin/Tazobactam |
| *Enterococcus faecalis* | Erythromycin |
|  | Gentamicin |
|  | Linezolid |
|  | Teicoplanin |
|  | Vancomycin |
| *Enterococcus faecium* | Aminopenicillin |
|  | Erythromycin |
|  | Gentamicin |
|  | Linezolid |
|  | Teicoplanin |
|  | Vancomycin |
| *Haemophilus influenzae* | Aminopenicillin |
|  | Amoxicillin/Clavulanic Acid |
|  | Cefotaxime |
|  | Levofloxacin |
|  | Tetracycline |
|  | Cotrimoxazole |
| *Klebsiella pneumoniae* | Amoxicillin/Clavulanic Acid |
|  | Amikacin |
|  | Ceftazidime |
|  | Ciprofloxacin |
|  | Cefotaxime |
|  | Gentamicin |
|  | Imipenem |
|  | Cotrimoxazole |
|  | Piperacillin/Tazobactam |
| *Morganella morganii* | Amikacin |
|  | Ceftazidime |
|  | Ciprofloxacin |
|  | Cefotaxime |
|  | Gentamicin |
|  | Imipenem |
|  | Cotrimoxazole |
|  | Piperacillin/Tazobactam |
| *Mycobacterium tuberculosis* | Ethambutol |
|  | Isoniazid |
|  | Rifampin |
|  | Streptomycin |
| *Neisseria gonorrhoeae* | Ciprofloxacin |
|  | Cefotaxime |
|  | Penicillin |
|  | Spectinomycin |
|  | Tetracycline |
| *Proteus mirabilis* | Amikacin |
|  | Aminopenicillin |
|  | Amoxicillin/Clavulanic Acid |
|  | Ceftazidime |
|  | Ciprofloxacin |
|  | Cefotaxime |
|  | Gentamicin |
|  | Cotrimoxazole |
|  | Piperacillin/Tazobactam |
| *Pseudomonas aeruginosa* | Amikacin |
|  | Ceftazidime |
|  | Ciprofloxacin |
|  | Gentamicin |
|  | Imipenem |
|  | Piperacillin/Tazobactam |
| *Serratia marcescens* | Ceftazidime |
|  | Ciprofloxacin |
|  | Cefotaxime |
|  | Gentamicin |
|  | Imipenem |
|  | Cotrimoxazole |
|  | Piperacillin/Tazobactam |
| *Salmonella spp.* | Aminopenicillin |
|  | Amoxicillin/Clavulanic Acid |
|  | Ceftazidime |
|  | Ciprofloxacin |
|  | Cefotaxime |
|  | Imipenem |
|  | Cotrimoxazole |
| *Shigella spp.* | \| Amikacin \| \| --- \| \| Aminopenicillin \| \| Amoxicillin/Clavulanic Acid \| \| Ceftazidime \| \| Ciprofloxacin \| \| Cefotaxime \| \| Gentamicin \| \| Imipenem \| \| Cotrimoxazole \| \| Piperacillin/Tazobactam \| |
| *Staphylococcus aureus* | Clindamycin |
|  | Fusidic acid |
|  | Gentamicin |
|  | Levofloxacin |
|  | Linezolid |
|  | Oxacillin |
|  | Pristinamycin |
|  | Rifampicin |
|  | Tetracycline |
|  | Cotrimoxazole |
| *Stenotrophomonas maltophilia* | Ceftazidime |
|  | Levofloxacin |
|  | Ticarcillin/Clavulanic Acid |
|  | Cotrimoxazole |
| *Streptococcus pneumoniae* | Aminopenicillin |
|  | Cefotaxime |
|  | Clindamycin |
|  | Moxifloxacin |
|  | Penicillin |
|  | Pristinamycin |
|  | Tetracycline |
|  | Cotrimoxazole |
| *Streptococcus pyogenes* | Erythromycin |
|  | Cefotaxime |
|  | Levofloxacin |
|  | Gentamicin |
|  | Tetracycline |
|  | Cotrimoxazole |
| *Streptococcus agalactiae* | Erythromycin |
|  | Cefotaxime |
|  | Gentamicin |
|  | Levofloxacin |
|  | Cotrimoxazole |

*Enterobacter cloacae* complex = *E. cloacae, E. cloacae sub cloacae, E. kobei, E. asburiae*

*Salmonella* spp. = S. Agona, S. Arechavaleta, S. Ayton, S. Assinie, S. Branderup, S. Enteritidis, S. Gloucester, S. Heidelberg, S. Ibadan, S. Infantis, S. Gallinarum, S. Minnesota, S. Mississippi, S. Papuana, S. Paratyphi C, S. Senftenberg, S. Stanley, S. Typhi, S. Typhimurium, S. Thompson, S. Weltevreden

*Shigella* spp. = *S. boydii, S. dysenteriae, S. flexneri, S. sonnei*

### Table S2: Susceptibility and resistance imputation rules.

| Reference molecule | Molecular compounds | Species or Genera | Time period |
| --- | --- | --- | --- |
| Aminopenicillin | Amoxicillin,  Ampicillin | All | 2005-2024 |
| Levofloxacin | Levofloxacin,  Nalidixic acid | *Haemophillus influenza* | 2005-2024 |
| Levofloxacin | Levofloxacin,  Ofloxacin | *Staphylococcus aureus* | 2005-2024 |
| Ciprofloxacin | Ciprofloxacin,  Nalidixic acid | *Neisseria gonorrhoeae* | 2005-2024 |
| Clindamycin | Clindamycin,  Lincomycin | *Staphylococcus aureus*  *Streptococcus pneumoniae* | 2005-2024 |
| Cefotaxime | Cefotaxime,  Ceftriaxone | *Citrobacter freundii,*  *Citrobacter koseri, Escherichia coli,*  *Enterobacter cloacae* complex*,*  *Haemophilus influenzae,*  *Klebsiella pneumoniae,*  *Morganella morganii,*  *Neisseria gonorrheae,*  *Proteus mirabilis, Serratia marcescens,*  *Salmonella* spp.*,*  *Streptococcus pneumoniae* | 2005-2024 |
| Penicillin | Penicillin,  Penicillin G | *Neisseria gonorrheae*  *Streptococcus pneumoniae* | 2005-2024 |
| Ceftriaxone | Ceftriaxone,  Cefotaxime,  Penicillin G (S) | *Neisseria gonorrhoeae* | 2010-2014 |

### Table S3: Imputation rules for High-Priority Pathogen (HPP) in New Caledonia.

| HHP | Species | Antibiotic |
| --- | --- | --- |
| Extended-spectrum β-lactamase (ESBL) | *Enterobacterales* | Cefotaxime |
| Methicillin-resistant *Staphylococcus aureus* (MRSA) | *Staphylococcus aureus* | Oxacillin |
| *Acinetobacter baumannii* resistant to imipenem (CR-AB) | *Acinetobacter baumannii* | Imipenem |
| *Pseudomonas aeruginosa* resistant to ceftazidime (CAZR-PA) | *Pseudomonas aeruginosa* | Ceftazidime |
| Vancomycin-resistant enterococci (VRE) | *Enterococcus faecium* | Vancomycin |

### Table S4: Antibiotic classes used for multidrug (MDR) and possible extensively-drug resistance (pXDR).

| Classes | Molecules |
| --- | --- |
| Aminoglycosides | Amikacin |
| Aminoglycosides | Gentamycin |
| Aminoglycosides | Netilmicin |
| Aminoglycosides | Spectinomycin |
| Anti-MRSA cephalosporins | Ceftaroline |
| Anti-pseudomonal 3GC | Ceftazidime |
| Anti-pseudomonal penicillin inhibitors | Ticarcillin/Clavulanic Acid |
| Anti-pseudomonal penicillin inhibiters | Piperacillin/Tazobactam |
| Anti-staphylococcal | Cefoxitin |
| Anti-staphylococcal | Oxacillin |
| Anti-tuberculosis | Ethambutol |
| Anti-tuberculosis | Isoniazid |
| Asamycins | Rifampin |
| 1G Cephalosporins | Cefaclor |
| 1GC | Cefazoline |
| 2G Cephalosporins | Cefotetan |
| 2GC | Cefuroxime |
| 3G Cephalosporins | Ceftazidime/Avibactam |
| 3GC | Ceftolozane/Tazobactam |
| 3GC | Cefixime |
| 3GC | Cefotaxime |
| 3GC | Cefpodoxime |
| 3GC | Cefpirome |
| Carbapenems | Ertapenem |
| Carbapenems | Imipenem |
| Carbapenems | Isepamicin |
| Fluoroquinolones | Ciprofloxacin |
| Fluoroquinolones | Gatifloxacin |
| Fluoroquinolones | Levofloxacin |
| Fluoroquinolones | Latamoxef |
| Fluoroquinolones | Moxifloxacin |
| Fluoroquinolones | Norfloxacin |
| Fluoroquinolones | Pefloxacine/2GQuinolone |
| Fluoroquinolones | Sparfoxacin |
| Folate inhibitors | Trimethoprime |
| Fucidanes | Fusidic acid |
| Glycopeptides | Teicoplanin |
| Glycopeptides | Vancomycin |
| Glycylcyclines | Tigecycline |
| Lincosamides | Clindamycin |
| Lincosamides | Clindamycin hydrochloride topical |
| Lincosamides | Lincomycin |
| Lipopeptides | Daptomycin |
| Macrolides | Clarithromycin |
| Macrolides | Erythromycin |
| Macrolides | Spiramycin |
| Macrolides | Telithromycin |
| Monobactam | Aztreonam |
| Oxazolidinones | Linezolid |
| Penicillin inhibitors | Amoxicillin/Clavulanic Acid |
| Penicillin inhibiters | Ampicillin/Sulbactam |
| Penicillins | Aminopenicillin |
| Penicillins | Pivmecillinam |
| Penicillins | Penicillin |
| Penicillins | Piperacillin |
| Penicillins | Pipemidique acid |
| Penicillins | Temocillin |
| Phenicols | Chloramphenicol |
| Phosphonic acids | Fosfomycin |
| Polymyxins | Colistin |
| Streptomycins | Streptomycin |
| Sulfamides | Cotrimoxazole |
| Synergistins | Pristinamycin |
| Tetracyclines | Minocycline |
| Tetracyclines | Tetracycline |
| Topical | Mupirocin |

### Table S5: Major classes used to identify XDR.

| Pathogen | Classes to be checked | Tested classes under check | Maximum susceptible classes | Maximum missing classes |
| --- | --- | --- | --- | --- |
| *Acinetobacter baumannii* | Aminoglycosides  β-lactam/BLIs Carbapenems Cephalosporins Fluoroquinolones  Folate inhibitors  Penicillins Polymyxins Tetracyclines | 6 | 2 | 0 if 4/6 tested 1 if 5/6 tested 2 if 6/6 tested |
| *Enterobacterales* | Aminoglycosides Carbapenems Cephalosporins Fluoroquinolones  Glycylcyclines  Monobactams  Phenicols  Phosphonic acids  Polymyxins^1^ Tetracyclines^2^ | 6 | 2 | 0 if 4/6 tested 1 if 5/6 tested 2 if 6/6 tested |
| *Enterococci* | Glycopeptides Aminoglycosides  Carbapenems^3^  Fluoroquinolones Oxazolidinones Lipopeptides Penicillins  Streptogramins^4^ |  |  | 0 if 5/7 tested 1 if 6/7 tested |
|  | Tetracycline | 7 | 2 | 2 if 7/7 tested |
| *Haemophilus influenzae* | Aminopenicillins  Cephalosporins  Carbapenems Fluoroquinolones Folate inhibitors  Tetracyclines | 5 | 2 | 0 if 3/5 tested 1 if 4/5 tested 2 if 5/5 tested |
| *Mycobacterium tuberculosis* | Asamycins Isoniazid Fluoroquinolones Oxazolidinones | 4 | 0 | 0 if 4/4 tested |
| *Neisseria gonorrhoeae* | Aminoglycosides  Cephalosporins Macrolides Fluoroquinolones Penicillins Tetracyclines | 6 | 2 | 0 if 4/6 tested 1 if 5/6 tested 2 if 6/6 tested |
| *Pseudomonas aeruginosa* | Aminoglycosides Carbapenems Cephalosporins Fluoroquinolones  β-lactam/BLIs Monobactams  Phosphonic acids Polymyxins | 6 | 2 | 0 if 4/6 tested 1 if 5/6 tested 2 if 6/6 tested |
| *Staphylococcus aureus* | Aminoglycosides  Ansamycins  Cephalosporins  Penicillins (oxacillin) Fluoroquinolones  Folate inhibitors  Fucidanes  Glycopeptides  Glycylcyclines  Lincosamides  Lipopeptides  Oxazolidinones  Phenicols  Phosphonic acids  Streptogramins Tetracyclines | 12 | 2 | 0 if 10/12 tested 1 if 11/12 tested 2 if 12/12 tested |
| *Stenotrophomonas maltophilia* | Cephalosporins  Fluoroquinolones  Tetracyclines Folate inhibitors β-lactams/BLIs | 5 | 2 | 0 if 3/5 tested 1 if 4/5 tested 2 if 5/5 tested |
| *Streptococci* | Aminoglycosides  Penicillins Cephalosporins Macrolides  Lincosamides  Fluoroquinolones Tetracyclines | 7 | 2 | 0 if 5/7 tested 1 if 6/7 tested 2 if 7/7 tested |

β-lactam/BLIs = Anti-pseudomonal penicillin inhibitors, Penicillin inhibitors

^1^ Polymyxins not used for *M. morganii*, *P. mirabilis*, *S. marcescens*

^2^ Tetracyclines not for *M. morganii*, and *P. mirabilis*

^3^ Carbapenems not used for *E. faecium*

^4^ Streptogramins not used for *E. faecalis*

### Table S6: Intrinsic resistance.

| Species | Antibiotic |
| --- | --- |
| Acinetobacter baumannii | Aminopenicillin  Amoxicillin/Clavulanic Acid  Cefotaxime  Cefoxitin  Clindamycin  Erythromycin  Pristinamycin  Spectinomycin  Linezolid  Vancomycin  Teicoplanin  Fusidic Acid  Rifampicin  Oxacillin  Penicillin |
| Citrobacter freundii | Aminopenicillin  Amoxicillin/Clavulanic Acid  Cefoxitin  Clindamycin  Erythromycin  Pristinamycin  Spectinomycin  Linezolid  Vancomycin  Teicoplanin  Fusidic Acid  Rifampicin  Oxacillin  Penicillin |
| Citrobacter koseri | Aminopenicillin  Ticarcillin  Vancomycin  Teicoplanin  Fusidic Acid  Rifampicin  Oxacillin  Penicillin |
| Escherichia coli | Aminopenicillin  Clindamycin  Erythromycin  Pristinamycin  Spectinomycin  Linezolid  Vancomycin  Teicoplanin  Fusidic Acid  Rifampicin  Oxacillin  Penicillin |
| Enterobacter cloacae complex | Amoxicillin/Clavulanic Acid  Cefoxitin  Clindamycin  Erythromycin  Pristinamycin  Spectinomycin  Linezolid  Vancomycin  Teicoplanin  Fusidic Acid  Rifampicin  Oxacillin  Penicillin |
| Enterococcus faecium | Cefotaxime  Cefoxitin  Ceftazidime  Clindamycin  Cotrimoxazole  Oxacillin |
| Enterococcus faecalis | Cefotaxime  Cefoxitin  Ceftazidime  Cotrimoxazole  Oxacillin |
| Haemophilus influenzae | Vancomycin  Teicoplanin  Fusidic Acid  Rifampicin  Oxacillin  Penicillin |
| Klebsiella pneumoniae | Aminopenicillin  Ticarcillin  Vancomycin  Teicoplanin  Fusidic Acid  Rifampicin  Oxacillin  Penicillin |
| Morganella morganii | Aminopenicillin  Amoxicillin/Clavulanic Acid  Ticarcillin  Cefoxitin  Clindamycin  Erythromycin  Pristinamycin  Spectinomycin  Linezolid  Vancomycin  Teicoplanin  Fusidic Acid  Rifampicin  Oxacillin  Penicillin |
| Neisseria gonorrhoeae | Oxacillin |
| Pseudomonas aeruginosa | Aminopenicillin  Amoxicillin/Clavulanic Acid  Cefotaxime  Cefoxitin  Clindamycin  Erythromycin  Pristinamycin  Spectinomycin  Linezolid  Vancomycin  Teicoplanin  Fusidic Acid  Rifampicin  Cotrimoxazole  Oxacillin  Penicillin |
| Salmonella *spp.* | Amikacin  Gentamicin  Vancomycin  Teicoplanin  Fusidic Acid  Rifampicin  Oxacillin  Penicillin |
| Serratia marcescens | Amikacin  Aminopenicillin  Amoxicillin/Clavulanic Acid  Cefoxitin  Clindamycin  Erythromycin  Pristinamycin  Spectinomycin  Linezolid  Vancomycin  Teicoplanin  Fusidic Acid  Rifampicin  Oxacillin  Penicillin |
| Staphylococcus aureus | Penicillin |
| Stenotrophomonas maltophilia | Aminopenicillin  Amoxicillin/Clavulanic Acid  Ticarcillin  Cefotaxime  Cefoxitin  Clindamycin  Erythromycin  Pristinamycin  Spectinomycin  Linezolid  Vancomycin  Teicoplanin  Fusidic Acid  Rifampicin  Amikacin  Gentamicin  Imipenem  Piperacillin/Tazobactam  Tetracycline  Fosfomycin  Oxacillin  Penicillin |

### Table S7: Data management by species or genera.

|  | **Extracted** | Screening | | **Diagnostic** | Non tested | | **Tested** | Duplicates | | **Included** |
| --- | --- | --- | --- | --- | --- | --- | --- | --- | --- | --- |
|  | N | N (%) | | N | N | (%) |  | N | (%) | N |
| *Acinetobacter baumannii* | 2,467 | 203 | (8.2) | 2,264 | 256 | (11.3) | 2,008 | 667 | (33.2) | 1,341 |
| *Citrobacter freundii* | 1,381 | 205 | (14.8) | 1,176 | 179 | (15.2) | 997 | 251 | (25.2) | 746 |
| *Citrobacter koseri* | 3,475 | 58 | (1.7) | 3,417 | 322 | (9.4) | 3,095 | 866 | (28.0) | 2,229 |
| *Escherichia coli* | 49,446 | 2,780 | (5.6) | 46,666 | 2,214 | (4.7) | 44,452 | 8,512 | (19.1) | 35,940 |
| *Enterobacter cloacae complex* | 8,810 | 1,392 | (15.8) | 7,418 | 1,066 | (14.4) | 6,352 | 1,923 | (30.3) | 4,429 |
| *Enterococcus faecium* | 1,158 | 510 | (44.0) | 648 | 102 | (15.7) | 546 | 158 | (28.9) | 388 |
| *Enterococcus faecalis* | 10,517 | 14 | (0.1) | 10,503 | 4,311 | (41.0) | 6,192 | 1,528 | (24.7) | 4,664 |
| *Haemophilus influenzae* | 7,728 | 33 | (0.4) | 7,695 | 493 | (6.4) | 7,202 | 1,495 | (20.8) | 5,707 |
| *Klebsiella pneumoniae* | 14,357 | 1,407 | (9.8) | 12,950 | 1,316 | (10.2) | 11,634 | 3,072 | (26.4) | 8,562 |
| *Stenotrophomonas maltophilia* | 1,259 | 29 | (2.3) | 1,230 | 263 | (21.4) | 967 | 351 | (36.3) | 616 |
| *Morganella morganii* | 2,322 | 33 | (1.4) | 2,289 | 1,091 | (47.7) | 1,198 | 230 | (19.2) | 968 |
| *Mycobacterium tuberculosis* | 723 | 0 | (0.0) | 723 | 15 | (2.1) | 708 | 61 | (8.6) | 647 |
| *Neisseria gonorrhoeae* | 3,120 | 0 | (0.0) | 3,120 | 94 | (3.0) | 3,026 | 176 | (5.8) | 2,850 |
| *Proteus mirabilis* | 7,216 | 38 | (0.5) | 7,178 | 764 | (10.6) | 6,414 | 1,592 | (24.8) | 4,822 |
| *Streptococcus pneumoniae* | 4,813 | 276 | (5.7) | 4,537 | 453 | (10.0) | 4,084 | 975 | (23.9) | 3,109 |
| *Pseudomonas aeruginosa* | 12,321 | 357 | (2.9) | 11,964 | 1,529 | (12.8) | 10,435 | 4,262 | (40.8) | 6,173 |
| *Staphylococcus aureus* | 42,390 | 1,323 | (3.1) | 41,067 | 5,486 | (13.4) | 35,581 | 11,175 | (31.4) | 24,406 |
| *Salmonella spp.* | 1,228 | 1 | (0.1) | 1,227 | 254 | (20.7) | 973 | 116 | (11.9) | 857 |
| *Shigella spp.* | 197 | 0 | (0.0) | 197 | 35 | (17.8) | 162 | 8 | (4.9) | 154 |
| *Serratia marcescens* | 2,225 | 99 | (4.4) | 2,126 | 329 | (15.5) | 1,797 | 550 | (30.6) | 1,247 |
| *Streptococcus agalactiae* | 13,543 | 3 | (0.0) | 13,540 | 13,087 | (96.7) | 453 | 132 | (29.1) | 321 |
| *Streptococcus pyogenes* | 8,496 | 11 | (0.1) | 8,485 | 7,333 | (86.4) | 1,152 | 306 | (26.6) | 846 |
| Total | **199,192** | 8772 | (4.4) | **190,420** | 40992 | (21.5) | **149,428** | 38,406 | (25.7) | **111,022** |

### Table S8: Number of patients, samples, and isolates per year.

| Year | Patients | Samples | Isolates |
| --- | --- | --- | --- |
| 2005 | 3,707 | 4,637 | 4,985 |
| 2006 | 3,750 | 4,654 | 5,011 |
| 2007 | 4,251 | 5,262 | 5,703 |
| 2008 | 4,468 | 5,434 | 5,961 |
| 2009 | 4,639 | 5,686 | 6,219 |
| 2010 | 4,479 | 5,678 | 6,188 |
| 2011 | 4,437 | 5,659 | 6,109 |
| 2012 | 4,166 | 5,235 | 5,679 |
| 2013 | 4,018 | 4,940 | 5,287 |
| 2014 | 4,209 | 5,230 | 5,675 |
| 2015 | 4,169 | 4,981 | 5,387 |
| 2016 | 4,153 | 5,048 | 5,537 |
| 2017 | 3,997 | 4,723 | 5,061 |
| 2018 | 4,272 | 5,099 | 5,450 |
| 2019 | 4,292 | 5,134 | 5,501 |
| 2020 | 4,182 | 4,964 | 5,399 |
| 2021 | 4,207 | 4,999 | 5,451 |
| 2022 | 4,663 | 5,536 | 6,071 |
| 2023 | 4,361 | 5,086 | 5,534 |
| 2024 | 3,832 | 4,434 | 4,814 |
| Total |  | 102,419 | 111,022 |

### Table S9: Prevalence of each bacterial species or genera among the total number of isolates.

| Group | Pathogen | Frequency | Percentage (%) | [95%CI] |
| --- | --- | --- | --- | --- |
| Enterobacterales | *Citrobacter freundii* | 746 | 0.7 | [0.6–0.7] |
|  | *Citrobacter koseri* | 2,229 | 2.0 | [1.9–2.1] |
|  | *Escherichia coli* | 35,94 | 32.4 | [32.1–32.6] |
|  | *Enterobacter cloacae complex* | 4,429 | 4.0 | [3.9–4.1] |
|  | *Klebsiella pneumoniae* | 8,562 | 7.7 | [7.6–7.9] |
|  | *Morganella morganii* | 968 | 0.9 | [0.8–0.9] |
|  | *Proteus mirabilis* | 4,822 | 4.3 | [4.2–4.5] |
|  | *Salmonella spp.* | 857 | 0.8 | [0.7–0.8] |
|  | *Shigella spp.* | 154 | 0.1 | [0.1–0.2] |
|  | *Serratia marcescens* | 1,247 | 1.1 | [1.1–1.2] |
| Enterococci | *Enterococcus faecium* | 388 | 0.3 | [0.3–0.4] |
|  | *Enterococcus faecalis* | 4,664 | 4.2 | [4.1–4.3] |
| Mycobacteria | *Mycobacterium tuberculosis* | 647 | 0.6 | [0.5–0.6] |
| Non-fermenters | *Acinetobacter baumannii* | 1,341 | 1.2 | [1.1–1.3] |
|  | *Stenotrophomonas maltophilia* | 616 | 0.6 | [0.5–0.6] |
|  | *Pseudomonas aeruginosa* | 6,173 | 5.6 | [5.4–5.7] |
| Other Gram-negatives | *Haemophilus influenzae* | 5,707 | 5.1 | [5–5.3] |
|  | *Neisseria gonorrhoeae* | 2,85 | 2.6 | [2.5–2.7] |
| Staphylococci | *Staphylococcus aureus* | 24,406 | 22 | [21.7–22.2] |
| Streptococci | *Streptococcus pneumoniae* | 3,109 | 2.8 | [2.7–2.9] |
|  | *Streptococcus agalactiae* | 321 | 0.3 | [0.3–0.3] |
|  | *Streptococcus pyogenes* | 846 | 0.8 | [0.7–0.8] |

# B) Enterobacterales and key antimicrobial agents

### Table S10: Logistic regression analysis of the effect of continuous time on antibiotic resistance of Enterobacterales from 2005 to 2024

| Antibiotic | β | OR | [95%CI] | P-value |  |
| --- | --- | --- | --- | --- | --- |
| Amikacin | 0.077 | 1.08 | [1.07–1.09] | 0.000 | *** |
| Fluoroquinolones | -0.009 | 0.99 | [0.99–1] | 0.001 | ** |
| 3G Cephalosporins | 0.024 | 1.02 | [1.02–1.03] | 0.000 | *** |
| Imipenem | 0.109 | 1.11 | [1.06–1.17] | 0.000 | *** |
| Cotrimoxazole | 0.002 | 1.00 | [1–1.01] | 0.350 |  |
| Piperacillin/Tazobactam | 0.057 | 1.06 | [1.05–1.06] | 0.000 | *** |

### Table S11: Annual percentage of Enterobacterales resistance to Amikacine from 2005 to 2024.

|  | Tested | Resistance | | Logistic model | |  | |
| --- | --- | --- | --- | --- | --- | --- | --- |
| Year | N | % | [95% CI] | OR | [95% CI] | P-value |  |
| 2005 | 2068 | 1.4 | [0.9–1.9] | – | – | – |  |
| 2006 | 1694 | 0.8 | [0.5–1.4] | 0.61 | [0.32–1.17] | 0.238 |  |
| 2007 | 2844 | 0.8 | [0.5–1.2] | 0.92 | [0.47–1.81] | 0.816 |  |
| 2008 | 2935 | 1.3 | [0.9–1.7] | 1.64 | [0.97–2.79] | 0.221 |  |
| 2009 | 3179 | 1.1 | [0.8–1.5] | 0.87 | [0.55–1.39] | 0.666 |  |
| 2010 | 3182 | 1.5 | [1.1–2] | 1.34 | [0.86–2.08] | 0.308 |  |
| 2011 | 3305 | 1.6 | [1.3–2.1] | 1.07 | [0.72–1.58] | 0.816 |  |
| 2012 | 3106 | 2.2 | [1.7–2.7] | 1.36 | [0.94–1.95] | 0.221 |  |
| 2013 | 2939 | 1.4 | [1–1.9] | 0.64 | [0.43–0.95] | 0.121 |  |
| 2014 | 3173 | 0.9 | [0.7–1.3] | 0.68 | [0.42–1.09] | 0.221 |  |
| 2015 | 2946 | 0.7 | [0.4–1] | 0.71 | [0.4–1.25] | 0.313 |  |
| 2016 | 3069 | 0.4 | [0.2–0.7] | 0.56 | [0.27–1.15] | 0.221 |  |
| 2017 | 2803 | 1.5 | [1.1–2] | 3.73 | [1.96–7.12] | 0.001 | *** |
| 2018 | 3027 | 2.7 | [2.2–3.3] | 1.89 | [1.29–2.76] | 0.007 | ** |
| 2019 | 3068 | 2.2 | [1.8–2.8] | 0.82 | [0.59–1.13] | 0.313 |  |
| 2020 | 3029 | 2.9 | [2.4–3.6] | 1.32 | [0.96–1.82] | 0.221 |  |
| 2021 | 3064 | 2.2 | [1.8–2.8] | 0.75 | [0.55–1.04] | 0.221 |  |
| 2022 | 3378 | 2.3 | [1.9–2.9] | 1.04 | [0.75–1.45] | 0.816 |  |
| 2023 | 3146 | 4.9 | [4.2–5.7] | 2.18 | [1.66–2.88] | 0.000 | *** |
| 2024 | 2702 | 4.4 | [3.7–5.2] | 0.87 | [0.68–1.12] | 0.358 |  |

### Table S12: Annual percentage of Enterobacterales resistance to Fluoroquinolones from 2005 to 2024.

|  | Tested | Resistance | | Logistic model | |  | |
| --- | --- | --- | --- | --- | --- | --- | --- |
| Year | N | % | [95% CI] | OR | [95% CI] | P-value |  |
| 2005 | 1858 | 8.2 | [7–9.5] | – | – | – |  |
| 2006 | 2471 | 6.8 | [5.9–7.9] | 0.84 | [0.67–1.06] | 0.279 |  |
| 2007 | 2844 | 6.8 | [6–7.8] | 0.96 | [0.78–1.19] | 0.820 |  |
| 2008 | 2927 | 5.9 | [5.1–6.8] | 0.84 | [0.68–1.04] | 0.279 |  |
| 2009 | 3180 | 6.6 | [5.8–7.5] | 1.18 | [0.96–1.46] | 0.279 |  |
| 2010 | 3182 | 5.6 | [4.8–6.4] | 0.81 | [0.66–1] | 0.279 |  |
| 2011 | 3305 | 8.5 | [7.6–9.5] | 1.5 | [1.23–1.82] | 0.001 | *** |
| 2012 | 3106 | 10.5 | [9.4–11.6] | 1.29 | [1.09–1.53] | 0.032 | * |
| 2013 | 2939 | 9.4 | [8.4–10.5] | 0.89 | [0.75–1.05] | 0.279 |  |
| 2014 | 3174 | 8.9 | [8–10] | 0.93 | [0.78–1.11] | 0.507 |  |
| 2015 | 2948 | 8.7 | [7.7–9.8] | 0.92 | [0.77–1.1] | 0.498 |  |
| 2016 | 3070 | 7.5 | [6.6–8.5] | 0.84 | [0.7–1.01] | 0.279 |  |
| 2017 | 2803 | 7.0 | [6.1–8] | 0.9 | [0.73–1.09] | 0.436 |  |
| 2018 | 2316 | 6.8 | [5.8–7.9] | 0.95 | [0.77–1.19] | 0.791 |  |
| 2019 | 2117 | 7.2 | [6.2–8.4] | 0.98 | [0.78–1.23] | 0.909 |  |
| 2020 | 2807 | 8.4 | [7.5–9.5] | 1.2 | [0.97–1.48] | 0.279 |  |
| 2021 | 2796 | 8.6 | [7.6–9.7] | 0.99 | [0.82–1.2] | 0.937 |  |
| 2022 | 3174 | 7.9 | [7–8.9] | 0.91 | [0.76–1.1] | 0.489 |  |
| 2023 | 2923 | 9.3 | [8.3–10.4] | 1.15 | [0.96–1.38] | 0.279 |  |
| 2024 | 2602 | 8.2 | [7.2–9.3] | 0.87 | [0.72–1.05] | 0.279 |  |

### Table S13: Annual percentage of Enterobacterales resistance to Third-generation cephalosporins from 2005 to 2024.

|  | Tested | Resistance | | Logistic model | |  | |
| --- | --- | --- | --- | --- | --- | --- | --- |
| Year | N | % | [95% CI] | OR | [95% CI] | P-value |  |
| 2005 | 2472 | 5.7 | [4.8–6.6] | – | – | – |  |
| 2006 | 2481 | 4.3 | [3.6–5.2] | 0.77 | [0.59–1] | 0.394 |  |
| 2007 | 2844 | 4.3 | [3.6–5.1] | 0.96 | [0.73–1.25] | 0.899 |  |
| 2008 | 2936 | 4.2 | [3.6–5] | 0.99 | [0.76–1.27] | 0.915 |  |
| 2009 | 3181 | 3.5 | [2.9–4.2] | 0.85 | [0.65–1.1] | 0.597 |  |
| 2010 | 3182 | 4.5 | [3.8–5.3] | 1.25 | [0.97–1.61] | 0.394 |  |
| 2011 | 3306 | 7.7 | [6.8–8.6] | 1.7 | [1.38–2.11] | 0.000 | *** |
| 2012 | 3106 | 7.7 | [6.8–8.7] | 1.02 | [0.85–1.23] | 0.899 |  |
| 2013 | 2940 | 7.9 | [7–9] | 1.04 | [0.86–1.26] | 0.899 |  |
| 2014 | 3174 | 8.4 | [7.5–9.4] | 1.06 | [0.89–1.28] | 0.802 |  |
| 2015 | 2949 | 7.7 | [6.8–8.8] | 0.88 | [0.73–1.06] | 0.525 |  |
| 2016 | 3070 | 8.5 | [7.6–9.6] | 1.1 | [0.92–1.33] | 0.635 |  |
| 2017 | 2804 | 7.6 | [6.7–8.7] | 0.87 | [0.72–1.05] | 0.525 |  |
| 2018 | 3026 | 7.2 | [6.3–8.2] | 0.92 | [0.75–1.11] | 0.722 |  |
| 2019 | 3068 | 8.2 | [7.3–9.2] | 1.11 | [0.92–1.34] | 0.635 |  |
| 2020 | 3034 | 7.8 | [6.9–8.9] | 0.96 | [0.79–1.15] | 0.899 |  |
| 2021 | 3068 | 7.5 | [6.6–8.4] | 0.93 | [0.77–1.13] | 0.802 |  |
| 2022 | 3382 | 7.7 | [6.8–8.6] | 1.02 | [0.85–1.23] | 0.899 |  |
| 2023 | 3146 | 9.0 | [8.1–10.1] | 1.18 | [0.99–1.41] | 0.394 |  |
| 2024 | 2752 | 8.9 | [7.9–10] | 0.98 | [0.81–1.17] | 0.899 |  |

### Table S14: Annual percentage of Enterobacterales resistance to Imipenem from 2005 to 2024.

|  | Tested | Resistance | | Logistic model | |  | |
| --- | --- | --- | --- | --- | --- | --- | --- |
| Year | N | % | [95% CI] | OR | [95% CI] | P-value |  |
| 2005 | 1239 | 0.1 | [0–0.5] | – | – | – |  |
| 2006 | 1140 | 0.2 | [0–0.6] | 2.24 | [0.25–19.68] | 1.000 |  |
| 2007 | 1398 | 0.1 | [0–0.4] | 0.39 | [0.04–3.44] | 1.000 |  |
| 2008 | 1365 | 0.1 | [0–0.4] | 0.99 | [0.08–12.19] | 1.000 |  |
| 2009 | 1568 | 0.1 | [0–0.4] | 0.89 | [0.07–10.88] | 1.000 |  |
| 2010 | 1537 | 0.1 | [0–0.4] | 0.97 | [0.08–11.97] | 1.000 |  |
| 2011 | 2873 | 0.1 | [0–0.3] | 1.13 | [0.13–9.89] | 1.000 |  |
| 2012 | 2931 | 0.0 | [0–0.2] | 0.5 | [0.06–4.42] | 1.000 |  |
| 2013 | 2732 | 0.1 | [0–0.3] | 2.15 | [0.24–18.84] | 1.000 |  |
| 2014 | 2933 | 0.0 | [0–0.1] | – | – | – |  |
| 2015 | 2349 | 0.0 | [0–0.2] | – | – | – |  |
| 2016 | 1811 | 0.3 | [0.1–0.6] | – | – | – |  |
| 2017 | 1507 | 0.1 | [0–0.5] | 0.47 | [0.11–2.09] | 1.000 |  |
| 2018 | 1555 | 0.6 | [0.3–1.2] | 4.9 | [1.24–19.37] | 0.449 |  |
| 2019 | 1879 | 0.3 | [0.1–0.7] | 0.51 | [0.2–1.29] | 0.741 |  |
| 2020 | 2731 | 0.1 | [0–0.3] | 0.36 | [0.1–1.26] | 0.694 |  |
| 2021 | 2732 | 0.1 | [0–0.3] | 0.99 | [0.23–4.21] | 1.000 |  |
| 2022 | 3024 | 0.2 | [0.1–0.4] | 1.79 | [0.51–6.27] | 1.000 |  |
| 2023 | 2339 | 0.4 | [0.2–0.8] | 2.14 | [0.85–5.34] | 0.694 |  |
| 2024 | 1812 | 0.4 | [0.2–0.8] | 0.88 | [0.37–2.11] | 1.000 |  |

### Table S15: Annual percentage of Enterobacterales resistance to Cotrimoxazole from 2005 to 2024.

|  | Tested | Resistance | | Logistic model | |  | |
| --- | --- | --- | --- | --- | --- | --- | --- |
| Year | N | % | [95% CI] | OR | [95% CI] | P-value |  |
| 2005 | 2472 | 15.0 | [13.6–16.4] | – | – | – |  |
| 2006 | 2479 | 14.7 | [13.4–16.2] | 1 | [0.85–1.18] | 0.975 |  |
| 2007 | 2844 | 15.8 | [14.5–17.1] | 1.07 | [0.92–1.24] | 0.771 |  |
| 2008 | 2936 | 15.7 | [14.4–17.1] | 1.01 | [0.87–1.16] | 0.975 |  |
| 2009 | 3177 | 15.4 | [14.2–16.7] | 0.99 | [0.86–1.13] | 0.975 |  |
| 2010 | 3179 | 15.9 | [14.7–17.3] | 1.04 | [0.91–1.19] | 0.975 |  |
| 2011 | 3304 | 17.2 | [16–18.5] | 1.07 | [0.94–1.23] | 0.771 |  |
| 2012 | 3105 | 20.3 | [18.9–21.7] | 1.23 | [1.08–1.39] | 0.029 | * |
| 2013 | 2939 | 17.5 | [16.2–18.9] | 0.84 | [0.73–0.95] | 0.049 | * |
| 2014 | 3172 | 17.2 | [15.9–18.5] | 0.97 | [0.85–1.11] | 0.975 |  |
| 2015 | 2947 | 14.8 | [13.6–16.2] | 0.83 | [0.72–0.95] | 0.049 | * |
| 2016 | 3063 | 15.7 | [14.5–17] | 1.07 | [0.93–1.23] | 0.771 |  |
| 2017 | 2800 | 16.7 | [15.4–18.1] | 1.08 | [0.94–1.24] | 0.771 |  |
| 2018 | 3023 | 16.8 | [15.5–18.1] | 0.98 | [0.86–1.13] | 0.975 |  |
| 2019 | 3064 | 17.2 | [15.9–18.6] | 1 | [0.87–1.14] | 0.975 |  |
| 2020 | 3020 | 17.4 | [16–18.7] | 1.02 | [0.89–1.16] | 0.975 |  |
| 2021 | 3037 | 16.6 | [15.3–17.9] | 0.94 | [0.82–1.08] | 0.771 |  |
| 2022 | 3381 | 16.5 | [15.3–17.8] | 0.99 | [0.87–1.13] | 0.975 |  |
| 2023 | 3140 | 18.7 | [17.4–20.1] | 1.16 | [1.02–1.31] | 0.129 |  |
| 2024 | 2738 | 17.8 | [16.4–19.2] | 0.94 | [0.82–1.07] | 0.771 |  |

### Table S16: Annual percentage of Enterobacterales resistance to Piperacillin/Tazobactam from 2005 to 2024.

|  | Tested | Resistance | | Logistic model | |  | |
| --- | --- | --- | --- | --- | --- | --- | --- |
| Year | N | % | [95% CI] | OR | [95% CI] | P-value |  |
| 2005 | 1319 | 3.9 | [3–5.1] | – | – | – |  |
| 2006 | 1389 | 1.7 | [1.2–2.6] | 0.44 | [0.27–0.72] | 0.005 | ** |
| 2007 | 1609 | 1.1 | [0.7–1.7] | 0.55 | [0.29–1.04] | 0.213 |  |
| 2008 | 1626 | 1.6 | [1.1–2.3] | 1.57 | [0.84–2.94] | 0.400 |  |
| 2009 | 1826 | 4.2 | [3.3–5.2] | 2.73 | [1.74–4.28] | 0.000 | *** |
| 2010 | 1749 | 10.1 | [8.7–11.6] | 2.51 | [1.9–3.31] | 0.000 | *** |
| 2011 | 2290 | 7.9 | [6.9–9.1] | 0.74 | [0.59–0.92] | 0.025 | * |
| 2012 | 2326 | 7.0 | [6–8.1] | 0.88 | [0.71–1.1] | 0.468 |  |
| 2013 | 2930 | 10.1 | [9–11.2] | 1.51 | [1.24–1.84] | 0.000 | *** |
| 2014 | 3159 | 10.5 | [9.5–11.7] | 1.05 | [0.89–1.24] | 0.667 |  |
| 2015 | 2931 | 11.8 | [10.7–13.1] | 1.12 | [0.95–1.31] | 0.400 |  |
| 2016 | 2976 | 11.9 | [10.8–13.1] | 1.01 | [0.86–1.18] | 0.932 |  |
| 2017 | 2709 | 11.5 | [10.3–12.7] | 0.95 | [0.81–1.12] | 0.667 |  |
| 2018 | 2955 | 12.0 | [10.9–13.2] | 1.04 | [0.88–1.22] | 0.712 |  |
| 2019 | 3013 | 13.2 | [12–14.4] | 1.08 | [0.93–1.26] | 0.523 |  |
| 2020 | 2979 | 12.6 | [11.4–13.8] | 0.96 | [0.82–1.11] | 0.667 |  |
| 2021 | 3041 | 11.6 | [10.5–12.8] | 0.9 | [0.77–1.06] | 0.422 |  |
| 2022 | 3355 | 10.9 | [9.9–12] | 0.93 | [0.8–1.09] | 0.535 |  |
| 2023 | 3058 | 11.9 | [10.8–13.1] | 1.09 | [0.94–1.27] | 0.468 |  |
| 2024 | 2708 | 12.3 | [11.1–13.6] | 1.03 | [0.88–1.21] | 0.712 |  |

# C) 157 pathogen-drug combinations

## Enterobacterales

### Figure S1: Annual percentage of *Citrobacter freundii* resistance from 2005 to 2024


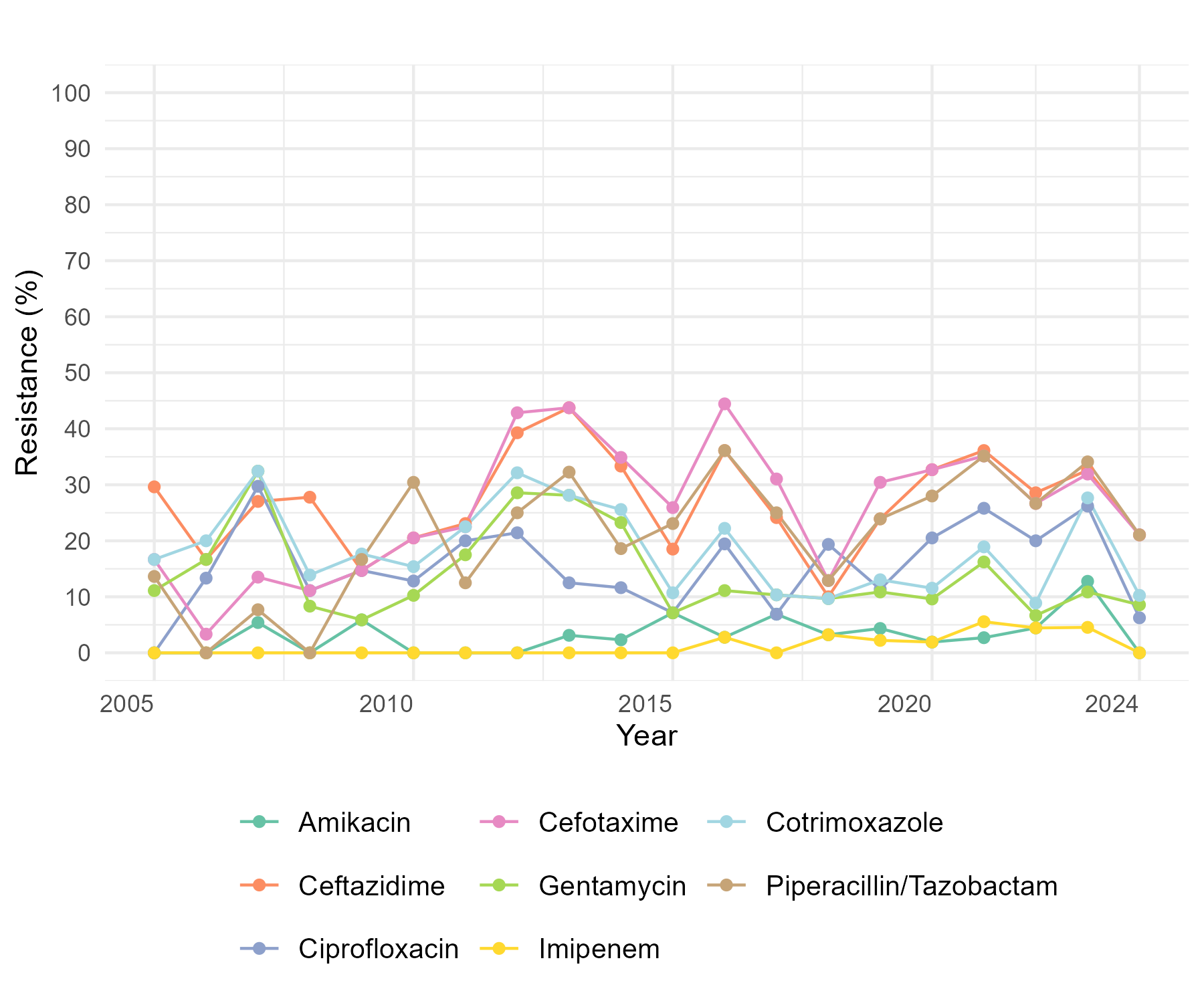


### Table S17: Logistic regression analysis of the effect of continuous time on the antibiotic resistance of *Citrobacter freundii* from 2005 to 2024.

| Antibiotic | β | OR | [95%CI] | P-value |  |
| --- | --- | --- | --- | --- | --- |
| Amikacin | 0.071 | 1.07 | [0.99–1.17] | 0.087 | . |
| Ceftazidime | 0.006 | 1.01 | [0.98–1.04] | 0.663 |  |
| Ciprofloxacin | 0.008 | 1.01 | [0.97–1.05] | 0.663 |  |
| Cefotaxime | 0.044 | 1.05 | [1.02–1.08] | 0.003 | ** |
| Gentamicin | -0.048 | 0.95 | [0.92–0.99] | 0.010 | ** |
| Imipenem | 0.234 | 1.26 | [1.1–1.46] | 0.001 | ** |
| Cotrimoxazole | -0.037 | 0.96 | [0.93–1] | 0.025 | * |
| Piperacillin/Tazobactam | 0.064 | 1.07 | [1.03–1.1] | 0.000 | *** |

### Table S18: Annual percentage of *Citrobacter freundii* resistance to Amikacin from 2005 to 2024.

|  | Tested | Resistance | | Logistic model | |  | |
| --- | --- | --- | --- | --- | --- | --- | --- |
| Year | N | % | [95% CI] | OR | [95% CI] | P-value |  |
| 2005 | 34 | 0.0 | [0–10.2] | – | – | – |  |
| 2006 | 26 | 0.0 | [0–12.9] | – | – | – |  |
| 2007 | 37 | 5.4 | [1.5–17.7] | – | – | – |  |
| 2008 | 36 | 0.0 | [0–9.6] | – | – | – |  |
| 2009 | 34 | 5.9 | [1.6–19.1] | – | – | – |  |
| 2010 | 39 | 0.0 | [0–9] | – | – | – |  |
| 2011 | 40 | 0.0 | [0–8.8] | – | – | – |  |
| 2012 | 28 | 0.0 | [0–12.1] | – | – | – |  |
| 2013 | 32 | 3.1 | [0.6–15.7] | – | – | – |  |
| 2014 | 43 | 2.3 | [0.4–12.1] | 0.76 | [0.03–17.91] | 1.000 |  |
| 2015 | 28 | 7.1 | [2–22.6] | 3.72 | [0.23–59.16] | 1.000 |  |
| 2016 | 36 | 2.8 | [0.5–14.2] | 0.38 | [0.02–6.12] | 1.000 |  |
| 2017 | 29 | 6.9 | [1.9–22] | 2.7 | [0.17–43.14] | 1.000 |  |
| 2018 | 31 | 3.2 | [0.6–16.2] | 0.45 | [0.03–7.44] | 1.000 |  |
| 2019 | 46 | 4.3 | [1.2–14.5] | 1.5 | [0.09–24.03] | 1.000 |  |
| 2020 | 52 | 1.9 | [0.3–10.1] | 0.36 | [0.02–5.53] | 1.000 |  |
| 2021 | 37 | 2.7 | [0.5–13.8] | 1.57 | [0.07–36.72] | 1.000 |  |
| 2022 | 45 | 4.4 | [1.2–14.8] | 1.46 | [0.09–22.79] | 1.000 |  |
| 2023 | 47 | 12.8 | [6–25.2] | 3.86 | [0.58–25.53] | 1.000 |  |
| 2024 | 39 | 0.0 | [0–9] | – | – | – |  |

### Table S19: Annual percentage of *Citrobacter freundii* resistance to Ceftazidime from 2005 to 2024.

|  | Tested | Resistance | | Logistic model | |  | |
| --- | --- | --- | --- | --- | --- | --- | --- |
| Year | N | % | [95% CI] | OR | [95% CI] | P-value |  |
| 2005 | 27 | 29.6 | [15.9–48.5] | – | – | – |  |
| 2006 | 30 | 16.7 | [7.3–33.6] | 0.45 | [0.12–1.65] | 0.617 |  |
| 2007 | 37 | 27.0 | [15.4–43] | 1.67 | [0.48–5.77] | 0.659 |  |
| 2008 | 36 | 27.8 | [15.8–44] | 0.95 | [0.33–2.74] | 0.972 |  |
| 2009 | 34 | 14.7 | [6.4–30.1] | 0.56 | [0.16–1.95] | 0.659 |  |
| 2010 | 39 | 20.5 | [10.8–35.5] | 1.27 | [0.36–4.52] | 0.845 |  |
| 2011 | 39 | 23.1 | [12.6–38.3] | 1.13 | [0.37–3.41] | 0.931 |  |
| 2012 | 28 | 39.3 | [23.6–57.6] | 2.35 | [0.79–7.04] | 0.617 |  |
| 2013 | 32 | 43.8 | [28.2–60.7] | 1 | [0.35–2.93] | 0.993 |  |
| 2014 | 42 | 33.3 | [21–48.4] | 0.67 | [0.25–1.78] | 0.659 |  |
| 2015 | 27 | 18.5 | [8.2–36.7] | 0.46 | [0.14–1.5] | 0.617 |  |
| 2016 | 36 | 36.1 | [22.5–52.4] | 2.73 | [0.8–9.24] | 0.617 |  |
| 2017 | 29 | 24.1 | [12.2–42.1] | 0.55 | [0.18–1.68] | 0.659 |  |
| 2018 | 30 | 10.0 | [3.5–25.6] | 0.35 | [0.08–1.56] | 0.617 |  |
| 2019 | 46 | 23.9 | [13.9–37.9] | 2.98 | [0.73–12.17] | 0.617 |  |
| 2020 | 52 | 32.7 | [21.5–46.2] | 1.42 | [0.57–3.56] | 0.659 |  |
| 2021 | 36 | 36.1 | [22.5–52.4] | 1.2 | [0.48–3] | 0.845 |  |
| 2022 | 42 | 28.6 | [17.2–43.6] | 0.66 | [0.25–1.76] | 0.659 |  |
| 2023 | 46 | 32.6 | [20.9–47] | 1.33 | [0.52–3.39] | 0.744 |  |
| 2024 | 38 | 21.1 | [11.1–36.3] | 0.52 | [0.19–1.45] | 0.617 |  |

### Table S20: Annual percentage of *Citrobacter freundii* resistance to Ciprofloxacin from 2005 to 2024.

|  | Tested | Resistance | | Logistic model | |  | |
| --- | --- | --- | --- | --- | --- | --- | --- |
| Year | N | % | [95% CI] | OR | [95% CI] | P-value |  |
| 2005 | 27 | 0.0 | [0–12.5] | – | – | – |  |
| 2006 | 30 | 13.3 | [5.3–29.7] | – | – | – |  |
| 2007 | 37 | 29.7 | [17.5–45.8] | 2.52 | [0.71–8.94] | 0.418 |  |
| 2008 | 36 | 11.1 | [4.4–25.3] | 0.22 | [0.06–0.77] | 0.263 |  |
| 2009 | 34 | 14.7 | [6.4–30.1] | 2.33 | [0.56–9.61] | 0.551 |  |
| 2010 | 39 | 12.8 | [5.6–26.7] | 0.57 | [0.15–2.2] | 0.551 |  |
| 2011 | 40 | 20.0 | [10.5–34.8] | 1.72 | [0.51–5.78] | 0.551 |  |
| 2012 | 28 | 21.4 | [10.2–39.5] | 1.24 | [0.38–4.06] | 0.814 |  |
| 2013 | 32 | 12.5 | [5–28.1] | 0.37 | [0.09–1.44] | 0.418 |  |
| 2014 | 43 | 11.6 | [5.1–24.5] | 0.97 | [0.24–3.87] | 0.982 |  |
| 2015 | 28 | 7.1 | [2–22.6] | 0.63 | [0.12–3.36] | 0.695 |  |
| 2016 | 36 | 19.4 | [9.8–35] | 3.4 | [0.67–17.38] | 0.418 |  |
| 2017 | 29 | 6.9 | [1.9–22] | 0.29 | [0.06–1.47] | 0.418 |  |
| 2018 | 31 | 19.4 | [9.2–36.3] | 3.85 | [0.72–20.52] | 0.418 |  |
| 2019 | 44 | 11.4 | [5–24] | 0.52 | [0.14–1.9] | 0.551 |  |
| 2020 | 39 | 20.5 | [10.8–35.5] | 1.69 | [0.51–5.64] | 0.551 |  |
| 2021 | 31 | 25.8 | [13.7–43.2] | 1.56 | [0.51–4.79] | 0.551 |  |
| 2022 | 35 | 20.0 | [10–35.9] | 0.6 | [0.19–1.89] | 0.551 |  |
| 2023 | 42 | 26.2 | [15.3–41.1] | 1.62 | [0.55–4.72] | 0.551 |  |
| 2024 | 32 | 6.2 | [1.7–20.1] | 0.17 | [0.04–0.82] | 0.263 |  |

### Table S21: Annual percentage of *Citrobacter freundii* resistance to Cefotaxime from 2005 to 2024.

|  | Tested | Resistance | | Logistic model | |  | |
| --- | --- | --- | --- | --- | --- | --- | --- |
| Year | N | % | [95% CI] | OR | [95% CI] | P-value |  |
| 2005 | 36 | 16.7 | [7.9–31.9] | – | – | – |  |
| 2006 | 30 | 3.3 | [0.6–16.7] | 0.17 | [0.02–1.56] | 0.446 |  |
| 2007 | 37 | 13.5 | [5.9–28] | 4.27 | [0.46–39.94] | 0.623 |  |
| 2008 | 36 | 11.1 | [4.4–25.3] | 0.74 | [0.18–3.1] | 0.877 |  |
| 2009 | 34 | 14.7 | [6.4–30.1] | 1.71 | [0.4–7.21] | 0.720 |  |
| 2010 | 39 | 20.5 | [10.8–35.5] | 1.29 | [0.37–4.54] | 0.877 |  |
| 2011 | 40 | 22.5 | [12.3–37.5] | 1.09 | [0.36–3.26] | 0.923 |  |
| 2012 | 28 | 42.9 | [26.5–60.9] | 2.75 | [0.93–8.07] | 0.446 |  |
| 2013 | 32 | 43.8 | [28.2–60.7] | 0.92 | [0.32–2.62] | 0.923 |  |
| 2014 | 43 | 34.9 | [22.4–49.8] | 0.71 | [0.27–1.85] | 0.720 |  |
| 2015 | 27 | 25.9 | [13.2–44.7] | 0.65 | [0.22–1.93] | 0.720 |  |
| 2016 | 36 | 44.4 | [29.5–60.4] | 2.43 | [0.8–7.36] | 0.446 |  |
| 2017 | 29 | 31.0 | [17.3–49.2] | 0.55 | [0.19–1.57] | 0.623 |  |
| 2018 | 31 | 12.9 | [5.1–28.9] | 0.34 | [0.09–1.3] | 0.446 |  |
| 2019 | 46 | 30.4 | [19.1–44.8] | 2.99 | [0.86–10.42] | 0.446 |  |
| 2020 | 52 | 32.7 | [21.5–46.2] | 1.04 | [0.44–2.5] | 0.923 |  |
| 2021 | 37 | 35.1 | [21.8–51.2] | 1.15 | [0.46–2.85] | 0.909 |  |
| 2022 | 45 | 26.7 | [16–41] | 0.63 | [0.24–1.66] | 0.720 |  |
| 2023 | 47 | 31.9 | [20.4–46.2] | 1.38 | [0.55–3.46] | 0.720 |  |
| 2024 | 38 | 21.1 | [11.1–36.3] | 0.55 | [0.2–1.51] | 0.623 |  |

### Table S22: Annual percentage of *Citrobacter freundii* resistance to Gentamicin from 2005 to 2024.

|  | Tested | Resistance | | Logistic model | |  | |
| --- | --- | --- | --- | --- | --- | --- | --- |
| Year | N | % | [95% CI] | OR | [95% CI] | P-value |  |
| 2005 | 36 | 11.1 | [4.4–25.3] | – | – | – |  |
| 2006 | 30 | 16.7 | [7.3–33.6] | 1.64 | [0.39–6.85] | 0.935 |  |
| 2007 | 37 | 32.4 | [19.6–48.5] | 2.26 | [0.68–7.54] | 0.828 |  |
| 2008 | 36 | 8.3 | [2.9–21.8] | 0.15 | [0.04–0.61] | 0.144 |  |
| 2009 | 34 | 5.9 | [1.6–19.1] | 1.01 | [0.15–6.53] | 0.995 |  |
| 2010 | 39 | 10.3 | [4.1–23.6] | 1.36 | [0.23–8.12] | 0.935 |  |
| 2011 | 40 | 17.5 | [8.7–31.9] | 1.85 | [0.49–7] | 0.935 |  |
| 2012 | 28 | 28.6 | [15.3–47.1] | 2.1 | [0.65–6.82] | 0.828 |  |
| 2013 | 32 | 28.1 | [15.6–45.4] | 0.78 | [0.24–2.46] | 0.935 |  |
| 2014 | 43 | 23.3 | [13.2–37.7] | 0.81 | [0.28–2.35] | 0.935 |  |
| 2015 | 28 | 7.1 | [2–22.6] | 0.26 | [0.05–1.28] | 0.828 |  |
| 2016 | 36 | 11.1 | [4.4–25.3] | 1.66 | [0.28–9.83] | 0.935 |  |
| 2017 | 29 | 10.3 | [3.6–26.4] | 0.91 | [0.19–4.48] | 0.995 |  |
| 2018 | 31 | 9.7 | [3.3–24.9] | 1.05 | [0.19–5.74] | 0.995 |  |
| 2019 | 46 | 10.9 | [4.7–23] | 1.12 | [0.25–5.15] | 0.995 |  |
| 2020 | 52 | 9.6 | [4.2–20.6] | 0.79 | [0.21–2.93] | 0.935 |  |
| 2021 | 37 | 16.2 | [7.7–31.1] | 2.01 | [0.56–7.23] | 0.903 |  |
| 2022 | 45 | 6.7 | [2.3–17.9] | 0.32 | [0.07–1.4] | 0.828 |  |
| 2023 | 46 | 10.9 | [4.7–23] | 1.83 | [0.41–8.17] | 0.935 |  |
| 2024 | 35 | 8.6 | [3–22.4] | 0.77 | [0.17–3.51] | 0.935 |  |

### Table S23: Annual percentage of *Citrobacter freundii* resistance to Imipenem from 2005 to 2024.

|  | Tested | Resistance | | Logistic model | |  | |
| --- | --- | --- | --- | --- | --- | --- | --- |
| Year | N | % | [95% CI] | OR | [95% CI] | P-value |  |
| 2005 | 22 | 0.0 | [0–14.9] | – | – | – |  |
| 2006 | 22 | 0.0 | [0–14.9] | – | – | – |  |
| 2007 | 27 | 0.0 | [0–12.5] | – | – | – |  |
| 2008 | 21 | 0.0 | [0–15.5] | – | – | – |  |
| 2009 | 24 | 0.0 | [0–13.8] | – | – | – |  |
| 2010 | 23 | 0.0 | [0–14.3] | – | – | – |  |
| 2011 | 40 | 0.0 | [0–8.8] | – | – | – |  |
| 2012 | 28 | 0.0 | [0–12.1] | – | – | – |  |
| 2013 | 32 | 0.0 | [0–10.7] | – | – | – |  |
| 2014 | 43 | 0.0 | [0–8.2] | – | – | – |  |
| 2015 | 28 | 0.0 | [0–12.1] | – | – | – |  |
| 2016 | 36 | 2.8 | [0.5–14.2] | – | – | – |  |
| 2017 | 28 | 0.0 | [0–12.1] | – | – | – |  |
| 2018 | 31 | 3.2 | [0.6–16.2] | – | – | – |  |
| 2019 | 45 | 2.2 | [0.4–11.6] | 0.75 | [0.12–4.88] | 1.000 |  |
| 2020 | 52 | 1.9 | [0.3–10.1] | 0.75 | [0.12–4.73] | 1.000 |  |
| 2021 | 36 | 5.6 | [1.5–18.1] | 3.06 | [0.61–15.37] | 1.000 |  |
| 2022 | 45 | 4.4 | [1.2–14.8] | 0.74 | [0.19–2.8] | 1.000 |  |
| 2023 | 44 | 4.5 | [1.3–15.1] | 1.23 | [0.32–4.68] | 1.000 |  |
| 2024 | 35 | 0.0 | [0–9.9] | – | – | – |  |

### Table S24: Annual percentage of *Citrobacter freundii* resistance to Cotrimoxazole from 2005 to 2024.

|  | Tested | Resistance | | Logistic model | |  | |
| --- | --- | --- | --- | --- | --- | --- | --- |
| Year | N | % | [95% CI] | OR | [95% CI] | P-value |  |
| 2005 | 36 | 16.7 | [7.9–31.9] | – | – | – |  |
| 2006 | 30 | 20.0 | [9.5–37.3] | 1.27 | [0.35–4.54] | 0.802 |  |
| 2007 | 37 | 32.4 | [19.6–48.5] | 1.74 | [0.55–5.53] | 0.631 |  |
| 2008 | 36 | 13.9 | [6.1–28.7] | 0.29 | [0.09–0.94] | 0.254 |  |
| 2009 | 34 | 17.6 | [8.3–33.5] | 1.85 | [0.49–7] | 0.631 |  |
| 2010 | 39 | 15.4 | [7.2–29.7] | 0.67 | [0.19–2.4] | 0.727 |  |
| 2011 | 40 | 22.5 | [12.3–37.5] | 1.55 | [0.48–4.99] | 0.690 |  |
| 2012 | 28 | 32.1 | [17.9–50.7] | 1.81 | [0.59–5.49] | 0.630 |  |
| 2013 | 32 | 28.1 | [15.6–45.4] | 0.66 | [0.21–2.05] | 0.690 |  |
| 2014 | 43 | 25.6 | [14.9–40.2] | 0.92 | [0.32–2.66] | 0.932 |  |
| 2015 | 28 | 10.7 | [3.7–27.2] | 0.35 | [0.09–1.41] | 0.558 |  |
| 2016 | 36 | 22.2 | [11.7–38.1] | 2.6 | [0.61–11.14] | 0.558 |  |
| 2017 | 29 | 10.3 | [3.6–26.4] | 0.39 | [0.09–1.67] | 0.558 |  |
| 2018 | 31 | 9.7 | [3.3–24.9] | 0.96 | [0.17–5.34] | 0.966 |  |
| 2019 | 46 | 13.0 | [6.1–25.7] | 1.45 | [0.33–6.41] | 0.796 |  |
| 2020 | 52 | 11.5 | [5.4–23] | 0.78 | [0.23–2.66] | 0.802 |  |
| 2021 | 37 | 18.9 | [9.5–34.2] | 1.89 | [0.57–6.28] | 0.630 |  |
| 2022 | 45 | 8.9 | [3.5–20.7] | 0.38 | [0.1–1.44] | 0.558 |  |
| 2023 | 47 | 27.7 | [16.9–41.8] | 4.46 | [1.31–15.18] | 0.254 |  |
| 2024 | 39 | 10.3 | [4.1–23.6] | 0.27 | [0.08–0.94] | 0.254 |  |

### Table S25: Annual percentage of *Citrobacter freundii* resistance to Piperacillin/Tazobactam from 2005 to 2024.

|  | Tested | Resistance | | Logistic model | |  | |
| --- | --- | --- | --- | --- | --- | --- | --- |
| Year | N | % | [95% CI] | OR | [95% CI] | P-value |  |
| 2005 | 22 | 13.6 | [4.7–33.3] | – | – | – |  |
| 2006 | 22 | 0.0 | [0–14.9] | – | – | – |  |
| 2007 | 26 | 7.7 | [2.1–24.1] | – | – | – |  |
| 2008 | 21 | 0.0 | [0–15.5] | – | – | – |  |
| 2009 | 24 | 16.7 | [6.7–35.9] | – | – | – |  |
| 2010 | 23 | 30.4 | [15.6–50.9] | 1.82 | [0.45–7.39] | 0.695 |  |
| 2011 | 40 | 12.5 | [5.5–26.1] | 0.32 | [0.09–1.16] | 0.555 |  |
| 2012 | 28 | 25.0 | [12.7–43.4] | 2.54 | [0.72–8.98] | 0.555 |  |
| 2013 | 31 | 32.3 | [18.6–49.9] | 1.22 | [0.39–3.82] | 0.972 |  |
| 2014 | 43 | 18.6 | [9.7–32.6] | 0.5 | [0.17–1.46] | 0.555 |  |
| 2015 | 26 | 23.1 | [11–42.1] | 1.3 | [0.4–4.28] | 0.966 |  |
| 2016 | 36 | 36.1 | [22.5–52.4] | 2.15 | [0.69–6.71] | 0.555 |  |
| 2017 | 28 | 25.0 | [12.7–43.4] | 0.59 | [0.2–1.74] | 0.695 |  |
| 2018 | 31 | 12.9 | [5.1–28.9] | 0.41 | [0.11–1.59] | 0.555 |  |
| 2019 | 46 | 23.9 | [13.9–37.9] | 2.26 | [0.65–7.85] | 0.555 |  |
| 2020 | 50 | 28.0 | [17.5–41.7] | 1.15 | [0.46–2.86] | 0.972 |  |
| 2021 | 37 | 35.1 | [21.8–51.2] | 1.37 | [0.55–3.41] | 0.791 |  |
| 2022 | 45 | 26.7 | [16–41] | 0.66 | [0.26–1.69] | 0.695 |  |
| 2023 | 44 | 34.1 | [21.9–48.9] | 1.57 | [0.63–3.9] | 0.695 |  |
| 2024 | 38 | 21.1 | [11.1–36.3] | 0.47 | [0.17–1.28] | 0.555 |  |

### Figure S2: Annual percentage of *Citrobacter kosseri* resistance from 2005 to 2024


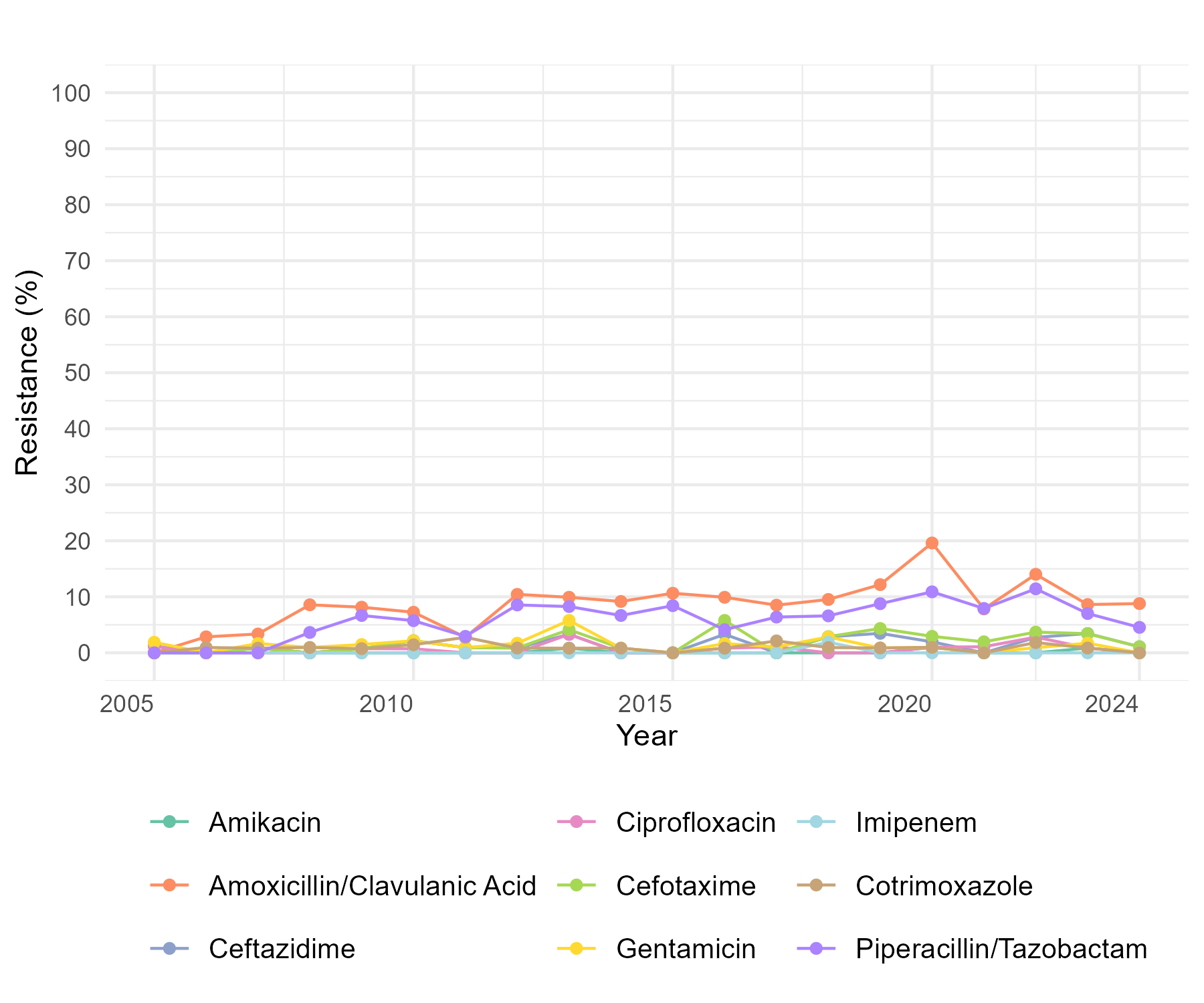


### Table S26: Logistic regression analysis of the effect of continuous time on the antibiotic resistance of *Citrobacter kosseri* from 2005 to 2024.

| Antibiotic | β | OR | [95%CI] | P-value |  |
| --- | --- | --- | --- | --- | --- |
| Amikacin | -0.043 | 0.96 | [0.82–1.12] | 0.581 |  |
| Amoxicillin/Clavulanic Acid | 0.058 | 1.06 | [1.03–1.09] | 0.000 | *** |
| Ceftazidime | 0.063 | 1.06 | [1–1.14] | 0.056 | . |
| Ciprofloxacin | 0.049 | 1.05 | [0.96–1.15] | 0.300 |  |
| Cefotaxime | 0.073 | 1.08 | [1.02–1.14] | 0.009 | ** |
| Gentamicin | -0.035 | 0.97 | [0.91–1.03] | 0.269 |  |
| Imipenem | 0.075 | 1.08 | [0.88–1.31] | 0.456 |  |
| Cotrimoxazole | -0.020 | 0.98 | [0.91–1.06] | 0.600 |  |
| Piperacillin/Tazobactam | 0.060 | 1.06 | [1.02–1.1] | 0.001 | ** |

### Table S27: Annual percentage of *Citrobacter kosseri* resistance to Amikacin from 2005 to 2024.

|  | Tested | Resistance | | Logistic model | |  | |
| --- | --- | --- | --- | --- | --- | --- | --- |
| Year | N | % | [95% CI] | OR | [95% CI] | P-value |  |
| 2005 | 82 | 1.2 | [0.2–6.6] | – | – | – |  |
| 2006 | 73 | 0.0 | [0–5] | – | – | – |  |
| 2007 | 120 | 0.8 | [0.1–4.6] | – | – | – |  |
| 2008 | 105 | 0.0 | [0–3.5] | – | – | – |  |
| 2009 | 135 | 0.0 | [0–2.8] | – | – | – |  |
| 2010 | 139 | 0.0 | [0–2.7] | – | – | – |  |
| 2011 | 108 | 0.0 | [0–3.4] | – | – | – |  |
| 2012 | 117 | 0.0 | [0–3.2] | – | – | – |  |
| 2013 | 121 | 0.8 | [0.1–4.5] | – | – | – |  |
| 2014 | 120 | 0.0 | [0–3.1] | – | – | – |  |
| 2015 | 95 | 0.0 | [0–3.9] | – | – | – |  |
| 2016 | 121 | 0.0 | [0–3.1] | – | – | – |  |
| 2017 | 94 | 0.0 | [0–3.9] | – | – | – |  |
| 2018 | 106 | 0.0 | [0–3.5] | – | – | – |  |
| 2019 | 115 | 0.0 | [0–3.2] | – | – | – |  |
| 2020 | 102 | 1.0 | [0.2–5.3] | – | – | – |  |
| 2021 | 102 | 0.0 | [0–3.6] | – | – | – |  |
| 2022 | 109 | 0.0 | [0–3.4] | – | – | – |  |
| 2023 | 117 | 0.9 | [0.2–4.7] | – | – | – |  |
| 2024 | 92 | 0.0 | [0–4] | – | – | – |  |

### Table S28: Annual percentage of *Citrobacter kosseri* resistance to Amoxicillin/Clavulanic Acid from 2005 to 2024.

|  | Tested | Resistance | | Logistic model | |  | |
| --- | --- | --- | --- | --- | --- | --- | --- |
| Year | N | % | [95% CI] | OR | [95% CI] | P-value |  |
| 2005 | 106 | 0.0 | [0–3.5] | – | – | – |  |
| 2006 | 105 | 2.9 | [1–8.1] | – | – | – |  |
| 2007 | 119 | 3.4 | [1.3–8.3] | 1.11 | [0.25–4.96] | 0.975 |  |
| 2008 | 105 | 8.6 | [4.6–15.5] | 2.7 | [0.82–8.85] | 0.419 |  |
| 2009 | 135 | 8.1 | [4.6–14] | 0.98 | [0.39–2.41] | 0.975 |  |
| 2010 | 138 | 7.2 | [4–12.8] | 0.88 | [0.36–2.11] | 0.975 |  |
| 2011 | 108 | 2.8 | [0.9–7.9] | 0.36 | [0.1–1.32] | 0.419 |  |
| 2012 | 115 | 10.4 | [6.1–17.4] | 4.06 | [1.14–14.47] | 0.289 |  |
| 2013 | 121 | 9.9 | [5.8–16.5] | 0.95 | [0.41–2.18] | 0.975 |  |
| 2014 | 120 | 9.2 | [5.2–15.7] | 0.89 | [0.38–2.08] | 0.975 |  |
| 2015 | 94 | 10.6 | [5.9–18.5] | 1.18 | [0.49–2.87] | 0.975 |  |
| 2016 | 121 | 9.9 | [5.8–16.5] | 0.91 | [0.38–2.18] | 0.975 |  |
| 2017 | 94 | 8.5 | [4.4–15.9] | 0.85 | [0.34–2.14] | 0.975 |  |
| 2018 | 105 | 9.5 | [5.3–16.6] | 1.11 | [0.43–2.89] | 0.975 |  |
| 2019 | 115 | 12.2 | [7.4–19.4] | 1.32 | [0.57–3.06] | 0.975 |  |
| 2020 | 102 | 19.6 | [13.1–28.4] | 1.77 | [0.85–3.67] | 0.419 |  |
| 2021 | 102 | 7.8 | [4–14.7] | 0.34 | [0.14–0.79] | 0.239 |  |
| 2022 | 107 | 14.0 | [8.7–21.8] | 1.98 | [0.81–4.83] | 0.419 |  |
| 2023 | 116 | 8.6 | [4.7–15.1] | 0.57 | [0.25–1.31] | 0.503 |  |
| 2024 | 91 | 8.8 | [4.5–16.4] | 1.02 | [0.39–2.64] | 0.975 |  |

### Table S29: Annual percentage of *Citrobacter kosseri* resistance to Ceftazidime from 2005 to 2024.

|  | Tested | Resistance | | Logistic model | |  | |
| --- | --- | --- | --- | --- | --- | --- | --- |
| Year | N | % | [95% CI] | OR | [95% CI] | P-value |  |
| 2005 | 88 | 0.0 | [0–4.2] | – | – | – |  |
| 2006 | 105 | 0.0 | [0–3.5] | – | – | – |  |
| 2007 | 120 | 0.8 | [0.1–4.6] | – | – | – |  |
| 2008 | 105 | 0.0 | [0–3.5] | – | – | – |  |
| 2009 | 135 | 0.7 | [0.1–4.1] | – | – | – |  |
| 2010 | 139 | 2.2 | [0.7–6.2] | 2.68 | [0.44–16.37] | 1.000 |  |
| 2011 | 108 | 0.9 | [0.2–5.1] | 0.43 | [0.07–2.59] | 1.000 |  |
| 2012 | 117 | 0.9 | [0.2–4.7] | 0.9 | [0.1–8.19] | 1.000 |  |
| 2013 | 121 | 3.3 | [1.3–8.2] | 4.08 | [0.71–23.5] | 1.000 |  |
| 2014 | 120 | 0.0 | [0–3.1] | – | – | – |  |
| 2015 | 95 | 0.0 | [0–3.9] | – | – | – |  |
| 2016 | 121 | 3.3 | [1.3–8.2] | – | – | – |  |
| 2017 | 93 | 0.0 | [0–4] | – | – | – |  |
| 2018 | 105 | 2.9 | [1–8.1] | – | – | – |  |
| 2019 | 115 | 3.5 | [1.4–8.6] | 1.21 | [0.36–4.06] | 1.000 |  |
| 2020 | 102 | 2.0 | [0.5–6.9] | 0.55 | [0.14–2.17] | 1.000 |  |
| 2021 | 97 | 0.0 | [0–3.8] | – | – | – |  |
| 2022 | 109 | 2.8 | [0.9–7.8] | – | – | – |  |
| 2023 | 116 | 3.4 | [1.3–8.5] | 1.24 | [0.37–4.16] | 1.000 |  |
| 2024 | 92 | 1.1 | [0.2–5.9] | 0.3 | [0.05–1.74] | 1.000 |  |

### Table S30: Annual percentage of *Citrobacter kosseri* resistance to Ciprofloxacin from 2005 to 2024.

|  | Tested | Resistance | | Logistic model | |  | |
| --- | --- | --- | --- | --- | --- | --- | --- |
| Year | N | % | [95% CI] | OR | [95% CI] | P-value |  |
| 2005 | 88 | 1.1 | [0.2–6.2] | – | – | – |  |
| 2006 | 105 | 0.0 | [0–3.5] | – | – | – |  |
| 2007 | 120 | 0.0 | [0–3.1] | – | – | – |  |
| 2008 | 105 | 0.0 | [0–3.5] | – | – | – |  |
| 2009 | 135 | 0.7 | [0.1–4.1] | – | – | – |  |
| 2010 | 139 | 0.7 | [0.1–4] | 0.82 | [0.1–6.56] | 1.000 |  |
| 2011 | 108 | 0.0 | [0–3.4] | – | – | – |  |
| 2012 | 117 | 0.0 | [0–3.2] | – | – | – |  |
| 2013 | 121 | 3.3 | [1.3–8.2] | – | – | – |  |
| 2014 | 120 | 0.0 | [0–3.1] | – | – | – |  |
| 2015 | 95 | 0.0 | [0–3.9] | – | – | – |  |
| 2016 | 121 | 0.8 | [0.1–4.5] | – | – | – |  |
| 2017 | 94 | 1.1 | [0.2–5.8] | 1.28 | [0.16–10.24] | 1.000 |  |
| 2018 | 106 | 0.0 | [0–3.5] | – | – | – |  |
| 2019 | 113 | 0.0 | [0–3.3] | – | – | – |  |
| 2020 | 100 | 1.0 | [0.2–5.4] | – | – | – |  |
| 2021 | 92 | 1.1 | [0.2–5.9] | 1 | [0.12–8.01] | 1.000 |  |
| 2022 | 107 | 2.8 | [1–7.9] | 3.02 | [0.55–16.71] | 1.000 |  |
| 2023 | 115 | 0.9 | [0.2–4.8] | 0.32 | [0.06–1.75] | 1.000 |  |
| 2024 | 87 | 0.0 | [0–4.2] | – | – | – |  |

### Table S31: Annual percentage of *Citrobacter kosseri* resistance to Cefotaxime from 2005 to 2024.

|  | Tested | Resistance | | Logistic model | |  | |
| --- | --- | --- | --- | --- | --- | --- | --- |
| Year | N | % | [95% CI] | OR | [95% CI] | P-value |  |
| 2005 | 106 | 0.0 | [0–3.5] | – | – | – |  |
| 2006 | 105 | 0.0 | [0–3.5] | – | – | – |  |
| 2007 | 119 | 0.8 | [0.1–4.6] | – | – | – |  |
| 2008 | 105 | 0.0 | [0–3.5] | – | – | – |  |
| 2009 | 135 | 0.7 | [0.1–4.1] | – | – | – |  |
| 2010 | 138 | 2.2 | [0.7–6.2] | 2.61 | [0.37–18.53] | 1.000 |  |
| 2011 | 108 | 0.9 | [0.2–5.1] | 0.42 | [0.06–3.02] | 1.000 |  |
| 2012 | 114 | 0.9 | [0.2–4.8] | 0.91 | [0.08–9.99] | 1.000 |  |
| 2013 | 121 | 4.1 | [1.8–9.3] | 5.1 | [0.79–32.83] | 0.822 |  |
| 2014 | 120 | 0.8 | [0.1–4.6] | 0.18 | [0.03–1.13] | 0.822 |  |
| 2015 | 95 | 0.0 | [0–3.9] | – | – | – |  |
| 2016 | 121 | 5.8 | [2.8–11.5] | – | – | – |  |
| 2017 | 94 | 0.0 | [0–3.9] | – | – | – |  |
| 2018 | 104 | 2.9 | [1–8.1] | – | – | – |  |
| 2019 | 115 | 4.3 | [1.9–9.8] | 1.5 | [0.43–5.28] | 1.000 |  |
| 2020 | 102 | 2.9 | [1–8.3] | 0.66 | [0.19–2.31] | 1.000 |  |
| 2021 | 102 | 2.0 | [0.5–6.9] | 0.59 | [0.12–2.79] | 1.000 |  |
| 2022 | 108 | 3.7 | [1.4–9.1] | 2.22 | [0.5–9.79] | 1.000 |  |
| 2023 | 117 | 3.4 | [1.3–8.5] | 0.92 | [0.27–3.11] | 1.000 |  |
| 2024 | 92 | 1.1 | [0.2–5.9] | 0.3 | [0.05–2.03] | 1.000 |  |

### Table S32: Annual percentage of *Citrobacter kosseri* resistance to Gentamicin from 2005 to 2024.

|  | Tested | Resistance | | Logistic model | |  | |
| --- | --- | --- | --- | --- | --- | --- | --- |
| Year | N | % | [95% CI] | OR | [95% CI] | P-value |  |
| 2005 | 106 | 1.9 | [0.5–6.6] | – | – | – |  |
| 2006 | 105 | 0.0 | [0–3.5] | – | – | – |  |
| 2007 | 120 | 1.7 | [0.5–5.9] | – | – | – |  |
| 2008 | 105 | 1.0 | [0.2–5.2] | 0.62 | [0.07–5.55] | 0.992 |  |
| 2009 | 135 | 1.5 | [0.4–5.2] | 1.6 | [0.18–14.3] | 0.992 |  |
| 2010 | 139 | 2.2 | [0.7–6.2] | 1.28 | [0.25–6.62] | 0.992 |  |
| 2011 | 108 | 0.9 | [0.2–5.1] | 0.43 | [0.05–3.38] | 0.992 |  |
| 2012 | 117 | 1.7 | [0.5–6] | 1.82 | [0.2–16.24] | 0.992 |  |
| 2013 | 121 | 5.8 | [2.8–11.5] | 3.64 | [0.86–15.45] | 0.764 |  |
| 2014 | 120 | 0.8 | [0.1–4.6] | 0.13 | [0.02–0.87] | 0.667 |  |
| 2015 | 95 | 0.0 | [0–3.9] | – | – | – |  |
| 2016 | 121 | 1.7 | [0.5–5.8] | – | – | – |  |
| 2017 | 94 | 1.1 | [0.2–5.8] | 0.66 | [0.07–5.88] | 0.992 |  |
| 2018 | 106 | 2.8 | [1–8] | 2.74 | [0.35–21.69] | 0.992 |  |
| 2019 | 115 | 0.9 | [0.2–4.8] | 0.29 | [0.04–2.31] | 0.992 |  |
| 2020 | 102 | 1.0 | [0.2–5.3] | 1.11 | [0.09–13.79] | 0.992 |  |
| 2021 | 97 | 0.0 | [0–3.8] | – | – | – |  |
| 2022 | 109 | 0.9 | [0.2–5] | – | – | – |  |
| 2023 | 116 | 1.7 | [0.5–6.1] | 1.92 | [0.22–17.14] | 0.992 |  |
| 2024 | 89 | 0.0 | [0–4.1] | – | – | – |  |

### Table S33: Annual percentage of *Citrobacter kosseri* resistance to Imipenem from 2005 to 2024.

|  | Tested | Resistance | | Logistic model | |  | |
| --- | --- | --- | --- | --- | --- | --- | --- |
| Year | N | % | [95% CI] | OR | [95% CI] | P-value |  |
| 2005 | 60 | 0.0 | [0–6] | – | – | – |  |
| 2006 | 55 | 0.0 | [0–6.5] | – | – | – |  |
| 2007 | 66 | 0.0 | [0–5.5] | – | – | – |  |
| 2008 | 55 | 0.0 | [0–6.5] | – | – | – |  |
| 2009 | 90 | 0.0 | [0–4.1] | – | – | – |  |
| 2010 | 87 | 0.0 | [0–4.2] | – | – | – |  |
| 2011 | 102 | 0.0 | [0–3.6] | – | – | – |  |
| 2012 | 117 | 0.0 | [0–3.2] | – | – | – |  |
| 2013 | 121 | 0.0 | [0–3.1] | – | – | – |  |
| 2014 | 120 | 0.0 | [0–3.1] | – | – | – |  |
| 2015 | 94 | 0.0 | [0–3.9] | – | – | – |  |
| 2016 | 121 | 0.0 | [0–3.1] | – | – | – |  |
| 2017 | 94 | 0.0 | [0–3.9] | – | – | – |  |
| 2018 | 106 | 1.9 | [0.5–6.6] | – | – | – |  |
| 2019 | 113 | 0.0 | [0–3.3] | – | – | – |  |
| 2020 | 102 | 0.0 | [0–3.6] | – | – | – |  |
| 2021 | 99 | 0.0 | [0–3.7] | – | – | – |  |
| 2022 | 109 | 0.0 | [0–3.4] | – | – | – |  |
| 2023 | 103 | 0.0 | [0–3.6] | – | – | – |  |
| 2024 | 62 | 0.0 | [0–5.8] | – | – | – |  |

### Table S34: Annual percentage of *Citrobacter kosseri* resistance to Cotrimoxazole from 2005 to 2024.

|  | Tested | Resistance | | Logistic model | |  | |
| --- | --- | --- | --- | --- | --- | --- | --- |
| Year | N | % | [95% CI] | OR | [95% CI] | P-value |  |
| 2005 | 106 | 0.0 | [0–3.5] | – | – | – |  |
| 2006 | 105 | 1.0 | [0.2–5.2] | – | – | – |  |
| 2007 | 120 | 0.8 | [0.1–4.6] | 0.78 | [0.07–9.2] | 0.995 |  |
| 2008 | 105 | 1.0 | [0.2–5.2] | 1.26 | [0.11–14.76] | 0.995 |  |
| 2009 | 134 | 0.7 | [0.1–4.1] | 0.84 | [0.07–9.85] | 0.995 |  |
| 2010 | 139 | 1.4 | [0.4–5.1] | 1.73 | [0.2–14.67] | 0.995 |  |
| 2011 | 108 | 2.8 | [0.9–7.9] | 1.98 | [0.4–9.79] | 0.995 |  |
| 2012 | 117 | 0.9 | [0.2–4.7] | 0.29 | [0.04–2.19] | 0.995 |  |
| 2013 | 121 | 0.8 | [0.1–4.5] | 0.99 | [0.08–11.62] | 0.995 |  |
| 2014 | 120 | 0.8 | [0.1–4.6] | 0.91 | [0.08–10.66] | 0.995 |  |
| 2015 | 95 | 0.0 | [0–3.9] | – | – | – |  |
| 2016 | 121 | 0.8 | [0.1–4.5] | – | – | – |  |
| 2017 | 94 | 2.1 | [0.6–7.4] | 2.71 | [0.32–22.92] | 0.995 |  |
| 2018 | 106 | 0.9 | [0.2–5.2] | 0.42 | [0.05–3.58] | 0.995 |  |
| 2019 | 115 | 0.9 | [0.2–4.8] | 0.91 | [0.08–10.64] | 0.995 |  |
| 2020 | 102 | 1.0 | [0.2–5.3] | 1.12 | [0.1–13.17] | 0.995 |  |
| 2021 | 100 | 0.0 | [0–3.7] | – | – | – |  |
| 2022 | 109 | 1.8 | [0.5–6.4] | – | – | – |  |
| 2023 | 117 | 0.9 | [0.2–4.7] | 0.46 | [0.05–3.86] | 0.995 |  |
| 2024 | 92 | 0.0 | [0–4] | – | – | – |  |

### Table S35: Annual percentage of *Citrobacter kosseri* resistance to Piperacillin/Tazobactam from 2005 to 2024.

|  | Tested | Resistance | | Logistic model | |  | |
| --- | --- | --- | --- | --- | --- | --- | --- |
| Year | N | % | [95% CI] | OR | [95% CI] | P-value |  |
| 2005 | 60 | 0.0 | [0–6] | – | – | – |  |
| 2006 | 58 | 0.0 | [0–6.2] | – | – | – |  |
| 2007 | 66 | 0.0 | [0–5.5] | – | – | – |  |
| 2008 | 55 | 3.6 | [1–12.3] | – | – | – |  |
| 2009 | 90 | 6.7 | [3.1–13.8] | 1.95 | [0.41–9.35] | 0.918 |  |
| 2010 | 87 | 5.7 | [2.5–12.8] | 0.87 | [0.27–2.81] | 1.000 |  |
| 2011 | 102 | 2.9 | [1–8.3] | 0.49 | [0.12–1.99] | 0.918 |  |
| 2012 | 117 | 8.5 | [4.7–15] | 3.1 | [0.88–10.95] | 0.918 |  |
| 2013 | 121 | 8.3 | [4.6–14.5] | 0.96 | [0.4–2.32] | 1.000 |  |
| 2014 | 120 | 6.7 | [3.4–12.6] | 0.8 | [0.32–2.01] | 0.918 |  |
| 2015 | 95 | 8.4 | [4.3–15.7] | 1.3 | [0.49–3.44] | 0.918 |  |
| 2016 | 121 | 4.1 | [1.8–9.3] | 0.47 | [0.16–1.42] | 0.918 |  |
| 2017 | 94 | 6.4 | [3–13.2] | 1.58 | [0.49–5.09] | 0.918 |  |
| 2018 | 106 | 6.6 | [3.2–13] | 1.03 | [0.35–3.03] | 1.000 |  |
| 2019 | 114 | 8.8 | [4.8–15.4] | 1.37 | [0.52–3.58] | 0.918 |  |
| 2020 | 101 | 10.9 | [6.2–18.5] | 1.28 | [0.54–3.02] | 0.918 |  |
| 2021 | 101 | 7.9 | [4.1–14.9] | 0.71 | [0.28–1.77] | 0.918 |  |
| 2022 | 105 | 11.4 | [6.7–18.9] | 1.48 | [0.6–3.64] | 0.918 |  |
| 2023 | 114 | 7.0 | [3.6–13.2] | 0.58 | [0.24–1.42] | 0.918 |  |
| 2024 | 88 | 4.5 | [1.8–11.1] | 0.63 | [0.19–2.05] | 0.918 |  |

### Figure S3: Annual percentage of *Escherichia coli* resistance from 2005 to 2024


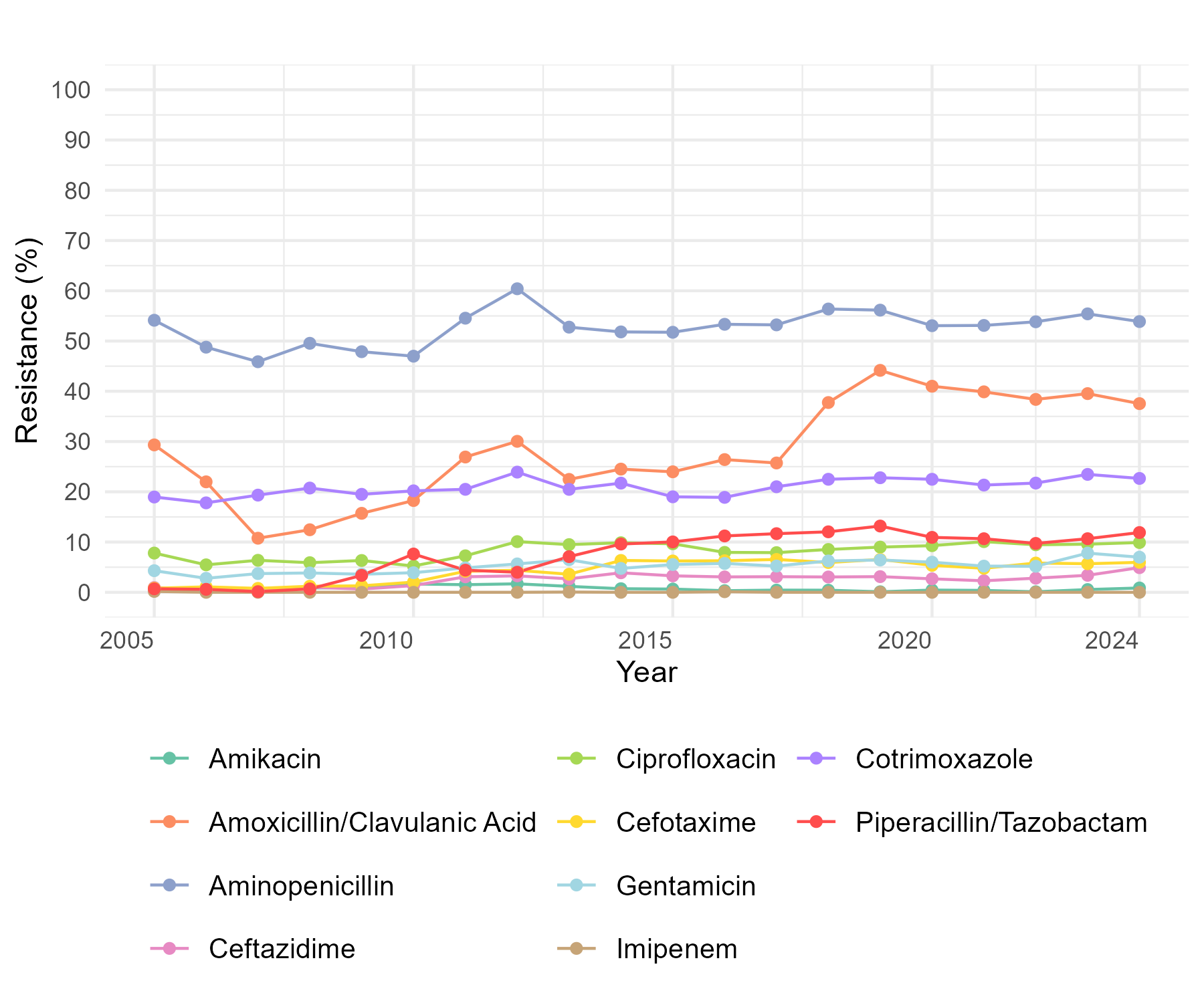


### Table S36: Logistic regression analysis of the effect of continuous time on the antibiotic resistance of *Escherichia coli* from 2005 to 2024.

| Antibiotic | β | OR | [95%CI] | P-value |  |
| --- | --- | --- | --- | --- | --- |
| Amikacin | -0.073 | 0.93 | [0.91–0.95] | 0.000 | *** |
| Amoxicillin/Clavulanic Acid | 0.065 | 1.07 | [1.06–1.07] | 0.000 | *** |
| Aminopenicillin | 0.005 | 1.01 | [1–1.01] | 0.008 | ** |
| Ceftazidime | 0.045 | 1.05 | [1.03–1.06] | 0.000 | *** |
| Ciprofloxacin | 0.005 | 1.00 | [1–1.01] | 0.209 |  |
| Cefotaxime | 0.061 | 1.06 | [1.05–1.07] | 0.000 | *** |
| Gentamicin | 0.022 | 1.02 | [1.01–1.03] | 0.000 | *** |
| Imipenem | -0.194 | 0.82 | [0.65–1.05] | 0.111 |  |
| Cotrimoxazole | 0.007 | 1.01 | [1–1.01] | 0.004 | ** |
| Piperacillin/Tazobactam | 0.070 | 1.07 | [1.06–1.08] | 0.000 | *** |

### Table S37: Annual percentage of *Escherichia coli* resistance to Amikacin from 2005 to 2024.

|  | Tested | Resistance | | Logistic model | |  | |
| --- | --- | --- | --- | --- | --- | --- | --- |
| Year | N | % | [95% CI] | OR | [95% CI] | P-value |  |
| 2005 | 1081 | 0.6 | [0.3–1.2] | – | – | – |  |
| 2006 | 892 | 0.1 | [0–0.6] | 0.22 | [0.03–1.74] | 0.318 |  |
| 2007 | 1665 | 0.6 | [0.3–1.1] | 4.77 | [0.63–36.01] | 0.318 |  |
| 2008 | 1663 | 0.8 | [0.5–1.4] | 1.42 | [0.64–3.16] | 0.620 |  |
| 2009 | 1863 | 1.0 | [0.7–1.6] | 1.24 | [0.62–2.45] | 0.744 |  |
| 2010 | 1873 | 1.7 | [1.2–2.3] | 1.6 | [0.91–2.83] | 0.318 |  |
| 2011 | 1913 | 1.5 | [1.1–2.2] | 0.87 | [0.53–1.44] | 0.744 |  |
| 2012 | 1894 | 1.7 | [1.2–2.4] | 1.14 | [0.69–1.87] | 0.744 |  |
| 2013 | 1865 | 1.2 | [0.8–1.8] | 0.68 | [0.4–1.17] | 0.318 |  |
| 2014 | 1956 | 0.7 | [0.4–1.2] | 0.61 | [0.31–1.18] | 0.318 |  |
| 2015 | 1862 | 0.6 | [0.4–1.1] | 0.87 | [0.41–1.86] | 0.771 |  |
| 2016 | 1862 | 0.3 | [0.1–0.7] | 0.49 | [0.19–1.3] | 0.318 |  |
| 2017 | 1838 | 0.4 | [0.2–0.9] | 1.3 | [0.46–3.67] | 0.744 |  |
| 2018 | 1907 | 0.4 | [0.2–0.8] | 0.92 | [0.35–2.4] | 0.857 |  |
| 2019 | 1898 | 0.1 | [0–0.4] | 0.24 | [0.05–1.08] | 0.318 |  |
| 2020 | 1804 | 0.4 | [0.2–0.9] | 4.31 | [0.94–19.77] | 0.318 |  |
| 2021 | 1841 | 0.4 | [0.2–0.8] | 0.84 | [0.31–2.28] | 0.771 |  |
| 2022 | 1907 | 0.1 | [0–0.4] | 0.27 | [0.06–1.27] | 0.318 |  |
| 2023 | 1830 | 0.5 | [0.3–1] | 5.05 | [1.13–22.45] | 0.318 |  |
| 2024 | 1627 | 0.9 | [0.5–1.4] | 1.59 | [0.71–3.54] | 0.441 |  |

### Table S38: Annual percentage of *Escherichia coli* resistance to Amoxicillin/Clavulanic Acid from 2005 to 2024.

|  | Tested | Resistance | | Logistic model | |  | |
| --- | --- | --- | --- | --- | --- | --- | --- |
| Year | N | % | [95% CI] | OR | [95% CI] | P-value |  |
| 2005 | 1367 | 29.3 | [27–31.8] | – | – | – |  |
| 2006 | 1474 | 22.0 | [19.9–24.2] | 0.71 | [0.59–0.84] | 0.000 | *** |
| 2007 | 1663 | 10.8 | [9.4–12.3] | 0.4 | [0.33–0.49] | 0.000 | *** |
| 2008 | 1663 | 12.4 | [10.9–14.1] | 1.19 | [0.96–1.47] | 0.179 |  |
| 2009 | 1864 | 15.7 | [14.1–17.4] | 1.3 | [1.07–1.58] | 0.020 | * |
| 2010 | 1873 | 18.3 | [16.6–20.1] | 1.18 | [1–1.4] | 0.117 |  |
| 2011 | 1913 | 26.9 | [25–29] | 1.63 | [1.39–1.9] | 0.000 | *** |
| 2012 | 1894 | 30.0 | [28–32.1] | 1.17 | [1.02–1.35] | 0.067 | . |
| 2013 | 1865 | 22.5 | [20.6–24.4] | 0.67 | [0.58–0.78] | 0.000 | *** |
| 2014 | 1954 | 24.5 | [22.7–26.5] | 1.13 | [0.97–1.31] | 0.179 |  |
| 2015 | 1860 | 24.0 | [22.1–26] | 0.96 | [0.83–1.11] | 0.595 |  |
| 2016 | 1863 | 26.4 | [24.5–28.5] | 1.14 | [0.98–1.32] | 0.156 |  |
| 2017 | 1838 | 25.7 | [23.8–27.8] | 0.95 | [0.82–1.11] | 0.594 |  |
| 2018 | 1904 | 37.8 | [35.6–40] | 1.72 | [1.5–1.98] | 0.000 | *** |
| 2019 | 1893 | 44.2 | [41.9–46.4] | 1.27 | [1.12–1.45] | 0.001 | *** |
| 2020 | 1807 | 41.0 | [38.8–43.3] | 0.88 | [0.77–1.01] | 0.118 |  |
| 2021 | 1840 | 39.9 | [37.7–42.1] | 0.95 | [0.83–1.08] | 0.493 |  |
| 2022 | 1907 | 38.4 | [36.2–40.6] | 0.93 | [0.82–1.07] | 0.396 |  |
| 2023 | 1821 | 39.5 | [37.3–41.8] | 1.04 | [0.91–1.18] | 0.595 |  |
| 2024 | 1622 | 37.5 | [35.2–39.9] | 0.92 | [0.8–1.06] | 0.322 |  |

### Table S39: Annual percentage of *Escherichia coli* resistance to Aminopenicillin from 2005 to 2024.

|  | Tested | Resistance | | Logistic model | |  | |
| --- | --- | --- | --- | --- | --- | --- | --- |
| Year | N | % | [95% CI] | OR | [95% CI] | P-value |  |
| 2005 | 1367 | 54.1 | [51.5–56.8] | – | – | – |  |
| 2006 | 1474 | 48.8 | [46.2–51.3] | 0.85 | [0.73–0.99] | 0.109 |  |
| 2007 | 1663 | 45.9 | [43.5–48.3] | 0.85 | [0.73–0.98] | 0.109 |  |
| 2008 | 1663 | 49.5 | [47.1–52] | 1.17 | [1.02–1.34] | 0.109 |  |
| 2009 | 1863 | 47.9 | [45.6–50.2] | 0.92 | [0.8–1.05] | 0.452 |  |
| 2010 | 1873 | 47.0 | [44.7–49.2] | 0.96 | [0.84–1.09] | 0.735 |  |
| 2011 | 1914 | 54.5 | [52.3–56.8] | 1.34 | [1.18–1.52] | 0.000 | *** |
| 2012 | 1894 | 60.4 | [58.2–62.6] | 1.28 | [1.12–1.46] | 0.001 | ** |
| 2013 | 1865 | 52.8 | [50.5–55] | 0.73 | [0.64–0.83] | 0.000 | *** |
| 2014 | 1955 | 51.8 | [49.6–54] | 0.97 | [0.85–1.1] | 0.803 |  |
| 2015 | 1863 | 51.7 | [49.5–54] | 0.99 | [0.87–1.12] | 0.891 |  |
| 2016 | 1862 | 53.3 | [51.1–55.6] | 1.06 | [0.93–1.21] | 0.648 |  |
| 2017 | 1834 | 53.2 | [50.9–55.5] | 0.99 | [0.87–1.12] | 0.891 |  |
| 2018 | 1907 | 56.4 | [54.1–58.6] | 1.11 | [0.98–1.27] | 0.247 |  |
| 2019 | 1895 | 56.1 | [53.9–58.4] | 0.97 | [0.85–1.1] | 0.803 |  |
| 2020 | 1802 | 53.1 | [50.7–55.3] | 0.88 | [0.78–1.01] | 0.171 |  |
| 2021 | 1836 | 53.1 | [50.8–55.4] | 1 | [0.87–1.14] | 0.966 |  |
| 2022 | 1906 | 53.8 | [51.6–56.1] | 1.02 | [0.9–1.17] | 0.850 |  |
| 2023 | 1821 | 55.4 | [53.1–57.7] | 1.05 | [0.93–1.2] | 0.683 |  |
| 2024 | 1613 | 53.9 | [51.4–56.3] | 0.94 | [0.82–1.08] | 0.648 |  |

### Table S40: Annual percentage of *Escherichia coli* resistance to Ceftazidime from 2005 to 2024.

|  | Tested | Resistance | | Logistic model | |  | |
| --- | --- | --- | --- | --- | --- | --- | --- |
| Year | N | % | [95% CI] | OR | [95% CI] | P-value |  |
| 2005 | 947 | 1.0 | [0.5–1.8] | – | – | – |  |
| 2006 | 1464 | 0.5 | [0.2–1] | 0.52 | [0.19–1.38] | 0.510 |  |
| 2007 | 1665 | 0.4 | [0.2–0.8] | 0.7 | [0.24–2.06] | 0.701 |  |
| 2008 | 1663 | 1.0 | [0.6–1.6] | 2.89 | [1.15–7.27] | 0.135 |  |
| 2009 | 1863 | 0.6 | [0.4–1.1] | 0.64 | [0.31–1.33] | 0.516 |  |
| 2010 | 1873 | 1.3 | [0.9–2] | 2.05 | [1.04–4.07] | 0.148 |  |
| 2011 | 1913 | 3.1 | [2.4–4] | 2.25 | [1.41–3.59] | 0.013 | * |
| 2012 | 1894 | 3.3 | [2.6–4.2] | 1.08 | [0.76–1.55] | 0.823 |  |
| 2013 | 1865 | 2.7 | [2–3.5] | 0.8 | [0.55–1.16] | 0.516 |  |
| 2014 | 1956 | 3.9 | [3.1–4.8] | 1.5 | [1.04–2.15] | 0.135 |  |
| 2015 | 1862 | 3.3 | [2.6–4.2] | 0.79 | [0.56–1.11] | 0.510 |  |
| 2016 | 1862 | 3.1 | [2.4–3.9] | 0.94 | [0.65–1.35] | 0.823 |  |
| 2017 | 1834 | 3.1 | [2.4–4] | 0.97 | [0.67–1.4] | 0.858 |  |
| 2018 | 1902 | 3.0 | [2.4–3.9] | 0.93 | [0.64–1.34] | 0.823 |  |
| 2019 | 1892 | 3.1 | [2.4–4] | 0.96 | [0.67–1.38] | 0.858 |  |
| 2020 | 1792 | 2.7 | [2–3.5] | 0.87 | [0.59–1.28] | 0.696 |  |
| 2021 | 1789 | 2.3 | [1.7–3.1] | 0.83 | [0.55–1.26] | 0.664 |  |
| 2022 | 1886 | 2.8 | [2.2–3.7] | 1.22 | [0.81–1.84] | 0.641 |  |
| 2023 | 1803 | 3.4 | [2.6–4.3] | 1.17 | [0.8–1.69] | 0.664 |  |
| 2024 | 1610 | 4.9 | [4–6.1] | 1.5 | [1.07–2.1] | 0.135 |  |

### Table S41: Annual percentage of *Escherichia coli* resistance to Ciprofloxacin from 2005 to 2024.

|  | Tested | Resistance | | Logistic model | |  | |
| --- | --- | --- | --- | --- | --- | --- | --- |
| Year | N | % | [95% CI] | OR | [95% CI] | P-value |  |
| 2005 | 948 | 7.8 | [6.3–9.7] | – | – | – |  |
| 2006 | 1464 | 5.5 | [4.4–6.7] | 0.69 | [0.49–0.95] | 0.190 |  |
| 2007 | 1665 | 6.4 | [5.3–7.6] | 1.09 | [0.81–1.48] | 0.773 |  |
| 2008 | 1663 | 5.9 | [4.9–7.1] | 0.94 | [0.7–1.24] | 0.773 |  |
| 2009 | 1864 | 6.3 | [5.3–7.5] | 1.12 | [0.84–1.47] | 0.773 |  |
| 2010 | 1873 | 5.2 | [4.3–6.3] | 0.79 | [0.6–1.04] | 0.370 |  |
| 2011 | 1914 | 7.3 | [6.2–8.5] | 1.35 | [1.03–1.76] | 0.190 |  |
| 2012 | 1894 | 10.1 | [8.8–11.5] | 1.49 | [1.18–1.87] | 0.015 | * |
| 2013 | 1865 | 9.5 | [8.2–10.9] | 0.92 | [0.74–1.14] | 0.773 |  |
| 2014 | 1957 | 9.9 | [8.6–11.3] | 1.06 | [0.85–1.31] | 0.773 |  |
| 2015 | 1864 | 9.7 | [8.4–11.1] | 0.92 | [0.74–1.15] | 0.773 |  |
| 2016 | 1863 | 7.9 | [6.8–9.3] | 0.8 | [0.63–1.01] | 0.265 |  |
| 2017 | 1837 | 7.9 | [6.7–9.2] | 0.94 | [0.74–1.19] | 0.773 |  |
| 2018 | 1197 | 8.5 | [7.1–10.2] | 1.07 | [0.82–1.4] | 0.773 |  |
| 2019 | 945 | 9.0 | [7.3–11] | 0.95 | [0.7–1.29] | 0.773 |  |
| 2020 | 1670 | 9.3 | [8–10.8] | 1.05 | [0.79–1.39] | 0.773 |  |
| 2021 | 1666 | 10.1 | [8.7–11.6] | 1.05 | [0.83–1.32] | 0.773 |  |
| 2022 | 1798 | 9.5 | [8.2–10.9] | 0.92 | [0.74–1.16] | 0.773 |  |
| 2023 | 1702 | 9.6 | [8.3–11.1] | 0.97 | [0.77–1.21] | 0.773 |  |
| 2024 | 1363 | 9.9 | [8.4–11.6] | 1.04 | [0.82–1.32] | 0.773 |  |

### Table S42: Annual percentage of *Escherichia coli* resistance to Cefotaxime from 2005 to 2024.

|  | Tested | Resistance | | Logistic model | |  | |
| --- | --- | --- | --- | --- | --- | --- | --- |
| Year | N | % | [95% CI] | OR | [95% CI] | P-value |  |
| 2005 | 1367 | 0.8 | [0.4–1.4] | – | – | – |  |
| 2006 | 1474 | 1.1 | [0.7–1.8] | 1.43 | [0.67–3.06] | 0.617 |  |
| 2007 | 1663 | 0.8 | [0.5–1.3] | 0.67 | [0.32–1.38] | 0.585 |  |
| 2008 | 1663 | 1.2 | [0.8–1.9] | 1.56 | [0.78–3.11] | 0.567 |  |
| 2009 | 1864 | 1.3 | [0.9–1.9] | 1.08 | [0.6–1.95] | 0.885 |  |
| 2010 | 1872 | 2.0 | [1.5–2.8] | 1.57 | [0.94–2.61] | 0.523 |  |
| 2011 | 1913 | 4.2 | [3.4–5.2] | 2.03 | [1.38–2.99] | 0.003 | ** |
| 2012 | 1894 | 4.4 | [3.5–5.4] | 1.07 | [0.78–1.46] | 0.831 |  |
| 2013 | 1865 | 3.6 | [2.8–4.5] | 0.8 | [0.58–1.11] | 0.567 |  |
| 2014 | 1956 | 6.3 | [5.3–7.5] | 1.86 | [1.38–2.52] | 0.001 | *** |
| 2015 | 1861 | 6.2 | [5.2–7.4] | 0.94 | [0.73–1.22] | 0.831 |  |
| 2016 | 1863 | 6.3 | [5.3–7.5] | 1.01 | [0.77–1.31] | 0.988 |  |
| 2017 | 1836 | 6.5 | [5.5–7.8] | 1 | [0.77–1.3] | 0.988 |  |
| 2018 | 1907 | 5.9 | [5–7.1] | 0.86 | [0.66–1.11] | 0.585 |  |
| 2019 | 1896 | 6.5 | [5.5–7.7] | 1.06 | [0.82–1.38] | 0.831 |  |
| 2020 | 1807 | 5.4 | [4.4–6.5] | 0.82 | [0.62–1.08] | 0.567 |  |
| 2021 | 1841 | 4.8 | [3.9–5.9] | 0.87 | [0.65–1.16] | 0.617 |  |
| 2022 | 1906 | 5.8 | [4.9–7] | 1.22 | [0.92–1.62] | 0.567 |  |
| 2023 | 1829 | 5.7 | [4.7–6.8] | 0.94 | [0.71–1.24] | 0.831 |  |
| 2024 | 1627 | 6.0 | [4.9–7.2] | 1.06 | [0.8–1.4] | 0.831 |  |

### Table S43: Annual percentage of *Escherichia coli* resistance to Gentamicin from 2005 to 2024.

|  | Tested | Resistance | | Logistic model | |  | |
| --- | --- | --- | --- | --- | --- | --- | --- |
| Year | N | % | [95% CI] | OR | [95% CI] | P-value |  |
| 2005 | 1371 | 4.3 | [3.4–5.5] | – | – | – |  |
| 2006 | 1473 | 2.8 | [2.1–3.8] | 0.66 | [0.44–0.99] | 0.280 |  |
| 2007 | 1665 | 3.7 | [2.9–4.7] | 1.21 | [0.81–1.82] | 0.616 |  |
| 2008 | 1663 | 3.8 | [3–4.9] | 1.1 | [0.77–1.58] | 0.843 |  |
| 2009 | 1863 | 3.6 | [2.8–4.5] | 0.94 | [0.66–1.34] | 0.843 |  |
| 2010 | 1873 | 3.9 | [3.1–4.9] | 1.06 | [0.76–1.49] | 0.843 |  |
| 2011 | 1914 | 4.9 | [4–5.9] | 1.22 | [0.89–1.67] | 0.616 |  |
| 2012 | 1894 | 5.6 | [4.7–6.8] | 1.18 | [0.89–1.58] | 0.616 |  |
| 2013 | 1865 | 6.5 | [5.5–7.7] | 1.15 | [0.88–1.51] | 0.616 |  |
| 2014 | 1957 | 4.8 | [3.9–5.8] | 0.72 | [0.55–0.96] | 0.214 |  |
| 2015 | 1864 | 5.5 | [4.6–6.7] | 1.14 | [0.85–1.52] | 0.616 |  |
| 2016 | 1863 | 5.7 | [4.8–6.9] | 1.05 | [0.79–1.38] | 0.843 |  |
| 2017 | 1838 | 5.2 | [4.3–6.3] | 0.88 | [0.66–1.17] | 0.616 |  |
| 2018 | 1908 | 6.2 | [5.2–7.4] | 1.16 | [0.88–1.53] | 0.616 |  |
| 2019 | 1882 | 6.4 | [5.4–7.6] | 0.99 | [0.76–1.28] | 0.928 |  |
| 2020 | 1785 | 6.0 | [5–7.2] | 0.94 | [0.72–1.23] | 0.843 |  |
| 2021 | 1785 | 5.2 | [4.3–6.3] | 0.84 | [0.63–1.13] | 0.616 |  |
| 2022 | 1891 | 5.2 | [4.3–6.3] | 0.99 | [0.74–1.32] | 0.928 |  |
| 2023 | 1809 | 7.8 | [6.6–9.1] | 1.51 | [1.16–1.97] | 0.048 | * |
| 2024 | 1446 | 7.0 | [5.8–8.4] | 0.89 | [0.68–1.16] | 0.616 |  |

### Table S44: Annual percentage of *Escherichia coli* resistance to Imipenem from 2005 to 2024.

|  | Tested | Resistance | | Logistic model | |  | |
| --- | --- | --- | --- | --- | --- | --- | --- |
| Year | N | % | [95% CI] | OR | [95% CI] | P-value |  |
| 2005 | 576 | 0.2 | [0–1] | – | – | – |  |
| 2006 | 648 | 0.0 | [0–0.6] | – | – | – |  |
| 2007 | 770 | 0.0 | [0–0.5] | – | – | – |  |
| 2008 | 739 | 0.0 | [0–0.5] | – | – | – |  |
| 2009 | 921 | 0.0 | [0–0.4] | – | – | – |  |
| 2010 | 852 | 0.0 | [0–0.4] | – | – | – |  |
| 2011 | 1759 | 0.0 | [0–0.2] | – | – | – |  |
| 2012 | 1894 | 0.0 | [0–0.2] | – | – | – |  |
| 2013 | 1862 | 0.1 | [0–0.3] | – | – | – |  |
| 2014 | 1957 | 0.0 | [0–0.2] | – | – | – |  |
| 2015 | 1497 | 0.0 | [0–0.3] | – | – | – |  |
| 2016 | 854 | 0.1 | [0–0.7] | – | – | – |  |
| 2017 | 747 | 0.0 | [0–0.5] | – | – | – |  |
| 2018 | 719 | 0.0 | [0–0.5] | – | – | – |  |
| 2019 | 994 | 0.0 | [0–0.4] | – | – | – |  |
| 2020 | 1792 | 0.0 | [0–0.2] | – | – | – |  |
| 2021 | 1801 | 0.0 | [0–0.2] | – | – | – |  |
| 2022 | 1885 | 0.0 | [0–0.2] | – | – | – |  |
| 2023 | 1425 | 0.0 | [0–0.3] | – | – | – |  |
| 2024 | 1106 | 0.0 | [0–0.3] | – | – | – |  |

### Table S45: Annual percentage of *Escherichia coli* resistance to Cotrimoxazole from 2005 to 2024.

|  | Tested | Resistance | | Logistic model | |  | |
| --- | --- | --- | --- | --- | --- | --- | --- |
| Year | N | % | [95% CI] | OR | [95% CI] | P-value |  |
| 2005 | 1371 | 19.0 | [17–21.1] | – | – | – |  |
| 2006 | 1472 | 17.8 | [15.9–19.8] | 0.95 | [0.78–1.16] | 0.849 |  |
| 2007 | 1665 | 19.3 | [17.5–21.3] | 1.08 | [0.89–1.3] | 0.744 |  |
| 2008 | 1663 | 20.7 | [18.9–22.8] | 1.12 | [0.95–1.34] | 0.698 |  |
| 2009 | 1862 | 19.5 | [17.8–21.4] | 0.91 | [0.77–1.08] | 0.698 |  |
| 2010 | 1872 | 20.2 | [18.4–22.1] | 1.04 | [0.89–1.22] | 0.849 |  |
| 2011 | 1913 | 20.5 | [18.7–22.4] | 1.01 | [0.86–1.18] | 0.986 |  |
| 2012 | 1894 | 23.9 | [22.1–25.9] | 1.22 | [1.05–1.43] | 0.094 | . |
| 2013 | 1865 | 20.5 | [18.7–22.4] | 0.82 | [0.7–0.95] | 0.094 | . |
| 2014 | 1956 | 21.7 | [20–23.6] | 1.08 | [0.93–1.27] | 0.698 |  |
| 2015 | 1863 | 19.0 | [17.3–20.8] | 0.84 | [0.71–0.98] | 0.169 |  |
| 2016 | 1858 | 18.9 | [17.2–20.7] | 0.99 | [0.84–1.17] | 0.986 |  |
| 2017 | 1833 | 21.0 | [19.2–22.9] | 1.13 | [0.96–1.33] | 0.642 |  |
| 2018 | 1903 | 22.5 | [20.7–24.4] | 1.08 | [0.92–1.26] | 0.698 |  |
| 2019 | 1895 | 22.8 | [21–24.7] | 1 | [0.86–1.17] | 0.993 |  |
| 2020 | 1801 | 22.5 | [20.6–24.5] | 0.99 | [0.85–1.15] | 0.986 |  |
| 2021 | 1823 | 21.3 | [19.5–23.3] | 0.93 | [0.79–1.09] | 0.698 |  |
| 2022 | 1905 | 21.7 | [19.9–23.6] | 1.02 | [0.87–1.19] | 0.986 |  |
| 2023 | 1824 | 23.5 | [21.6–25.5] | 1.09 | [0.94–1.28] | 0.698 |  |
| 2024 | 1624 | 22.7 | [20.7–24.8] | 0.96 | [0.82–1.12] | 0.849 |  |

### Table S46: Annual percentage of *Escherichia coli* resistance to Piperacillin/Tazobactam from 2005 to 2024.

|  | Tested | Resistance | | Logistic model | |  | |
| --- | --- | --- | --- | --- | --- | --- | --- |
| Year | N | % | [95% CI] | OR | [95% CI] | P-value |  |
| 2005 | 575 | 0.7 | [0.3–1.8] | – | – | – |  |
| 2006 | 677 | 0.6 | [0.2–1.5] | 0.88 | [0.22–3.52] | 0.902 |  |
| 2007 | 770 | 0.1 | [0–0.7] | 0.2 | [0.02–1.78] | 0.352 |  |
| 2008 | 740 | 0.7 | [0.3–1.6] | 5.13 | [0.6–43.65] | 0.352 |  |
| 2009 | 922 | 3.4 | [2.4–4.7] | 5.11 | [1.98–13.19] | 0.004 | ** |
| 2010 | 853 | 7.6 | [6–9.6] | 2.33 | [1.5–3.61] | 0.002 | ** |
| 2011 | 1159 | 4.4 | [3.4–5.7] | 0.52 | [0.36–0.76] | 0.004 | ** |
| 2012 | 1277 | 4.0 | [3.1–5.2] | 0.91 | [0.61–1.35] | 0.855 |  |
| 2013 | 1860 | 7.1 | [6–8.4] | 1.86 | [1.33–2.59] | 0.002 | ** |
| 2014 | 1947 | 9.6 | [8.4–11] | 1.4 | [1.11–1.77] | 0.017 | * |
| 2015 | 1855 | 10.0 | [8.7–11.5] | 1.03 | [0.83–1.28] | 0.884 |  |
| 2016 | 1855 | 11.2 | [9.9–12.7] | 1.13 | [0.92–1.4] | 0.469 |  |
| 2017 | 1817 | 11.7 | [10.3–13.2] | 1.03 | [0.84–1.26] | 0.884 |  |
| 2018 | 1886 | 12.0 | [10.6–13.6] | 1.01 | [0.83–1.23] | 0.932 |  |
| 2019 | 1858 | 13.2 | [11.7–14.8] | 1.08 | [0.89–1.31] | 0.671 |  |
| 2020 | 1775 | 10.9 | [9.6–12.5] | 0.82 | [0.67–1] | 0.151 |  |
| 2021 | 1820 | 10.7 | [9.3–12.2] | 0.96 | [0.78–1.19] | 0.884 |  |
| 2022 | 1891 | 9.7 | [8.5–11.1] | 0.9 | [0.73–1.11] | 0.558 |  |
| 2023 | 1791 | 10.7 | [9.3–12.2] | 1.09 | [0.88–1.35] | 0.671 |  |
| 2024 | 1606 | 11.9 | [10.4–13.6] | 1.13 | [0.92–1.4] | 0.469 |  |

### Figure S4: Annual percentage of *Enterococcus cloacae* complex resistance from 2005 to 2024


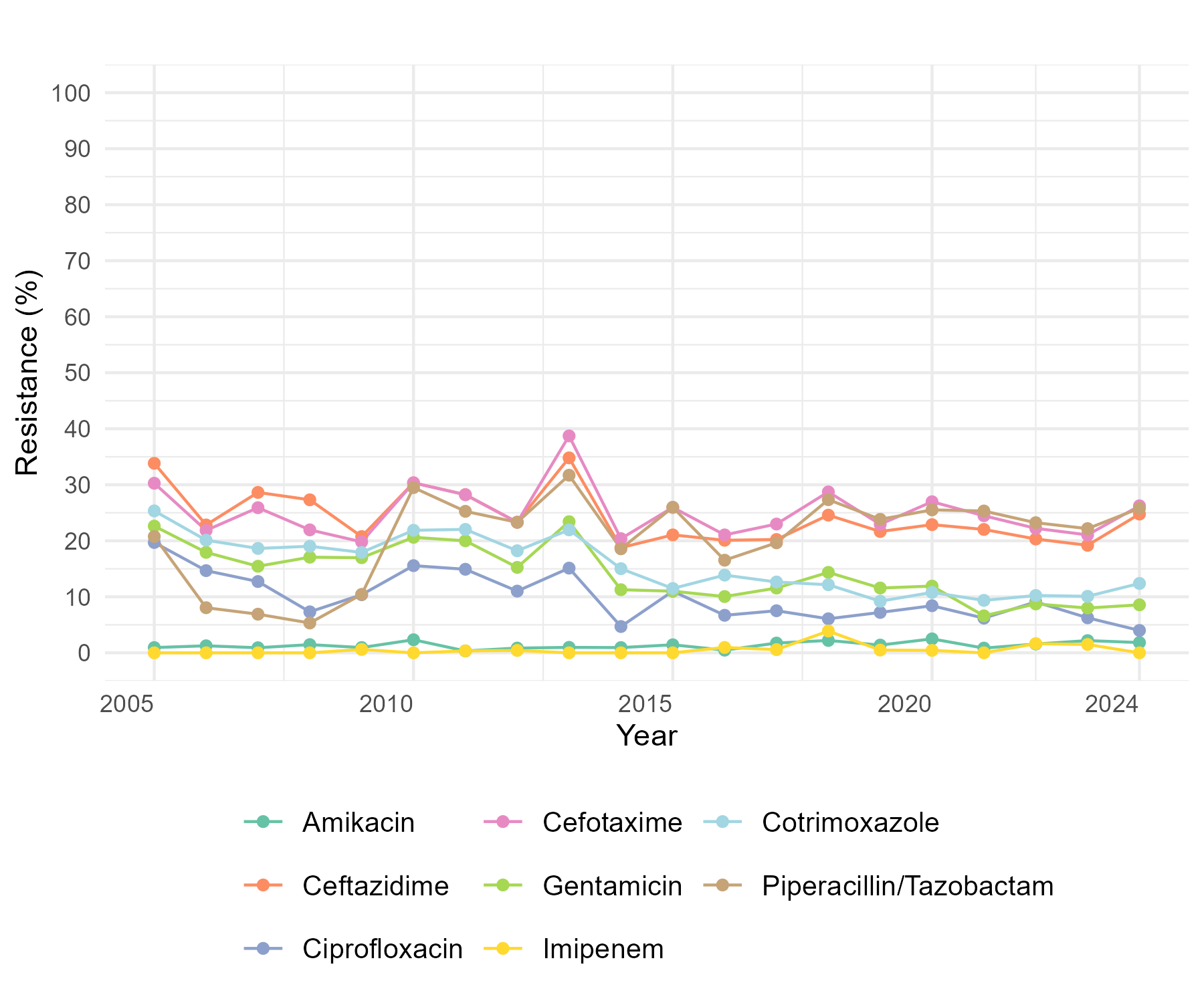


### Table S47: Logistic regression analysis of the effect of continuous time on the antibiotic resistance of *Enterococcus cloacae* complex from 2005 to 2024.

| Antibiotic | β | OR | [95%CI] | P-value |  |
| --- | --- | --- | --- | --- | --- |
| Amikacin | 0.033 | 1.03 | [0.99–1.08] | 0.152 |  |
| Ceftazidime | -0.026 | 0.97 | [0.96–0.99] | 0.000 | *** |
| Ciprofloxacin | -0.065 | 0.94 | [0.92–0.95] | 0.000 | *** |
| Cefotaxime | -0.008 | 0.99 | [0.98–1] | 0.174 |  |
| Gentamicin | -0.065 | 0.94 | [0.92–0.95] | 0.000 | *** |
| Imipenem | 0.126 | 1.13 | [1.05–1.23] | 0.003 | ** |
| Cotrimoxazole | -0.064 | 0.94 | [0.92–0.95] | 0.000 | *** |
| Piperacillin/Tazobactam | 0.036 | 1.04 | [1.02–1.05] | 0.000 | *** |

### Table S48: Annual percentage of *Enterococcus cloacae* complex resistance to Amikacin from 2005 to 2024.

|  | Tested | Resistance | | Logistic model | |  | |
| --- | --- | --- | --- | --- | --- | --- | --- |
| Year | N | % | [95% CI] | OR | [95% CI] | P-value |  |
| 2005 | 213 | 0.9 | [0.3–3.4] | – | – | – |  |
| 2006 | 159 | 1.3 | [0.3–4.5] | 1.34 | [0.19–9.62] | 0.917 |  |
| 2007 | 220 | 0.9 | [0.2–3.3] | 0.74 | [0.1–5.33] | 0.917 |  |
| 2008 | 205 | 1.5 | [0.5–4.2] | 1.53 | [0.25–9.25] | 0.917 |  |
| 2009 | 212 | 0.9 | [0.3–3.4] | 0.69 | [0.11–4.15] | 0.917 |  |
| 2010 | 257 | 2.3 | [1.1–5] | 2.33 | [0.47–11.66] | 0.917 |  |
| 2011 | 294 | 0.3 | [0.1–1.9] | 0.14 | [0.02–1.2] | 0.917 |  |
| 2012 | 236 | 0.8 | [0.2–3] | 2.66 | [0.24–29.38] | 0.917 |  |
| 2013 | 205 | 1.0 | [0.3–3.5] | 1.13 | [0.16–8.05] | 0.955 |  |
| 2014 | 213 | 0.9 | [0.3–3.4] | 0.95 | [0.13–6.75] | 0.956 |  |
| 2015 | 209 | 1.4 | [0.5–4.1] | 1.54 | [0.26–9.31] | 0.917 |  |
| 2016 | 209 | 0.5 | [0.1–2.7] | 0.32 | [0.03–3.04] | 0.917 |  |
| 2017 | 172 | 1.7 | [0.6–5] | 4.02 | [0.42–38.8] | 0.917 |  |
| 2018 | 181 | 2.2 | [0.9–5.5] | 1.19 | [0.26–5.39] | 0.917 |  |
| 2019 | 217 | 1.4 | [0.5–4] | 0.59 | [0.13–2.66] | 0.917 |  |
| 2020 | 241 | 2.5 | [1.1–5.3] | 1.9 | [0.47–7.69] | 0.917 |  |
| 2021 | 237 | 0.8 | [0.2–3] | 0.35 | [0.07–1.74] | 0.917 |  |
| 2022 | 255 | 1.6 | [0.6–4] | 1.78 | [0.32–9.79] | 0.917 |  |
| 2023 | 229 | 2.2 | [0.9–5] | 1.38 | [0.37–5.19] | 0.917 |  |
| 2024 | 218 | 1.8 | [0.7–4.6] | 0.83 | [0.22–3.13] | 0.917 |  |

### Table S49: Annual percentage of *Enterococcus cloacae* complex resistance to Ceftazidime from 2005 to 2024.

|  | Tested | Resistance | | Logistic model | |  | |
| --- | --- | --- | --- | --- | --- | --- | --- |
| Year | N | % | [95% CI] | OR | [95% CI] | P-value |  |
| 2005 | 198 | 33.8 | [27.6–40.7] | – | – | – |  |
| 2006 | 184 | 22.8 | [17.4–29.4] | 0.59 | [0.37–0.93] | 0.155 |  |
| 2007 | 220 | 28.6 | [23.1–34.9] | 1.35 | [0.85–2.14] | 0.566 |  |
| 2008 | 205 | 27.3 | [21.7–33.8] | 0.91 | [0.59–1.4] | 0.808 |  |
| 2009 | 212 | 20.8 | [15.8–26.7] | 0.73 | [0.46–1.16] | 0.566 |  |
| 2010 | 257 | 30.4 | [25.1–36.2] | 1.58 | [1.02–2.44] | 0.190 |  |
| 2011 | 294 | 28.2 | [23.4–33.6] | 0.91 | [0.63–1.33] | 0.808 |  |
| 2012 | 236 | 23.3 | [18.4–29.1] | 0.81 | [0.54–1.21] | 0.707 |  |
| 2013 | 204 | 34.8 | [28.6–41.6] | 1.76 | [1.15–2.71] | 0.089 | . |
| 2014 | 213 | 18.8 | [14.1–24.6] | 0.42 | [0.26–0.66] | 0.004 | ** |
| 2015 | 209 | 21.1 | [16.1–27.1] | 1.16 | [0.71–1.88] | 0.808 |  |
| 2016 | 209 | 20.1 | [15.2–26] | 0.91 | [0.56–1.48] | 0.808 |  |
| 2017 | 173 | 20.2 | [14.9–26.8] | 1.07 | [0.64–1.79] | 0.835 |  |
| 2018 | 179 | 24.6 | [18.9–31.4] | 1.22 | [0.73–2.04] | 0.808 |  |
| 2019 | 217 | 21.7 | [16.7–27.6] | 0.82 | [0.51–1.32] | 0.808 |  |
| 2020 | 236 | 22.9 | [18–28.6] | 1.11 | [0.71–1.75] | 0.808 |  |
| 2021 | 227 | 22.0 | [17.1–27.9] | 1 | [0.64–1.56] | 0.999 |  |
| 2022 | 251 | 20.3 | [15.8–25.7] | 0.86 | [0.55–1.35] | 0.808 |  |
| 2023 | 224 | 19.2 | [14.6–24.9] | 0.92 | [0.58–1.46] | 0.808 |  |
| 2024 | 218 | 24.8 | [19.5–30.9] | 1.37 | [0.86–2.18] | 0.566 |  |

### Table S50: Annual percentage of *Enterococcus cloacae* complex resistance to Ciprofloxacin from 2005 to 2024.

|  | Tested | Resistance | | Logistic model | |  | |
| --- | --- | --- | --- | --- | --- | --- | --- |
| Year | N | % | [95% CI] | OR | [95% CI] | P-value |  |
| 2005 | 198 | 19.7 | [14.8–25.8] | – | – | – |  |
| 2006 | 184 | 14.7 | [10.3–20.5] | 0.69 | [0.4–1.2] | 0.514 |  |
| 2007 | 220 | 12.7 | [9–17.8] | 0.85 | [0.47–1.51] | 0.642 |  |
| 2008 | 205 | 7.3 | [4.5–11.7] | 0.48 | [0.25–0.92] | 0.177 |  |
| 2009 | 212 | 10.4 | [7–15.2] | 1.7 | [0.85–3.37] | 0.502 |  |
| 2010 | 257 | 15.6 | [11.6–20.5] | 1.46 | [0.83–2.55] | 0.514 |  |
| 2011 | 295 | 14.9 | [11.3–19.4] | 0.98 | [0.61–1.56] | 0.919 |  |
| 2012 | 236 | 11.0 | [7.6–15.7] | 0.76 | [0.45–1.29] | 0.536 |  |
| 2013 | 205 | 15.1 | [10.9–20.7] | 1.42 | [0.8–2.5] | 0.536 |  |
| 2014 | 213 | 4.7 | [2.6–8.4] | 0.25 | [0.12–0.52] | 0.004 | ** |
| 2015 | 209 | 11.0 | [7.4–16] | 2.68 | [1.25–5.76] | 0.110 |  |
| 2016 | 209 | 6.7 | [4–10.9] | 0.52 | [0.26–1.04] | 0.311 |  |
| 2017 | 173 | 7.5 | [4.4–12.4] | 1.3 | [0.6–2.84] | 0.642 |  |
| 2018 | 181 | 6.1 | [3.4–10.6] | 0.71 | [0.31–1.63] | 0.616 |  |
| 2019 | 208 | 7.2 | [4.4–11.6] | 1.14 | [0.51–2.53] | 0.793 |  |
| 2020 | 226 | 8.4 | [5.4–12.8] | 1.25 | [0.62–2.51] | 0.642 |  |
| 2021 | 210 | 6.2 | [3.7–10.3] | 0.8 | [0.39–1.67] | 0.642 |  |
| 2022 | 242 | 9.1 | [6.1–13.4] | 1.35 | [0.66–2.74] | 0.616 |  |
| 2023 | 208 | 6.2 | [3.7–10.4] | 0.66 | [0.33–1.35] | 0.536 |  |
| 2024 | 200 | 4.0 | [2–7.7] | 0.61 | [0.25–1.5] | 0.536 |  |

### Table S51: Annual percentage of *Enterococcus cloacae* complex resistance to Cefotaxime from 2005 to 2024.

|  | Tested | Resistance | | Logistic model | |  | |
| --- | --- | --- | --- | --- | --- | --- | --- |
| Year | N | % | [95% CI] | OR | [95% CI] | P-value |  |
| 2005 | 218 | 30.3 | [24.6–36.7] | – | – | – |  |
| 2006 | 183 | 21.9 | [16.5–28.4] | 0.65 | [0.41–1.04] | 0.338 |  |
| 2007 | 220 | 25.9 | [20.6–32.1] | 1.24 | [0.77–1.99] | 0.548 |  |
| 2008 | 205 | 22.0 | [16.8–28.1] | 0.78 | [0.49–1.23] | 0.488 |  |
| 2009 | 212 | 19.8 | [15–25.7] | 0.93 | [0.57–1.5] | 0.761 |  |
| 2010 | 257 | 30.4 | [25.1–36.2] | 1.68 | [1.08–2.61] | 0.131 |  |
| 2011 | 294 | 28.2 | [23.4–33.6] | 0.91 | [0.63–1.33] | 0.739 |  |
| 2012 | 236 | 23.3 | [18.4–29.1] | 0.8 | [0.54–1.2] | 0.488 |  |
| 2013 | 204 | 38.7 | [32.3–45.6] | 2.11 | [1.38–3.22] | 0.005 | ** |
| 2014 | 211 | 20.4 | [15.5–26.3] | 0.39 | [0.25–0.61] | 0.001 | *** |
| 2015 | 208 | 26.0 | [20.5–32.3] | 1.38 | [0.87–2.2] | 0.488 |  |
| 2016 | 209 | 21.1 | [16.1–27.1] | 0.73 | [0.46–1.16] | 0.488 |  |
| 2017 | 174 | 23.0 | [17.4–29.8] | 1.19 | [0.73–1.95] | 0.619 |  |
| 2018 | 181 | 28.7 | [22.6–35.7] | 1.29 | [0.79–2.1] | 0.488 |  |
| 2019 | 218 | 22.9 | [17.9–29] | 0.71 | [0.45–1.12] | 0.488 |  |
| 2020 | 241 | 27.0 | [21.8–32.9] | 1.28 | [0.83–1.98] | 0.488 |  |
| 2021 | 237 | 24.5 | [19.4–30.3] | 0.91 | [0.6–1.39] | 0.739 |  |
| 2022 | 252 | 22.2 | [17.5–27.8] | 0.85 | [0.56–1.31] | 0.619 |  |
| 2023 | 228 | 21.1 | [16.3–26.8] | 0.92 | [0.59–1.43] | 0.739 |  |
| 2024 | 221 | 26.2 | [20.9–32.4] | 1.33 | [0.85–2.07] | 0.488 |  |

### Table S52: Annual percentage of *Enterococcus cloacae* complex resistance to Gentamicin from 2005 to 2024.

|  | Tested | Resistance | | Logistic model | |  | |
| --- | --- | --- | --- | --- | --- | --- | --- |
| Year | N | % | [95% CI] | OR | [95% CI] | P-value |  |
| 2005 | 221 | 22.6 | [17.6–28.6] | – | – | – |  |
| 2006 | 184 | 17.9 | [13.1–24.1] | 0.74 | [0.44–1.26] | 0.930 |  |
| 2007 | 220 | 15.5 | [11.3–20.8] | 0.84 | [0.48–1.46] | 0.952 |  |
| 2008 | 205 | 17.1 | [12.5–22.8] | 1.04 | [0.6–1.79] | 0.982 |  |
| 2009 | 212 | 17.0 | [12.5–22.6] | 1.12 | [0.65–1.92] | 0.952 |  |
| 2010 | 257 | 20.6 | [16.1–26] | 1.15 | [0.7–1.89] | 0.952 |  |
| 2011 | 295 | 20.0 | [15.8–24.9] | 0.98 | [0.63–1.53] | 0.982 |  |
| 2012 | 236 | 15.3 | [11.2–20.4] | 0.77 | [0.48–1.25] | 0.930 |  |
| 2013 | 205 | 23.4 | [18.1–29.7] | 1.72 | [1.03–2.87] | 0.356 |  |
| 2014 | 213 | 11.3 | [7.7–16.2] | 0.37 | [0.21–0.66] | 0.012 | * |
| 2015 | 209 | 11.0 | [7.4–16] | 0.99 | [0.53–1.87] | 0.982 |  |
| 2016 | 209 | 10.0 | [6.7–14.9] | 0.84 | [0.44–1.61] | 0.952 |  |
| 2017 | 173 | 11.6 | [7.6–17.2] | 1.33 | [0.68–2.62] | 0.952 |  |
| 2018 | 181 | 14.4 | [10–20.2] | 1.18 | [0.61–2.27] | 0.952 |  |
| 2019 | 216 | 11.6 | [8–16.5] | 0.72 | [0.39–1.33] | 0.930 |  |
| 2020 | 235 | 11.9 | [8.4–16.7] | 1.09 | [0.6–1.99] | 0.981 |  |
| 2021 | 227 | 6.6 | [4–10.6] | 0.56 | [0.28–1.11] | 0.621 |  |
| 2022 | 252 | 8.7 | [5.8–12.9] | 1.24 | [0.61–2.52] | 0.952 |  |
| 2023 | 225 | 8.0 | [5.1–12.3] | 0.88 | [0.45–1.72] | 0.952 |  |
| 2024 | 210 | 8.6 | [5.5–13.1] | 1.08 | [0.53–2.19] | 0.982 |  |

### Table S53: Annual percentage of *Enterococcus cloacae* complex resistance to Imipenem from 2005 to 2024.

|  | Tested | Resistance | | Logistic model | |  | |
| --- | --- | --- | --- | --- | --- | --- | --- |
| Year | N | % | [95% CI] | OR | [95% CI] | P-value |  |
| 2005 | 183 | 0.0 | [0–2.1] | – | – | – |  |
| 2006 | 143 | 0.0 | [0–2.6] | – | – | – |  |
| 2007 | 174 | 0.0 | [0–2.2] | – | – | – |  |
| 2008 | 169 | 0.0 | [0–2.2] | – | – | – |  |
| 2009 | 165 | 0.6 | [0.1–3.4] | – | – | – |  |
| 2010 | 201 | 0.0 | [0–1.9] | – | – | – |  |
| 2011 | 289 | 0.3 | [0.1–1.9] | – | – | – |  |
| 2012 | 235 | 0.4 | [0.1–2.4] | 1.24 | [0.17–8.96] | 1.000 |  |
| 2013 | 203 | 0.0 | [0–1.9] | – | – | – |  |
| 2014 | 213 | 0.0 | [0–1.8] | – | – | – |  |
| 2015 | 207 | 0.0 | [0–1.8] | – | – | – |  |
| 2016 | 208 | 1.0 | [0.3–3.4] | – | – | – |  |
| 2017 | 174 | 0.6 | [0.1–3.2] | 0.59 | [0.11–3.27] | 1.000 |  |
| 2018 | 180 | 3.9 | [1.9–7.8] | 7.18 | [1.6–32.28] | 0.097 | . |
| 2019 | 204 | 0.5 | [0.1–2.7] | 0.12 | [0.03–0.54] | 0.097 | . |
| 2020 | 228 | 0.4 | [0.1–2.4] | 0.9 | [0.12–6.56] | 1.000 |  |
| 2021 | 222 | 0.0 | [0–1.7] | – | – | – |  |
| 2022 | 245 | 1.6 | [0.6–4.1] | – | – | – |  |
| 2023 | 201 | 1.5 | [0.5–4.3] | 0.93 | [0.32–2.74] | 1.000 |  |
| 2024 | 177 | 0.0 | [0–2.1] | – | – | – |  |

### Table S54: Annual percentage of *Enterococcus cloacae* complex resistance to Cotrimoxazole from 2005 to 2024.

|  | Tested | Resistance | | Logistic model | |  | |
| --- | --- | --- | --- | --- | --- | --- | --- |
| Year | N | % | [95% CI] | OR | [95% CI] | P-value |  |
| 2005 | 221 | 25.3 | [20.1–31.5] | – | – | – |  |
| 2006 | 184 | 20.1 | [15–26.5] | 0.74 | [0.45–1.23] | 0.983 |  |
| 2007 | 220 | 18.6 | [14–24.3] | 0.92 | [0.54–1.55] | 0.983 |  |
| 2008 | 205 | 19.0 | [14.2–24.9] | 0.95 | [0.57–1.58] | 0.983 |  |
| 2009 | 212 | 17.9 | [13.3–23.6] | 1.02 | [0.61–1.72] | 0.983 |  |
| 2010 | 256 | 21.9 | [17.2–27.3] | 1.17 | [0.72–1.9] | 0.983 |  |
| 2011 | 295 | 22.0 | [17.7–27.1] | 1.03 | [0.68–1.59] | 0.983 |  |
| 2012 | 236 | 18.2 | [13.8–23.6] | 0.84 | [0.54–1.33] | 0.983 |  |
| 2013 | 205 | 22.0 | [16.8–28.1] | 1.25 | [0.76–2.04] | 0.983 |  |
| 2014 | 213 | 15.0 | [10.8–20.4] | 0.59 | [0.35–1.01] | 0.983 |  |
| 2015 | 209 | 11.5 | [7.8–16.5] | 0.73 | [0.4–1.32] | 0.983 |  |
| 2016 | 209 | 13.9 | [9.8–19.2] | 1.18 | [0.65–2.16] | 0.983 |  |
| 2017 | 174 | 12.6 | [8.5–18.4] | 0.99 | [0.53–1.85] | 0.983 |  |
| 2018 | 181 | 12.2 | [8.2–17.7] | 0.87 | [0.45–1.69] | 0.983 |  |
| 2019 | 217 | 9.2 | [6–13.8] | 0.68 | [0.35–1.32] | 0.983 |  |
| 2020 | 241 | 10.8 | [7.5–15.3] | 1.26 | [0.67–2.38] | 0.983 |  |
| 2021 | 235 | 9.4 | [6.3–13.8] | 0.91 | [0.49–1.7] | 0.983 |  |
| 2022 | 254 | 10.2 | [7.1–14.6] | 1.03 | [0.55–1.91] | 0.983 |  |
| 2023 | 228 | 10.1 | [6.8–14.7] | 0.95 | [0.52–1.77] | 0.983 |  |
| 2024 | 218 | 12.4 | [8.7–17.4] | 1.26 | [0.68–2.34] | 0.983 |  |

### Table S55: Annual percentage of *Enterococcus cloacae* complex resistance to Piperacillin/Tazobactam from 2005 to 2024.

|  | Tested | Resistance | | Logistic model | |  | |
| --- | --- | --- | --- | --- | --- | --- | --- |
| Year | N | % | [95% CI] | OR | [95% CI] | P-value |  |
| 2005 | 183 | 20.8 | [15.5–27.2] | – | – | – |  |
| 2006 | 149 | 8.1 | [4.7–13.5] | 0.33 | [0.16–0.67] | 0.014 | * |
| 2007 | 174 | 6.9 | [4–11.7] | 0.77 | [0.33–1.83] | 0.739 |  |
| 2008 | 169 | 5.3 | [2.8–9.8] | 0.81 | [0.32–2.02] | 0.766 |  |
| 2009 | 163 | 10.4 | [6.6–16.1] | 2.2 | [0.94–5.15] | 0.207 |  |
| 2010 | 200 | 29.5 | [23.6–36.2] | 3.36 | [1.85–6.12] | 0.001 | ** |
| 2011 | 289 | 25.3 | [20.6–30.6] | 0.83 | [0.55–1.26] | 0.712 |  |
| 2012 | 236 | 23.3 | [18.4–29.1] | 0.95 | [0.63–1.43] | 0.862 |  |
| 2013 | 205 | 31.7 | [25.7–38.4] | 1.53 | [0.99–2.35] | 0.207 |  |
| 2014 | 210 | 18.6 | [13.9–24.4] | 0.48 | [0.3–0.76] | 0.014 | * |
| 2015 | 204 | 26.0 | [20.4–32.4] | 1.54 | [0.96–2.49] | 0.207 |  |
| 2016 | 127 | 16.5 | [11.1–24] | 0.54 | [0.3–0.95] | 0.160 |  |
| 2017 | 112 | 19.6 | [13.3–28] | 1.28 | [0.65–2.52] | 0.712 |  |
| 2018 | 139 | 27.3 | [20.6–35.3] | 1.58 | [0.86–2.9] | 0.343 |  |
| 2019 | 214 | 23.8 | [18.6–30] | 0.78 | [0.47–1.29] | 0.702 |  |
| 2020 | 239 | 25.5 | [20.4–31.4] | 1.13 | [0.73–1.75] | 0.739 |  |
| 2021 | 237 | 25.3 | [20.2–31.2] | 1.02 | [0.67–1.56] | 0.921 |  |
| 2022 | 254 | 23.2 | [18.5–28.8] | 0.86 | [0.56–1.31] | 0.712 |  |
| 2023 | 221 | 22.2 | [17.2–28.1] | 0.95 | [0.61–1.47] | 0.862 |  |
| 2024 | 217 | 25.8 | [20.4–32] | 1.2 | [0.76–1.88] | 0.712 |  |

### Figure S5: Annual percentage of *Klebsiella pneumoniae* resistance from 2005 to 2024


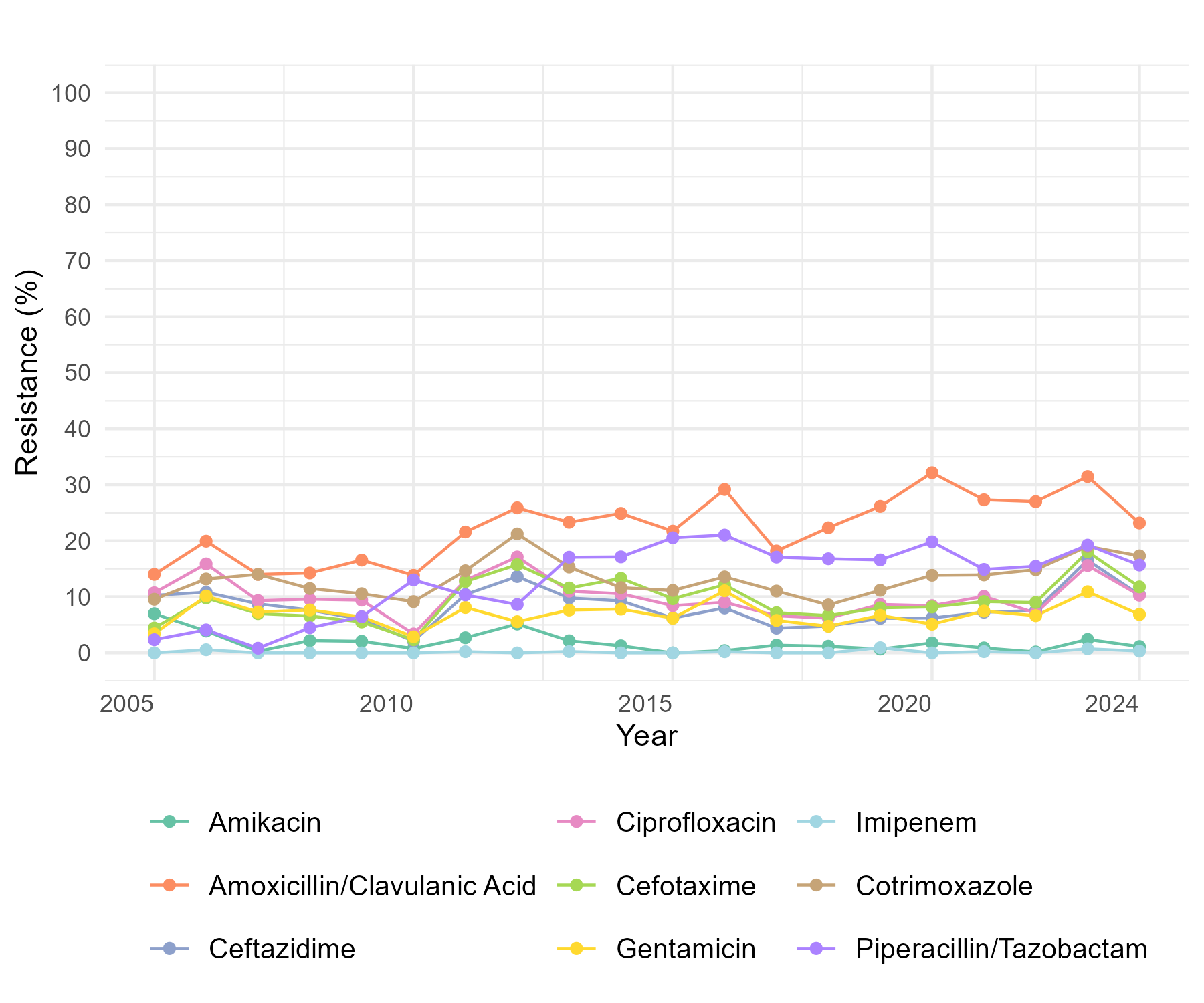


### Table S56: Logistic regression analysis of the effect of continuous time on the antibiotic resistance of *Klebsiella pneumoniae* from 2005 to 2024.

| Antibiotic | β | OR | [95%CI] | P-value |  |
| --- | --- | --- | --- | --- | --- |
| Amoxicillin/Clavulanic Acid | 0.037 | 1.04 | [1.03–1.05] | 0.000 | *** |
| Amikacin | -0.084 | 0.92 | [0.89–0.95] | 0.000 | *** |
| Ceftazidime | -0.006 | 0.99 | [0.98–1.01] | 0.398 |  |
| Ciprofloxacin | -0.022 | 0.98 | [0.97–0.99] | 0.001 | ** |
| Cefotaxime | 0.015 | 1.02 | [1–1.03] | 0.022 | * |
| Gentamicin | -0.002 | 1.00 | [0.98–1.01] | 0.762 |  |
| Imipenem | 0.093 | 1.10 | [0.97–1.24] | 0.133 |  |
| Cotrimoxazole | 0.009 | 1.01 | [1–1.02] | 0.104 |  |
| Piperacillin/Tazobactam | 0.058 | 1.06 | [1.05–1.07] | 0.000 | *** |

### Table S57: Annual percentage of *Klebsiella pneumoniae* resistance to Amoxicillin/Clavulanic Acid from 2005 to 2024.

|  | Tested | Resistance | | Logistic model | |  | |
| --- | --- | --- | --- | --- | --- | --- | --- |
| Year | N | % | [95% CI] | OR | [95% CI] | P-value |  |
| 2005 | 293 | 14.0 | [10.5–18.4] | – | – | – |  |
| 2006 | 296 | 19.9 | [15.8–24.9] | 1.49 | [0.96–2.31] | 0.204 |  |
| 2007 | 343 | 14.0 | [10.7–18.1] | 0.66 | [0.43–1] | 0.165 |  |
| 2008 | 365 | 14.2 | [11–18.2] | 1.01 | [0.66–1.54] | 0.975 |  |
| 2009 | 435 | 16.6 | [13.4–20.3] | 1.23 | [0.83–1.82] | 0.406 |  |
| 2010 | 383 | 13.8 | [10.7–17.7] | 0.82 | [0.56–1.21] | 0.406 |  |
| 2011 | 519 | 21.6 | [18.3–25.3] | 1.69 | [1.18–2.42] | 0.028 | * |
| 2012 | 483 | 25.9 | [22.2–30] | 1.24 | [0.92–1.66] | 0.280 |  |
| 2013 | 416 | 23.3 | [19.5–27.6] | 0.89 | [0.65–1.21] | 0.532 |  |
| 2014 | 474 | 24.9 | [21.2–29] | 1.07 | [0.78–1.46] | 0.746 |  |
| 2015 | 405 | 21.7 | [18–26] | 0.8 | [0.59–1.1] | 0.283 |  |
| 2016 | 487 | 29.2 | [25.3–33.3] | 1.51 | [1.11–2.06] | 0.042 | * |
| 2017 | 363 | 18.2 | [14.6–22.5] | 0.54 | [0.39–0.75] | 0.006 | ** |
| 2018 | 421 | 22.3 | [18.6–26.5] | 1.29 | [0.9–1.84] | 0.280 |  |
| 2019 | 440 | 26.1 | [22.3–30.4] | 1.18 | [0.86–1.61] | 0.406 |  |
| 2020 | 451 | 32.2 | [28–36.6] | 1.37 | [1.02–1.84] | 0.130 |  |
| 2021 | 443 | 27.3 | [23.4–31.6] | 0.78 | [0.58–1.04] | 0.220 |  |
| 2022 | 578 | 27.0 | [23.5–30.8] | 0.99 | [0.75–1.32] | 0.975 |  |
| 2023 | 499 | 31.5 | [27.5–35.7] | 1.21 | [0.93–1.58] | 0.280 |  |
| 2024 | 440 | 23.2 | [19.5–27.3] | 0.65 | [0.49–0.88] | 0.028 | * |

### Table S58: Annual percentage of *Klebsiella pneumoniae* resistance to Amikacin from 2005 to 2024.

|  | Tested | Resistance | | Logistic model | |  | |
| --- | --- | --- | --- | --- | --- | --- | --- |
| Year | N | % | [95% CI] | OR | [95% CI] | P-value |  |
| 2005 | 258 | 7.0 | [4.5–10.8] | – | – | – |  |
| 2006 | 231 | 3.9 | [2.1–7.2] | 0.46 | [0.2–1.06] | 0.183 |  |
| 2007 | 343 | 0.3 | [0.1–1.6] | 0.08 | [0.01–0.61] | 0.148 |  |
| 2008 | 366 | 2.2 | [1.1–4.3] | 7.7 | [0.96–62.06] | 0.181 |  |
| 2009 | 436 | 2.1 | [1.1–3.9] | 0.98 | [0.37–2.58] | 0.967 |  |
| 2010 | 384 | 0.8 | [0.3–2.3] | 0.38 | [0.1–1.41] | 0.241 |  |
| 2011 | 519 | 2.7 | [1.6–4.5] | 3.45 | [0.98–12.13] | 0.181 |  |
| 2012 | 485 | 5.2 | [3.5–7.5] | 1.92 | [0.98–3.75] | 0.181 |  |
| 2013 | 418 | 2.2 | [1.1–4] | 0.41 | [0.19–0.89] | 0.148 |  |
| 2014 | 474 | 1.3 | [0.6–2.7] | 0.57 | [0.2–1.62] | 0.398 |  |
| 2015 | 405 | 0.0 | [0–0.9] | – | – | – |  |
| 2016 | 488 | 0.4 | [0.1–1.5] | – | – | – |  |
| 2017 | 363 | 1.4 | [0.6–3.2] | 3.41 | [0.66–17.73] | 0.241 |  |
| 2018 | 421 | 1.2 | [0.5–2.7] | 0.85 | [0.24–2.98] | 0.953 |  |
| 2019 | 440 | 0.7 | [0.2–2] | 0.54 | [0.13–2.3] | 0.518 |  |
| 2020 | 452 | 1.8 | [0.9–3.5] | 2.71 | [0.71–10.3] | 0.241 |  |
| 2021 | 451 | 0.9 | [0.3–2.3] | 0.48 | [0.14–1.61] | 0.341 |  |
| 2022 | 581 | 0.2 | [0–1] | 0.2 | [0.02–1.77] | 0.241 |  |
| 2023 | 500 | 2.4 | [1.4–4.1] | 13.54 | [1.75–104.68] | 0.148 |  |
| 2024 | 442 | 1.1 | [0.5–2.6] | 0.46 | [0.16–1.33] | 0.241 |  |

### Table S59: Annual percentage of *Klebsiella pneumoniae* resistance to Ceftazidime from 2005 to 2024.

|  | Tested | Resistance | | Logistic model | |  | |
| --- | --- | --- | --- | --- | --- | --- | --- |
| Year | N | % | [95% CI] | OR | [95% CI] | P-value |  |
| 2005 | 224 | 10.3 | [6.9–14.9] | – | – | – |  |
| 2006 | 296 | 10.8 | [7.8–14.9] | 1 | [0.56–1.79] | 0.994 |  |
| 2007 | 343 | 8.7 | [6.2–12.2] | 0.83 | [0.48–1.41] | 0.784 |  |
| 2008 | 366 | 7.7 | [5.3–10.8] | 0.84 | [0.49–1.46] | 0.784 |  |
| 2009 | 436 | 6.0 | [4.1–8.6] | 0.8 | [0.46–1.41] | 0.784 |  |
| 2010 | 384 | 2.1 | [1.1–4.1] | 0.34 | [0.15–0.77] | 0.047 | * |
| 2011 | 519 | 10.4 | [8.1–13.3] | 5.47 | [2.56–11.72] | 0.000 | *** |
| 2012 | 485 | 13.6 | [10.8–16.9] | 1.31 | [0.88–1.94] | 0.425 |  |
| 2013 | 419 | 9.8 | [7.3–13] | 0.69 | [0.45–1.06] | 0.252 |  |
| 2014 | 474 | 9.3 | [7–12.2] | 0.91 | [0.58–1.44] | 0.871 |  |
| 2015 | 403 | 6.2 | [4.2–9] | 0.59 | [0.35–0.99] | 0.139 |  |
| 2016 | 487 | 8.0 | [5.9–10.8] | 1.37 | [0.81–2.32] | 0.518 |  |
| 2017 | 363 | 4.4 | [2.7–7] | 0.52 | [0.29–0.96] | 0.139 |  |
| 2018 | 419 | 4.8 | [3.1–7.3] | 1.08 | [0.55–2.13] | 0.883 |  |
| 2019 | 439 | 6.2 | [4.3–8.8] | 1.21 | [0.66–2.21] | 0.784 |  |
| 2020 | 434 | 6.2 | [4.3–8.9] | 1.06 | [0.61–1.85] | 0.883 |  |
| 2021 | 441 | 7.3 | [5.2–10.1] | 1.13 | [0.66–1.94] | 0.871 |  |
| 2022 | 557 | 7.5 | [5.6–10] | 1.08 | [0.67–1.76] | 0.883 |  |
| 2023 | 484 | 16.5 | [13.5–20.1] | 2.32 | [1.55–3.47] | 0.000 | *** |
| 2024 | 434 | 10.4 | [7.8–13.6] | 0.57 | [0.38–0.85] | 0.040 | * |

### Table S60: Annual percentage of *Klebsiella pneumoniae* resistance to Ciprofloxacin from 2005 to 2024.

|  | Tested | Resistance | | Logistic model | |  | |
| --- | --- | --- | --- | --- | --- | --- | --- |
| Year | N | % | [95% CI] | OR | [95% CI] | P-value |  |
| 2005 | 224 | 10.7 | [7.3–15.4] | – | – | – |  |
| 2006 | 296 | 15.9 | [12.2–20.5] | 1.52 | [0.89–2.58] | 0.260 |  |
| 2007 | 343 | 9.3 | [6.7–12.9] | 0.56 | [0.34–0.9] | 0.067 | . |
| 2008 | 366 | 9.6 | [7–13] | 1.01 | [0.61–1.67] | 0.971 |  |
| 2009 | 436 | 9.4 | [7–12.5] | 1.04 | [0.65–1.67] | 0.971 |  |
| 2010 | 384 | 3.4 | [2–5.7] | 0.34 | [0.18–0.64] | 0.005 | ** |
| 2011 | 518 | 12.9 | [10.3–16.1] | 4.31 | [2.35–7.89] | 0.000 | *** |
| 2012 | 485 | 17.1 | [14–20.7] | 1.35 | [0.95–1.92] | 0.260 |  |
| 2013 | 418 | 11.0 | [8.4–14.4] | 0.59 | [0.4–0.88] | 0.043 | * |
| 2014 | 474 | 10.5 | [8.1–13.6] | 0.92 | [0.6–1.41] | 0.896 |  |
| 2015 | 404 | 8.4 | [6.1–11.5] | 0.71 | [0.45–1.12] | 0.260 |  |
| 2016 | 488 | 9.0 | [6.8–11.9] | 1.11 | [0.7–1.78] | 0.883 |  |
| 2017 | 363 | 6.6 | [4.5–9.6] | 0.71 | [0.42–1.19] | 0.331 |  |
| 2018 | 420 | 6.2 | [4.3–8.9] | 0.92 | [0.52–1.63] | 0.915 |  |
| 2019 | 428 | 8.6 | [6.3–11.7] | 1.33 | [0.79–2.23] | 0.442 |  |
| 2020 | 404 | 8.4 | [6.1–11.5] | 1.02 | [0.63–1.65] | 0.971 |  |
| 2021 | 417 | 10.1 | [7.5–13.3] | 1.19 | [0.74–1.91] | 0.691 |  |
| 2022 | 521 | 7.1 | [5.2–9.6] | 0.7 | [0.44–1.12] | 0.260 |  |
| 2023 | 443 | 15.6 | [12.5–19.2] | 2.32 | [1.52–3.54] | 0.001 | *** |
| 2024 | 370 | 10.3 | [7.6–13.8] | 0.61 | [0.4–0.93] | 0.072 | . |

### Table S61: Annual percentage of *Klebsiella pneumoniae* resistance to Cefotaxime from 2005 to 2024.

|  | Tested | Resistance | | Logistic model | |  | |
| --- | --- | --- | --- | --- | --- | --- | --- |
| Year | N | % | [95% CI] | OR | [95% CI] | P-value |  |
| 2005 | 293 | 4.4 | [2.6–7.4] | – | – | – |  |
| 2006 | 296 | 9.8 | [6.9–13.7] | 2.14 | [1.09–4.21] | 0.096 | . |
| 2007 | 343 | 7.0 | [4.7–10.2] | 0.72 | [0.41–1.27] | 0.443 |  |
| 2008 | 365 | 6.6 | [4.5–9.6] | 0.91 | [0.51–1.64] | 0.850 |  |
| 2009 | 435 | 5.5 | [3.7–8.1] | 0.87 | [0.48–1.56] | 0.850 |  |
| 2010 | 383 | 2.3 | [1.2–4.4] | 0.42 | [0.19–0.92] | 0.096 | . |
| 2011 | 519 | 12.7 | [10.1–15.9] | 6.11 | [3.01–12.41] | 0.000 | *** |
| 2012 | 483 | 15.7 | [12.8–19.3] | 1.24 | [0.86–1.77] | 0.443 |  |
| 2013 | 415 | 11.6 | [8.8–15] | 0.71 | [0.48–1.05] | 0.203 |  |
| 2014 | 474 | 13.3 | [10.5–16.6] | 1.14 | [0.76–1.71] | 0.834 |  |
| 2015 | 404 | 9.7 | [7.1–12.9] | 0.63 | [0.41–0.97] | 0.096 | . |
| 2016 | 486 | 12.1 | [9.5–15.3] | 1.35 | [0.88–2.07] | 0.371 |  |
| 2017 | 363 | 7.2 | [4.9–10.3] | 0.55 | [0.34–0.9] | 0.077 | . |
| 2018 | 421 | 6.7 | [4.6–9.4] | 0.91 | [0.52–1.58] | 0.850 |  |
| 2019 | 436 | 8.0 | [5.8–11] | 1.14 | [0.68–1.91] | 0.850 |  |
| 2020 | 452 | 8.2 | [6–11.1] | 1.06 | [0.65–1.72] | 0.861 |  |
| 2021 | 448 | 9.2 | [6.8–12.2] | 1.1 | [0.69–1.75] | 0.850 |  |
| 2022 | 579 | 9.0 | [6.9–11.6] | 1 | [0.65–1.54] | 0.988 |  |
| 2023 | 499 | 18.0 | [14.9–21.6] | 2.13 | [1.47–3.07] | 0.001 | *** |
| 2024 | 442 | 11.8 | [9.1–15.1] | 0.6 | [0.41–0.87] | 0.044 | * |

### Table S62: Annual percentage of *Klebsiella pneumoniae* resistance to Gentamicin from 2005 to 2024.

|  | Tested | Resistance | | Logistic model | |  | |
| --- | --- | --- | --- | --- | --- | --- | --- |
| Year | N | % | [95% CI] | OR | [95% CI] | P-value |  |
| 2005 | 293 | 3.4 | [1.9–6.2] | – | – | – |  |
| 2006 | 296 | 10.1 | [7.2–14.1] | 2.91 | [1.4–6.04] | 0.032 | * |
| 2007 | 343 | 7.3 | [5–10.5] | 0.73 | [0.42–1.27] | 0.411 |  |
| 2008 | 366 | 7.7 | [5.3–10.8] | 1.04 | [0.59–1.83] | 0.932 |  |
| 2009 | 436 | 6.4 | [4.5–9.1] | 0.87 | [0.5–1.49] | 0.717 |  |
| 2010 | 383 | 2.9 | [1.6–5.1] | 0.44 | [0.22–0.89] | 0.085 | . |
| 2011 | 519 | 8.1 | [6–10.8] | 2.94 | [1.5–5.78] | 0.032 | * |
| 2012 | 485 | 5.6 | [3.9–8] | 0.63 | [0.38–1.04] | 0.175 |  |
| 2013 | 419 | 7.6 | [5.5–10.6] | 1.45 | [0.85–2.46] | 0.355 |  |
| 2014 | 474 | 7.8 | [5.7–10.6] | 1 | [0.61–1.63] | 0.990 |  |
| 2015 | 405 | 6.2 | [4.2–9] | 0.71 | [0.42–1.2] | 0.355 |  |
| 2016 | 488 | 11.1 | [8.6–14.2] | 1.98 | [1.21–3.25] | 0.032 | * |
| 2017 | 363 | 5.8 | [3.8–8.7] | 0.49 | [0.29–0.82] | 0.032 | * |
| 2018 | 421 | 4.8 | [3.1–7.2] | 0.8 | [0.43–1.5] | 0.615 |  |
| 2019 | 434 | 6.7 | [4.7–9.4] | 1.35 | [0.75–2.41] | 0.467 |  |
| 2020 | 430 | 5.1 | [3.4–7.6] | 0.79 | [0.45–1.39] | 0.558 |  |
| 2021 | 443 | 7.4 | [5.4–10.3] | 1.43 | [0.82–2.49] | 0.355 |  |
| 2022 | 558 | 6.6 | [4.8–9] | 0.91 | [0.56–1.47] | 0.776 |  |
| 2023 | 486 | 10.9 | [8.4–14] | 1.62 | [1.05–2.52] | 0.091 | . |
| 2024 | 394 | 6.9 | [4.8–9.8] | 0.59 | [0.37–0.96] | 0.091 | . |

### Table S63: Annual percentage of *Klebsiella pneumoniae* resistance to Imipenem from 2005 to 2024.

|  | Tested | Resistance | | Logistic model | |  | |
| --- | --- | --- | --- | --- | --- | --- | --- |
| Year | N | % | [95% CI] | OR | [95% CI] | P-value |  |
| 2005 | 170 | 0.0 | [0–2.2] | – | – | – |  |
| 2006 | 178 | 0.6 | [0.1–3.1] | – | – | – |  |
| 2007 | 240 | 0.0 | [0–1.6] | – | – | – |  |
| 2008 | 223 | 0.0 | [0–1.7] | – | – | – |  |
| 2009 | 264 | 0.0 | [0–1.4] | – | – | – |  |
| 2010 | 247 | 0.0 | [0–1.5] | – | – | – |  |
| 2011 | 472 | 0.2 | [0–1.2] | – | – | – |  |
| 2012 | 485 | 0.0 | [0–0.8] | – | – | – |  |
| 2013 | 418 | 0.2 | [0–1.3] | – | – | – |  |
| 2014 | 473 | 0.0 | [0–0.8] | – | – | – |  |
| 2015 | 401 | 0.0 | [0–0.9] | – | – | – |  |
| 2016 | 483 | 0.2 | [0–1.2] | – | – | – |  |
| 2017 | 359 | 0.0 | [0–1.1] | – | – | – |  |
| 2018 | 417 | 0.0 | [0–0.9] | – | – | – |  |
| 2019 | 435 | 0.9 | [0.4–2.3] | – | – | – |  |
| 2020 | 450 | 0.0 | [0–0.8] | – | – | – |  |
| 2021 | 447 | 0.2 | [0–1.3] | – | – | – |  |
| 2022 | 576 | 0.0 | [0–0.7] | – | – | – |  |
| 2023 | 408 | 0.7 | [0.3–2.1] | – | – | – |  |
| 2024 | 309 | 0.3 | [0.1–1.8] | 0.41 | [0.1–1.74] | 1.000 |  |

### Table S64: Annual percentage of *Klebsiella pneumoniae* resistance to Cotrimoxazole from 2005 to 2024.

|  | Tested | Resistance | | Logistic model | |  | |
| --- | --- | --- | --- | --- | --- | --- | --- |
| Year | N | % | [95% CI] | OR | [95% CI] | P-value |  |
| 2005 | 293 | 9.6 | [6.7–13.5] | – | – | – |  |
| 2006 | 296 | 13.2 | [9.8–17.5] | 1.36 | [0.81–2.29] | 0.470 |  |
| 2007 | 343 | 14.0 | [10.7–18.1] | 1.1 | [0.69–1.74] | 0.765 |  |
| 2008 | 366 | 11.5 | [8.6–15.1] | 0.79 | [0.5–1.23] | 0.470 |  |
| 2009 | 436 | 10.6 | [8–13.8] | 0.94 | [0.6–1.47] | 0.818 |  |
| 2010 | 383 | 9.1 | [6.6–12.4] | 0.87 | [0.54–1.38] | 0.744 |  |
| 2011 | 519 | 14.6 | [11.9–17.9] | 1.68 | [1.09–2.58] | 0.169 |  |
| 2012 | 485 | 21.2 | [17.8–25.1] | 1.54 | [1.11–2.15] | 0.169 |  |
| 2013 | 418 | 15.3 | [12.2–19.1] | 0.68 | [0.48–0.96] | 0.180 |  |
| 2014 | 473 | 11.6 | [9–14.8] | 0.71 | [0.48–1.05] | 0.402 |  |
| 2015 | 404 | 11.1 | [8.4–14.6] | 0.91 | [0.59–1.38] | 0.765 |  |
| 2016 | 487 | 13.6 | [10.8–16.9] | 1.28 | [0.85–1.92] | 0.470 |  |
| 2017 | 363 | 11.0 | [8.2–14.7] | 0.8 | [0.52–1.21] | 0.470 |  |
| 2018 | 421 | 8.6 | [6.2–11.6] | 0.74 | [0.46–1.2] | 0.470 |  |
| 2019 | 439 | 11.2 | [8.5–14.5] | 1.29 | [0.81–2.03] | 0.470 |  |
| 2020 | 448 | 13.8 | [10.9–17.3] | 1.31 | [0.88–1.97] | 0.470 |  |
| 2021 | 446 | 13.9 | [11–17.4] | 0.98 | [0.67–1.44] | 0.934 |  |
| 2022 | 580 | 14.8 | [12.2–18] | 1.09 | [0.77–1.56] | 0.765 |  |
| 2023 | 500 | 19.0 | [15.8–22.7] | 1.31 | [0.94–1.81] | 0.402 |  |
| 2024 | 439 | 17.3 | [14.1–21.1] | 0.89 | [0.64–1.25] | 0.737 |  |

### Table S65: Annual percentage of *Klebsiella pneumoniae* resistance to Piperacillin/Tazobactam from 2005 to 2024.

|  | Tested | Resistance | | Logistic model | |  | |
| --- | --- | --- | --- | --- | --- | --- | --- |
| Year | N | % | [95% CI] | OR | [95% CI] | P-value |  |
| 2005 | 170 | 2.4 | [0.9–5.9] | – | – | – |  |
| 2006 | 195 | 4.1 | [2.1–7.9] | 1.73 | [0.51–5.86] | 0.509 |  |
| 2007 | 240 | 0.8 | [0.2–3] | 0.2 | [0.04–0.95] | 0.168 |  |
| 2008 | 223 | 4.5 | [2.5–8.1] | 5.5 | [1.19–25.31] | 0.168 |  |
| 2009 | 264 | 6.4 | [4.1–10.1] | 1.52 | [0.68–3.38] | 0.480 |  |
| 2010 | 246 | 13.0 | [9.4–17.8] | 2.16 | [1.16–3.99] | 0.138 |  |
| 2011 | 426 | 10.3 | [7.8–13.6] | 0.78 | [0.48–1.26] | 0.480 |  |
| 2012 | 441 | 8.6 | [6.3–11.6] | 0.8 | [0.5–1.26] | 0.480 |  |
| 2013 | 416 | 17.1 | [13.8–21] | 2.22 | [1.46–3.37] | 0.004 | ** |
| 2014 | 473 | 17.1 | [14–20.8] | 0.99 | [0.7–1.41] | 0.961 |  |
| 2015 | 404 | 20.5 | [16.9–24.8] | 1.22 | [0.87–1.71] | 0.480 |  |
| 2016 | 485 | 21.0 | [17.6–24.9] | 1.04 | [0.75–1.44] | 0.914 |  |
| 2017 | 357 | 17.1 | [13.5–21.3] | 0.78 | [0.55–1.11] | 0.383 |  |
| 2018 | 417 | 16.8 | [13.5–20.7] | 0.97 | [0.67–1.42] | 0.936 |  |
| 2019 | 434 | 16.6 | [13.4–20.4] | 0.95 | [0.66–1.36] | 0.903 |  |
| 2020 | 439 | 19.8 | [16.4–23.8] | 1.27 | [0.9–1.79] | 0.383 |  |
| 2021 | 450 | 14.9 | [11.9–18.5] | 0.7 | [0.49–0.99] | 0.168 |  |
| 2022 | 576 | 15.5 | [12.7–18.6] | 1.06 | [0.75–1.49] | 0.903 |  |
| 2023 | 468 | 19.2 | [15.9–23] | 1.28 | [0.93–1.77] | 0.383 |  |
| 2024 | 434 | 15.7 | [12.6–19.4] | 0.78 | [0.55–1.1] | 0.383 |  |

**Figure S6: Annual percentage of *Morganella morganii* resistance from 2005 to 2024**


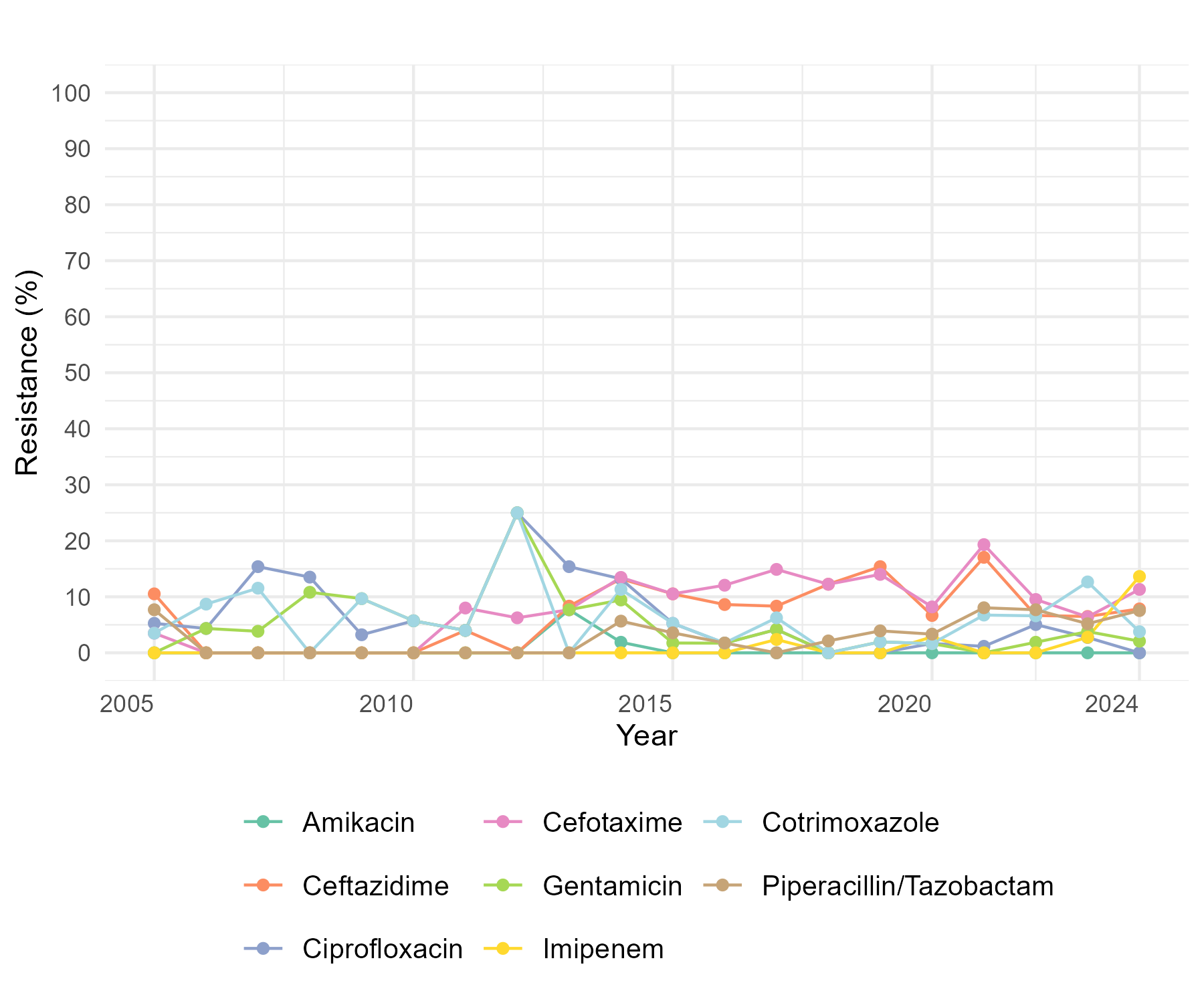


### Table S66: Logistic regression analysis of the effect of continuous time on the antibiotic resistance of *Morganella morganii* from 2005 to 2024.

| Antibiotic | β | OR | [95%CI] | P-value |  |
| --- | --- | --- | --- | --- | --- |
| Amikacin | -0.049 | 0.95 | [0.81–1.12] | 0.549 |  |
| Ceftazidime | 0.053 | 1.05 | [1.01–1.1] | 0.023 | * |
| Ciprofloxacin | -0.106 | 0.90 | [0.85–0.95] | 0.000 | *** |
| Cefotaxime | 0.077 | 1.08 | [1.03–1.13] | 0.001 | *** |
| Gentamicin | -0.080 | 0.92 | [0.87–0.98] | 0.004 | ** |
| Imipenem | 0.508 | 1.66 | [1.11–2.48] | 0.013 | * |
| Cotrimoxazole | -0.004 | 1.00 | [0.95–1.04] | 0.867 |  |
| Piperacillin/Tazobactam | 0.088 | 1.09 | [1–1.19] | 0.053 | . |

### Table S67: Annual percentage of *Morganella morganii* resistance to Amikacin from 2005 to 2024.

|  | Tested | Resistance | | Logistic model | |  | |
| --- | --- | --- | --- | --- | --- | --- | --- |
| Year | N | % | [95% CI] | OR | [95% CI] | P-value |  |
| 2005 | 51 | 0.0 | [0–7] | – | – | – |  |
| 2006 | 8 | 0.0 | [0–32.4] | – | – | – |  |
| 2007 | 26 | 0.0 | [0–12.9] | – | – | – |  |
| 2008 | 37 | 0.0 | [0–9.4] | – | – | – |  |
| 2009 | 31 | 0.0 | [0–11] | – | – | – |  |
| 2010 | 35 | 0.0 | [0–9.9] | – | – | – |  |
| 2011 | 25 | 0.0 | [0–13.3] | – | – | – |  |
| 2012 | 16 | 0.0 | [0–19.4] | – | – | – |  |
| 2013 | 13 | 7.7 | [1.4–33.3] | – | – | – |  |
| 2014 | 53 | 1.9 | [0.3–9.9] | 0.24 | [0.14–0.41] | 0.000 | *** |
| 2015 | 56 | 0.0 | [0–6.4] | – | – | – |  |
| 2016 | 58 | 0.0 | [0–6.2] | – | – | – |  |
| 2017 | 48 | 0.0 | [0–7.4] | – | – | – |  |
| 2018 | 49 | 0.0 | [0–7.3] | – | – | – |  |
| 2019 | 52 | 0.0 | [0–6.9] | – | – | – |  |
| 2020 | 60 | 0.0 | [0–6] | – | – | – |  |
| 2021 | 88 | 0.0 | [0–4.2] | – | – | – |  |
| 2022 | 106 | 0.0 | [0–3.5] | – | – | – |  |
| 2023 | 79 | 0.0 | [0–4.6] | – | – | – |  |
| 2024 | 46 | 0.0 | [0–7.7] | – | – | – |  |

### Table S68: Annual percentage of *Morganella morganii* resistance to Ceftazidime from 2005 to 2024.

|  | Tested | Resistance | | Logistic model | |  | |
| --- | --- | --- | --- | --- | --- | --- | --- |
| Year | N | % | [95% CI] | OR | [95% CI] | P-value |  |
| 2005 | 38 | 10.5 | [4.2–24.1] | – | – | – |  |
| 2006 | 23 | 0.0 | [0–14.3] | – | – | – |  |
| 2007 | 26 | 0.0 | [0–12.9] | – | – | – |  |
| 2008 | 37 | 0.0 | [0–9.4] | – | – | – |  |
| 2009 | 31 | 0.0 | [0–11] | – | – | – |  |
| 2010 | 35 | 0.0 | [0–9.9] | – | – | – |  |
| 2011 | 25 | 4.0 | [0.7–19.5] | – | – | – |  |
| 2012 | 16 | 0.0 | [0–19.4] | – | – | – |  |
| 2013 | 12 | 8.3 | [1.5–35.4] | – | – | – |  |
| 2014 | 53 | 13.2 | [6.5–24.8] | 1.79 | [0.24–13.4] | 1.000 |  |
| 2015 | 57 | 10.5 | [4.9–21.1] | 0.76 | [0.26–2.2] | 1.000 |  |
| 2016 | 58 | 8.6 | [3.7–18.6] | 0.78 | [0.25–2.44] | 1.000 |  |
| 2017 | 48 | 8.3 | [3.3–19.6] | 1.03 | [0.29–3.6] | 1.000 |  |
| 2018 | 49 | 12.2 | [5.7–24.2] | 1.49 | [0.44–5.02] | 1.000 |  |
| 2019 | 52 | 15.4 | [8–27.5] | 1.3 | [0.46–3.69] | 1.000 |  |
| 2020 | 60 | 6.7 | [2.6–15.9] | 0.33 | [0.1–1.04] | 0.375 |  |
| 2021 | 88 | 17.0 | [10.6–26.2] | 3.39 | [1.18–9.79] | 0.226 |  |
| 2022 | 105 | 6.7 | [3.3–13.1] | 0.32 | [0.14–0.77] | 0.209 |  |
| 2023 | 77 | 6.5 | [2.8–14.3] | 0.97 | [0.33–2.85] | 1.000 |  |
| 2024 | 51 | 7.8 | [3.1–18.5] | 1.29 | [0.37–4.5] | 1.000 |  |

### Table S69: Annual percentage of *Morganella morganii* resistance to Ciprofloxacin from 2005 to 2024.

|  | Tested | Resistance | | Logistic model | |  | |
| --- | --- | --- | --- | --- | --- | --- | --- |
| Year | N | % | [95% CI] | OR | [95% CI] | P-value |  |
| 2005 | 38 | 5.3 | [1.5–17.3] | – | – | – |  |
| 2006 | 23 | 4.3 | [0.8–21] | 0.92 | [0.1–8.36] | 1.000 |  |
| 2007 | 26 | 15.4 | [6.2–33.5] | 3.59 | [0.47–27.62] | 0.695 |  |
| 2008 | 37 | 13.5 | [5.9–28] | 0.91 | [0.25–3.28] | 1.000 |  |
| 2009 | 31 | 3.2 | [0.6–16.2] | 0.25 | [0.03–1.83] | 0.695 |  |
| 2010 | 35 | 5.7 | [1.6–18.6] | 1.66 | [0.18–14.97] | 1.000 |  |
| 2011 | 25 | 4.0 | [0.7–19.5] | 0.54 | [0.06–4.96] | 1.000 |  |
| 2012 | 16 | 25.0 | [10.2–49.5] | 11.92 | [1.47–96.45] | 0.383 |  |
| 2013 | 13 | 15.4 | [4.3–42.2] | 0.4 | [0.07–2.26] | 0.820 |  |
| 2014 | 53 | 13.2 | [6.5–24.8] | 0.83 | [0.18–3.86] | 1.000 |  |
| 2015 | 57 | 5.3 | [1.8–14.4] | 0.38 | [0.11–1.36] | 0.695 |  |
| 2016 | 58 | 1.7 | [0.3–9.1] | 0.27 | [0.03–2.08] | 0.695 |  |
| 2017 | 48 | 4.2 | [1.2–14] | 2.59 | [0.29–22.73] | 0.929 |  |
| 2018 | 49 | 0.0 | [0–7.3] | – | – | – |  |
| 2019 | 52 | 0.0 | [0–6.9] | – | – | – |  |
| 2020 | 60 | 1.7 | [0.3–8.9] | – | – | – |  |
| 2021 | 86 | 1.2 | [0.2–6.3] | 0.74 | [0.06–8.91] | 1.000 |  |
| 2022 | 99 | 5.1 | [2.2–11.3] | 4.81 | [0.69–33.38] | 0.695 |  |
| 2023 | 73 | 2.7 | [0.8–9.5] | 0.56 | [0.13–2.48] | 0.935 |  |
| 2024 | 45 | 0.0 | [0–7.9] | – | – | – |  |

### Table S70: Annual percentage of *Morganella morganii* resistance to Cefotaxime from 2005 to 2024.

|  | Tested | Resistance | | Logistic model | |  | |
| --- | --- | --- | --- | --- | --- | --- | --- |
| Year | N | % | [95% CI] | OR | [95% CI] | P-value |  |
| 2005 | 57 | 3.5 | [1–11.9] | – | – | – |  |
| 2006 | 23 | 0.0 | [0–14.3] | – | – | – |  |
| 2007 | 26 | 0.0 | [0–12.9] | – | – | – |  |
| 2008 | 37 | 0.0 | [0–9.4] | – | – | – |  |
| 2009 | 31 | 0.0 | [0–11] | – | – | – |  |
| 2010 | 35 | 0.0 | [0–9.9] | – | – | – |  |
| 2011 | 25 | 8.0 | [2.2–25] | – | – | – |  |
| 2012 | 16 | 6.2 | [1.1–28.3] | 0.89 | [0.09–9.11] | 1.000 |  |
| 2013 | 13 | 7.7 | [1.4–33.3] | 1.13 | [0.08–16.58] | 1.000 |  |
| 2014 | 52 | 13.5 | [6.7–25.3] | 1.92 | [0.25–14.88] | 1.000 |  |
| 2015 | 57 | 10.5 | [4.9–21.1] | 0.75 | [0.25–2.23] | 1.000 |  |
| 2016 | 58 | 12.1 | [6–22.9] | 1.13 | [0.38–3.34] | 1.000 |  |
| 2017 | 47 | 14.9 | [7.4–27.7] | 1.33 | [0.46–3.82] | 1.000 |  |
| 2018 | 49 | 12.2 | [5.7–24.2] | 0.78 | [0.26–2.34] | 1.000 |  |
| 2019 | 50 | 14.0 | [7–26.2] | 1.15 | [0.38–3.44] | 1.000 |  |
| 2020 | 61 | 8.2 | [3.6–17.8] | 0.49 | [0.16–1.53] | 1.000 |  |
| 2021 | 88 | 19.3 | [12.4–28.8] | 3.03 | [1.12–8.19] | 0.303 |  |
| 2022 | 105 | 9.5 | [5.3–16.6] | 0.42 | [0.19–0.93] | 0.303 |  |
| 2023 | 78 | 6.4 | [2.8–14.1] | 0.65 | [0.23–1.83] | 1.000 |  |
| 2024 | 53 | 11.3 | [5.3–22.6] | 1.9 | [0.59–6.09] | 1.000 |  |

### Table S71: Annual percentage of *Morganella morganii* resistance to Gentamicin from 2005 to 2024.

|  | Tested | Resistance | | Logistic model | |  | |
| --- | --- | --- | --- | --- | --- | --- | --- |
| Year | N | % | [95% CI] | OR | [95% CI] | P-value |  |
| 2005 | 57 | 0.0 | [0–6.3] | – | – | – |  |
| 2006 | 23 | 4.3 | [0.8–21] | – | – | – |  |
| 2007 | 26 | 3.8 | [0.7–18.9] | 0.81 | [0.07–9.22] | 0.990 |  |
| 2008 | 37 | 10.8 | [4.3–24.7] | 3.18 | [0.46–22.14] | 0.990 |  |
| 2009 | 31 | 9.7 | [3.3–24.9] | 1.14 | [0.28–4.57] | 0.990 |  |
| 2010 | 35 | 5.7 | [1.6–18.6] | 0.53 | [0.1–2.7] | 0.990 |  |
| 2011 | 25 | 4.0 | [0.7–19.5] | 0.46 | [0.05–3.82] | 0.990 |  |
| 2012 | 16 | 25.0 | [10.2–49.5] | 13.67 | [1.8–103.91] | 0.218 |  |
| 2013 | 13 | 7.7 | [1.4–33.3] | 0.16 | [0.02–1.29] | 0.542 |  |
| 2014 | 53 | 9.4 | [4.1–20.3] | 1.29 | [0.19–8.93] | 0.990 |  |
| 2015 | 57 | 1.8 | [0.3–9.3] | 0.18 | [0.03–1.16] | 0.542 |  |
| 2016 | 58 | 1.7 | [0.3–9.1] | 0.82 | [0.07–8.95] | 0.990 |  |
| 2017 | 48 | 4.2 | [1.2–14] | 2.76 | [0.34–22.13] | 0.990 |  |
| 2018 | 49 | 0.0 | [0–7.3] | – | – | – |  |
| 2019 | 51 | 2.0 | [0.3–10.3] | – | – | – |  |
| 2020 | 61 | 1.6 | [0.3–8.7] | 0.75 | [0.07–8.17] | 0.990 |  |
| 2021 | 88 | 0.0 | [0–4.2] | – | – | – |  |
| 2022 | 106 | 1.9 | [0.5–6.6] | – | – | – |  |
| 2023 | 79 | 3.8 | [1.3–10.6] | 2.1 | [0.44–9.91] | 0.990 |  |
| 2024 | 48 | 2.1 | [0.4–10.9] | 0.54 | [0.08–3.85] | 0.990 |  |

### Table S72: Annual percentage of *Morganella morganii* resistance to Imipenem from 2005 to 2024.

|  | Tested | Resistance | | Logistic model | |  | |
| --- | --- | --- | --- | --- | --- | --- | --- |
| Year | N | % | [95% CI] | OR | [95% CI] | P-value |  |
| 2005 | 26 | 0.0 | [0–12.9] | – | – | – |  |
| 2006 | 5 | 0.0 | [0–43.4] | – | – | – |  |
| 2007 | 5 | 0.0 | [0–43.4] | – | – | – |  |
| 2008 | 10 | 0.0 | [0–27.8] | – | – | – |  |
| 2009 | 9 | 0.0 | [0–29.9] | – | – | – |  |
| 2010 | 4 | 0.0 | [0–49] | – | – | – |  |
| 2011 | 21 | 0.0 | [0–15.5] | – | – | – |  |
| 2012 | 15 | 0.0 | [0–20.4] | – | – | – |  |
| 2013 | 13 | 0.0 | [0–22.8] | – | – | – |  |
| 2014 | 38 | 0.0 | [0–9.2] | – | – | – |  |
| 2015 | 53 | 0.0 | [0–6.8] | – | – | – |  |
| 2016 | 48 | 0.0 | [0–7.4] | – | – | – |  |
| 2017 | 42 | 2.4 | [0.4–12.3] | – | – | – |  |
| 2018 | 40 | 0.0 | [0–8.8] | – | – | – |  |
| 2019 | 35 | 0.0 | [0–9.9] | – | – | – |  |
| 2020 | 35 | 2.9 | [0.5–14.5] | – | – | – |  |
| 2021 | 59 | 0.0 | [0–6.1] | – | – | – |  |
| 2022 | 62 | 0.0 | [0–5.8] | – | – | – |  |
| 2023 | 72 | 2.8 | [0.8–9.6] | – | – | – |  |
| 2024 | 44 | 13.6 | [6.4–26.7] | 6.04 | [2.35–15.52] | 0.004 | ** |

### Table S73: Annual percentage of *Morganella morganii* resistance to Cotrimoxazole from 2005 to 2024.

|  | Tested | Resistance | | Logistic model | |  | |
| --- | --- | --- | --- | --- | --- | --- | --- |
| Year | N | % | [95% CI] | OR | [95% CI] | P-value |  |
| 2005 | 57 | 3.5 | [1–11.9] | – | – | – |  |
| 2006 | 23 | 8.7 | [2.4–26.8] | 2.9 | [0.43–19.77] | 0.655 |  |
| 2007 | 26 | 11.5 | [4–29] | 1.26 | [0.21–7.53] | 0.992 |  |
| 2008 | 37 | 0.0 | [0–9.4] | – | – | – |  |
| 2009 | 31 | 9.7 | [3.3–24.9] | – | – | – |  |
| 2010 | 35 | 5.7 | [1.6–18.6] | 0.55 | [0.09–3.22] | 0.992 |  |
| 2011 | 25 | 4.0 | [0.7–19.5] | 0.51 | [0.05–5.19] | 0.992 |  |
| 2012 | 16 | 25.0 | [10.2–49.5] | 11.15 | [1.25–99.82] | 0.590 |  |
| 2013 | 13 | 0.0 | [0–22.8] | – | – | – |  |
| 2014 | 53 | 11.3 | [5.3–22.6] | – | – | – |  |
| 2015 | 57 | 5.3 | [1.8–14.4] | 0.44 | [0.11–1.73] | 0.655 |  |
| 2016 | 57 | 1.8 | [0.3–9.3] | 0.29 | [0.03–2.51] | 0.655 |  |
| 2017 | 48 | 6.2 | [2.1–16.8] | 4 | [0.46–34.72] | 0.655 |  |
| 2018 | 49 | 0.0 | [0–7.3] | – | – | – |  |
| 2019 | 51 | 2.0 | [0.3–10.3] | – | – | – |  |
| 2020 | 61 | 1.6 | [0.3–8.7] | 0.75 | [0.05–10.39] | 0.992 |  |
| 2021 | 89 | 6.7 | [3.1–13.9] | 4.99 | [0.66–37.44] | 0.622 |  |
| 2022 | 106 | 6.6 | [3.2–13] | 0.96 | [0.33–2.8] | 0.992 |  |
| 2023 | 79 | 12.7 | [7–21.8] | 2.09 | [0.8–5.45] | 0.622 |  |
| 2024 | 53 | 3.8 | [1–12.8] | 0.26 | [0.06–1.14] | 0.622 |  |

### Table S74: Annual percentage of *Morganella morganii* resistance to Piperacillin/Tazobactam from 2005 to 2024.

|  | Tested | Resistance | | Logistic model | |  | |
| --- | --- | --- | --- | --- | --- | --- | --- |
| Year | N | % | [95% CI] | OR | [95% CI] | P-value |  |
| 2005 | 26 | 7.7 | [2.1–24.1] | – | – | – |  |
| 2006 | 5 | 0.0 | [0–43.4] | – | – | – |  |
| 2007 | 6 | 0.0 | [0–39] | – | – | – |  |
| 2008 | 10 | 0.0 | [0–27.8] | – | – | – |  |
| 2009 | 9 | 0.0 | [0–29.9] | – | – | – |  |
| 2010 | 5 | 0.0 | [0–43.4] | – | – | – |  |
| 2011 | 21 | 0.0 | [0–15.5] | – | – | – |  |
| 2012 | 15 | 0.0 | [0–20.4] | – | – | – |  |
| 2013 | 13 | 0.0 | [0–22.8] | – | – | – |  |
| 2014 | 53 | 5.7 | [1.9–15.4] | – | – | – |  |
| 2015 | 56 | 3.6 | [1–12.1] | 0.61 | [0.11–3.35] | 1.000 |  |
| 2016 | 57 | 1.8 | [0.3–9.3] | 0.49 | [0.05–4.67] | 1.000 |  |
| 2017 | 48 | 0.0 | [0–7.4] | – | – | – |  |
| 2018 | 47 | 2.1 | [0.4–11.1] | – | – | – |  |
| 2019 | 51 | 3.9 | [1.1–13.2] | 1.9 | [0.2–18.31] | 1.000 |  |
| 2020 | 60 | 3.3 | [0.9–11.4] | 0.8 | [0.12–5.2] | 1.000 |  |
| 2021 | 87 | 8.0 | [4–15.7] | 2.64 | [0.59–11.86] | 1.000 |  |
| 2022 | 104 | 7.7 | [3.9–14.4] | 0.93 | [0.35–2.51] | 1.000 |  |
| 2023 | 77 | 5.2 | [2–12.6] | 0.66 | [0.21–2.07] | 1.000 |  |
| 2024 | 53 | 7.5 | [3–17.9] | 1.52 | [0.4–5.8] | 1.000 |  |

### Figure S7: Annual percentage of *Proteus mirabilis* resistance from 2005 to 2024


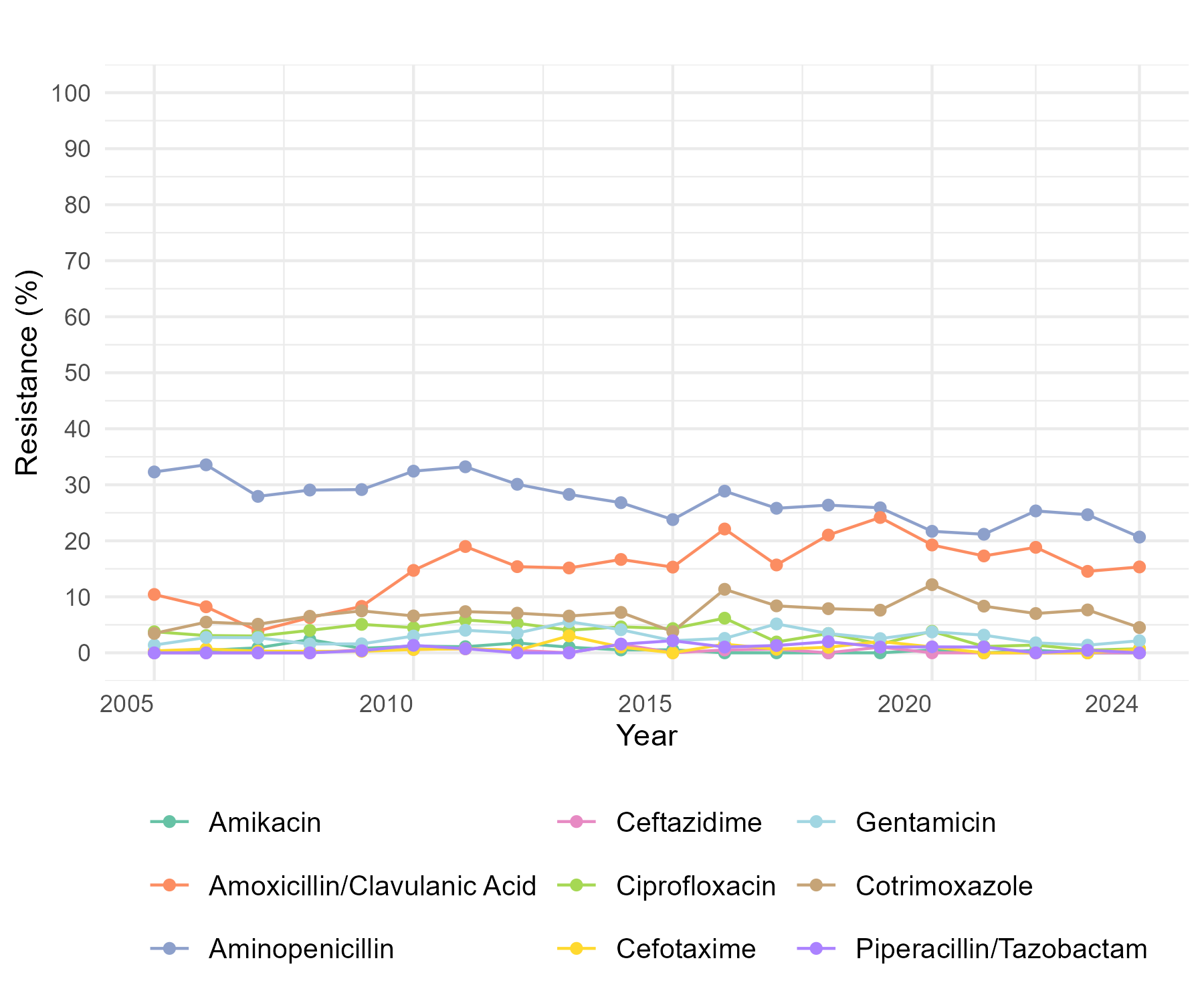


### Table S75: Logistic regression analysis of the effect of continuous time on the antibiotic resistance of *Proteus mirabilis* from 2005 to 2024.

| Antibiotic | β | OR | [95%CI] | P-value |  |
| --- | --- | --- | --- | --- | --- |
| Amikacin | -0.095 | 0.91 | [0.85–0.98] | 0.010 | ** |
| Aminopenicillin | -0.029 | 0.97 | [0.96–0.98] | 0.000 | *** |
| Amoxicillin/Clavulanic Acid | 0.052 | 1.05 | [1.04–1.07] | 0.000 | *** |
| Ceftazidime | -0.027 | 0.97 | [0.89–1.07] | 0.562 |  |
| Ciprofloxacin | -0.079 | 0.92 | [0.9–0.95] | 0.000 | *** |
| Cefotaxime | 0.000 | 1.00 | [0.94–1.06] | 0.997 |  |
| Gentamicin | -0.003 | 1.00 | [0.97–1.03] | 0.833 |  |
| Cotrimoxazole | 0.006 | 1.01 | [0.99–1.03] | 0.542 |  |
| Piperacillin/Tazobactam | 0.018 | 1.02 | [0.96–1.08] | 0.577 |  |

### Table S76: Annual percentage of *Proteus mirabilis* resistance to Amikacin from 2005 to 2024.

|  | Tested | Resistance | | Logistic model | |  | |
| --- | --- | --- | --- | --- | --- | --- | --- |
| Year | N | % | [95% CI] | OR | [95% CI] | P-value |  |
| 2005 | 251 | 0.0 | [0–1.5] | – | – | – |  |
| 2006 | 232 | 0.4 | [0.1–2.4] | – | – | – |  |
| 2007 | 333 | 0.9 | [0.3–2.6] | 1.33 | [0.19–9.35] | 1.000 |  |
| 2008 | 382 | 2.4 | [1.2–4.4] | 3.95 | [1.14–13.72] | 0.418 |  |
| 2009 | 374 | 0.8 | [0.3–2.3] | 0.34 | [0.12–0.97] | 0.418 |  |
| 2010 | 334 | 1.2 | [0.5–3] | 1.51 | [0.45–5.09] | 1.000 |  |
| 2011 | 274 | 1.1 | [0.4–3.2] | 0.96 | [0.28–3.27] | 1.000 |  |
| 2012 | 227 | 1.8 | [0.7–4.4] | 1.62 | [0.48–5.5] | 1.000 |  |
| 2013 | 198 | 1.0 | [0.3–3.6] | 0.56 | [0.14–2.25] | 1.000 |  |
| 2014 | 194 | 0.5 | [0.1–2.9] | 0.51 | [0.07–3.58] | 1.000 |  |
| 2015 | 185 | 0.5 | [0.1–3] | 1.08 | [0.11–10.23] | 1.000 |  |
| 2016 | 194 | 0.0 | [0–1.9] | – | – | – |  |
| 2017 | 155 | 0.0 | [0–2.4] | – | – | – |  |
| 2018 | 203 | 0.0 | [0–1.9] | – | – | – |  |
| 2019 | 197 | 0.0 | [0–1.9] | – | – | – |  |
| 2020 | 191 | 0.5 | [0.1–2.9] | – | – | – |  |
| 2021 | 193 | 0.0 | [0–2] | – | – | – |  |
| 2022 | 228 | 0.4 | [0.1–2.4] | – | – | – |  |
| 2023 | 222 | 0.0 | [0–1.7] | – | – | – |  |
| 2024 | 124 | 0.0 | [0–3] | – | – | – |  |

### Table S77: Annual percentage of *Proteus mirabilis* resistance to Aminopenicillin from 2005 to 2024.

|  | Tested | Resistance | | Logistic model | |  | |
| --- | --- | --- | --- | --- | --- | --- | --- |
| Year | N | % | [95% CI] | OR | [95% CI] | P-value |  |
| 2005 | 288 | 32.3 | [27.2–37.9] | – | – | – |  |
| 2006 | 292 | 33.6 | [28.4–39.2] | 1.04 | [0.73–1.47] | 0.980 |  |
| 2007 | 333 | 27.9 | [23.4–33] | 0.78 | [0.55–1.11] | 0.980 |  |
| 2008 | 382 | 29.1 | [24.7–33.8] | 1.05 | [0.75–1.45] | 0.980 |  |
| 2009 | 374 | 29.1 | [24.8–33.9] | 1.01 | [0.74–1.38] | 0.980 |  |
| 2010 | 333 | 32.4 | [27.6–37.6] | 1.16 | [0.84–1.6] | 0.980 |  |
| 2011 | 274 | 33.2 | [27.9–39] | 1.02 | [0.72–1.43] | 0.980 |  |
| 2012 | 226 | 30.1 | [24.5–36.4] | 0.87 | [0.59–1.27] | 0.980 |  |
| 2013 | 198 | 28.3 | [22.5–34.9] | 0.92 | [0.6–1.4] | 0.980 |  |
| 2014 | 194 | 26.8 | [21.1–33.4] | 0.93 | [0.59–1.45] | 0.980 |  |
| 2015 | 185 | 23.8 | [18.2–30.4] | 0.84 | [0.53–1.34] | 0.980 |  |
| 2016 | 194 | 28.9 | [22.9–35.6] | 1.31 | [0.83–2.08] | 0.980 |  |
| 2017 | 155 | 25.8 | [19.6–33.2] | 0.83 | [0.52–1.34] | 0.980 |  |
| 2018 | 201 | 26.4 | [20.8–32.9] | 1.04 | [0.64–1.67] | 0.980 |  |
| 2019 | 197 | 25.9 | [20.3–32.4] | 0.99 | [0.63–1.56] | 0.980 |  |
| 2020 | 189 | 21.7 | [16.4–28.1] | 0.78 | [0.49–1.25] | 0.980 |  |
| 2021 | 189 | 21.2 | [15.9–27.5] | 0.98 | [0.6–1.6] | 0.980 |  |
| 2022 | 225 | 25.3 | [20.1–31.4] | 1.25 | [0.79–1.98] | 0.980 |  |
| 2023 | 219 | 24.7 | [19.4–30.8] | 0.98 | [0.63–1.5] | 0.980 |  |
| 2024 | 150 | 20.7 | [15–27.8] | 0.79 | [0.48–1.3] | 0.980 |  |

### Table S78: Annual percentage of *Proteus mirabilis* resistance to Amoxicillin/Clavulanic Acid from 2005 to 2024.

|  | Tested | Resistance | | Logistic model | |  | |
| --- | --- | --- | --- | --- | --- | --- | --- |
| Year | N | % | [95% CI] | OR | [95% CI] | P-value |  |
| 2005 | 288 | 10.4 | [7.4–14.5] | – | – | – |  |
| 2006 | 292 | 8.2 | [5.6–11.9] | 0.75 | [0.42–1.32] | 0.504 |  |
| 2007 | 333 | 3.9 | [2.3–6.6] | 0.48 | [0.24–0.96] | 0.356 |  |
| 2008 | 382 | 6.3 | [4.3–9.2] | 1.62 | [0.81–3.23] | 0.504 |  |
| 2009 | 374 | 8.3 | [5.9–11.5] | 1.35 | [0.78–2.35] | 0.504 |  |
| 2010 | 333 | 14.7 | [11.3–18.9] | 1.9 | [1.18–3.06] | 0.161 |  |
| 2011 | 274 | 19.0 | [14.8–24] | 1.33 | [0.86–2.04] | 0.504 |  |
| 2012 | 221 | 15.4 | [11.2–20.7] | 0.78 | [0.48–1.25] | 0.504 |  |
| 2013 | 198 | 15.2 | [10.8–20.8] | 0.99 | [0.58–1.69] | 0.966 |  |
| 2014 | 186 | 16.7 | [12–22.7] | 1.12 | [0.64–1.93] | 0.818 |  |
| 2015 | 183 | 15.3 | [10.8–21.2] | 0.89 | [0.51–1.56] | 0.818 |  |
| 2016 | 190 | 22.1 | [16.8–28.5] | 1.59 | [0.93–2.7] | 0.504 |  |
| 2017 | 153 | 15.7 | [10.8–22.3] | 0.63 | [0.36–1.1] | 0.504 |  |
| 2018 | 195 | 21.0 | [15.9–27.3] | 1.44 | [0.83–2.52] | 0.504 |  |
| 2019 | 178 | 24.2 | [18.5–30.9] | 1.22 | [0.75–1.98] | 0.625 |  |
| 2020 | 187 | 19.3 | [14.2–25.5] | 0.73 | [0.44–1.21] | 0.504 |  |
| 2021 | 185 | 17.3 | [12.5–23.4] | 0.88 | [0.52–1.5] | 0.818 |  |
| 2022 | 223 | 18.8 | [14.2–24.5] | 1.09 | [0.66–1.82] | 0.818 |  |
| 2023 | 220 | 14.5 | [10.5–19.8] | 0.75 | [0.45–1.23] | 0.504 |  |
| 2024 | 150 | 15.3 | [10.4–22] | 1.05 | [0.59–1.88] | 0.917 |  |

### Table S79: Annual percentage of *Proteus mirabilis* resistance to Ceftazidime from 2005 to 2024.

|  | Tested | Resistance | | Logistic model | |  | |
| --- | --- | --- | --- | --- | --- | --- | --- |
| Year | N | % | [95% CI] | OR | [95% CI] | P-value |  |
| 2005 | 239 | 0.0 | [0–1.6] | – | – | – |  |
| 2006 | 292 | 0.0 | [0–1.3] | – | – | – |  |
| 2007 | 333 | 0.0 | [0–1.1] | – | – | – |  |
| 2008 | 382 | 0.0 | [0–1] | – | – | – |  |
| 2009 | 374 | 0.5 | [0.1–1.9] | – | – | – |  |
| 2010 | 334 | 0.6 | [0.2–2.2] | 1.12 | [0.31–4.06] | 1.000 |  |
| 2011 | 274 | 0.7 | [0.2–2.6] | 1.11 | [0.31–4.02] | 1.000 |  |
| 2012 | 227 | 0.4 | [0.1–2.5] | 0.57 | [0.12–2.77] | 1.000 |  |
| 2013 | 198 | 0.0 | [0–1.9] | – | – | – |  |
| 2014 | 194 | 1.5 | [0.5–4.4] | – | – | – |  |
| 2015 | 185 | 0.0 | [0–2] | – | – | – |  |
| 2016 | 194 | 0.5 | [0.1–2.9] | – | – | – |  |
| 2017 | 155 | 0.6 | [0.1–3.6] | 1.11 | [0.18–6.84] | 1.000 |  |
| 2018 | 203 | 0.0 | [0–1.9] | – | – | – |  |
| 2019 | 196 | 1.0 | [0.3–3.6] | – | – | – |  |
| 2020 | 190 | 0.0 | [0–2] | – | – | – |  |
| 2021 | 191 | 0.0 | [0–2] | – | – | – |  |
| 2022 | 224 | 0.0 | [0–1.7] | – | – | – |  |
| 2023 | 220 | 0.0 | [0–1.7] | – | – | – |  |
| 2024 | 154 | 0.0 | [0–2.4] | – | – | – |  |

### Table S80: Annual percentage of *Proteus mirabilis* resistance to Ciprofloxacin from 2005 to 2024.

|  | Tested | Resistance | | Logistic model | |  | |
| --- | --- | --- | --- | --- | --- | --- | --- |
| Year | N | % | [95% CI] | OR | [95% CI] | P-value |  |
| 2005 | 239 | 3.8 | [2–7] | – | – | – |  |
| 2006 | 292 | 3.1 | [1.6–5.8] | 0.81 | [0.3–2.21] | 0.933 |  |
| 2007 | 333 | 3.0 | [1.6–5.4] | 1.16 | [0.44–3.1] | 0.933 |  |
| 2008 | 374 | 4.0 | [2.4–6.5] | 1.27 | [0.53–3.05] | 0.933 |  |
| 2009 | 374 | 5.1 | [3.3–7.8] | 1.25 | [0.59–2.63] | 0.933 |  |
| 2010 | 334 | 4.5 | [2.7–7.3] | 0.88 | [0.42–1.86] | 0.933 |  |
| 2011 | 273 | 5.9 | [3.6–9.3] | 1.1 | [0.51–2.39] | 0.933 |  |
| 2012 | 227 | 5.3 | [3–9] | 0.83 | [0.36–1.9] | 0.933 |  |
| 2013 | 198 | 4.0 | [2.1–7.8] | 0.86 | [0.32–2.29] | 0.933 |  |
| 2014 | 194 | 4.6 | [2.5–8.6] | 1.04 | [0.37–2.94] | 0.943 |  |
| 2015 | 185 | 4.3 | [2.2–8.3] | 0.84 | [0.3–2.39] | 0.933 |  |
| 2016 | 194 | 6.2 | [3.6–10.5] | 1.6 | [0.6–4.27] | 0.933 |  |
| 2017 | 155 | 1.9 | [0.7–5.5] | 0.23 | [0.06–0.92] | 0.707 |  |
| 2018 | 203 | 3.4 | [1.7–6.9] | 2.01 | [0.47–8.6] | 0.933 |  |
| 2019 | 193 | 1.6 | [0.5–4.5] | 0.44 | [0.1–1.89] | 0.933 |  |
| 2020 | 182 | 3.8 | [1.9–7.7] | 2.29 | [0.54–9.77] | 0.933 |  |
| 2021 | 177 | 1.1 | [0.3–4] | 0.29 | [0.05–1.56] | 0.933 |  |
| 2022 | 217 | 1.4 | [0.5–4] | 1.13 | [0.17–7.62] | 0.943 |  |
| 2023 | 211 | 0.5 | [0.1–2.6] | 0.37 | [0.03–4.11] | 0.933 |  |
| 2024 | 137 | 0.7 | [0.1–4] | 1.37 | [0.07–25.72] | 0.933 |  |

### Table S81: Annual percentage of *Proteus mirabilis* resistance to Cefotaxime from 2005 to 2024.

|  | Tested | Resistance | | Logistic model | |  | |
| --- | --- | --- | --- | --- | --- | --- | --- |
| Year | N | % | [95% CI] | OR | [95% CI] | P-value |  |
| 2005 | 288 | 0.3 | [0.1–1.9] | – | – | – |  |
| 2006 | 292 | 0.7 | [0.2–2.5] | 1.98 | [0.22–17.47] | 1.000 |  |
| 2007 | 333 | 0.3 | [0.1–1.7] | 0.48 | [0.05–4.2] | 1.000 |  |
| 2008 | 382 | 0.3 | [0–1.5] | 0.81 | [0.07–10.01] | 1.000 |  |
| 2009 | 374 | 0.3 | [0–1.5] | 1.02 | [0.08–12.59] | 1.000 |  |
| 2010 | 333 | 0.6 | [0.2–2.2] | 2.26 | [0.26–19.95] | 1.000 |  |
| 2011 | 274 | 0.7 | [0.2–2.6] | 1.16 | [0.19–6.87] | 1.000 |  |
| 2012 | 227 | 0.4 | [0.1–2.5] | 0.56 | [0.06–4.98] | 1.000 |  |
| 2013 | 198 | 3.0 | [1.4–6.5] | 7.43 | [1.08–50.99] | 0.782 |  |
| 2014 | 194 | 1.0 | [0.3–3.7] | 0.32 | [0.07–1.4] | 1.000 |  |
| 2015 | 185 | 0.0 | [0–2] | – | – | – |  |
| 2016 | 194 | 1.5 | [0.5–4.4] | – | – | – |  |
| 2017 | 155 | 0.6 | [0.1–3.6] | 0.38 | [0.05–3.02] | 1.000 |  |
| 2018 | 203 | 1.0 | [0.3–3.5] | 1.58 | [0.18–13.97] | 1.000 |  |
| 2019 | 197 | 2.0 | [0.8–5.1] | 1.99 | [0.42–9.36] | 1.000 |  |
| 2020 | 191 | 1.0 | [0.3–3.7] | 0.51 | [0.11–2.39] | 1.000 |  |
| 2021 | 193 | 0.0 | [0–2] | – | – | – |  |
| 2022 | 228 | 0.0 | [0–1.7] | – | – | – |  |
| 2023 | 222 | 0.0 | [0–1.7] | – | – | – |  |
| 2024 | 154 | 0.6 | [0.1–3.6] | – | – | – |  |

### Table S82: Annual percentage of *Proteus mirabilis* resistance to Gentamicin from 2005 to 2024.

|  | Tested | Resistance | | Logistic model | |  | |
| --- | --- | --- | --- | --- | --- | --- | --- |
| Year | N | % | [95% CI] | OR | [95% CI] | P-value |  |
| 2005 | 288 | 1.4 | [0.5–3.5] | – | – | – |  |
| 2006 | 292 | 2.7 | [1.4–5.3] | 2.01 | [0.6–6.76] | 0.856 |  |
| 2007 | 333 | 2.7 | [1.4–5.1] | 1.04 | [0.4–2.74] | 0.968 |  |
| 2008 | 383 | 1.6 | [0.7–3.4] | 0.55 | [0.19–1.56] | 0.856 |  |
| 2009 | 374 | 1.6 | [0.7–3.5] | 1.02 | [0.33–3.2] | 0.968 |  |
| 2010 | 334 | 3.0 | [1.6–5.4] | 1.89 | [0.68–5.25] | 0.856 |  |
| 2011 | 273 | 4.0 | [2.3–7.1] | 1.27 | [0.53–3.05] | 0.856 |  |
| 2012 | 227 | 3.5 | [1.8–6.8] | 0.85 | [0.33–2.14] | 0.856 |  |
| 2013 | 198 | 5.6 | [3.1–9.7] | 1.67 | [0.66–4.25] | 0.856 |  |
| 2014 | 194 | 4.1 | [2.1–7.9] | 0.71 | [0.28–1.82] | 0.856 |  |
| 2015 | 185 | 2.2 | [0.8–5.4] | 0.49 | [0.15–1.66] | 0.856 |  |
| 2016 | 194 | 2.6 | [1.1–5.9] | 1.24 | [0.33–4.69] | 0.856 |  |
| 2017 | 155 | 5.2 | [2.6–9.9] | 1.87 | [0.6–5.85] | 0.856 |  |
| 2018 | 203 | 3.4 | [1.7–6.9] | 0.67 | [0.24–1.9] | 0.856 |  |
| 2019 | 197 | 2.5 | [1.1–5.8] | 0.74 | [0.23–2.37] | 0.856 |  |
| 2020 | 187 | 3.7 | [1.8–7.5] | 1.44 | [0.45–4.61] | 0.856 |  |
| 2021 | 190 | 3.2 | [1.5–6.7] | 0.84 | [0.28–2.56] | 0.856 |  |
| 2022 | 224 | 1.8 | [0.7–4.5] | 0.54 | [0.15–1.93] | 0.856 |  |
| 2023 | 219 | 1.4 | [0.5–3.9] | 0.8 | [0.18–3.59] | 0.856 |  |
| 2024 | 139 | 2.2 | [0.7–6.2] | 1.52 | [0.3–7.61] | 0.856 |  |

### Table S83: Annual percentage of *Proteus mirabilis* resistance to Cotrimoxazole from 2005 to 2024.

|  | Tested | Resistance | | Logistic model | |  | |
| --- | --- | --- | --- | --- | --- | --- | --- |
| Year | N | % | [95% CI] | OR | [95% CI] | P-value |  |
| 2005 | 288 | 3.5 | [1.9–6.3] | – | – | – |  |
| 2006 | 292 | 5.5 | [3.4–8.7] | 1.62 | [0.72–3.64] | 0.655 |  |
| 2007 | 333 | 5.1 | [3.2–8] | 1.01 | [0.5–2.04] | 0.998 |  |
| 2008 | 383 | 6.5 | [4.5–9.5] | 1.24 | [0.66–2.35] | 0.998 |  |
| 2009 | 374 | 7.5 | [5.2–10.6] | 1.16 | [0.66–2.03] | 0.998 |  |
| 2010 | 334 | 6.6 | [4.4–9.8] | 0.86 | [0.48–1.55] | 0.998 |  |
| 2011 | 272 | 7.4 | [4.8–11.1] | 1.01 | [0.54–1.9] | 0.998 |  |
| 2012 | 226 | 7.1 | [4.4–11.2] | 0.93 | [0.47–1.85] | 0.998 |  |
| 2013 | 198 | 6.6 | [3.9–10.9] | 0.98 | [0.46–2.09] | 0.998 |  |
| 2014 | 194 | 7.2 | [4.3–11.7] | 1.07 | [0.49–2.34] | 0.998 |  |
| 2015 | 185 | 3.8 | [1.8–7.6] | 0.47 | [0.19–1.2] | 0.655 |  |
| 2016 | 194 | 11.3 | [7.6–16.6] | 3.47 | [1.44–8.36] | 0.104 |  |
| 2017 | 155 | 8.4 | [5–13.8] | 0.61 | [0.3–1.27] | 0.655 |  |
| 2018 | 203 | 7.9 | [4.9–12.4] | 0.98 | [0.45–2.1] | 0.998 |  |
| 2019 | 197 | 7.6 | [4.7–12.2] | 1 | [0.48–2.09] | 0.998 |  |
| 2020 | 189 | 12.2 | [8.2–17.6] | 1.58 | [0.79–3.14] | 0.655 |  |
| 2021 | 192 | 8.3 | [5.2–13.1] | 0.66 | [0.34–1.31] | 0.655 |  |
| 2022 | 228 | 7.0 | [4.4–11.1] | 0.79 | [0.38–1.62] | 0.998 |  |
| 2023 | 222 | 7.7 | [4.8–11.9] | 1.17 | [0.57–2.37] | 0.998 |  |
| 2024 | 155 | 4.5 | [2.2–9] | 0.53 | [0.21–1.32] | 0.655 |  |

### Table S84: Annual percentage of *Proteus mirabilis* resistance to Piperacillin/Tazobactam from 2005 to 2024.

|  | Tested | Resistance | | Logistic model | |  | |
| --- | --- | --- | --- | --- | --- | --- | --- |
| Year | N | % | [95% CI] | OR | [95% CI] | P-value |  |
| 2005 | 191 | 0.0 | [0–2] | – | – | – |  |
| 2006 | 212 | 0.0 | [0–1.8] | – | – | – |  |
| 2007 | 233 | 0.0 | [0–1.6] | – | – | – |  |
| 2008 | 276 | 0.0 | [0–1.4] | – | – | – |  |
| 2009 | 267 | 0.4 | [0.1–2.1] | – | – | – |  |
| 2010 | 224 | 1.3 | [0.5–3.9] | 3.67 | [0.78–17.28] | 1.000 |  |
| 2011 | 136 | 0.7 | [0.1–4] | 0.5 | [0.1–2.36] | 1.000 |  |
| 2012 | 129 | 0.0 | [0–2.9] | – | – | – |  |
| 2013 | 197 | 0.0 | [0–1.9] | – | – | – |  |
| 2014 | 194 | 1.5 | [0.5–4.4] | – | – | – |  |
| 2015 | 185 | 2.2 | [0.8–5.4] | 1.35 | [0.48–3.8] | 1.000 |  |
| 2016 | 194 | 1.0 | [0.3–3.7] | 0.5 | [0.15–1.6] | 1.000 |  |
| 2017 | 153 | 1.3 | [0.4–4.6] | 1.12 | [0.29–4.31] | 1.000 |  |
| 2018 | 201 | 2.0 | [0.8–5] | 1.71 | [0.53–5.52] | 1.000 |  |
| 2019 | 193 | 1.0 | [0.3–3.7] | 0.44 | [0.14–1.42] | 1.000 |  |
| 2020 | 189 | 1.1 | [0.3–3.8] | 1.04 | [0.27–4.02] | 1.000 |  |
| 2021 | 193 | 1.0 | [0.3–3.7] | 0.89 | [0.23–3.43] | 1.000 |  |
| 2022 | 227 | 0.0 | [0–1.7] | – | – | – |  |
| 2023 | 221 | 0.5 | [0.1–2.5] | – | – | – |  |
| 2024 | 154 | 0.0 | [0–2.4] | – | – | – |  |

### Figure S8: Annual percentage of *Salmonella spp.* resistance from 2005 to 2024.


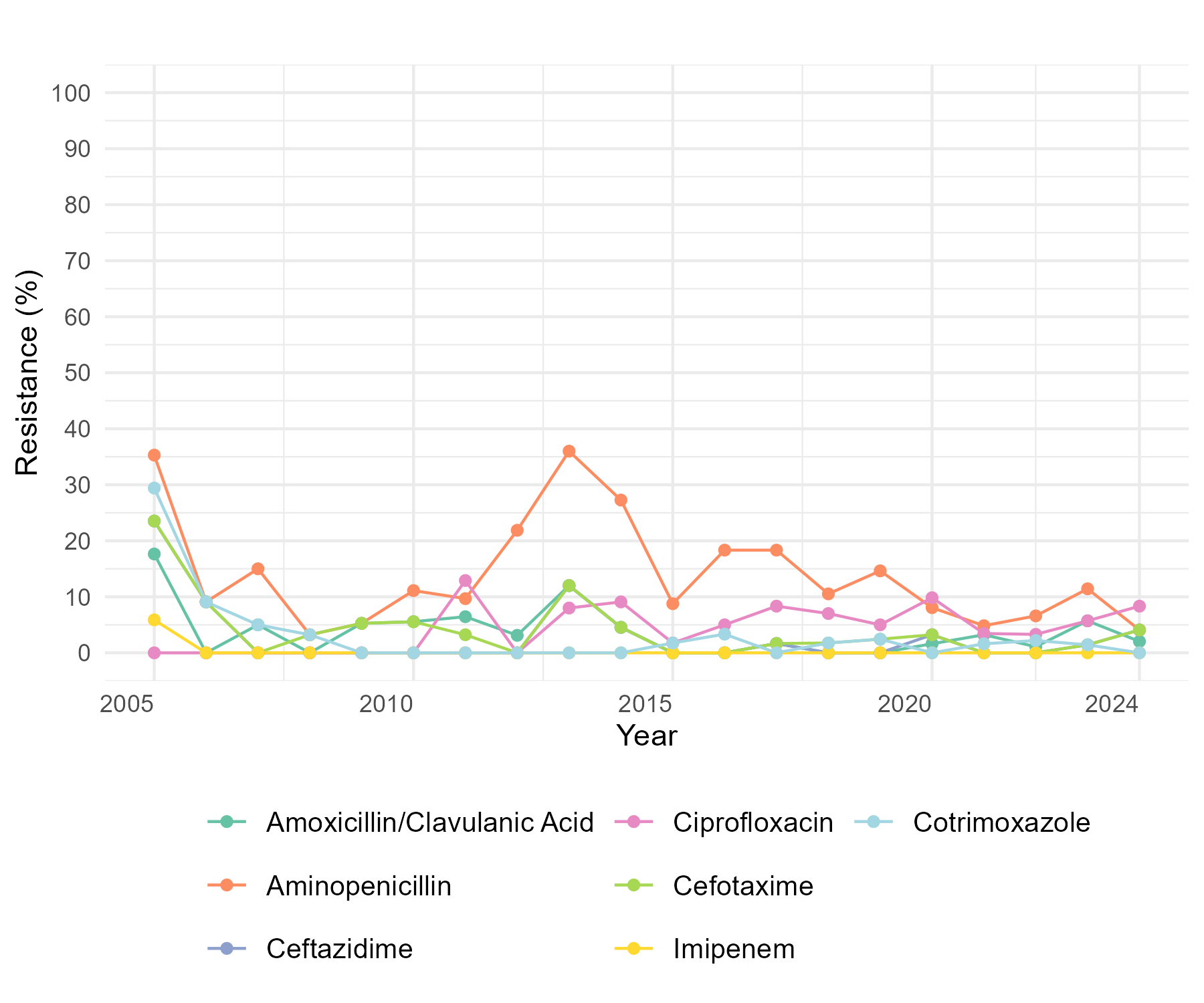


### Table S85: Logistic regression analysis of the effect of continuous time on the antibiotic resistance of *Salmonella spp.* from 2005 to 2024.

| Antibiotic | β | OR | [95%CI] | P-value |  |
| --- | --- | --- | --- | --- | --- |
| Aminopenicillin | -0.053 | 0.95 | [0.91–0.99] | 0.007 | ** |
| Amoxicillin/Clavulanic Acid | -0.066 | 0.94 | [0.87–1.01] | 0.090 | . |
| Ceftazidime | -0.102 | 0.90 | [0.82–1] | 0.043 | * |
| Ciprofloxacin | 0.053 | 1.05 | [0.99–1.13] | 0.110 |  |
| Cefotaxime | -0.114 | 0.89 | [0.82–0.97] | 0.007 | ** |
| Imipenem | -35.019 | 0.00 | [0–0] | 0.000 | *** |
| Cotrimoxazole | -0.125 | 0.88 | [0.8–0.97] | 0.014 | * |

### Table S86: Annual percentage of *Salmonella spp.* resistance to Aminopenicillin from 2005 to 2024.

|  | Tested | Resistance | | Logistic model | |  | |
| --- | --- | --- | --- | --- | --- | --- | --- |
| Year | N | % | [95% CI] | OR | [95% CI] | P-value |  |
| 2005 | 17 | 35.3 | [17.3–58.7] | – | – | – |  |
| 2006 | 11 | 9.1 | [1.6–37.7] | 0.17 | [0.02–1.78] | 0.483 |  |
| 2007 | 20 | 15.0 | [5.2–36] | 2.14 | [0.19–24.57] | 0.728 |  |
| 2008 | 31 | 3.2 | [0.6–16.2] | 0.17 | [0.02–1.82] | 0.483 |  |
| 2009 | 19 | 5.3 | [0.9–24.6] | 1.95 | [0.11–34.76] | 0.728 |  |
| 2010 | 18 | 11.1 | [3.1–32.8] | 1.89 | [0.15–23.94] | 0.728 |  |
| 2011 | 31 | 9.7 | [3.3–24.9] | 0.84 | [0.12–5.76] | 0.905 |  |
| 2012 | 32 | 21.9 | [11–38.8] | 2.82 | [0.64–12.46] | 0.483 |  |
| 2013 | 25 | 36.0 | [20.2–55.5] | 2.29 | [0.69–7.61] | 0.483 |  |
| 2014 | 44 | 27.3 | [16.3–41.8] | 0.55 | [0.18–1.65] | 0.590 |  |
| 2015 | 57 | 8.8 | [3.8–18.9] | 0.28 | [0.09–0.91] | 0.483 |  |
| 2016 | 60 | 18.3 | [10.6–29.9] | 2.41 | [0.76–7.64] | 0.483 |  |
| 2017 | 60 | 18.3 | [10.6–29.9] | 0.94 | [0.37–2.43] | 0.907 |  |
| 2018 | 57 | 10.5 | [4.9–21.1] | 0.56 | [0.19–1.67] | 0.590 |  |
| 2019 | 41 | 14.6 | [6.9–28.4] | 1.4 | [0.41–4.8] | 0.728 |  |
| 2020 | 62 | 8.1 | [3.5–17.5] | 0.54 | [0.15–1.94] | 0.590 |  |
| 2021 | 62 | 4.8 | [1.7–13.3] | 0.55 | [0.12–2.49] | 0.699 |  |
| 2022 | 91 | 6.6 | [3.1–13.6] | 1.44 | [0.34–6.15] | 0.728 |  |
| 2023 | 70 | 11.4 | [5.9–21] | 1.78 | [0.58–5.5] | 0.590 |  |
| 2024 | 49 | 4.1 | [1.1–13.7] | 0.32 | [0.06–1.6] | 0.483 |  |

### Table S87: Annual percentage of *Salmonella spp.* resistance to Amoxicillin/Clavulanic Acid from 2005 to 2024.

|  | Tested | Resistance | | Logistic model | |  | |
| --- | --- | --- | --- | --- | --- | --- | --- |
| Year | N | % | [95% CI] | OR | [95% CI] | P-value |  |
| 2005 | 17 | 17.6 | [6.2–41] | – | – | – |  |
| 2006 | 11 | 0.0 | [0–25.9] | – | – | – |  |
| 2007 | 20 | 5.0 | [0.9–23.6] | – | – | – |  |
| 2008 | 31 | 0.0 | [0–11] | – | – | – |  |
| 2009 | 19 | 5.3 | [0.9–24.6] | – | – | – |  |
| 2010 | 18 | 5.6 | [1–25.8] | 0.91 | [0.08–10.04] | 1.000 |  |
| 2011 | 31 | 6.5 | [1.8–20.7] | 1.18 | [0.15–9.41] | 1.000 |  |
| 2012 | 32 | 3.1 | [0.6–15.7] | 0.49 | [0.06–3.83] | 1.000 |  |
| 2013 | 25 | 12.0 | [4.2–30] | 4.7 | [0.67–33] | 1.000 |  |
| 2014 | 44 | 4.5 | [1.3–15.1] | 0.33 | [0.07–1.56] | 1.000 |  |
| 2015 | 57 | 0.0 | [0–6.3] | – | – | – |  |
| 2016 | 60 | 0.0 | [0–6] | – | – | – |  |
| 2017 | 60 | 1.7 | [0.3–8.9] | – | – | – |  |
| 2018 | 57 | 0.0 | [0–6.3] | – | – | – |  |
| 2019 | 41 | 0.0 | [0–8.6] | – | – | – |  |
| 2020 | 62 | 1.6 | [0.3–8.6] | – | – | – |  |
| 2021 | 62 | 3.2 | [0.9–11] | 1.96 | [0.26–14.87] | 1.000 |  |
| 2022 | 91 | 1.1 | [0.2–6] | 0.34 | [0.05–2.6] | 1.000 |  |
| 2023 | 70 | 5.7 | [2.2–13.8] | 5.31 | [0.84–33.78] | 1.000 |  |
| 2024 | 49 | 2.0 | [0.4–10.7] | 0.33 | [0.05–2.13] | 1.000 |  |

### Table S88: Annual percentage of *Salmonella spp.* resistance to Ceftazidime from 2005 to 2024.

|  | Tested | Resistance | | Logistic model | |  | |
| --- | --- | --- | --- | --- | --- | --- | --- |
| Year | N | % | [95% CI] | OR | [95% CI] | P-value |  |
| 2005 | 17 | 23.5 | [9.6–47.3] | – | – | – |  |
| 2006 | 11 | 9.1 | [1.6–37.7] | 0.29 | [0.06–1.38] | 1.000 |  |
| 2007 | 20 | 0.0 | [0–16.1] | – | – | – |  |
| 2008 | 31 | 0.0 | [0–11] | – | – | – |  |
| 2009 | 19 | 0.0 | [0–16.8] | – | – | – |  |
| 2010 | 18 | 0.0 | [0–17.6] | – | – | – |  |
| 2011 | 30 | 0.0 | [0–11.4] | – | – | – |  |
| 2012 | 32 | 0.0 | [0–10.7] | – | – | – |  |
| 2013 | 25 | 12.0 | [4.2–30] | – | – | – |  |
| 2014 | 44 | 4.5 | [1.3–15.1] | 0.33 | [0.09–1.17] | 1.000 |  |
| 2015 | 55 | 0.0 | [0–6.5] | – | – | – |  |
| 2016 | 60 | 0.0 | [0–6] | – | – | – |  |
| 2017 | 60 | 1.7 | [0.3–8.9] | – | – | – |  |
| 2018 | 57 | 0.0 | [0–6.3] | – | – | – |  |
| 2019 | 41 | 0.0 | [0–8.6] | – | – | – |  |
| 2020 | 62 | 3.2 | [0.9–11] | – | – | – |  |
| 2021 | 61 | 0.0 | [0–5.9] | – | – | – |  |
| 2022 | 91 | 0.0 | [0–4.1] | – | – | – |  |
| 2023 | 70 | 1.4 | [0.3–7.7] | – | – | – |  |
| 2024 | 49 | 4.1 | [1.1–13.7] | 2.77 | [0.54–14.08] | 1.000 |  |

### Table S89: Annual percentage of *Salmonella spp.* resistance to Ciprofloxacin from 2005 to 2024.

|  | Tested | Resistance | | Logistic model | |  | |
| --- | --- | --- | --- | --- | --- | --- | --- |
| Year | N | % | [95% CI] | OR | [95% CI] | P-value |  |
| 2005 | 17 | 0.0 | [0–18.4] | – | – | – |  |
| 2006 | 11 | 0.0 | [0–25.9] | – | – | – |  |
| 2007 | 20 | 0.0 | [0–16.1] | – | – | – |  |
| 2008 | 31 | 0.0 | [0–11] | – | – | – |  |
| 2009 | 19 | 0.0 | [0–16.8] | – | – | – |  |
| 2010 | 18 | 0.0 | [0–17.6] | – | – | – |  |
| 2011 | 31 | 12.9 | [5.1–28.9] | – | – | – |  |
| 2012 | 32 | 0.0 | [0–10.7] | – | – | – |  |
| 2013 | 25 | 8.0 | [2.2–25] | – | – | – |  |
| 2014 | 44 | 9.1 | [3.6–21.2] | 1.16 | [0.22–5.97] | 1.000 |  |
| 2015 | 57 | 1.8 | [0.3–9.3] | 0.18 | [0.02–1.38] | 1.000 |  |
| 2016 | 60 | 5.0 | [1.7–13.7] | 2.79 | [0.33–23.25] | 1.000 |  |
| 2017 | 60 | 8.3 | [3.6–18.1] | 1.77 | [0.45–6.92] | 1.000 |  |
| 2018 | 57 | 7.0 | [2.8–16.7] | 0.86 | [0.24–3.04] | 1.000 |  |
| 2019 | 40 | 5.0 | [1.4–16.5] | 0.68 | [0.14–3.4] | 1.000 |  |
| 2020 | 61 | 9.8 | [4.6–19.8] | 2.01 | [0.44–9.26] | 1.000 |  |
| 2021 | 58 | 3.4 | [1–11.7] | 0.33 | [0.07–1.5] | 1.000 |  |
| 2022 | 91 | 3.3 | [1.1–9.2] | 0.95 | [0.18–5.09] | 1.000 |  |
| 2023 | 70 | 5.7 | [2.2–13.8] | 1.8 | [0.44–7.36] | 1.000 |  |
| 2024 | 48 | 8.3 | [3.3–19.6] | 1.5 | [0.4–5.65] | 1.000 |  |

### Table S90: Annual percentage of *Salmonella spp.* resistance to Cefotaxime from 2005 to 2024.

|  | Tested | Resistance | | Logistic model | |  | |
| --- | --- | --- | --- | --- | --- | --- | --- |
| Year | N | % | [95% CI] | OR | [95% CI] | P-value |  |
| 2005 | 17 | 23.5 | [9.6–47.3] | – | – | – |  |
| 2006 | 11 | 9.1 | [1.6–37.7] | 0.31 | [0.05–2.16] | 1.000 |  |
| 2007 | 20 | 0.0 | [0–16.1] | – | – | – |  |
| 2008 | 31 | 3.2 | [0.6–16.2] | – | – | – |  |
| 2009 | 19 | 5.3 | [0.9–24.6] | 1.9 | [0.18–19.88] | 1.000 |  |
| 2010 | 18 | 5.6 | [1–25.8] | 0.89 | [0.08–9.47] | 1.000 |  |
| 2011 | 31 | 3.2 | [0.6–16.2] | 0.57 | [0.05–5.91] | 1.000 |  |
| 2012 | 32 | 0.0 | [0–10.7] | – | – | – |  |
| 2013 | 25 | 12.0 | [4.2–30] | – | – | – |  |
| 2014 | 44 | 4.5 | [1.3–15.1] | 0.32 | [0.07–1.51] | 1.000 |  |
| 2015 | 57 | 0.0 | [0–6.3] | – | – | – |  |
| 2016 | 60 | 0.0 | [0–6] | – | – | – |  |
| 2017 | 60 | 1.7 | [0.3–8.9] | – | – | – |  |
| 2018 | 57 | 1.8 | [0.3–9.3] | 1.12 | [0.11–11.14] | 1.000 |  |
| 2019 | 41 | 2.4 | [0.4–12.6] | 1.35 | [0.14–13.49] | 1.000 |  |
| 2020 | 62 | 3.2 | [0.9–11] | 1.42 | [0.19–10.49] | 1.000 |  |
| 2021 | 62 | 0.0 | [0–5.8] | – | – | – |  |
| 2022 | 91 | 0.0 | [0–4.1] | – | – | – |  |
| 2023 | 70 | 1.4 | [0.3–7.7] | – | – | – |  |
| 2024 | 49 | 4.1 | [1.1–13.7] | 2.83 | [0.38–20.83] | 1.000 |  |

### Table S91: Annual percentage of *Salmonella spp.* resistance to Imipenem from 2005 to 2024.

|  | Tested | Resistance | | Logistic model | |  | |
| --- | --- | --- | --- | --- | --- | --- | --- |
| Year | N | % | [95% CI] | OR | [95% CI] | P-value |  |
| 2005 | 17 | 5.9 | [1–27] | – | – | – |  |
| 2006 | 11 | 0.0 | [0–25.9] | – | – | – |  |
| 2007 | 20 | 0.0 | [0–16.1] | – | – | – |  |
| 2008 | 29 | 0.0 | [0–11.7] | – | – | – |  |
| 2009 | 19 | 0.0 | [0–16.8] | – | – | – |  |
| 2010 | 18 | 0.0 | [0–17.6] | – | – | – |  |
| 2011 | 31 | 0.0 | [0–11] | – | – | – |  |
| 2012 | 32 | 0.0 | [0–10.7] | – | – | – |  |
| 2013 | 25 | 0.0 | [0–13.3] | – | – | – |  |
| 2014 | 44 | 0.0 | [0–8] | – | – | – |  |
| 2015 | 57 | 0.0 | [0–6.3] | – | – | – |  |
| 2016 | 60 | 0.0 | [0–6] | – | – | – |  |
| 2017 | 60 | 0.0 | [0–6] | – | – | – |  |
| 2018 | 57 | 0.0 | [0–6.3] | – | – | – |  |
| 2019 | 41 | 0.0 | [0–8.6] | – | – | – |  |
| 2020 | 62 | 0.0 | [0–5.8] | – | – | – |  |
| 2021 | 62 | 0.0 | [0–5.8] | – | – | – |  |
| 2022 | 91 | 0.0 | [0–4.1] | – | – | – |  |
| 2023 | 70 | 0.0 | [0–5.2] | – | – | – |  |
| 2024 | 49 | 0.0 | [0–7.3] | – | – | – |  |

### Table S92: Annual percentage of *Salmonella spp.* resistance to Cotrimoxazole from 2005 to 2024.

|  | Tested | Resistance | | Logistic model | |  | |
| --- | --- | --- | --- | --- | --- | --- | --- |
| Year | N | % | [95% CI] | OR | [95% CI] | P-value |  |
| 2005 | 17 | 29.4 | [13.3–53.1] | – | – | – |  |
| 2006 | 11 | 9.1 | [1.6–37.7] | 0.24 | [0.04–1.52] | 1.000 |  |
| 2007 | 20 | 5.0 | [0.9–23.6] | 0.69 | [0.07–6.99] | 1.000 |  |
| 2008 | 31 | 3.2 | [0.6–16.2] | 0.59 | [0.06–5.77] | 1.000 |  |
| 2009 | 19 | 0.0 | [0–16.8] | – | – | – |  |
| 2010 | 18 | 0.0 | [0–17.6] | – | – | – |  |
| 2011 | 31 | 0.0 | [0–11] | – | – | – |  |
| 2012 | 32 | 0.0 | [0–10.7] | – | – | – |  |
| 2013 | 25 | 0.0 | [0–13.3] | – | – | – |  |
| 2014 | 44 | 0.0 | [0–8] | – | – | – |  |
| 2015 | 57 | 1.8 | [0.3–9.3] | – | – | – |  |
| 2016 | 60 | 3.3 | [0.9–11.4] | 2.44 | [0.34–17.28] | 1.000 |  |
| 2017 | 60 | 0.0 | [0–6] | – | – | – |  |
| 2018 | 57 | 1.8 | [0.3–9.3] | – | – | – |  |
| 2019 | 41 | 2.4 | [0.4–12.6] | 1.39 | [0.15–13.06] | 1.000 |  |
| 2020 | 62 | 0.0 | [0–5.8] | – | – | – |  |
| 2021 | 62 | 1.6 | [0.3–8.6] | – | – | – |  |
| 2022 | 91 | 2.2 | [0.6–7.7] | 1.44 | [0.21–10.02] | 1.000 |  |
| 2023 | 70 | 1.4 | [0.3–7.7] | 0.6 | [0.09–4.14] | 1.000 |  |
| 2024 | 49 | 0.0 | [0–7.3] | – | – | – |  |

### Figure S9: Annual percentage of *Shigella spp.* resistance from 2005 to 2024.


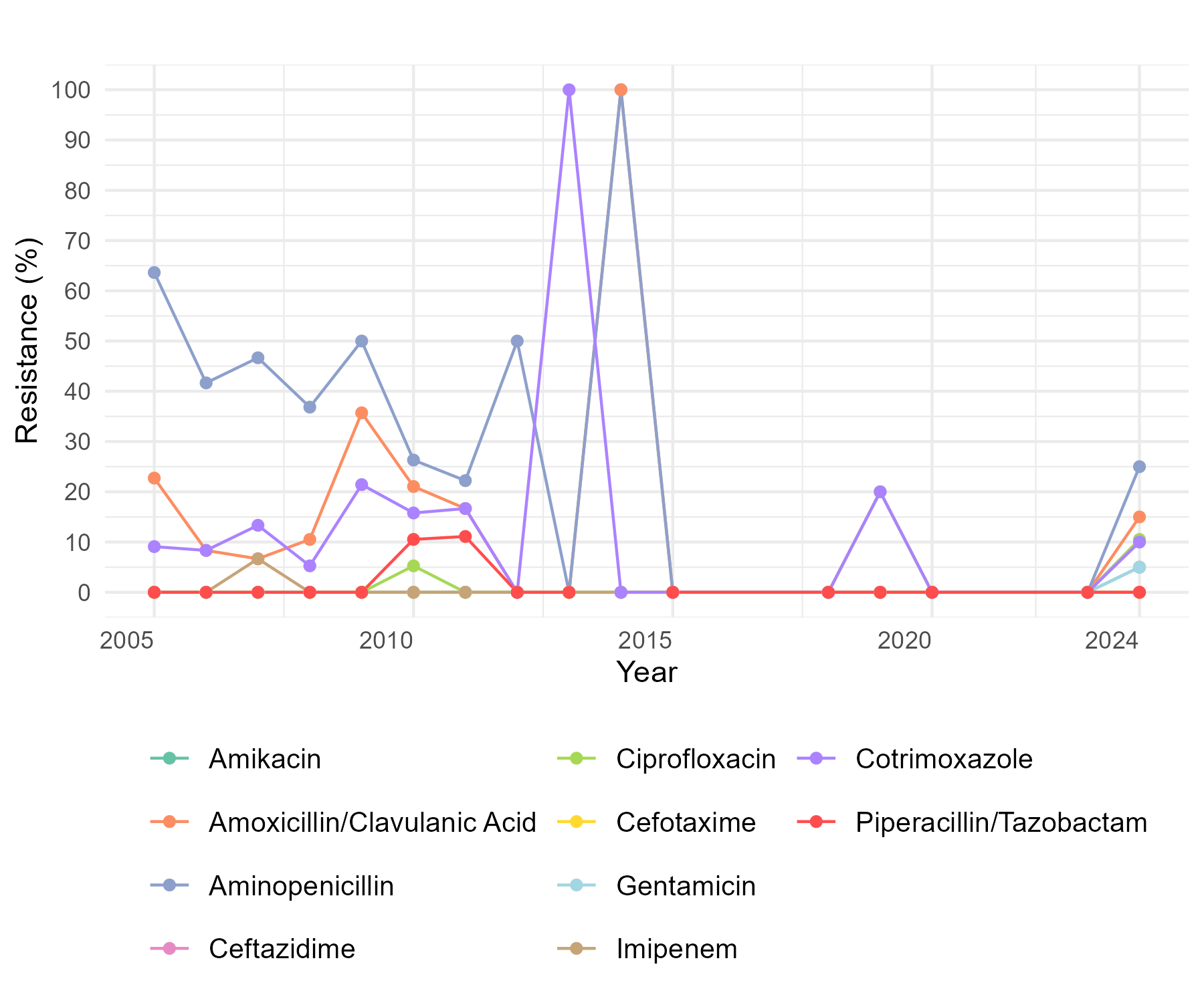


### Table S93: Logistic regression analysis of the effect of continuous time on the antibiotic resistance of *Shigella spp.* from 2005 to 2024.

| Antibiotic | β | OR | [95%CI] | P-value |  |
| --- | --- | --- | --- | --- | --- |
| Aminopenicillin | -0.100 | 0.90 | [0.84–0.97] | 0.006 | ** |
| Amoxicillin/Clavulanic Acid | -0.023 | 0.98 | [0.9–1.06] | 0.571 |  |
| Ceftazidime | 0.000 | 1.00 | [0.97–1.03] | 1.000 |  |
| Ciprofloxacin | 0.176 | 1.19 | [0.99–1.44] | 0.068 | . |
| Cefotaxime | 0.000 | 1.00 | [0.97–1.03] | 1.000 |  |
| Imipenem | -0.768 | 0.46 | [0.33–0.65] | 0.000 | *** |
| Cotrimoxazole | 0.000 | 1.00 | [0.92–1.09] | 0.993 |  |
| Piperacillin/Tazobactam | -0.015 | 0.99 | [0.82–1.19] | 0.878 |  |

### Table S94: Annual percentage of *Shigella spp.* resistance to Amikacin from 2005 to 2024.

|  | Tested | Resistance | | Logistic model | |  | |
| --- | --- | --- | --- | --- | --- | --- | --- |
| Year | N | % | [95% CI] | OR | [95% CI] | P-value |  |
| 2005 | 22 | 0 | [0–14.9] | – | – | – |  |
| 2006 | 12 | 0 | [0–24.2] | – | – | – |  |
| 2007 | 15 | 0 | [0–20.4] | – | – | – |  |
| 2008 | 19 | 0 | [0–16.8] | – | – | – |  |
| 2009 | 14 | 0 | [0–21.5] | – | – | – |  |
| 2010 | 19 | 0 | [0–16.8] | – | – | – |  |
| 2011 | 18 | 0 | [0–17.6] | – | – | – |  |
| 2012 | 2 | 0 | [0–65.8] | – | – | – |  |
| 2013 | 1 | 0 | [0–79.3] | – | – | – |  |
| 2014 | 1 | 0 | [0–79.3] | – | – | – |  |
| 2015 | 1 | 0 | [0–79.3] | – | – | – |  |
| 2018 | 2 | 0 | [0–65.8] | – | – | – |  |
| 2019 | 5 | 0 | [0–43.4] | – | – | – |  |
| 2020 | 2 | 0 | [0–65.8] | – | – | – |  |
| 2023 | 1 | 0 | [0–79.3] | – | – | – |  |
| 2024 | 20 | 5 | [0.9–23.6] | – | – | – |  |

### Table S95: Annual percentage of *Shigella spp.* resistance to Aminopenicillin from 2005 to 2024.

|  | Tested | Resistance | | Logistic model | |  | |
| --- | --- | --- | --- | --- | --- | --- | --- |
| Year | N | % | [95% CI] | OR | [95% CI] | P-value |  |
| 2005 | 22 | 63.6 | [43–80.3] | – | – | – |  |
| 2006 | 12 | 41.7 | [19.3–68] | 0.41 | [0.09–1.81] | 1.000 |  |
| 2007 | 15 | 46.7 | [24.8–69.9] | 1.22 | [0.26–5.81] | 1.000 |  |
| 2008 | 19 | 36.8 | [19.1–59] | 0.68 | [0.17–2.77] | 1.000 |  |
| 2009 | 14 | 50.0 | [26.8–73.2] | 1.54 | [0.36–6.53] | 1.000 |  |
| 2010 | 19 | 26.3 | [11.8–48.8] | 0.37 | [0.08–1.66] | 1.000 |  |
| 2011 | 18 | 22.2 | [9–45.2] | 0.73 | [0.15–3.44] | 1.000 |  |
| 2012 | 2 | 50.0 | [9.5–90.5] | 4.01 | [0.19–84.86] | 1.000 |  |
| 2013 | 1 | 0.0 | [0–79.3] | – | – | – |  |
| 2014 | 1 | 100.0 | [20.7–100] | – | – | – |  |
| 2015 | 1 | 0.0 | [0–79.3] | – | – | – |  |
| 2018 | 2 | 0.0 | [0–65.8] | – | – | – |  |
| 2019 | 5 | 0.0 | [0–43.4] | – | – | – |  |
| 2020 | 2 | 0.0 | [0–65.8] | – | – | – |  |
| 2023 | 1 | 0.0 | [0–79.3] | – | – | – |  |
| 2024 | 20 | 25.0 | [11.2–46.9] | – | – | – |  |

### Table S96: Annual percentage of *Shigella spp.* resistance to Amoxicillin/Clavulanic Acid from 2005 to 2024.

|  | Tested | Resistance | | Logistic model | |  | |
| --- | --- | --- | --- | --- | --- | --- | --- |
| Year | N | % | [95% CI] | OR | [95% CI] | P-value |  |
| 2005 | 22 | 22.7 | [10.1–43.4] | – | – | – |  |
| 2006 | 12 | 8.3 | [1.5–35.4] | 0.29 | [0.03–2.89] | 1.000 |  |
| 2007 | 15 | 6.7 | [1.2–29.8] | 0.78 | [0.04–14.15] | 1.000 |  |
| 2008 | 19 | 10.5 | [2.9–31.4] | 1.58 | [0.13–19.59] | 1.000 |  |
| 2009 | 14 | 35.7 | [16.3–61.2] | 5.11 | [0.79–32.92] | 1.000 |  |
| 2010 | 19 | 21.1 | [8.5–43.3] | 0.46 | [0.09–2.2] | 1.000 |  |
| 2011 | 18 | 16.7 | [5.8–39.2] | 0.81 | [0.15–4.41] | 1.000 |  |
| 2012 | 2 | 0.0 | [0–65.8] | – | – | – |  |
| 2013 | 1 | 0.0 | [0–79.3] | – | – | – |  |
| 2014 | 1 | 100.0 | [20.7–100] | – | – | – |  |
| 2015 | 1 | 0.0 | [0–79.3] | – | – | – |  |
| 2018 | 2 | 0.0 | [0–65.8] | – | – | – |  |
| 2019 | 5 | 0.0 | [0–43.4] | – | – | – |  |
| 2020 | 2 | 0.0 | [0–65.8] | – | – | – |  |
| 2023 | 1 | 0.0 | [0–79.3] | – | – | – |  |
| 2024 | 20 | 15.0 | [5.2–36] | – | – | – |  |

### Table S97: Annual percentage of *Shigella spp.* resistance to Ceftazidime from 2005 to 2024.

|  | Tested | Resistance | | Logistic model | |  | |
| --- | --- | --- | --- | --- | --- | --- | --- |
| Year | N | % | [95% CI] | OR | [95% CI] | P-value |  |
| 2005 | 22 | 0 | [0–14.9] | – | – | – |  |
| 2006 | 12 | 0 | [0–24.2] | 1 | [0.47–2.12] | 1.000 |  |
| 2007 | 15 | 0 | [0–20.4] | 1 | [0.45–2.24] | 1.000 |  |
| 2008 | 19 | 0 | [0–16.8] | 1 | [0.49–2.06] | 1.000 |  |
| 2009 | 14 | 0 | [0–21.5] | 1 | [0.48–2.1] | 1.000 |  |
| 2010 | 19 | 0 | [0–16.8] | 1 | [0.48–2.09] | 1.000 |  |
| 2011 | 18 | 0 | [0–17.6] | 1 | [0.5–2] | 1.000 |  |
| 2012 | 2 | 0 | [0–65.8] | 1 | [0.21–4.75] | 1.000 |  |
| 2013 | 1 | 0 | [0–79.3] | 1 | [0.08–13.03] | 1.000 |  |
| 2014 | 1 | 0 | [0–79.3] | 1 | [0.05–19.66] | 1.000 |  |
| 2015 | 1 | 0 | [0–79.3] | 1 | [0.05–19.13] | 1.000 |  |
| 2018 | 2 | 0 | [0–65.8] | 1 | [0.08–12.94] | 1.000 |  |
| 2019 | 5 | 0 | [0–43.4] | 1 | [0.17–5.94] | 1.000 |  |
| 2020 | 2 | 0 | [0–65.8] | 1 | [0.17–5.76] | 1.000 |  |
| 2023 | 1 | 0 | [0–79.3] | 1 | [0.08–12.96] | 1.000 |  |
| 2024 | 20 | 0 | [0–16.1] | 1 | [0.12–8.56] | 1.000 |  |

### Table S98: Annual percentage of *Shigella spp.* resistance to Ciprofloxacin from 2005 to 2024.

|  | Tested | Resistance | | Logistic model | |  | |
| --- | --- | --- | --- | --- | --- | --- | --- |
| Year | N | % | [95% CI] | OR | [95% CI] | P-value |  |
| 2005 | 22 | 0.0 | [0–14.9] | – | – | – |  |
| 2006 | 12 | 0.0 | [0–24.2] | – | – | – |  |
| 2007 | 15 | 0.0 | [0–20.4] | – | – | – |  |
| 2008 | 19 | 0.0 | [0–16.8] | – | – | – |  |
| 2009 | 14 | 0.0 | [0–21.5] | – | – | – |  |
| 2010 | 19 | 5.3 | [0.9–24.6] | – | – | – |  |
| 2011 | 18 | 0.0 | [0–17.6] | – | – | – |  |
| 2012 | 2 | 0.0 | [0–65.8] | – | – | – |  |
| 2013 | 1 | 0.0 | [0–79.3] | – | – | – |  |
| 2014 | 1 | 0.0 | [0–79.3] | – | – | – |  |
| 2015 | 1 | 0.0 | [0–79.3] | – | – | – |  |
| 2018 | 2 | 0.0 | [0–65.8] | – | – | – |  |
| 2019 | 5 | 20.0 | [3.6–62.4] | – | – | – |  |
| 2020 | 2 | 0.0 | [0–65.8] | – | – | – |  |
| 2023 | 1 | 0.0 | [0–79.3] | – | – | – |  |
| 2024 | 19 | 10.5 | [2.9–31.4] | – | – | – |  |

### Table S99: Annual percentage of *Shigella spp.* resistance to Cefotaxime from 2005 to 2024.

|  | Tested | Resistance | | Logistic model | |  | |
| --- | --- | --- | --- | --- | --- | --- | --- |
| Year | N | % | [95% CI] | OR | [95% CI] | P-value |  |
| 2005 | 22 | 0 | [0–14.9] | – | – | – |  |
| 2006 | 12 | 0 | [0–24.2] | 1 | [0.47–2.12] | 1.000 |  |
| 2007 | 15 | 0 | [0–20.4] | 1 | [0.45–2.24] | 1.000 |  |
| 2008 | 19 | 0 | [0–16.8] | 1 | [0.49–2.06] | 1.000 |  |
| 2009 | 14 | 0 | [0–21.5] | 1 | [0.48–2.1] | 1.000 |  |
| 2010 | 19 | 0 | [0–16.8] | 1 | [0.48–2.09] | 1.000 |  |
| 2011 | 18 | 0 | [0–17.6] | 1 | [0.5–2] | 1.000 |  |
| 2012 | 2 | 0 | [0–65.8] | 1 | [0.21–4.75] | 1.000 |  |
| 2013 | 1 | 0 | [0–79.3] | 1 | [0.08–13.03] | 1.000 |  |
| 2014 | 1 | 0 | [0–79.3] | 1 | [0.05–19.66] | 1.000 |  |
| 2015 | 1 | 0 | [0–79.3] | 1 | [0.05–19.13] | 1.000 |  |
| 2018 | 2 | 0 | [0–65.8] | 1 | [0.08–12.94] | 1.000 |  |
| 2019 | 5 | 0 | [0–43.4] | 1 | [0.17–5.94] | 1.000 |  |
| 2020 | 2 | 0 | [0–65.8] | 1 | [0.17–5.76] | 1.000 |  |
| 2023 | 1 | 0 | [0–79.3] | 1 | [0.08–12.96] | 1.000 |  |
| 2024 | 20 | 0 | [0–16.1] | 1 | [0.12–8.56] | 1.000 |  |

### Table S100: Annual percentage of *Shigella spp.* resistance to Gentamicin from 2005 to 2024.

|  | Tested | Resistance | | Logistic model | |  | |
| --- | --- | --- | --- | --- | --- | --- | --- |
| Year | N | % | [95% CI] | OR | [95% CI] | P-value |  |
| 2005 | 22 | 0 | [0–14.9] | – | – | – |  |
| 2006 | 12 | 0 | [0–24.2] | – | – | – |  |
| 2007 | 15 | 0 | [0–20.4] | – | – | – |  |
| 2008 | 19 | 0 | [0–16.8] | – | – | – |  |
| 2009 | 14 | 0 | [0–21.5] | – | – | – |  |
| 2010 | 19 | 0 | [0–16.8] | – | – | – |  |
| 2011 | 18 | 0 | [0–17.6] | – | – | – |  |
| 2012 | 2 | 0 | [0–65.8] | – | – | – |  |
| 2013 | 1 | 0 | [0–79.3] | – | – | – |  |
| 2014 | 1 | 0 | [0–79.3] | – | – | – |  |
| 2015 | 1 | 0 | [0–79.3] | – | – | – |  |
| 2018 | 2 | 0 | [0–65.8] | – | – | – |  |
| 2019 | 5 | 0 | [0–43.4] | – | – | – |  |
| 2020 | 2 | 0 | [0–65.8] | – | – | – |  |
| 2023 | 1 | 0 | [0–79.3] | – | – | – |  |
| 2024 | 20 | 5 | [0.9–23.6] | – | – | – |  |

### Table S101: Annual percentage of *Shigella spp.* resistance to Imipenem from 2005 to 2024.

|  | Tested | Resistance | | Logistic model | |  | |
| --- | --- | --- | --- | --- | --- | --- | --- |
| Year | N | % | [95% CI] | OR | [95% CI] | P-value |  |
| 2005 | 22 | 9.1 | [2.5–27.8] | – | – | – |  |
| 2006 | 12 | 8.3 | [1.5–35.4] | 1.06 | [0.08–14.67] | 1.000 |  |
| 2007 | 15 | 13.3 | [3.7–37.9] | 1.79 | [0.13–25.21] | 1.000 |  |
| 2008 | 19 | 5.3 | [0.9–24.6] | 0.39 | [0.03–5.4] | 1.000 |  |
| 2009 | 14 | 21.4 | [7.6–47.6] | 4.69 | [0.38–57.32] | 1.000 |  |
| 2010 | 19 | 15.8 | [5.5–37.6] | 0.74 | [0.11–4.83] | 1.000 |  |
| 2011 | 18 | 16.7 | [5.8–39.2] | 0.93 | [0.15–5.97] | 1.000 |  |
| 2012 | 2 | 0.0 | [0–65.8] | – | – | – |  |
| 2013 | 1 | 100.0 | [20.7–100] | – | – | – |  |
| 2014 | 1 | 0.0 | [0–79.3] | – | – | – |  |
| 2015 | 1 | 0.0 | [0–79.3] | – | – | – |  |
| 2018 | 2 | 0.0 | [0–65.8] | – | – | – |  |
| 2019 | 5 | 20.0 | [3.6–62.4] | – | – | – |  |
| 2020 | 2 | 0.0 | [0–65.8] | – | – | – |  |
| 2023 | 1 | 0.0 | [0–79.3] | – | – | – |  |
| 2024 | 20 | 10.0 | [2.8–30.1] | – | – | – |  |

### Table S102: Annual percentage of *Shigella spp.* resistance to Imipenem from 2005 to 2024.

|  | Tested | Resistance | | Logistic model | |  | |
| --- | --- | --- | --- | --- | --- | --- | --- |
| Year | N | % | [95% CI] | OR | [95% CI] | P-value |  |
| 2005 | 22 | 0.0 | [0–14.9] | – | – | – |  |
| 2006 | 12 | 0.0 | [0–24.2] | – | – | – | – |
| 2007 | 15 | 6.7 | [1.2–29.8] | – | – | – | – |
| 2008 | 19 | 0.0 | [0–16.8] | 0 | [0–0] | 0.000 | *** |
| 2009 | 14 | 0.0 | [0–21.5] | 0 | [0–0] | 0.000 | *** |
| 2010 | 19 | 0.0 | [0–16.8] | 0.07 | [0–39.57] | 0.440 |  |
| 2011 | 18 | 0.0 | [0–17.6] | 0.12 | [0–44.73] | 0.488 |  |
| 2012 | 2 | 0.0 | [0–65.8] | – | – | – | – |
| 2013 | 1 | 0.0 | [0–79.3] | 0 | [0–0] | 0.000 | *** |
| 2014 | 1 | 0.0 | [0–79.3] | – | – | – | – |
| 2015 | 1 | 0.0 | [0–79.3] | – | – | – | – |
| 2018 | 2 | 0.0 | [0–65.8] | 0 | [0–0] | 0.002 | ** |
| 2019 | 5 | 0.0 | [0–43.4] | 0 | [0–0] | 0.000 | *** |
| 2020 | 2 | 0.0 | [0–65.8] | – | – | – | – |
| 2023 | 1 | 0.0 | [0–79.3] | 0 | [0–0] | 0.001 | ** |
| 2024 | 19 | 0.0 | [0–16.8] | 0 | [0–0] | 0.000 | *** |

### Table S103: Annual percentage of *Shigella spp.* resistance to Cotrimoxazole from 2005 to 2024.

|  | Tested | Resistance | | Logistic model | |  | |
| --- | --- | --- | --- | --- | --- | --- | --- |
| Year | N | % | [95% CI] | OR | [95% CI] | P-value |  |
| 2005 | 22 | 0.0 | [0–14.9] | – | – | – |  |
| 2006 | 12 | 0.0 | [0–24.2] | – | – | – |  |
| 2007 | 15 | 0.0 | [0–20.4] | – | – | – |  |
| 2008 | 19 | 0.0 | [0–16.8] | – | – | – |  |
| 2009 | 14 | 0.0 | [0–21.5] | – | – | – |  |
| 2010 | 19 | 10.5 | [2.9–31.4] | – | – | – |  |
| 2011 | 18 | 11.1 | [3.1–32.8] | 1.67 | [0.48–5.77] | 1.000 |  |
| 2012 | 2 | 0.0 | [0–65.8] | – | – | – |  |
| 2013 | 1 | 0.0 | [0–79.3] | – | – | – |  |
| 2014 | 0 | 0.0 | [NaN–NaN] | – | – | – |  |
| 2015 | 1 | 0.0 | [0–79.3] | – | – | – |  |
| 2018 | 2 | 0.0 | [0–65.8] | – | – | – |  |
| 2019 | 5 | 0.0 | [0–43.4] | – | – | – |  |
| 2020 | 2 | 0.0 | [0–65.8] | – | – | – |  |
| 2023 | 1 | 0.0 | [0–79.3] | – | – | – |  |
| 2024 | 20 | 0.0 | [0–16.1] | – | – | – |  |

### Figure S10: Annual percentage of *Serratia marcescens* resistance from 2005 to 2024.


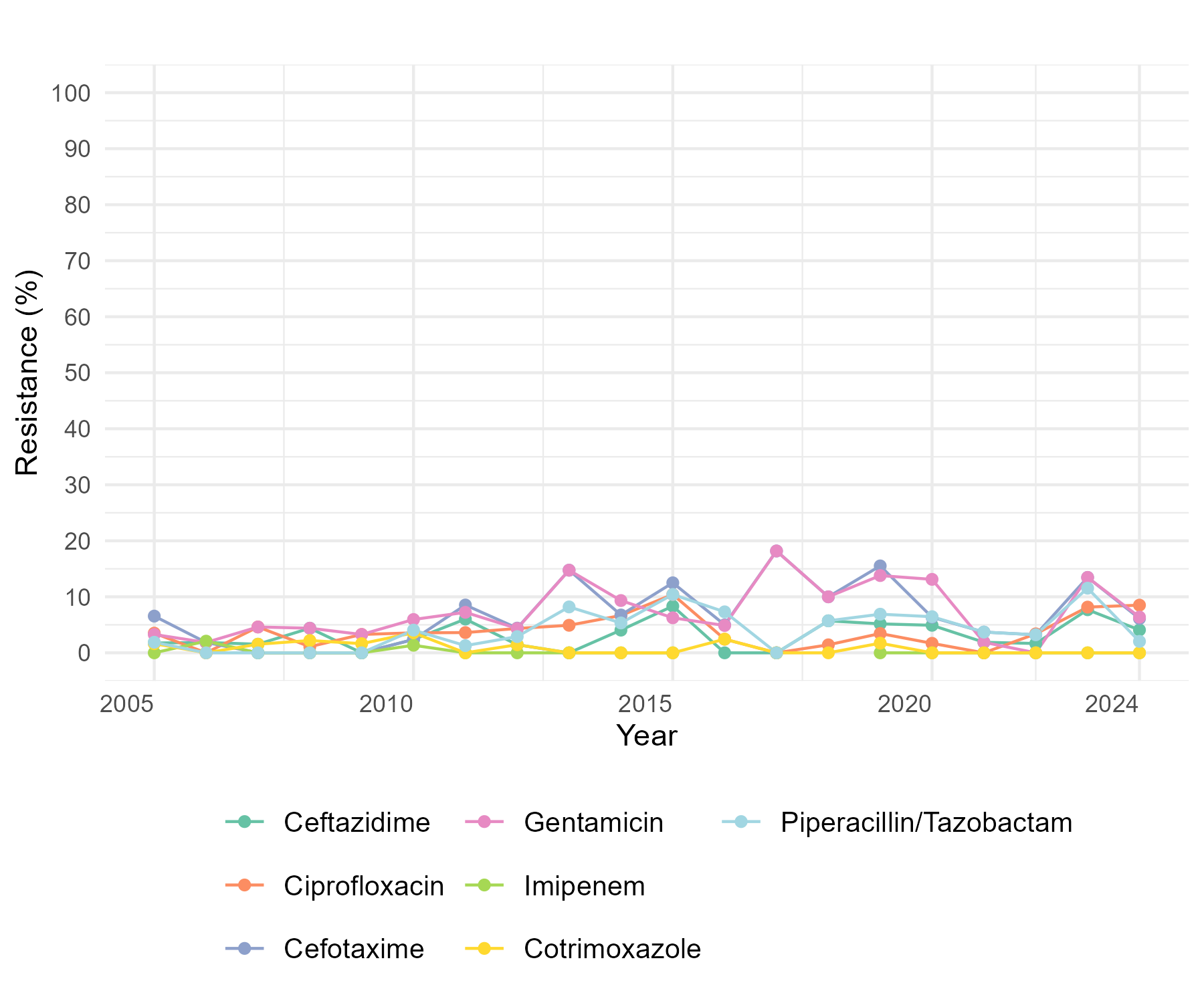


### Table S104: Logistic regression analysis of the effect of continuous time on the antibiotic resistance of *Serratia marcescens* from 2005 to 2024.

| Antibiotic | β | OR | [95%CI] | P-value |  |
| --- | --- | --- | --- | --- | --- |
| Ceftazidime | 0.043 | 1.04 | [0.99–1.1] | 0.141 |  |
| Ciprofloxacin | 0.024 | 1.02 | [0.97–1.08] | 0.374 |  |
| Cefotaxime | 0.067 | 1.07 | [1.03–1.11] | 0.001 | ** |
| Gentamicin | 0.043 | 1.04 | [1.01–1.08] | 0.026 | * |
| Imipenem | -0.109 | 0.90 | [0.65–1.24] | 0.511 |  |
| Cotrimoxazole | -0.112 | 0.89 | [0.79–1.01] | 0.070 | . |
| Piperacillin/Tazobactam | 0.087 | 1.09 | [1.03–1.15] | 0.002 | ** |

### Table S105: Annual percentage of *Serratia marcescens* resistance to Ceftazidime from 2005 to 2024.

|  | Tested | Resistance | | Logistic model | |  | |
| --- | --- | --- | --- | --- | --- | --- | --- |
| Year | N | % | [95% CI] | OR | [95% CI] | P-value |  |
| 2005 | 57 | 1.8 | [0.3–9.3] | – | – | – |  |
| 2006 | 54 | 1.9 | [0.3–9.8] | 1.01 | [0.08–13.6] | 1.000 |  |
| 2007 | 65 | 1.5 | [0.3–8.2] | 0.89 | [0.07–11.99] | 1.000 |  |
| 2008 | 91 | 4.4 | [1.7–10.8] | 3.05 | [0.39–23.87] | 1.000 |  |
| 2009 | 61 | 0.0 | [0–5.9] | – | – | – |  |
| 2010 | 84 | 2.4 | [0.7–8.3] | – | – | – |  |
| 2011 | 83 | 6.0 | [2.6–13.3] | 2.61 | [0.55–12.32] | 1.000 |  |
| 2012 | 69 | 1.4 | [0.3–7.8] | 0.21 | [0.03–1.59] | 1.000 |  |
| 2013 | 61 | 0.0 | [0–5.9] | – | – | – |  |
| 2014 | 74 | 4.1 | [1.4–11.3] | – | – | – |  |
| 2015 | 48 | 8.3 | [3.3–19.6] | 1.65 | [0.36–7.59] | 1.000 |  |
| 2016 | 41 | 0.0 | [0–8.6] | – | – | – |  |
| 2017 | 44 | 0.0 | [0–8] | – | – | – |  |
| 2018 | 70 | 5.7 | [2.2–13.8] | – | – | – |  |
| 2019 | 58 | 5.2 | [1.8–14.1] | 0.83 | [0.2–3.47] | 1.000 |  |
| 2020 | 61 | 4.9 | [1.7–13.5] | 0.98 | [0.21–4.53] | 1.000 |  |
| 2021 | 53 | 1.9 | [0.3–9.9] | 0.38 | [0.05–3.19] | 1.000 |  |
| 2022 | 60 | 1.7 | [0.3–8.9] | 0.88 | [0.07–11.77] | 1.000 |  |
| 2023 | 52 | 7.7 | [3–18.2] | 5.09 | [0.64–40.26] | 1.000 |  |
| 2024 | 49 | 4.1 | [1.1–13.7] | 0.48 | [0.09–2.42] | 1.000 |  |

### Table S106: Annual percentage of *Serratia marcescens* resistance to Ciprofloxacin from 2005 to 2024.

|  | Tested | Resistance | | Logistic model | |  | |
| --- | --- | --- | --- | --- | --- | --- | --- |
| Year | N | % | [95% CI] | OR | [95% CI] | P-value |  |
| 2005 | 57 | 3.5 | [1–11.9] | – | – | – |  |
| 2006 | 54 | 0.0 | [0–6.6] | – | – | – |  |
| 2007 | 65 | 4.6 | [1.6–12.7] | – | – | – |  |
| 2008 | 91 | 1.1 | [0.2–6] | 0.19 | [0.02–1.56] | 0.992 |  |
| 2009 | 61 | 3.3 | [0.9–11.2] | 3.33 | [0.35–31.59] | 0.992 |  |
| 2010 | 84 | 3.6 | [1.2–10] | 1.13 | [0.21–6.13] | 0.992 |  |
| 2011 | 83 | 3.6 | [1.2–10.1] | 0.98 | [0.22–4.45] | 0.992 |  |
| 2012 | 69 | 4.3 | [1.5–12] | 1.3 | [0.29–5.96] | 0.992 |  |
| 2013 | 61 | 4.9 | [1.7–13.5] | 1.22 | [0.26–5.59] | 0.992 |  |
| 2014 | 75 | 6.7 | [2.9–14.7] | 1.29 | [0.33–5.09] | 0.992 |  |
| 2015 | 48 | 10.4 | [4.5–22.2] | 1.37 | [0.38–4.91] | 0.992 |  |
| 2016 | 41 | 2.4 | [0.4–12.6] | 0.26 | [0.03–2.1] | 0.992 |  |
| 2017 | 44 | 0.0 | [0–8] | – | – | – |  |
| 2018 | 70 | 1.4 | [0.3–7.7] | – | – | – |  |
| 2019 | 58 | 3.4 | [1–11.7] | 2.35 | [0.25–22.44] | 0.992 |  |
| 2020 | 59 | 1.7 | [0.3–9] | 0.47 | [0.05–4.51] | 0.992 |  |
| 2021 | 52 | 0.0 | [0–6.9] | – | – | – |  |
| 2022 | 59 | 3.4 | [0.9–11.5] | – | – | – |  |
| 2023 | 49 | 8.2 | [3.2–19.2] | 2.24 | [0.44–11.35] | 0.992 |  |
| 2024 | 47 | 8.5 | [3.4–19.9] | 1.16 | [0.3–4.48] | 0.992 |  |

### Table S107: Annual percentage of *Serratia marcescens* resistance to Cefotaxime from 2005 to 2024.

|  | Tested | Resistance | | Logistic model | |  | |
| --- | --- | --- | --- | --- | --- | --- | --- |
| Year | N | % | [95% CI] | OR | [95% CI] | P-value |  |
| 2005 | 61 | 6.6 | [2.6–15.7] | – | – | – |  |
| 2006 | 54 | 1.9 | [0.3–9.8] | 0.28 | [0.04–2.19] | 0.477 |  |
| 2007 | 65 | 0.0 | [0–5.6] | – | – | – |  |
| 2008 | 91 | 0.0 | [0–4.1] | – | – | – |  |
| 2009 | 61 | 0.0 | [0–5.9] | – | – | – |  |
| 2010 | 84 | 2.4 | [0.7–8.3] | – | – | – |  |
| 2011 | 82 | 8.5 | [4.2–16.6] | 3.81 | [0.87–16.75] | 0.310 |  |
| 2012 | 68 | 4.4 | [1.5–12.2] | 0.51 | [0.14–1.86] | 0.510 |  |
| 2013 | 61 | 14.8 | [8–25.7] | 3.83 | [1.09–13.45] | 0.310 |  |
| 2014 | 74 | 6.8 | [2.9–14.9] | 0.41 | [0.14–1.18] | 0.310 |  |
| 2015 | 48 | 12.5 | [5.9–24.7] | 1.65 | [0.5–5.49] | 0.600 |  |
| 2016 | 40 | 5.0 | [1.4–16.5] | 0.45 | [0.09–2.14] | 0.510 |  |
| 2017 | 44 | 18.2 | [9.5–32] | 4.49 | [1.01–20.05] | 0.310 |  |
| 2018 | 70 | 10.0 | [4.9–19.2] | 0.47 | [0.17–1.3] | 0.391 |  |
| 2019 | 58 | 15.5 | [8.4–26.9] | 1.64 | [0.62–4.38] | 0.510 |  |
| 2020 | 63 | 6.3 | [2.5–15.2] | 0.37 | [0.12–1.15] | 0.310 |  |
| 2021 | 54 | 3.7 | [1–12.5] | 0.57 | [0.11–2.82] | 0.661 |  |
| 2022 | 62 | 3.2 | [0.9–11] | 0.83 | [0.13–5.26] | 1.000 |  |
| 2023 | 52 | 13.5 | [6.7–25.3] | 4.5 | [1.01–20.1] | 0.310 |  |
| 2024 | 49 | 6.1 | [2.1–16.5] | 0.45 | [0.12–1.65] | 0.477 |  |

### Table S108: Annual percentage of *Serratia marcescens* resistance to Gentamicin from 2005 to 2024.

|  | Tested | Resistance | | Logistic model | |  | |
| --- | --- | --- | --- | --- | --- | --- | --- |
| Year | N | % | [95% CI] | OR | [95% CI] | P-value |  |
| 2005 | 61 | 3.3 | [0.9–11.2] | – | – | – |  |
| 2006 | 54 | 1.9 | [0.3–9.8] | 0.58 | [0.05–6.4] | 0.979 |  |
| 2007 | 65 | 4.6 | [1.6–12.7] | 2.52 | [0.26–24.52] | 0.972 |  |
| 2008 | 91 | 4.4 | [1.7–10.8] | 0.9 | [0.2–4.11] | 0.979 |  |
| 2009 | 61 | 3.3 | [0.9–11.2] | 0.75 | [0.14–4.17] | 0.979 |  |
| 2010 | 84 | 6.0 | [2.6–13.2] | 1.88 | [0.36–9.91] | 0.972 |  |
| 2011 | 83 | 7.2 | [3.4–14.9] | 1.23 | [0.36–4.15] | 0.979 |  |
| 2012 | 69 | 4.3 | [1.5–12] | 0.59 | [0.14–2.43] | 0.972 |  |
| 2013 | 61 | 14.8 | [8–25.7] | 3.86 | [1.01–14.84] | 0.437 |  |
| 2014 | 75 | 9.3 | [4.6–18] | 0.58 | [0.21–1.66] | 0.972 |  |
| 2015 | 48 | 6.2 | [2.1–16.8] | 0.66 | [0.16–2.67] | 0.972 |  |
| 2016 | 41 | 4.9 | [1.3–16.1] | 0.76 | [0.12–4.74] | 0.979 |  |
| 2017 | 44 | 18.2 | [9.5–32] | 4.43 | [0.89–22] | 0.437 |  |
| 2018 | 70 | 10.0 | [4.9–19.2] | 0.49 | [0.17–1.47] | 0.968 |  |
| 2019 | 58 | 13.8 | [7.2–24.9] | 1.4 | [0.48–4.11] | 0.972 |  |
| 2020 | 61 | 13.1 | [6.8–23.8] | 0.94 | [0.33–2.67] | 0.979 |  |
| 2021 | 53 | 1.9 | [0.3–9.9] | 0.13 | [0.02–1.05] | 0.437 |  |
| 2022 | 60 | 0.0 | [0–6] | – | – | – |  |
| 2023 | 52 | 13.5 | [6.7–25.3] | – | – | – |  |
| 2024 | 47 | 6.4 | [2.2–17.2] | 0.45 | [0.11–1.84] | 0.972 |  |

### Table S109: Annual percentage of *Serratia marcescens* resistance to Imipenem from 2005 to 2024.

|  | Tested | Resistance | | Logistic model | |  | |
| --- | --- | --- | --- | --- | --- | --- | --- |
| Year | N | % | [95% CI] | OR | [95% CI] | P-value |  |
| 2005 | 53 | 0.0 | [0–6.8] | – | – | – |  |
| 2006 | 48 | 2.1 | [0.4–10.9] | – | – | – |  |
| 2007 | 59 | 0.0 | [0–6.1] | – | – | – |  |
| 2008 | 84 | 0.0 | [0–4.4] | – | – | – |  |
| 2009 | 54 | 0.0 | [0–6.6] | – | – | – |  |
| 2010 | 74 | 1.4 | [0.2–7.3] | – | – | – |  |
| 2011 | 78 | 0.0 | [0–4.7] | – | – | – |  |
| 2012 | 39 | 0.0 | [0–9] | – | – | – |  |
| 2013 | 6 | 0.0 | [0–39] | – | – | – |  |
| 2014 | 7 | 0.0 | [0–35.4] | – | – | – |  |
| 2015 | 1 | 0.0 | [0–79.3] | – | – | – |  |
| 2016 | 0 | 0.0 | [NaN–NaN] | – | – | – |  |
| 2017 | 1 | 0.0 | [0–79.3] | – | – | – |  |
| 2018 | 0 | 0.0 | [NaN–NaN] | – | – | – |  |
| 2019 | 6 | 0.0 | [0–39] | – | – | – |  |
| 2020 | 6 | 0.0 | [0–39] | – | – | – |  |
| 2021 | 5 | 0.0 | [0–43.4] | – | – | – |  |
| 2022 | 7 | 0.0 | [0–35.4] | – | – | – |  |
| 2023 | 7 | 0.0 | [0–35.4] | – | – | – |  |
| 2024 | 6 | 0.0 | [0–39] | – | – | – |  |

### Table S110: Annual percentage of *Serratia marcescens* resistance to Cotrimoxazole from 2005 to 2024.

|  | Tested | Resistance | | Logistic model | |  | |
| --- | --- | --- | --- | --- | --- | --- | --- |
| Year | N | % | [95% CI] | OR | [95% CI] | P-value |  |
| 2005 | 61 | 1.6 | [0.3–8.7] | – | – | – |  |
| 2006 | 54 | 0.0 | [0–6.6] | – | – | – |  |
| 2007 | 65 | 1.5 | [0.3–8.2] | – | – | – |  |
| 2008 | 91 | 2.2 | [0.6–7.7] | – | – | – |  |
| 2009 | 61 | 1.6 | [0.3–8.7] | 0.73 | [0.14–3.76] | 1.000 |  |
| 2010 | 84 | 3.6 | [1.2–10] | 2.41 | [0.51–11.37] | 1.000 |  |
| 2011 | 83 | 0.0 | [0–4.4] | – | – | – |  |
| 2012 | 69 | 1.4 | [0.3–7.8] | – | – | – |  |
| 2013 | 61 | 0.0 | [0–5.9] | – | – | – |  |
| 2014 | 75 | 0.0 | [0–4.9] | – | – | – |  |
| 2015 | 48 | 0.0 | [0–7.4] | – | – | – |  |
| 2016 | 41 | 2.4 | [0.4–12.6] | – | – | – |  |
| 2017 | 44 | 0.0 | [0–8] | – | – | – |  |
| 2018 | 70 | 0.0 | [0–5.2] | – | – | – |  |
| 2019 | 58 | 1.7 | [0.3–9.1] | – | – | – |  |
| 2020 | 62 | 0.0 | [0–5.8] | – | – | – |  |
| 2021 | 53 | 0.0 | [0–6.8] | – | – | – |  |
| 2022 | 63 | 0.0 | [0–5.7] | – | – | – |  |
| 2023 | 52 | 0.0 | [0–6.9] | – | – | – |  |
| 2024 | 49 | 0.0 | [0–7.3] | – | – | – |  |

### Table 111: Annual percentage of *Serratia marcescens* resistance to Piperacillin/Tazobactam from 2005 to 2024.

|  | Tested | Resistance | | Logistic model | |  | |
| --- | --- | --- | --- | --- | --- | --- | --- |
| Year | N | % | [95% CI] | OR | [95% CI] | P-value |  |
| 2005 | 53 | 1.9 | [0.3–9.9] | – | – | – |  |
| 2006 | 48 | 0.0 | [0–7.4] | – | – | – |  |
| 2007 | 59 | 0.0 | [0–6.1] | – | – | – |  |
| 2008 | 84 | 0.0 | [0–4.4] | – | – | – |  |
| 2009 | 54 | 0.0 | [0–6.6] | – | – | – |  |
| 2010 | 74 | 4.1 | [1.4–11.3] | – | – | – |  |
| 2011 | 78 | 1.3 | [0.2–6.9] | 0.31 | [0.04–2.3] | 1.000 |  |
| 2012 | 69 | 2.9 | [0.8–10] | 2.32 | [0.27–19.55] | 1.000 |  |
| 2013 | 61 | 8.2 | [3.6–17.8] | 3.01 | [0.69–13.21] | 0.907 |  |
| 2014 | 75 | 5.3 | [2.1–12.9] | 0.62 | [0.19–2.07] | 1.000 |  |
| 2015 | 48 | 10.4 | [4.5–22.2] | 1.65 | [0.47–5.85] | 1.000 |  |
| 2016 | 41 | 7.3 | [2.5–19.4] | 0.86 | [0.22–3.39] | 1.000 |  |
| 2017 | 44 | 0.0 | [0–8] | – | – | – |  |
| 2018 | 70 | 5.7 | [2.2–13.8] | – | – | – |  |
| 2019 | 58 | 6.9 | [2.7–16.4] | 1.2 | [0.34–4.26] | 1.000 |  |
| 2020 | 62 | 6.5 | [2.5–15.4] | 0.93 | [0.26–3.3] | 1.000 |  |
| 2021 | 54 | 3.7 | [1–12.5] | 0.56 | [0.12–2.59] | 1.000 |  |
| 2022 | 62 | 3.2 | [0.9–11] | 0.85 | [0.15–4.95] | 1.000 |  |
| 2023 | 52 | 11.5 | [5.4–23] | 3.84 | [0.9–16.38] | 0.655 |  |
| 2024 | 49 | 2.0 | [0.4–10.7] | 0.16 | [0.02–1.09] | 0.655 |  |

## Enterococci

### Figure S11: Annual percentage of *Enterococcus faecium* resistance from 2005 to 2024.


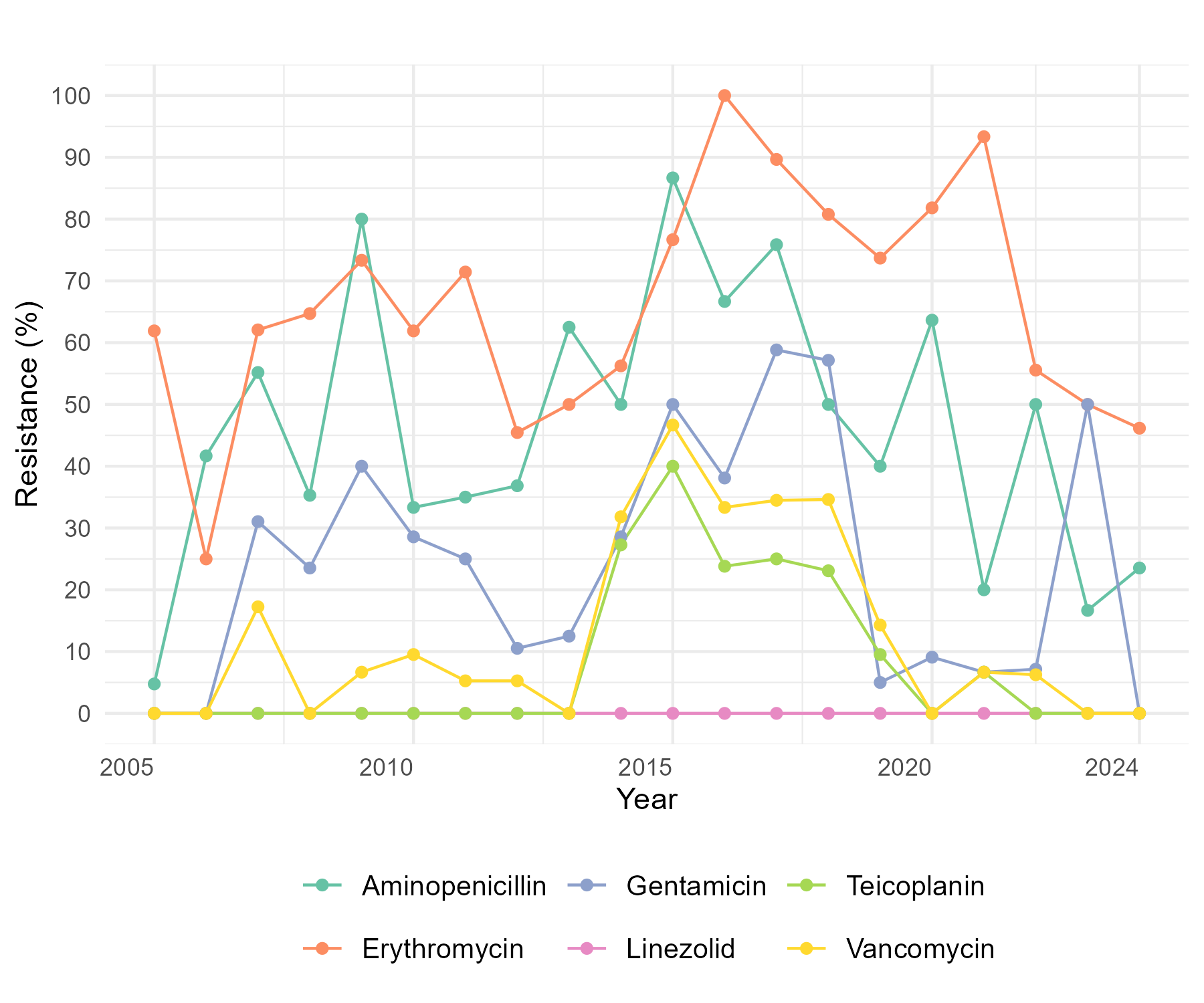


### Table S112: Logistic regression analysis of the effect of continuous time on the antibiotic resistance of *Enterococcus faecium* from 2005 to 2024.

| Antibiotic | β | OR | [95%CI] | P-value |  |
| --- | --- | --- | --- | --- | --- |
| Aminopenicillin | 0.000 | 1.00 | [0.96–1.04] | 0.988 |  |
| Erythromycin | 0.059 | 1.06 | [1.01–1.11] | 0.012 | * |
| Gentamicin | -0.002 | 1.00 | [0.95–1.05] | 0.929 |  |
| Linezolid | 0.000 | 1.00 | [0.98–1.02] | 1.000 |  |
| Teicoplanin | 0.057 | 1.06 | [1–1.13] | 0.071 | . |
| Vancomycin | 0.018 | 1.02 | [0.97–1.07] | 0.480 |  |

### Table S113: Annual percentage of *Enterococcus faecium* resistance to Aminopenicillin from 2005 to 2024.

|  | Tested | Resistance | | Logistic model | |  | |
| --- | --- | --- | --- | --- | --- | --- | --- |
| Year | N | % | [95% CI] | OR | [95% CI] | P-value |  |
| 2005 | 21 | 4.8 | [0.8–22.7] | – | – | – |  |
| 2006 | 12 | 41.7 | [19.3–68] | 14.44 | [1.32–157.46] | 0.126 |  |
| 2007 | 29 | 55.2 | [37.5–71.6] | 1.65 | [0.4–6.81] | 0.627 |  |
| 2008 | 17 | 35.3 | [17.3–58.7] | 0.48 | [0.13–1.74] | 0.415 |  |
| 2009 | 15 | 80.0 | [54.8–93] | 6.93 | [1.3–36.95] | 0.126 |  |
| 2010 | 21 | 33.3 | [17.2–54.6] | 0.12 | [0.02–0.59] | 0.093 | . |
| 2011 | 20 | 35.0 | [18.1–56.7] | 1.06 | [0.28–4.07] | 0.928 |  |
| 2012 | 19 | 36.8 | [19.1–59] | 1.07 | [0.28–4.14] | 0.928 |  |
| 2013 | 8 | 62.5 | [30.6–86.3] | 3.34 | [0.56–19.82] | 0.351 |  |
| 2014 | 22 | 50.0 | [30.7–69.3] | 0.56 | [0.1–3.1] | 0.627 |  |
| 2015 | 30 | 86.7 | [70.3–94.7] | 6.22 | [1.55–24.91] | 0.093 | . |
| 2016 | 21 | 66.7 | [45.4–82.8] | 0.31 | [0.07–1.3] | 0.255 |  |
| 2017 | 29 | 75.9 | [57.9–87.8] | 1.51 | [0.42–5.48] | 0.627 |  |
| 2018 | 26 | 50.0 | [32.1–67.9] | 0.33 | [0.1–1.07] | 0.174 |  |
| 2019 | 20 | 40.0 | [21.9–61.3] | 0.66 | [0.19–2.22] | 0.627 |  |
| 2020 | 11 | 63.6 | [35.4–84.8] | 2.72 | [0.57–13.11] | 0.366 |  |
| 2021 | 15 | 20.0 | [7–45.2] | 0.14 | [0.02–0.85] | 0.126 |  |
| 2022 | 16 | 50.0 | [28–72] | 3.71 | [0.71–19.37] | 0.255 |  |
| 2023 | 18 | 16.7 | [5.8–39.2] | 0.21 | [0.04–1.05] | 0.174 |  |
| 2024 | 17 | 23.5 | [9.6–47.3] | 1.6 | [0.29–8.99] | 0.660 |  |

### Table S114: Annual percentage of *Enterococcus faecium* resistance to Erythromycin from 2005 to 2024.

|  | Tested | Resistance | | Logistic model | |  | |
| --- | --- | --- | --- | --- | --- | --- | --- |
| Year | N | % | [95% CI] | OR | [95% CI] | P-value |  |
| 2005 | 21 | 61.9 | [40.9–79.2] | – | – | – |  |
| 2006 | 12 | 25.0 | [8.9–53.2] | 0.2 | [0.04–0.98] | 0.340 |  |
| 2007 | 29 | 62.1 | [44–77.3] | 5.16 | [1.12–23.83] | 0.340 |  |
| 2008 | 17 | 64.7 | [41.3–82.7] | 1.01 | [0.28–3.6] | 0.989 |  |
| 2009 | 15 | 73.3 | [48–89.1] | 1.64 | [0.35–7.63] | 0.989 |  |
| 2010 | 21 | 61.9 | [40.9–79.2] | 0.59 | [0.14–2.56] | 0.989 |  |
| 2011 | 14 | 71.4 | [45.4–88.3] | 1.59 | [0.37–6.91] | 0.989 |  |
| 2012 | 11 | 45.5 | [21.3–72] | 0.33 | [0.06–1.73] | 0.718 |  |
| 2013 | 4 | 50.0 | [15–85] | 1.13 | [0.11–11.52] | 0.989 |  |
| 2014 | 16 | 56.2 | [33.2–76.9] | 1.37 | [0.15–12.56] | 0.989 |  |
| 2015 | 30 | 76.7 | [59.1–88.2] | 2.58 | [0.7–9.53] | 0.718 |  |
| 2016 | 21 | 100.0 | [84.5–100] | – | – | – |  |
| 2017 | 29 | 89.7 | [73.6–96.4] | – | – | – |  |
| 2018 | 26 | 80.8 | [62.1–91.5] | 0.47 | [0.1–2.23] | 0.989 |  |
| 2019 | 19 | 73.7 | [51.2–88.2] | 0.67 | [0.16–2.77] | 0.989 |  |
| 2020 | 11 | 81.8 | [52.3–94.9] | 1.58 | [0.25–10.04] | 0.989 |  |
| 2021 | 15 | 93.3 | [70.2–98.8] | 3.2 | [0.25–41.09] | 0.989 |  |
| 2022 | 9 | 55.6 | [26.7–81.1] | 0.09 | [0.01–1.04] | 0.340 |  |
| 2023 | 4 | 50.0 | [15–85] | 0.78 | [0.07–8.42] | 0.989 |  |
| 2024 | 13 | 46.2 | [23.2–70.9] | 0.85 | [0.09–8.09] | 0.989 |  |

### Table S115: Annual percentage of *Enterococcus faecium* resistance to Gentamicin from 2005 to 2024.

|  | Tested | Resistance | | Logistic model | |  | |
| --- | --- | --- | --- | --- | --- | --- | --- |
| Year | N | % | [95% CI] | OR | [95% CI] | P-value |  |
| 2005 | 21 | 0.0 | [0–15.5] | – | – | – |  |
| 2006 | 12 | 0.0 | [0–24.2] | – | – | – |  |
| 2007 | 29 | 31.0 | [17.3–49.2] | – | – | – |  |
| 2008 | 17 | 23.5 | [9.6–47.3] | 0.66 | [0.17–2.64] | 0.967 |  |
| 2009 | 15 | 40.0 | [19.8–64.3] | 2.33 | [0.5–10.87] | 0.764 |  |
| 2010 | 21 | 28.6 | [13.8–50] | 0.44 | [0.11–1.85] | 0.764 |  |
| 2011 | 20 | 25.0 | [11.2–46.9] | 0.78 | [0.19–3.15] | 1.000 |  |
| 2012 | 19 | 10.5 | [2.9–31.4] | 0.33 | [0.06–1.9] | 0.764 |  |
| 2013 | 8 | 12.5 | [2.2–47.1] | 2.28 | [0.18–28.44] | 0.967 |  |
| 2014 | 21 | 28.6 | [13.8–50] | 2.22 | [0.23–21.45] | 0.967 |  |
| 2015 | 30 | 50.0 | [33.2–66.8] | 2.15 | [0.65–7.13] | 0.764 |  |
| 2016 | 21 | 38.1 | [20.8–59.1] | 0.63 | [0.2–1.97] | 0.967 |  |
| 2017 | 17 | 58.8 | [36–78.4] | 2.14 | [0.57–7.96] | 0.764 |  |
| 2018 | 14 | 57.1 | [32.6–78.6] | 0.95 | [0.23–4.04] | 1.000 |  |
| 2019 | 20 | 5.0 | [0.9–23.6] | 0.04 | [0–0.32] | 0.059 | . |
| 2020 | 11 | 9.1 | [1.6–37.7] | 1.81 | [0.11–30.27] | 1.000 |  |
| 2021 | 15 | 6.7 | [1.2–29.8] | 0.74 | [0.04–12.6] | 1.000 |  |
| 2022 | 14 | 7.1 | [1.3–31.5] | 0.77 | [0.05–12.45] | 1.000 |  |
| 2023 | 2 | 50.0 | [9.5–90.5] | 23.22 | [0.81–669.28] | 0.633 |  |
| 2024 | 4 | 0.0 | [0–49] | – | – | – |  |

### Table S116: Annual percentage of *Enterococcus faecium* resistance to Linezolid from 2005 to 2024.

|  | Tested | Resistance | | Logistic model | |  | |
| --- | --- | --- | --- | --- | --- | --- | --- |
| Year | N | % | [95% CI] | OR | [95% CI] | P-value |  |
| 2005 | 3 | 0 | [0–56.1] | – | – | – |  |
| 2006 | 12 | 0 | [0–24.2] | 1 | [0.27–3.71] | 1.000 |  |
| 2007 | 29 | 0 | [0–11.7] | 1 | [0.5–2.02] | 1.000 |  |
| 2008 | 17 | 0 | [0–18.4] | 1 | [0.53–1.88] | 1.000 |  |
| 2009 | 15 | 0 | [0–20.4] | 1 | [0.48–2.07] | 1.000 |  |
| 2010 | 21 | 0 | [0–15.5] | 1 | [0.5–1.99] | 1.000 |  |
| 2011 | 20 | 0 | [0–16.1] | 1 | [0.53–1.89] | 1.000 |  |
| 2012 | 19 | 0 | [0–16.8] | 1 | [0.52–1.91] | 1.000 |  |
| 2013 | 8 | 0 | [0–32.4] | 1 | [0.42–2.36] | 1.000 |  |
| 2014 | 22 | 0 | [0–14.9] | 1 | [0.43–2.31] | 1.000 |  |
| 2015 | 30 | 0 | [0–11.4] | 1 | [0.57–1.77] | 1.000 |  |
| 2016 | 21 | 0 | [0–15.5] | 1 | [0.56–1.78] | 1.000 |  |
| 2017 | 29 | 0 | [0–11.7] | 1 | [0.56–1.79] | 1.000 |  |
| 2018 | 26 | 0 | [0–12.9] | 1 | [0.58–1.73] | 1.000 |  |
| 2019 | 19 | 0 | [0–16.8] | 1 | [0.54–1.84] | 1.000 |  |
| 2020 | 11 | 0 | [0–25.9] | 1 | [0.46–2.15] | 1.000 |  |
| 2021 | 15 | 0 | [0–20.4] | 1 | [0.45–2.24] | 1.000 |  |
| 2022 | 16 | 0 | [0–19.4] | 1 | [0.48–2.07] | 1.000 |  |
| 2023 | 16 | 0 | [0–19.4] | 1 | [0.49–2.04] | 1.000 |  |
| 2024 | 17 | 0 | [0–18.4] | 1 | [0.49–2.02] | 1.000 |  |

### Table S117: Annual percentage of *Enterococcus faecium* resistance to Teicoplanin from 2005 to 2024.

|  | Tested | Resistance | | Logistic model | |  | |
| --- | --- | --- | --- | --- | --- | --- | --- |
| Year | N | % | [95% CI] | OR | [95% CI] | P-value |  |
| 2005 | 21 | 0.0 | [0–15.5] | – | – | – |  |
| 2006 | 12 | 0.0 | [0–24.2] | – | – | – |  |
| 2007 | 29 | 0.0 | [0–11.7] | – | – | – |  |
| 2008 | 17 | 0.0 | [0–18.4] | – | – | – |  |
| 2009 | 15 | 0.0 | [0–20.4] | – | – | – |  |
| 2010 | 21 | 0.0 | [0–15.5] | – | – | – |  |
| 2011 | 20 | 0.0 | [0–16.1] | – | – | – |  |
| 2012 | 19 | 0.0 | [0–16.8] | – | – | – |  |
| 2013 | 8 | 0.0 | [0–32.4] | – | – | – |  |
| 2014 | 22 | 27.3 | [13.2–48.2] | – | – | – |  |
| 2015 | 30 | 40.0 | [24.6–57.7] | 1.63 | [0.73–3.67] | 1.000 |  |
| 2016 | 21 | 23.8 | [10.6–45.1] | 0.52 | [0.22–1.21] | 1.000 |  |
| 2017 | 28 | 25.0 | [12.7–43.4] | 0.91 | [0.37–2.25] | 1.000 |  |
| 2018 | 26 | 23.1 | [11–42.1] | 0.98 | [0.42–2.3] | 1.000 |  |
| 2019 | 21 | 9.5 | [2.7–28.9] | 0.34 | [0.11–1.08] | 1.000 |  |
| 2020 | 11 | 0.0 | [0–25.9] | – | – | – |  |
| 2021 | 15 | 6.7 | [1.2–29.8] | – | – | – |  |
| 2022 | 16 | 0.0 | [0–19.4] | – | – | – |  |
| 2023 | 18 | 0.0 | [0–17.6] | – | – | – |  |
| 2024 | 17 | 0.0 | [0–18.4] | – | – | – |  |

### Table S118: Annual percentage of *Enterococcus faecium* resistance to Vancomycin from 2005 to 2024.

|  | Tested | Resistance | | Logistic model | |  | |
| --- | --- | --- | --- | --- | --- | --- | --- |
| Year | N | % | [95% CI] | OR | [95% CI] | P-value |  |
| 2005 | 21 | 0.0 | [0–15.5] | – | – | – |  |
| 2006 | 12 | 0.0 | [0–24.2] | – | – | – |  |
| 2007 | 29 | 17.2 | [7.6–34.5] | – | – | – |  |
| 2008 | 17 | 0.0 | [0–18.4] | – | – | – |  |
| 2009 | 15 | 6.7 | [1.2–29.8] | – | – | – |  |
| 2010 | 21 | 9.5 | [2.7–28.9] | 1.49 | [0.17–13.39] | 1.000 |  |
| 2011 | 19 | 5.3 | [0.9–24.6] | 0.47 | [0.05–4.17] | 1.000 |  |
| 2012 | 19 | 5.3 | [0.9–24.6] | 0.95 | [0.08–11.44] | 1.000 |  |
| 2013 | 8 | 0.0 | [0–32.4] | – | – | – |  |
| 2014 | 22 | 31.8 | [16.4–52.7] | – | – | – |  |
| 2015 | 30 | 46.7 | [30.2–63.9] | 1.7 | [0.61–4.69] | 1.000 |  |
| 2016 | 21 | 33.3 | [17.2–54.6] | 0.63 | [0.23–1.75] | 1.000 |  |
| 2017 | 29 | 34.5 | [19.9–52.7] | 0.9 | [0.31–2.59] | 1.000 |  |
| 2018 | 26 | 34.6 | [19.4–53.8] | 1.11 | [0.41–2.96] | 1.000 |  |
| 2019 | 21 | 14.3 | [5–34.6] | 0.3 | [0.08–1.09] | 1.000 |  |
| 2020 | 11 | 0.0 | [0–25.9] | – | – | – |  |
| 2021 | 15 | 6.7 | [1.2–29.8] | – | – | – |  |
| 2022 | 16 | 6.2 | [1.1–28.3] | 0.85 | [0.07–10.41] | 1.000 |  |
| 2023 | 18 | 0.0 | [0–17.6] | – | – | – |  |
| 2024 | 17 | 0.0 | [0–18.4] | – | – | – |  |

### Figure S12: Annual percentage of *Enterococcus faecalis* resistance from 2005 to 2024.


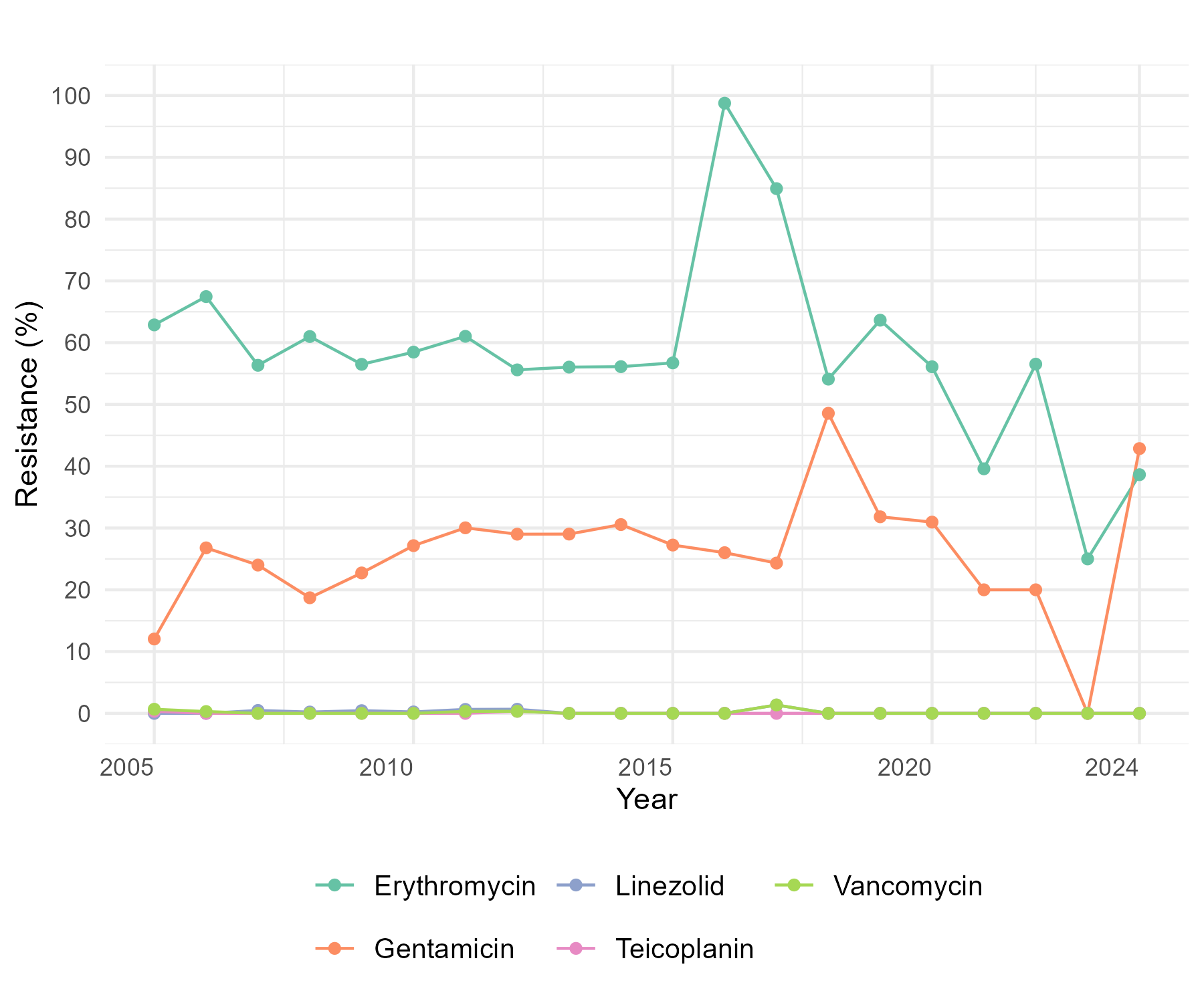


### Table S119: Logistic regression analysis of the effect of continuous time on the antibiotic resistance of *Enterococcus faecalis* from 2005 to 2024.

| Antibiotic | β | OR | [95%CI] | P-value |  |
| --- | --- | --- | --- | --- | --- |
| Erythromycin | 0.008 | 1.01 | [0.99–1.02] | 0.322 |  |
| Gentamicin | 0.014 | 1.01 | [1–1.03] | 0.129 |  |
| Linezolid | -0.076 | 0.93 | [0.81–1.07] | 0.288 |  |
| Teicoplanin | -0.204 | 0.82 | [0.54–1.23] | 0.331 |  |
| Vancomycin | -0.022 | 0.98 | [0.81–1.18] | 0.819 |  |

### Table S120: Annual percentage of *Enterococcus faecalis* resistance to Erythromycin from 2005 to 2024.

|  | Tested | Resistance | | Logistic model | |  | |
| --- | --- | --- | --- | --- | --- | --- | --- |
| Year | N | % | [95% CI] | OR | [95% CI] | P-value |  |
| 2005 | 299 | 62.9 | [57.3–68.2] | – | – | – |  |
| 2006 | 338 | 67.5 | [62.3–72.2] | 1.24 | [0.89–1.73] | 0.382 |  |
| 2007 | 426 | 56.3 | [51.6–61] | 0.61 | [0.45–0.83] | 0.006 | ** |
| 2008 | 464 | 61.0 | [56.5–65.3] | 1.21 | [0.93–1.59] | 0.370 |  |
| 2009 | 462 | 56.5 | [51.9–60.9] | 0.83 | [0.64–1.08] | 0.370 |  |
| 2010 | 431 | 58.5 | [53.8–63] | 1.05 | [0.8–1.37] | 0.865 |  |
| 2011 | 254 | 61.0 | [54.9–66.8] | 1.08 | [0.79–1.49] | 0.796 |  |
| 2012 | 277 | 55.6 | [49.7–61.3] | 0.81 | [0.57–1.14] | 0.391 |  |
| 2013 | 182 | 56.0 | [48.8–63.1] | 0.99 | [0.68–1.45] | 0.976 |  |
| 2014 | 278 | 56.1 | [50.2–61.8] | 0.98 | [0.67–1.44] | 0.976 |  |
| 2015 | 275 | 56.7 | [50.8–62.5] | 1.01 | [0.72–1.42] | 0.976 |  |
| 2016 | 324 | 98.8 | [96.9–99.5] | 63.19 | [22.83–174.88] | 0.000 | *** |
| 2017 | 146 | 84.9 | [78.2–89.8] | 0.07 | [0.02–0.2] | 0.000 | *** |
| 2018 | 61 | 54.1 | [41.7–66] | 0.21 | [0.1–0.41] | 0.000 | *** |
| 2019 | 44 | 63.6 | [48.9–76.2] | 1.48 | [0.66–3.28] | 0.537 |  |
| 2020 | 41 | 56.1 | [41–70.1] | 0.69 | [0.29–1.66] | 0.599 |  |
| 2021 | 48 | 39.6 | [27–53.7] | 0.52 | [0.22–1.22] | 0.370 |  |
| 2022 | 23 | 56.5 | [36.8–74.4] | 2.06 | [0.75–5.69] | 0.370 |  |
| 2023 | 8 | 25.0 | [7.1–59.1] | 0.26 | [0.04–1.6] | 0.370 |  |
| 2024 | 44 | 38.6 | [25.7–53.4] | 1.81 | [0.32–10.14] | 0.676 |  |

### Table S121: Annual percentage of *Enterococcus faecalis* resistance to Gentamicin from 2005 to 2024.

|  | Tested | Resistance | | Logistic model | |  | |
| --- | --- | --- | --- | --- | --- | --- | --- |
| Year | N | % | [95% CI] | OR | [95% CI] | P-value |  |
| 2005 | 299 | 12.0 | [8.8–16.2] | – | – | – |  |
| 2006 | 336 | 26.8 | [22.3–31.8] | 2.68 | [1.74–4.12] | 0.000 | *** |
| 2007 | 425 | 24.0 | [20.2–28.3] | 0.87 | [0.63–1.22] | 0.864 |  |
| 2008 | 465 | 18.7 | [15.4–22.5] | 0.72 | [0.52–0.99] | 0.278 |  |
| 2009 | 462 | 22.7 | [19.1–26.8] | 1.29 | [0.94–1.78] | 0.506 |  |
| 2010 | 431 | 27.1 | [23.2–31.5] | 1.19 | [0.87–1.62] | 0.703 |  |
| 2011 | 313 | 30.0 | [25.2–35.3] | 1.08 | [0.78–1.5] | 0.953 |  |
| 2012 | 300 | 29.0 | [24.2–34.4] | 0.99 | [0.69–1.4] | 0.953 |  |
| 2013 | 224 | 29.0 | [23.5–35.3] | 0.94 | [0.64–1.39] | 0.953 |  |
| 2014 | 324 | 30.6 | [25.8–35.8] | 1.04 | [0.71–1.52] | 0.953 |  |
| 2015 | 279 | 27.2 | [22.4–32.7] | 0.83 | [0.58–1.18] | 0.703 |  |
| 2016 | 323 | 26.0 | [21.5–31.1] | 0.99 | [0.68–1.42] | 0.953 |  |
| 2017 | 111 | 24.3 | [17.3–33.1] | 0.82 | [0.5–1.37] | 0.864 |  |
| 2018 | 35 | 48.6 | [33–64.4] | 2.83 | [1.27–6.29] | 0.103 |  |
| 2019 | 44 | 31.8 | [20–46.6] | 0.49 | [0.2–1.24] | 0.506 |  |
| 2020 | 42 | 31.0 | [19.1–46] | 0.86 | [0.35–2.15] | 0.953 |  |
| 2021 | 50 | 20.0 | [11.2–33] | 0.57 | [0.22–1.48] | 0.703 |  |
| 2022 | 25 | 20.0 | [8.9–39.1] | 1.07 | [0.32–3.56] | 0.953 |  |
| 2023 | 3 | 0.0 | [0–56.1] | 0 | [0–2.091889958816e+154] | 0.953 |  |
| 2024 | 7 | 42.9 | [15.8–75] | 190544.82 | [0–2.69055504046314e+164] | 0.953 |  |

### Table S122: Annual percentage of *Enterococcus faecalis* resistance to Linezolid from 2005 to 2024.

|  | Tested | Resistance | | Logistic model | |  | |
| --- | --- | --- | --- | --- | --- | --- | --- |
| Year | N | % | [95% CI] | OR | [95% CI] | P-value |  |
| 2005 | 78 | 0.0 | [0–4.7] | – | – | – |  |
| 2006 | 335 | 0.0 | [0–1.1] | – | – | – |  |
| 2007 | 425 | 0.5 | [0.1–1.7] | – | – | – |  |
| 2008 | 465 | 0.2 | [0–1.2] | 0.46 | [0.08–2.57] | 1.000 |  |
| 2009 | 458 | 0.4 | [0.1–1.6] | 2.04 | [0.37–11.37] | 1.000 |  |
| 2010 | 431 | 0.2 | [0–1.3] | 0.46 | [0.08–2.55] | 1.000 |  |
| 2011 | 313 | 0.6 | [0.2–2.3] | 2.46 | [0.44–13.72] | 1.000 |  |
| 2012 | 300 | 0.7 | [0.2–2.4] | 1.12 | [0.27–4.57] | 1.000 |  |
| 2013 | 222 | 0.0 | [0–1.7] | – | – | – |  |
| 2014 | 325 | 0.0 | [0–1.2] | – | – | – |  |
| 2015 | 279 | 0.0 | [0–1.4] | – | – | – |  |
| 2016 | 325 | 0.0 | [0–1.2] | – | – | – |  |
| 2017 | 148 | 1.4 | [0.4–4.8] | – | – | – |  |
| 2018 | 61 | 0.0 | [0–5.9] | – | – | – |  |
| 2019 | 46 | 0.0 | [0–7.7] | – | – | – |  |
| 2020 | 41 | 0.0 | [0–8.6] | – | – | – |  |
| 2021 | 47 | 0.0 | [0–7.6] | – | – | – |  |
| 2022 | 26 | 0.0 | [0–12.9] | – | – | – |  |
| 2023 | 42 | 0.0 | [0–8.4] | – | – | – |  |
| 2024 | 51 | 0.0 | [0–7] | – | – | – |  |

### Table S123: Annual percentage of *Enterococcus faecalis* resistance to Teicoplanin from 2005 to 2024.

|  | Tested | Resistance | | Logistic model | |  | |
| --- | --- | --- | --- | --- | --- | --- | --- |
| Year | N | % | [95% CI] | OR | [95% CI] | P-value |  |
| 2005 | 299 | 0.3 | [0.1–1.9] | – | – | – |  |
| 2006 | 338 | 0.0 | [0–1.1] | – | – | – |  |
| 2007 | 425 | 0.0 | [0–0.9] | – | – | – |  |
| 2008 | 465 | 0.0 | [0–0.8] | – | – | – |  |
| 2009 | 462 | 0.0 | [0–0.8] | – | – | – |  |
| 2010 | 431 | 0.0 | [0–0.9] | – | – | – |  |
| 2011 | 313 | 0.0 | [0–1.2] | – | – | – |  |
| 2012 | 300 | 0.3 | [0.1–1.9] | – | – | – |  |
| 2013 | 223 | 0.0 | [0–1.7] | – | – | – |  |
| 2014 | 326 | 0.0 | [0–1.2] | – | – | – |  |
| 2015 | 279 | 0.0 | [0–1.4] | – | – | – |  |
| 2016 | 325 | 0.0 | [0–1.2] | – | – | – |  |
| 2017 | 147 | 0.0 | [0–2.5] | – | – | – |  |
| 2018 | 61 | 0.0 | [0–5.9] | – | – | – |  |
| 2019 | 45 | 0.0 | [0–7.9] | – | – | – |  |
| 2020 | 42 | 0.0 | [0–8.4] | – | – | – |  |
| 2021 | 50 | 0.0 | [0–7.1] | – | – | – |  |
| 2022 | 26 | 0.0 | [0–12.9] | – | – | – |  |
| 2023 | 42 | 0.0 | [0–8.4] | – | – | – |  |
| 2024 | 51 | 0.0 | [0–7] | – | – | – |  |

### Table S124: Annual percentage of *Enterococcus faecalis* resistance to Vancomycin from 2005 to 2024.

|  | Tested | Resistance | | Logistic model | |  | |
| --- | --- | --- | --- | --- | --- | --- | --- |
| Year | N | % | [95% CI] | OR | [95% CI] | P-value |  |
| 2005 | 299 | 0.7 | [0.2–2.4] | – | – | – |  |
| 2006 | 338 | 0.3 | [0.1–1.7] | 0.46 | [0.12–1.73] | 1.000 |  |
| 2007 | 426 | 0.0 | [0–0.9] | – | – | – |  |
| 2008 | 465 | 0.0 | [0–0.8] | – | – | – |  |
| 2009 | 462 | 0.0 | [0–0.8] | – | – | – |  |
| 2010 | 430 | 0.0 | [0–0.9] | – | – | – |  |
| 2011 | 313 | 0.3 | [0.1–1.8] | – | – | – |  |
| 2012 | 300 | 0.3 | [0.1–1.9] | 1.01 | [0.22–4.68] | 1.000 |  |
| 2013 | 223 | 0.0 | [0–1.7] | – | – | – |  |
| 2014 | 326 | 0.0 | [0–1.2] | – | – | – |  |
| 2015 | 279 | 0.0 | [0–1.4] | – | – | – |  |
| 2016 | 325 | 0.0 | [0–1.2] | – | – | – |  |
| 2017 | 147 | 1.4 | [0.4–4.8] | – | – | – |  |
| 2018 | 61 | 0.0 | [0–5.9] | – | – | – |  |
| 2019 | 46 | 0.0 | [0–7.7] | – | – | – |  |
| 2020 | 43 | 0.0 | [0–8.2] | – | – | – |  |
| 2021 | 50 | 0.0 | [0–7.1] | – | – | – |  |
| 2022 | 26 | 0.0 | [0–12.9] | – | – | – |  |
| 2023 | 42 | 0.0 | [0–8.4] | – | – | – |  |
| 2024 | 51 | 0.0 | [0–7] | – | – | – |  |

## Mycobacteria

### Figure S13: Annual percentage of *Mycobacterium tuberculosis* resistance from 2005 to 2024.


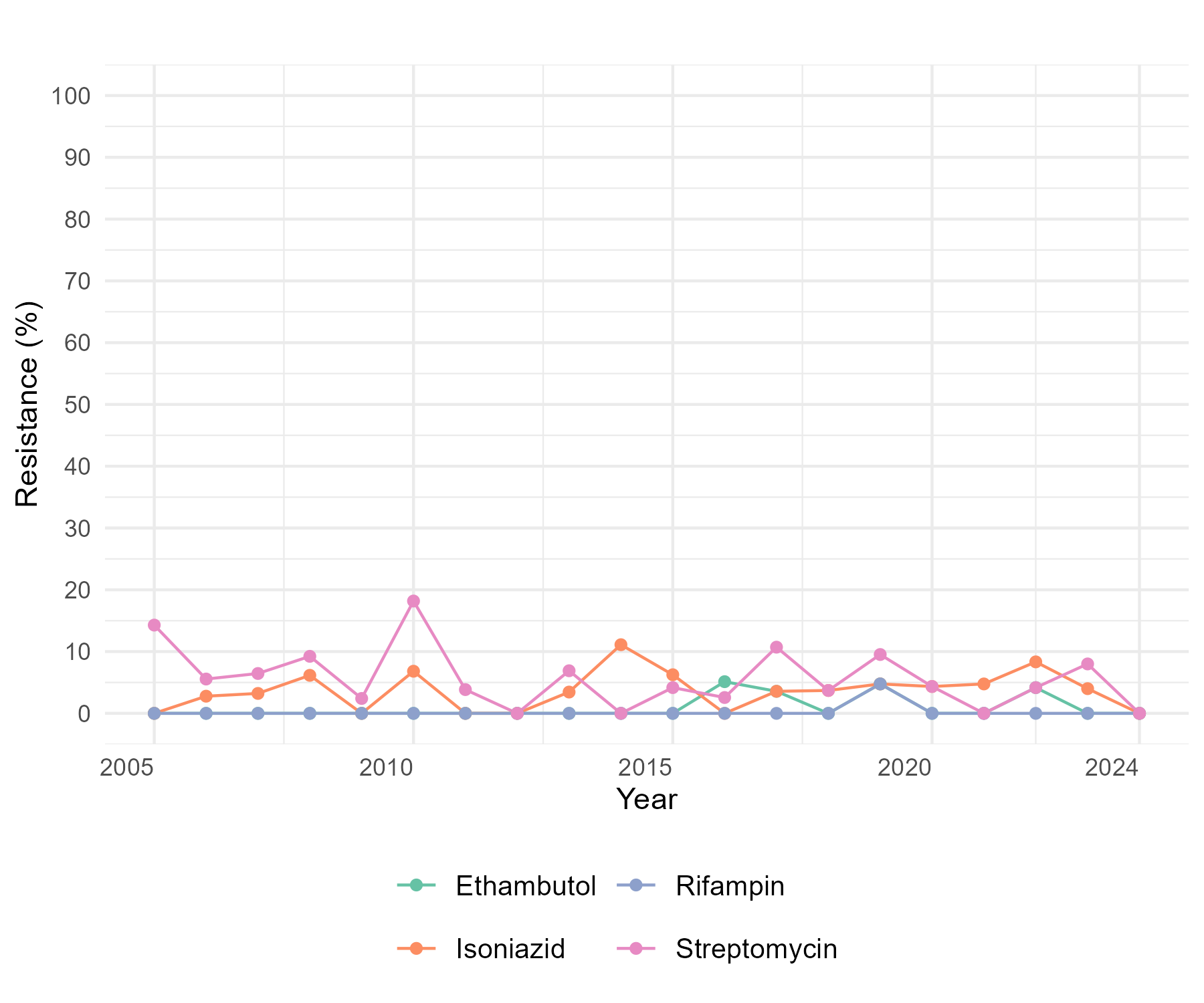


### Table S125: Logistic regression analysis of the effect of continuous time on the antibiotic resistance of *Mycobacterium tuberculosis* from 2005 to 2024.

| Antibiotic | β | OR | [95%CI] | P-value |  |
| --- | --- | --- | --- | --- | --- |
| Ethambutol | 0.151 | 1.16 | [1–1.35] | 0.051 | . |
| Isoniazid | 0.038 | 1.04 | [0.97–1.12] | 0.286 |  |
| Rifampin | 0.209 | 1.23 | [1.01–1.5] | 0.037 | * |
| Streptomycin | -0.040 | 0.96 | [0.9–1.03] | 0.236 |  |

### Table S126: Annual percentage of *Mycobacterium tuberculosis* resistance to Ethambutol from 2005 to 2024.

|  | Tested | Resistance | | Logistic model | |  | |
| --- | --- | --- | --- | --- | --- | --- | --- |
| Year | N | % | [95% CI] | OR | [95% CI] | P-value |  |
| 2005 | 7 | 0.0 | [0–35.4] | – | – | – |  |
| 2006 | 36 | 0.0 | [0–9.6] | – | – | – |  |
| 2007 | 31 | 0.0 | [0–11] | – | – | – |  |
| 2008 | 65 | 0.0 | [0–5.6] | – | – | – |  |
| 2009 | 83 | 0.0 | [0–4.4] | – | – | – |  |
| 2010 | 44 | 0.0 | [0–8] | – | – | – |  |
| 2011 | 26 | 0.0 | [0–12.9] | – | – | – |  |
| 2012 | 32 | 0.0 | [0–10.7] | – | – | – |  |
| 2013 | 29 | 0.0 | [0–11.7] | – | – | – |  |
| 2014 | 18 | 0.0 | [0–17.6] | – | – | – |  |
| 2015 | 48 | 0.0 | [0–7.4] | – | – | – |  |
| 2016 | 39 | 5.1 | [1.4–16.9] | – | – | – |  |
| 2017 | 28 | 3.6 | [0.6–17.7] | 0.84 | [0.29–2.43] | 1.000 |  |
| 2018 | 27 | 0.0 | [0–12.5] | – | – | – |  |
| 2019 | 21 | 4.8 | [0.8–22.7] | – | – | – |  |
| 2020 | 23 | 0.0 | [0–14.3] | – | – | – |  |
| 2021 | 21 | 0.0 | [0–15.5] | – | – | – |  |
| 2022 | 24 | 4.2 | [0.7–20.2] | – | – | – |  |
| 2023 | 25 | 0.0 | [0–13.3] | – | – | – |  |
| 2024 | 19 | 0.0 | [0–16.8] | – | – | – |  |

### Table S127: Annual percentage of *Mycobacterium tuberculosis* resistance to Isoniazid from 2005 to 2024.

|  | Tested | Resistance | | Logistic model | |  | |
| --- | --- | --- | --- | --- | --- | --- | --- |
| Year | N | % | [95% CI] | OR | [95% CI] | P-value |  |
| 2005 | 7 | 0.0 | [0–35.4] | – | – | – |  |
| 2006 | 36 | 2.8 | [0.5–14.2] | – | – | – |  |
| 2007 | 31 | 3.2 | [0.6–16.2] | 1.31 | [0.13–13.41] | 1.000 |  |
| 2008 | 65 | 6.2 | [2.4–14.8] | 1.74 | [0.27–11.12] | 1.000 |  |
| 2009 | 83 | 0.0 | [0–4.4] | – | – | – |  |
| 2010 | 44 | 6.8 | [2.3–18.2] | – | – | – |  |
| 2011 | 27 | 0.0 | [0–12.5] | – | – | – |  |
| 2012 | 32 | 0.0 | [0–10.7] | – | – | – |  |
| 2013 | 29 | 3.4 | [0.6–17.2] | – | – | – |  |
| 2014 | 18 | 11.1 | [3.1–32.8] | 2.97 | [0.37–23.99] | 1.000 |  |
| 2015 | 48 | 6.2 | [2.1–16.8] | 0.87 | [0.17–4.47] | 1.000 |  |
| 2016 | 39 | 0.0 | [0–9] | – | – | – |  |
| 2017 | 28 | 3.6 | [0.6–17.7] | – | – | – |  |
| 2018 | 27 | 3.7 | [0.7–18.3] | 0.94 | [0.09–9.81] | 1.000 |  |
| 2019 | 21 | 4.8 | [0.8–22.7] | 1.63 | [0.15–17.3] | 1.000 |  |
| 2020 | 23 | 4.3 | [0.8–21] | 0.53 | [0.05–5.74] | 1.000 |  |
| 2021 | 21 | 4.8 | [0.8–22.7] | 1.77 | [0.16–19.16] | 1.000 |  |
| 2022 | 24 | 8.3 | [2.3–25.8] | 1.16 | [0.14–9.69] | 1.000 |  |
| 2023 | 25 | 4.0 | [0.7–19.5] | 0.61 | [0.08–5] | 1.000 |  |
| 2024 | 19 | 0.0 | [0–16.8] | – | – | – |  |

### Table S128: Annual percentage of *Mycobacterium tuberculosis* resistance to Rifampicin from 2005 to 2024.

|  | Tested | Resistance | | Logistic model | |  | |
| --- | --- | --- | --- | --- | --- | --- | --- |
| Year | N | % | [95% CI] | OR | [95% CI] | P-value |  |
| 2005 | 7 | 0.0 | [0–35.4] | – | – | – |  |
| 2006 | 36 | 0.0 | [0–9.6] | – | – | – |  |
| 2007 | 31 | 0.0 | [0–11] | – | – | – |  |
| 2008 | 65 | 0.0 | [0–5.6] | – | – | – |  |
| 2009 | 83 | 0.0 | [0–4.4] | – | – | – |  |
| 2010 | 44 | 0.0 | [0–8] | – | – | – |  |
| 2011 | 27 | 0.0 | [0–12.5] | – | – | – |  |
| 2012 | 32 | 0.0 | [0–10.7] | – | – | – |  |
| 2013 | 29 | 0.0 | [0–11.7] | – | – | – |  |
| 2014 | 18 | 0.0 | [0–17.6] | – | – | – |  |
| 2015 | 48 | 0.0 | [0–7.4] | – | – | – |  |
| 2016 | 39 | 0.0 | [0–9] | – | – | – |  |
| 2017 | 28 | 0.0 | [0–12.1] | – | – | – |  |
| 2018 | 27 | 0.0 | [0–12.5] | – | – | – |  |
| 2019 | 21 | 4.8 | [0.8–22.7] | – | – | – |  |
| 2020 | 23 | 0.0 | [0–14.3] | – | – | – |  |
| 2021 | 21 | 0.0 | [0–15.5] | – | – | – |  |
| 2022 | 24 | 0.0 | [0–13.8] | – | – | – |  |
| 2023 | 25 | 0.0 | [0–13.3] | – | – | – |  |
| 2024 | 19 | 0.0 | [0–16.8] | – | – | – |  |

### Table S129: Annual percentage of *Mycobacterium tuberculosis* resistance to Streptomycin from 2005 to 2024.

|  | Tested | Resistance | | Logistic model | |  | |
| --- | --- | --- | --- | --- | --- | --- | --- |
| Year | N | % | [95% CI] | OR | [95% CI] | P-value |  |
| 2005 | 7 | 14.3 | [2.6–51.3] | – | – | – |  |
| 2006 | 36 | 5.6 | [1.5–18.1] | 0.32 | [0.03–3.97] | 0.961 |  |
| 2007 | 31 | 6.5 | [1.8–20.7] | 1.21 | [0.16–8.96] | 0.995 |  |
| 2008 | 65 | 9.2 | [4.3–18.7] | 1.41 | [0.27–7.37] | 0.995 |  |
| 2009 | 83 | 2.4 | [0.7–8.4] | 0.21 | [0.04–1.08] | 0.589 |  |
| 2010 | 44 | 18.2 | [9.5–32] | 10.37 | [2.11–50.84] | 0.075 | . |
| 2011 | 26 | 3.8 | [0.7–18.9] | 0.19 | [0.02–1.54] | 0.750 |  |
| 2012 | 32 | 0.0 | [0–10.7] | – | – | – |  |
| 2013 | 29 | 6.9 | [1.9–22] | – | – | – |  |
| 2014 | 18 | 0.0 | [0–17.6] | – | – | – |  |
| 2015 | 48 | 4.2 | [1.2–14] | – | – | – |  |
| 2016 | 39 | 2.6 | [0.5–13.2] | 0.71 | [0.06–7.89] | 0.995 |  |
| 2017 | 28 | 10.7 | [3.7–27.2] | 4.15 | [0.42–41.22] | 0.961 |  |
| 2018 | 27 | 3.7 | [0.7–18.3] | 0.3 | [0.03–3.04] | 0.961 |  |
| 2019 | 21 | 9.5 | [2.7–28.9] | 3.07 | [0.27–35.39] | 0.961 |  |
| 2020 | 23 | 4.3 | [0.8–21] | 0.35 | [0.03–4.11] | 0.961 |  |
| 2021 | 21 | 0.0 | [0–15.5] | – | – | – |  |
| 2022 | 24 | 4.2 | [0.7–20.2] | – | – | – |  |
| 2023 | 25 | 8.0 | [2.2–25] | 2 | [0.17–23.11] | 0.995 |  |
| 2024 | 19 | 0.0 | [0–16.8] | – | – | – |  |

## Non-fermenters

### Figure S14: Annual percentage of *Acinetobacter baumannii* resistance from 2005 to 2024.


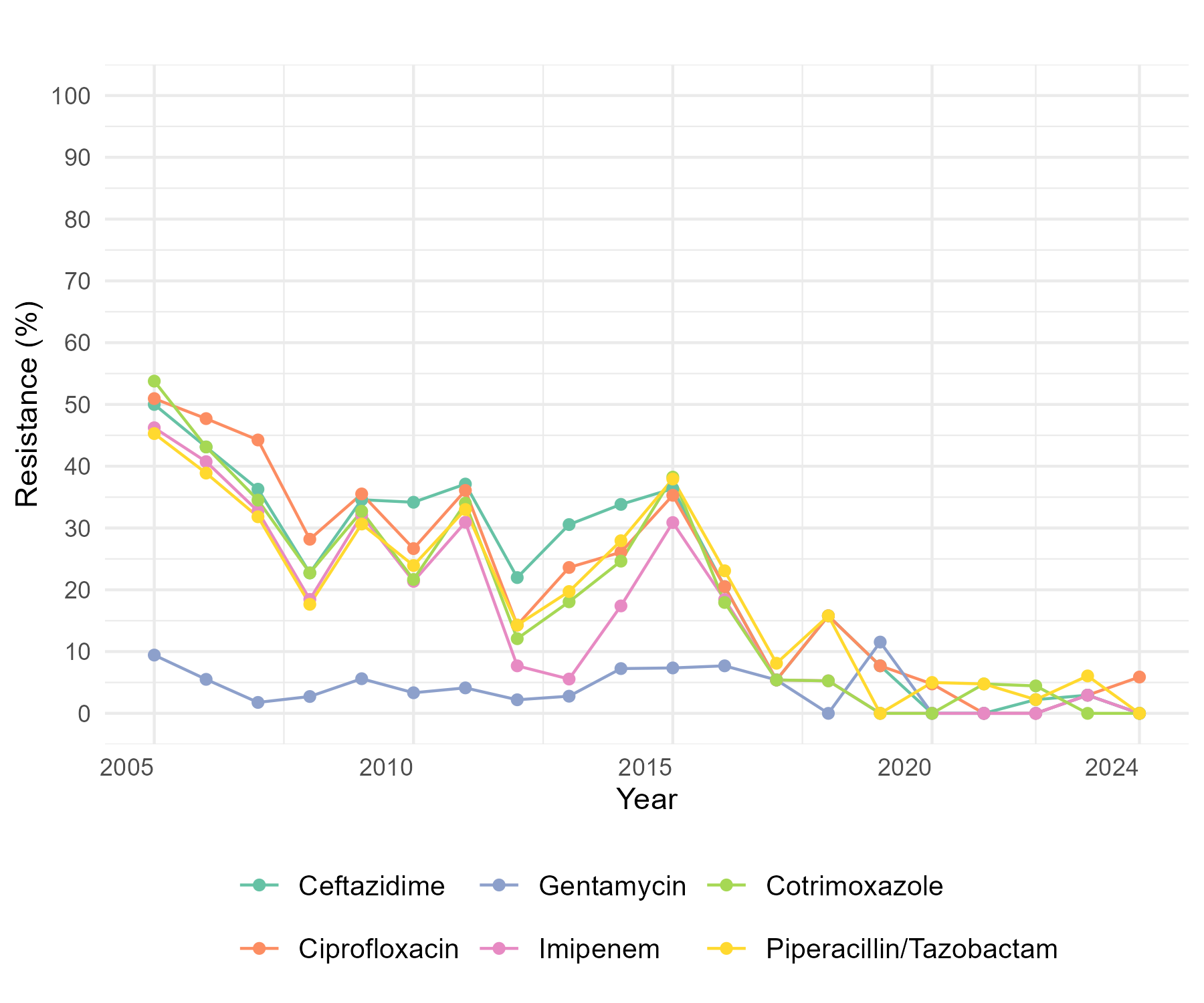


### Table S130: Logistic regression analysis of the effect of continuous time on the antibiotic resistance of *Acinetobacter baumannii* from 2005 to 2024.

| Antibiotic | β | OR | [95%CI] | P-value |  |
| --- | --- | --- | --- | --- | --- |
| Ceftazidime | -0.143 | 0.87 | [0.84–0.89] | 0.000 | *** |
| Ciprofloxacin | -0.170 | 0.84 | [0.81–0.87] | 0.000 | *** |
| Gentamicin | -0.038 | 0.96 | [0.91–1.02] | 0.177 |  |
| Imipenem | -0.187 | 0.83 | [0.8–0.86] | 0.000 | *** |
| Cotrimoxazole | -0.171 | 0.84 | [0.82–0.87] | 0.000 | *** |
| Piperacillin/Tazobactam | -0.130 | 0.88 | [0.85–0.9] | 0.000 | *** |

### Table S131: Annual percentage of *Acinetobacter baumannii* resistance to Ceftazidime from 2005 to 2024.

|  | Tested | Resistance | | Logistic model | |  | |
| --- | --- | --- | --- | --- | --- | --- | --- |
| Year | N | % | [95% CI] | OR | [95% CI] | P-value |  |
| 2005 | 106 | 50.0 | [40.6–59.4] | – | – | – |  |
| 2006 | 109 | 43.1 | [34.2–52.5] | 0.68 | [0.39–1.2] | 0.490 |  |
| 2007 | 113 | 36.3 | [28–45.5] | 0.75 | [0.43–1.32] | 0.680 |  |
| 2008 | 110 | 22.7 | [15.9–31.4] | 0.52 | [0.28–0.95] | 0.236 |  |
| 2009 | 107 | 34.6 | [26.2–44] | 2.05 | [1.1–3.82] | 0.236 |  |
| 2010 | 120 | 34.2 | [26.3–43] | 0.94 | [0.53–1.66] | 1.000 |  |
| 2011 | 97 | 37.1 | [28.2–47] | 1.3 | [0.72–2.33] | 0.723 |  |
| 2012 | 91 | 22.0 | [14.7–31.5] | 0.49 | [0.25–0.96] | 0.236 |  |
| 2013 | 72 | 30.6 | [21.1–42] | 1.15 | [0.56–2.4] | 1.000 |  |
| 2014 | 68 | 33.8 | [23.7–45.7] | 1.32 | [0.63–2.74] | 0.798 |  |
| 2015 | 66 | 36.4 | [25.8–48.4] | 0.9 | [0.43–1.86] | 1.000 |  |
| 2016 | 39 | 20.5 | [10.8–35.5] | 0.47 | [0.18–1.21] | 0.402 |  |
| 2017 | 37 | 5.4 | [1.5–17.7] | 0.23 | [0.05–1.14] | 0.342 |  |
| 2018 | 19 | 15.8 | [5.5–37.6] | 4.32 | [0.66–28.26] | 0.402 |  |
| 2019 | 26 | 7.7 | [2.1–24.1] | 0.38 | [0.06–2.56] | 0.680 |  |
| 2020 | 20 | 0.0 | [0–16.1] | – | – | – |  |
| 2021 | 21 | 0.0 | [0–15.5] | – | – | – |  |
| 2022 | 45 | 2.2 | [0.4–11.6] | – | – | – |  |
| 2023 | 34 | 2.9 | [0.5–14.9] | 1.6 | [0.1–25.21] | 1.000 |  |
| 2024 | 34 | 0.0 | [0–10.2] | – | – | – |  |

### Table S132: Annual percentage of *Acinetobacter baumannii* resistance to Ciprofloxacin from 2005 to 2024.

|  | Tested | Resistance | | Logistic model | |  | |
| --- | --- | --- | --- | --- | --- | --- | --- |
| Year | N | % | [95% CI] | OR | [95% CI] | P-value |  |
| 2005 | 106 | 50.9 | [41.6–60.3] | – | – | – |  |
| 2006 | 109 | 47.7 | [38.6–57] | 0.79 | [0.41–1.5] | 0.816 |  |
| 2007 | 113 | 44.2 | [35.4–53.4] | 0.88 | [0.47–1.66] | 0.826 |  |
| 2008 | 110 | 28.2 | [20.6–37.2] | 0.48 | [0.25–0.94] | 0.305 |  |
| 2009 | 107 | 35.5 | [27.1–44.9] | 1.59 | [0.8–3.16] | 0.443 |  |
| 2010 | 120 | 26.7 | [19.6–35.2] | 0.58 | [0.29–1.14] | 0.431 |  |
| 2011 | 97 | 36.1 | [27.2–46] | 1.93 | [0.97–3.84] | 0.395 |  |
| 2012 | 91 | 14.3 | [8.5–22.9] | 0.28 | [0.12–0.64] | 0.053 | . |
| 2013 | 72 | 23.6 | [15.3–34.6] | 1.36 | [0.54–3.44] | 0.816 |  |
| 2014 | 69 | 26.1 | [17.2–37.5] | 1.34 | [0.55–3.27] | 0.816 |  |
| 2015 | 68 | 35.3 | [25–47.2] | 1.25 | [0.54–2.93] | 0.816 |  |
| 2016 | 39 | 20.5 | [10.8–35.5] | 0.49 | [0.17–1.41] | 0.443 |  |
| 2017 | 37 | 5.4 | [1.5–17.7] | 0.22 | [0.04–1.38] | 0.431 |  |
| 2018 | 19 | 15.8 | [5.5–37.6] | 4.59 | [0.55–38.08] | 0.443 |  |
| 2019 | 26 | 7.7 | [2.1–24.1] | 0.37 | [0.04–3.15] | 0.761 |  |
| 2020 | 21 | 4.8 | [0.8–22.7] | 0.54 | [0.03–8.58] | 0.826 |  |
| 2021 | 21 | 0.0 | [0–15.5] | – | – | – |  |
| 2022 | 44 | 0.0 | [0–8] | – | – | – |  |
| 2023 | 34 | 2.9 | [0.5–14.9] | – | – | – |  |
| 2024 | 34 | 5.9 | [1.6–19.1] | 2.22 | [0.15–33.32] | 0.816 |  |

### Table S133: Annual percentage of *Acinetobacter baumannii* resistance to Gentamycin from 2005 to 2024.

|  | Tested | Resistance | | Logistic model | |  | |
| --- | --- | --- | --- | --- | --- | --- | --- |
| Year | N | % | [95% CI] | OR | [95% CI] | P-value |  |
| 2005 | 106 | 9.4 | [5.2–16.5] | – | – | – |  |
| 2006 | 109 | 5.5 | [2.5–11.5] | 0.54 | [0.2–1.42] | 1.000 |  |
| 2007 | 113 | 1.8 | [0.5–6.2] | 0.31 | [0.07–1.4] | 1.000 |  |
| 2008 | 110 | 2.7 | [0.9–7.7] | 1.63 | [0.31–8.58] | 1.000 |  |
| 2009 | 107 | 5.6 | [2.6–11.7] | 2.18 | [0.59–8] | 1.000 |  |
| 2010 | 120 | 3.3 | [1.3–8.3] | 0.57 | [0.17–1.86] | 1.000 |  |
| 2011 | 97 | 4.1 | [1.6–10.1] | 1.33 | [0.36–4.88] | 1.000 |  |
| 2012 | 91 | 2.2 | [0.6–7.7] | 0.56 | [0.11–2.71] | 1.000 |  |
| 2013 | 72 | 2.8 | [0.8–9.6] | 1.05 | [0.17–6.55] | 1.000 |  |
| 2014 | 69 | 7.2 | [3.1–15.9] | 2.93 | [0.63–13.68] | 1.000 |  |
| 2015 | 68 | 7.4 | [3.2–16.1] | 0.91 | [0.28–2.98] | 1.000 |  |
| 2016 | 39 | 7.7 | [2.7–20.3] | 1.1 | [0.28–4.37] | 1.000 |  |
| 2017 | 37 | 5.4 | [1.5–17.7] | 0.72 | [0.13–3.95] | 1.000 |  |
| 2018 | 19 | 0.0 | [0–16.8] | – | – | – |  |
| 2019 | 26 | 11.5 | [4–29] | – | – | – |  |
| 2020 | 17 | 0.0 | [0–18.4] | – | – | – |  |
| 2021 | 20 | 0.0 | [0–16.1] | – | – | – |  |
| 2022 | 45 | 0.0 | [0–7.9] | – | – | – |  |
| 2023 | 34 | 2.9 | [0.5–14.9] | – | – | – |  |
| 2024 | 34 | 0.0 | [0–10.2] | – | – | – |  |

### Table S134: Annual percentage of *Acinetobacter baumannii* resistance to Imipenem from 2005 to 2024.

|  | Tested | Resistance | | Logistic model | |  | |
| --- | --- | --- | --- | --- | --- | --- | --- |
| Year | N | % | [95% CI] | OR | [95% CI] | P-value |  |
| 2005 | 106 | 46.2 | [37–55.7] | – | – | – |  |
| 2006 | 108 | 40.7 | [31.9–50.2] | 0.72 | [0.42–1.24] | 0.407 |  |
| 2007 | 110 | 32.7 | [24.7–41.9] | 0.69 | [0.4–1.2] | 0.367 |  |
| 2008 | 103 | 18.4 | [12.1–27] | 0.48 | [0.26–0.9] | 0.105 |  |
| 2009 | 106 | 32.1 | [24–41.5] | 2.37 | [1.25–4.49] | 0.068 | . |
| 2010 | 117 | 21.4 | [14.9–29.6] | 0.52 | [0.29–0.95] | 0.118 |  |
| 2011 | 97 | 30.9 | [22.6–40.7] | 1.92 | [1.04–3.54] | 0.118 |  |
| 2012 | 91 | 7.7 | [3.8–15] | 0.17 | [0.07–0.41] | 0.001 | ** |
| 2013 | 72 | 5.6 | [2.2–13.4] | 0.5 | [0.15–1.68] | 0.420 |  |
| 2014 | 69 | 17.4 | [10.2–28] | 4.34 | [1.41–13.4] | 0.068 | . |
| 2015 | 68 | 30.9 | [21.2–42.6] | 1.82 | [0.83–3.98] | 0.321 |  |
| 2016 | 38 | 18.4 | [9.2–33.4] | 0.52 | [0.2–1.34] | 0.367 |  |
| 2017 | 37 | 5.4 | [1.5–17.7] | 0.26 | [0.06–1.26] | 0.255 |  |
| 2018 | 19 | 5.3 | [0.9–24.6] | 1.23 | [0.12–12.55] | 1.000 |  |
| 2019 | 26 | 0.0 | [0–12.9] | – | – | – |  |
| 2020 | 21 | 0.0 | [0–15.5] | – | – | – |  |
| 2021 | 22 | 0.0 | [0–14.9] | – | – | – |  |
| 2022 | 45 | 0.0 | [0–7.9] | – | – | – |  |
| 2023 | 34 | 2.9 | [0.5–14.9] | – | – | – |  |
| 2024 | 34 | 0.0 | [0–10.2] | – | – | – |  |

### Table S135: Annual percentage of *Acinetobacter baumannii* resistance to Cotrimoxazole from 2005 to 2024.

|  | Tested | Resistance | | Logistic model | |  | |
| --- | --- | --- | --- | --- | --- | --- | --- |
| Year | N | % | [95% CI] | OR | [95% CI] | P-value |  |
| 2005 | 106 | 53.8 | [44.3–63] | – | – | – |  |
| 2006 | 109 | 43.1 | [34.2–52.5] | 0.56 | [0.32–0.98] | 0.141 |  |
| 2007 | 113 | 34.5 | [26.4–43.7] | 0.68 | [0.39–1.2] | 0.349 |  |
| 2008 | 110 | 22.7 | [15.9–31.4] | 0.56 | [0.31–1.03] | 0.172 |  |
| 2009 | 107 | 32.7 | [24.6–42.1] | 1.89 | [1.02–3.52] | 0.141 |  |
| 2010 | 120 | 21.7 | [15.2–29.9] | 0.5 | [0.27–0.92] | 0.141 |  |
| 2011 | 97 | 34.0 | [25.4–43.9] | 2.32 | [1.24–4.32] | 0.078 | . |
| 2012 | 91 | 12.1 | [6.9–20.4] | 0.25 | [0.12–0.55] | 0.009 | ** |
| 2013 | 72 | 18.1 | [10.9–28.5] | 1.17 | [0.49–2.81] | 1.000 |  |
| 2014 | 69 | 24.6 | [16–36] | 1.77 | [0.78–4.01] | 0.349 |  |
| 2015 | 68 | 38.2 | [27.6–50.1] | 1.6 | [0.76–3.36] | 0.373 |  |
| 2016 | 39 | 17.9 | [9–32.7] | 0.35 | [0.13–0.91] | 0.141 |  |
| 2017 | 37 | 5.4 | [1.5–17.7] | 0.27 | [0.05–1.36] | 0.269 |  |
| 2018 | 19 | 5.3 | [0.9–24.6] | 1.23 | [0.11–13.51] | 1.000 |  |
| 2019 | 26 | 0.0 | [0–12.9] | – | – | – |  |
| 2020 | 21 | 0.0 | [0–15.5] | – | – | – |  |
| 2021 | 21 | 4.8 | [0.8–22.7] | – | – | – |  |
| 2022 | 45 | 4.4 | [1.2–14.8] | 0.7 | [0.06–7.55] | 1.000 |  |
| 2023 | 33 | 0.0 | [0–10.4] | – | – | – |  |
| 2024 | 34 | 0.0 | [0–10.2] | – | – | – |  |

### Table S136: Annual percentage of *Acinetobacter baumannii* resistance to Piperacillin/Tazobactam from 2005 to 2024.

|  | Tested | Resistance | | Logistic model | |  | |
| --- | --- | --- | --- | --- | --- | --- | --- |
| Year | N | % | [95% CI] | OR | [95% CI] | P-value |  |
| 2005 | 106 | 45.3 | [36.1–54.8] | – | – | – |  |
| 2006 | 108 | 38.9 | [30.2–48.3] | 0.69 | [0.39–1.2] | 0.421 |  |
| 2007 | 110 | 31.8 | [23.9–41] | 0.72 | [0.41–1.28] | 0.452 |  |
| 2008 | 102 | 17.6 | [11.5–26.2] | 0.47 | [0.25–0.91] | 0.158 |  |
| 2009 | 62 | 30.6 | [20.6–43] | 2.45 | [1.15–5.23] | 0.158 |  |
| 2010 | 117 | 23.9 | [17.1–32.4] | 0.63 | [0.31–1.28] | 0.421 |  |
| 2011 | 97 | 33.0 | [24.4–42.8] | 1.82 | [0.99–3.36] | 0.259 |  |
| 2012 | 91 | 14.3 | [8.5–22.9] | 0.33 | [0.16–0.69] | 0.059 | . |
| 2013 | 71 | 19.7 | [12.1–30.4] | 1.09 | [0.48–2.49] | 0.991 |  |
| 2014 | 68 | 27.9 | [18.7–39.6] | 1.89 | [0.86–4.17] | 0.364 |  |
| 2015 | 58 | 37.9 | [26.6–50.8] | 1.3 | [0.61–2.76] | 0.679 |  |
| 2016 | 39 | 23.1 | [12.6–38.3] | 0.5 | [0.2–1.26] | 0.389 |  |
| 2017 | 37 | 8.1 | [2.8–21.3] | 0.3 | [0.08–1.17] | 0.315 |  |
| 2018 | 19 | 15.8 | [5.5–37.6] | 2.83 | [0.53–15.07] | 0.421 |  |
| 2019 | 25 | 0.0 | [0–13.3] | – | – | – |  |
| 2020 | 20 | 5.0 | [0.9–23.6] | – | – | – |  |
| 2021 | 21 | 4.8 | [0.8–22.7] | 1.02 | [0.07–15.74] | 0.991 |  |
| 2022 | 45 | 2.2 | [0.4–11.6] | 0.36 | [0.02–5.38] | 0.671 |  |
| 2023 | 33 | 6.1 | [1.7–19.6] | 3.44 | [0.33–35.95] | 0.479 |  |
| 2024 | 34 | 0.0 | [0–10.2] | – | – | – |  |

### Figure S15: Annual percentage of *Stenotrophomonas maltophilia* resistance from 2005 to 2024.


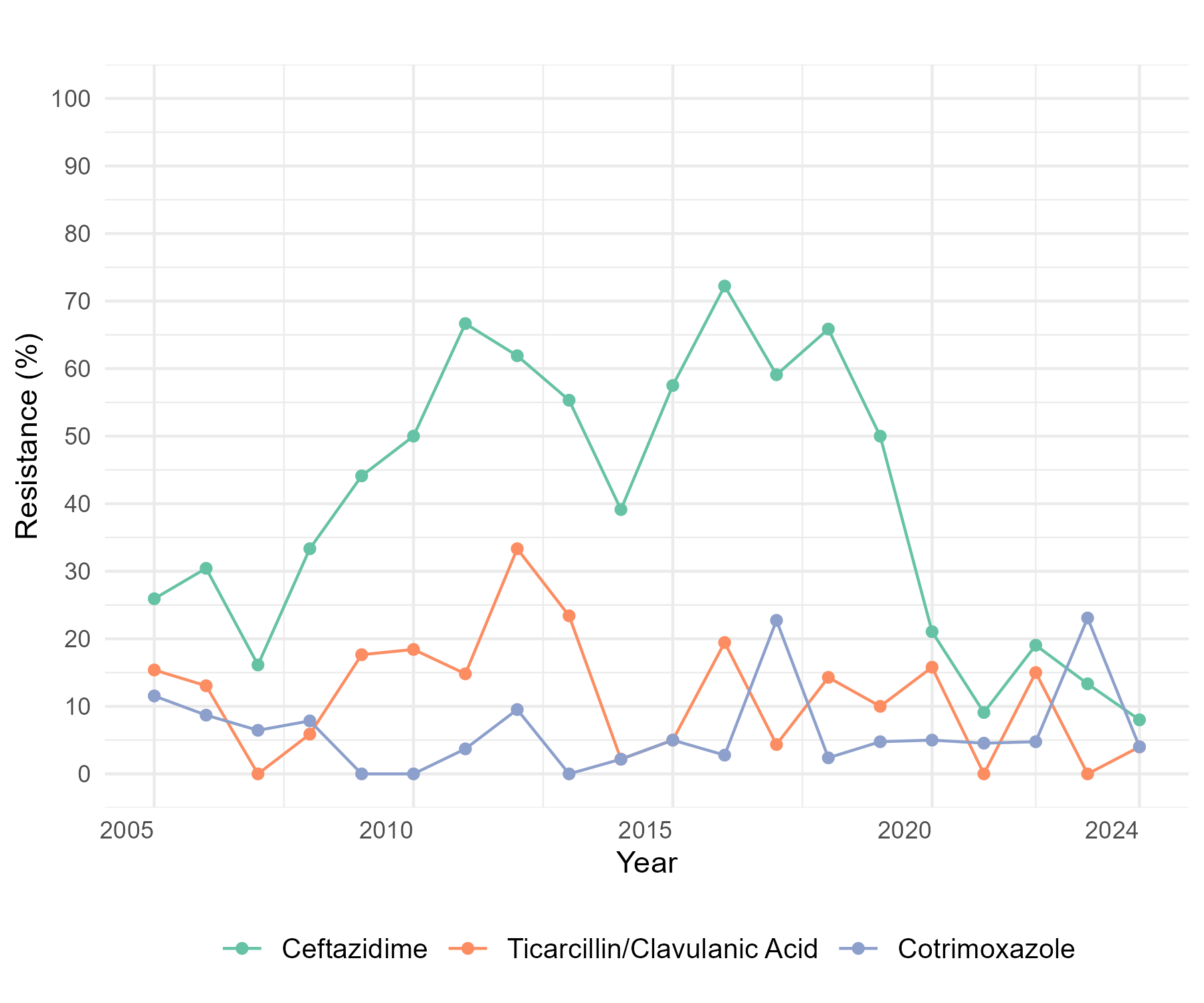


### Table S137: Logistic regression analysis of the effect of continuous time on the antibiotic resistance of *Stenotrophomonas maltophilia* from 2005 to 2024.

| Antibiotic | β | OR | [95%CI] | P-value |
| --- | --- | --- | --- | --- |
| Ceftazidime | -0.010 | 0.99 | [0.96–1.02] | 0.539 |
| Levofloxacin | -0.018 | 0.98 | [0.9–1.07] | 0.658 |
| Ticarcillin/Clavulanic Acid | -0.035 | 0.97 | [0.92–1.01] | 0.159 |
| Cotrimoxazole | 0.002 | 1.00 | [0.94–1.07] | 0.951 |

### Table S138: Annual percentage of *Stenotrophomonas maltophilia* resistance to Ceftazidime from 2005 to 2024.

|  | Tested | Resistance | | Logistic model | |  | |
| --- | --- | --- | --- | --- | --- | --- | --- |
| Year | N | % | [95% CI] | OR | [95% CI] | P-value |  |
| 2005 | 27 | 25.9 | [13.2–44.7] | – | – | – |  |
| 2006 | 23 | 30.4 | [15.6–50.9] | 1.04 | [0.29–3.74] | 0.949 |  |
| 2007 | 31 | 16.1 | [7.1–32.6] | 0.49 | [0.13–1.89] | 0.559 |  |
| 2008 | 51 | 33.3 | [22–47] | 2.6 | [0.82–8.21] | 0.559 |  |
| 2009 | 34 | 44.1 | [28.9–60.5] | 1.65 | [0.66–4.13] | 0.559 |  |
| 2010 | 32 | 50.0 | [33.6–66.4] | 1.22 | [0.45–3.33] | 0.778 |  |
| 2011 | 15 | 66.7 | [41.7–84.8] | 2.14 | [0.57–7.98] | 0.559 |  |
| 2012 | 21 | 61.9 | [40.9–79.2] | 0.81 | [0.2–3.38] | 0.818 |  |
| 2013 | 47 | 55.3 | [41.2–68.6] | 0.67 | [0.23–1.99] | 0.685 |  |
| 2014 | 46 | 39.1 | [26.4–53.5] | 0.56 | [0.24–1.31] | 0.559 |  |
| 2015 | 40 | 57.5 | [42.2–71.5] | 2.08 | [0.86–5.04] | 0.559 |  |
| 2016 | 36 | 72.2 | [56–84.2] | 1.99 | [0.74–5.34] | 0.559 |  |
| 2017 | 22 | 59.1 | [38.7–76.7] | 0.58 | [0.18–1.83] | 0.559 |  |
| 2018 | 41 | 65.9 | [50.5–78.4] | 1.26 | [0.42–3.81] | 0.778 |  |
| 2019 | 18 | 50.0 | [29–71] | 0.5 | [0.16–1.59] | 0.559 |  |
| 2020 | 19 | 21.1 | [8.5–43.3] | 0.29 | [0.07–1.26] | 0.559 |  |
| 2021 | 22 | 9.1 | [2.5–27.8] | 0.35 | [0.05–2.24] | 0.559 |  |
| 2022 | 21 | 19.0 | [7.7–40] | 2.42 | [0.38–15.58] | 0.559 |  |
| 2023 | 15 | 13.3 | [3.7–37.9] | 0.65 | [0.1–4.29] | 0.778 |  |
| 2024 | 25 | 8.0 | [2.2–25] | 0.6 | [0.07–5.05] | 0.778 |  |

### Table 139: Annual percentage of *Stenotrophomonas maltophilia* resistance to Levofloxacin from 2005 to 2024.

|  | Tested | Resistance | | Logistic model | |  | |
| --- | --- | --- | --- | --- | --- | --- | --- |
| Year | N | % | [95% CI] | OR | [95% CI] | P-value |  |
| 2005 | 0 | 0.0 | [NaN–NaN] | – | – | – |  |
| 2006 | 0 | 0.0 | [NaN–NaN] | – | – | – |  |
| 2007 | 0 | 0.0 | [NaN–NaN] | – | – | – |  |
| 2008 | 0 | 0.0 | [NaN–NaN] | – | – | – |  |
| 2009 | 0 | 0.0 | [NaN–NaN] | – | – | – |  |
| 2010 | 0 | 0.0 | [NaN–NaN] | – | – | – |  |
| 2011 | 11 | 9.1 | [1.6–37.7] | – | – | – |  |
| 2012 | 20 | 10.0 | [2.8–30.1] | 1.16 | [0.09–14.5] | 0.946 |  |
| 2013 | 35 | 8.6 | [3–22.4] | 0.83 | [0.13–5.54] | 0.946 |  |
| 2014 | 38 | 23.7 | [13–39.2] | 3.2 | [0.78–13.06] | 0.685 |  |
| 2015 | 37 | 18.9 | [9.5–34.2] | 0.71 | [0.23–2.19] | 0.946 |  |
| 2016 | 36 | 13.9 | [6.1–28.7] | 0.74 | [0.21–2.59] | 0.946 |  |
| 2017 | 22 | 13.6 | [4.7–33.3] | 1.06 | [0.22–4.98] | 0.946 |  |
| 2018 | 43 | 14.0 | [6.6–27.3] | 0.84 | [0.18–3.82] | 0.946 |  |
| 2019 | 21 | 9.5 | [2.7–28.9] | 0.76 | [0.14–4.16] | 0.946 |  |
| 2020 | 20 | 20.0 | [8.1–41.6] | 1.99 | [0.32–12.51] | 0.946 |  |
| 2021 | 25 | 16.0 | [6.4–34.7] | 0.84 | [0.18–3.9] | 0.946 |  |
| 2022 | 21 | 9.5 | [2.7–28.9] | 0.56 | [0.09–3.42] | 0.946 |  |
| 2023 | 15 | 26.7 | [10.9–52] | 3.21 | [0.5–20.62] | 0.946 |  |
| 2024 | 25 | 4.0 | [0.7–19.5] | 0.12 | [0.01–1.24] | 0.685 |  |

### Table S140: Annual percentage of *Stenotrophomonas maltophilia* resistance to Ticarcillin/Clavulanic Acid from 2005 to 2024.

|  | Tested | Resistance | | Logistic model | |  | |
| --- | --- | --- | --- | --- | --- | --- | --- |
| Year | N | % | [95% CI] | OR | [95% CI] | P-value |  |
| 2005 | 26 | 15.4 | [6.2–33.5] | – | – | – |  |
| 2006 | 23 | 13.0 | [4.5–32.1] | 0.7 | [0.15–3.23] | 0.992 |  |
| 2007 | 30 | 0.0 | [0–11.4] | – | – | – |  |
| 2008 | 51 | 5.9 | [2–15.9] | – | – | – |  |
| 2009 | 34 | 17.6 | [8.3–33.5] | 3.87 | [0.98–15.27] | 0.285 |  |
| 2010 | 38 | 18.4 | [9.2–33.4] | 0.86 | [0.28–2.68] | 0.992 |  |
| 2011 | 27 | 14.8 | [5.9–32.5] | 0.78 | [0.22–2.76] | 0.992 |  |
| 2012 | 21 | 33.3 | [17.2–54.6] | 3.57 | [0.95–13.45] | 0.285 |  |
| 2013 | 47 | 23.4 | [13.6–37.2] | 0.48 | [0.16–1.42] | 0.579 |  |
| 2014 | 46 | 2.2 | [0.4–11.3] | 0.08 | [0.01–0.54] | 0.186 |  |
| 2015 | 40 | 5.0 | [1.4–16.5] | 2.18 | [0.22–21.17] | 0.992 |  |
| 2016 | 36 | 19.4 | [9.8–35] | 5.19 | [1.11–24.23] | 0.285 |  |
| 2017 | 23 | 4.3 | [0.8–21] | 0.22 | [0.03–1.64] | 0.526 |  |
| 2018 | 42 | 14.3 | [6.7–27.8] | 2.67 | [0.35–20.58] | 0.942 |  |
| 2019 | 20 | 10.0 | [2.8–30.1] | 0.78 | [0.16–3.83] | 0.992 |  |
| 2020 | 19 | 15.8 | [5.5–37.6] | 1.5 | [0.25–9.06] | 0.992 |  |
| 2021 | 24 | 0.0 | [0–13.8] | – | – | – |  |
| 2022 | 20 | 15.0 | [5.2–36] | – | – | – |  |
| 2023 | 15 | 0.0 | [0–20.4] | – | – | – |  |
| 2024 | 25 | 4.0 | [0.7–19.5] | – | – | – |  |

### Table S141: Annual percentage of *Stenotrophomonas maltophilia* resistance to Cotrimoxazole from 2005 to 2024.

|  | Tested | Resistance | | Logistic model | |  | |
| --- | --- | --- | --- | --- | --- | --- | --- |
| Year | N | % | [95% CI] | OR | [95% CI] | P-value |  |
| 2005 | 26 | 11.5 | [4–29] | – | – | – |  |
| 2006 | 23 | 8.7 | [2.4–26.8] | 0.79 | [0.14–4.46] | 1.000 |  |
| 2007 | 31 | 6.5 | [1.8–20.7] | 0.67 | [0.11–4.27] | 1.000 |  |
| 2008 | 51 | 7.8 | [3.1–18.5] | 1.28 | [0.26–6.3] | 1.000 |  |
| 2009 | 34 | 0.0 | [0–10.2] | – | – | – |  |
| 2010 | 38 | 0.0 | [0–9.2] | – | – | – |  |
| 2011 | 27 | 3.7 | [0.7–18.3] | – | – | – |  |
| 2012 | 21 | 9.5 | [2.7–28.9] | 3.04 | [0.32–28.49] | 1.000 |  |
| 2013 | 47 | 0.0 | [0–7.6] | – | – | – |  |
| 2014 | 46 | 2.2 | [0.4–11.3] | – | – | – |  |
| 2015 | 40 | 5.0 | [1.4–16.5] | 2.3 | [0.25–20.79] | 1.000 |  |
| 2016 | 36 | 2.8 | [0.5–14.2] | 0.56 | [0.06–5.12] | 1.000 |  |
| 2017 | 22 | 22.7 | [10.1–43.4] | 10.94 | [1.46–82.05] | 0.189 |  |
| 2018 | 42 | 2.4 | [0.4–12.3] | 0.07 | [0.01–0.54] | 0.189 |  |
| 2019 | 21 | 4.8 | [0.8–22.7] | 2.32 | [0.18–29.72] | 1.000 |  |
| 2020 | 20 | 5.0 | [0.9–23.6] | 0.91 | [0.07–11.9] | 1.000 |  |
| 2021 | 22 | 4.5 | [0.8–21.8] | 0.97 | [0.07–12.61] | 1.000 |  |
| 2022 | 21 | 4.8 | [0.8–22.7] | 1.07 | [0.08–13.96] | 1.000 |  |
| 2023 | 13 | 23.1 | [8.2–50.3] | 5.67 | [0.65–49.2] | 0.547 |  |
| 2024 | 25 | 4.0 | [0.7–19.5] | 0.15 | [0.02–1.27] | 0.514 |  |

### Figure S16: Annual percentage of *Pseudomonas aeruginosa* resistance from 2005 to 2024


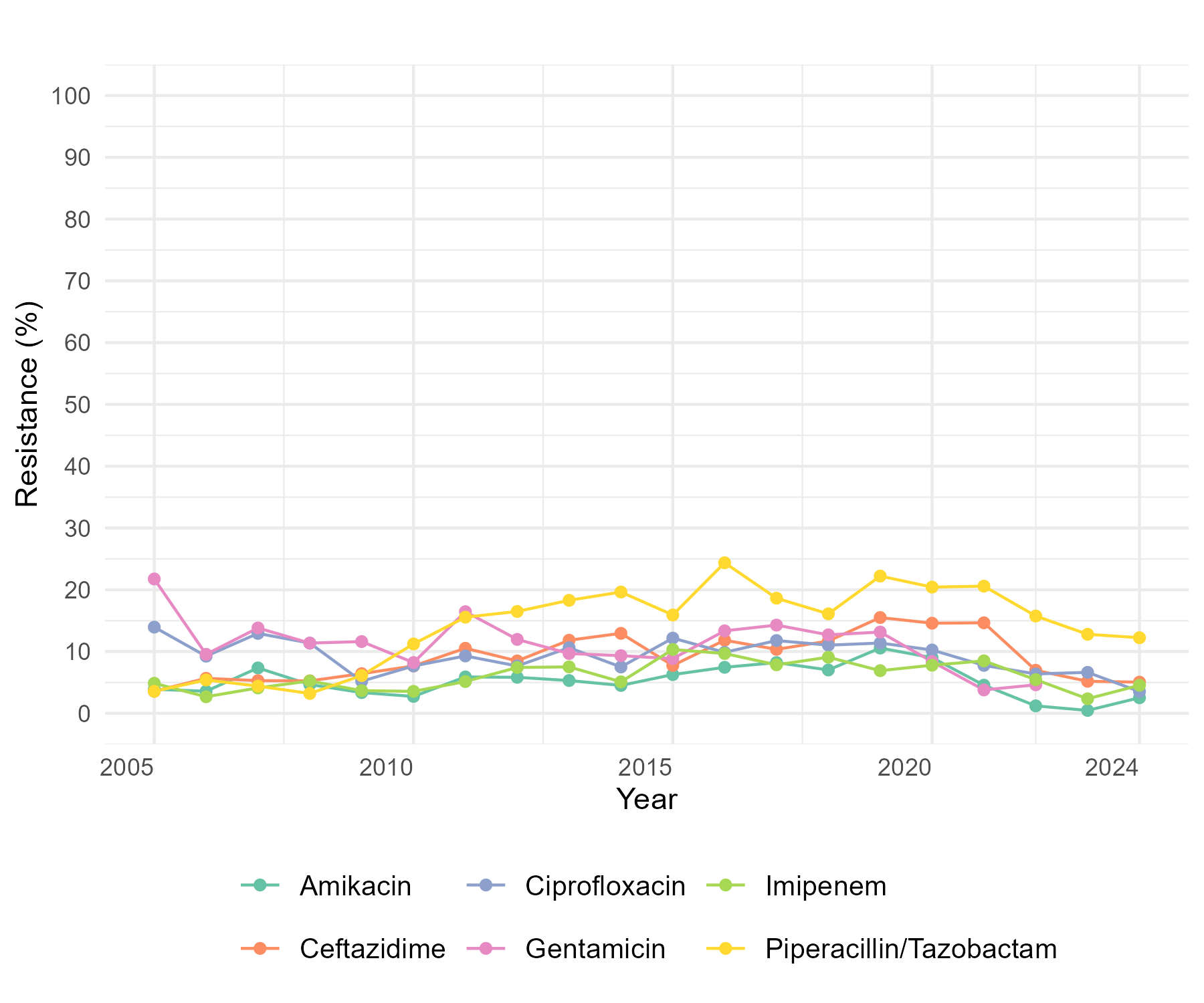


### Table S142: Logistic regression analysis of the effect of continuous time on the antibiotic resistance of *Pseudomonas aeruginosa* from 2005 to 2024.

| Antibiotic | β | OR | [95%CI] | P-value |  |
| --- | --- | --- | --- | --- | --- |
| Amikacin | 0.000 | 1.00 | [0.98–1.02] | 0.968 |  |
| Ceftazidime | 0.037 | 1.04 | [1.02–1.05] | 0.000 | *** |
| Ciprofloxacin | -0.022 | 0.98 | [0.96–0.99] | 0.005 | ** |
| Gentamicin | -0.042 | 0.96 | [0.94–0.98] | 0.000 | *** |
| Imipenem | 0.029 | 1.03 | [1.01–1.05] | 0.003 | ** |
| Piperacillin/Tazobactam | 0.072 | 1.07 | [1.06–1.09] | 0.000 | *** |

### Table S143: Annual percentage of *Pseudomonas aeruginosa* resistance to Amikacin from 2005 to 2024.

|  | Tested | Resistance | | Logistic model | |  | |
| --- | --- | --- | --- | --- | --- | --- | --- |
| Year | N | % | [95% CI] | OR | [95% CI] | P-value |  |
| 2005 | 308 | 3.9 | [2.2–6.7] | – | – | – |  |
| 2006 | 335 | 3.6 | [2.1–6.2] | 0.87 | [0.39–1.95] | 0.830 |  |
| 2007 | 340 | 7.4 | [5–10.6] | 2.16 | [1.08–4.35] | 0.190 |  |
| 2008 | 343 | 4.7 | [2.9–7.4] | 0.63 | [0.33–1.19] | 0.481 |  |
| 2009 | 327 | 3.4 | [1.9–5.9] | 0.74 | [0.34–1.61] | 0.777 |  |
| 2010 | 365 | 2.7 | [1.5–5] | 0.75 | [0.32–1.78] | 0.824 |  |
| 2011 | 407 | 5.9 | [4–8.6] | 2.33 | [1.11–4.9] | 0.190 |  |
| 2012 | 377 | 5.8 | [3.9–8.7] | 0.95 | [0.53–1.72] | 0.878 |  |
| 2013 | 320 | 5.3 | [3.3–8.3] | 0.91 | [0.48–1.74] | 0.830 |  |
| 2014 | 332 | 4.5 | [2.8–7.3] | 0.84 | [0.41–1.69] | 0.830 |  |
| 2015 | 271 | 6.3 | [4–9.8] | 1.39 | [0.68–2.81] | 0.777 |  |
| 2016 | 322 | 7.5 | [5.1–10.9] | 1.28 | [0.68–2.43] | 0.777 |  |
| 2017 | 280 | 8.2 | [5.5–12] | 1.09 | [0.6–1.97] | 0.830 |  |
| 2018 | 299 | 7.0 | [4.6–10.5] | 0.85 | [0.46–1.56] | 0.830 |  |
| 2019 | 246 | 10.6 | [7.3–15] | 1.41 | [0.78–2.57] | 0.694 |  |
| 2020 | 233 | 9.0 | [6–13.4] | 0.9 | [0.49–1.64] | 0.830 |  |
| 2021 | 307 | 4.6 | [2.7–7.5] | 0.48 | [0.24–0.97] | 0.190 |  |
| 2022 | 330 | 1.2 | [0.5–3.1] | 0.24 | [0.08–0.74] | 0.190 |  |
| 2023 | 210 | 0.5 | [0.1–2.6] | 0.4 | [0.05–3.52] | 0.777 |  |
| 2024 | 198 | 2.5 | [1.1–5.8] | 5.62 | [0.67–47.3] | 0.426 |  |

### Table S144: Annual percentage of *Pseudomonas aeruginosa* resistance to Ceftazidime from 2005 to 2024.

|  | Tested | Resistance | | Logistic model | |  | |
| --- | --- | --- | --- | --- | --- | --- | --- |
| Year | N | % | [95% CI] | OR | [95% CI] | P-value |  |
| 2005 | 308 | 3.6 | [2–6.3] | – | – | – |  |
| 2006 | 335 | 5.7 | [3.7–8.7] | 1.6 | [0.75–3.43] | 0.688 |  |
| 2007 | 340 | 5.3 | [3.4–8.2] | 0.93 | [0.48–1.81] | 1.000 |  |
| 2008 | 342 | 5.3 | [3.4–8.2] | 1 | [0.51–1.96] | 1.000 |  |
| 2009 | 327 | 6.4 | [4.2–9.6] | 1.26 | [0.66–2.41] | 0.896 |  |
| 2010 | 365 | 7.7 | [5.4–10.9] | 1.18 | [0.66–2.13] | 0.896 |  |
| 2011 | 408 | 10.5 | [7.9–13.9] | 1.43 | [0.87–2.36] | 0.607 |  |
| 2012 | 377 | 8.5 | [6.1–11.7] | 0.78 | [0.48–1.26] | 0.735 |  |
| 2013 | 321 | 11.8 | [8.7–15.8] | 1.45 | [0.88–2.38] | 0.607 |  |
| 2014 | 332 | 13.0 | [9.8–17] | 1.11 | [0.7–1.77] | 0.896 |  |
| 2015 | 271 | 7.7 | [5.1–11.6] | 0.56 | [0.32–0.97] | 0.352 |  |
| 2016 | 321 | 11.8 | [8.7–15.8] | 1.63 | [0.93–2.86] | 0.557 |  |
| 2017 | 280 | 10.4 | [7.3–14.5] | 0.87 | [0.52–1.45] | 0.896 |  |
| 2018 | 299 | 11.7 | [8.5–15.8] | 1.13 | [0.67–1.91] | 0.896 |  |
| 2019 | 245 | 15.5 | [11.5–20.6] | 1.34 | [0.81–2.19] | 0.688 |  |
| 2020 | 233 | 14.6 | [10.6–19.7] | 0.97 | [0.59–1.61] | 1.000 |  |
| 2021 | 307 | 14.7 | [11.1–19.1] | 1 | [0.61–1.61] | 1.000 |  |
| 2022 | 330 | 7.0 | [4.7–10.2] | 0.43 | [0.25–0.73] | 0.033 | * |
| 2023 | 212 | 5.2 | [2.9–9.1] | 0.75 | [0.36–1.57] | 0.896 |  |
| 2024 | 198 | 5.1 | [2.8–9] | 0.98 | [0.41–2.37] | 1.000 |  |

### Table S145: Annual percentage of *Pseudomonas aeruginosa* resistance to Ciprofloxacin from 2005 to 2024.

|  | Tested | Resistance | | Logistic model | |  | |
| --- | --- | --- | --- | --- | --- | --- | --- |
| Year | N | % | [95% CI] | OR | [95% CI] | P-value |  |
| 2005 | 308 | 14.0 | [10.5–18.3] | – | – | – |  |
| 2006 | 335 | 9.3 | [6.6–12.8] | 0.6 | [0.37–0.99] | 0.405 |  |
| 2007 | 340 | 12.9 | [9.8–16.9] | 1.47 | [0.9–2.39] | 0.472 |  |
| 2008 | 343 | 11.4 | [8.4–15.2] | 0.88 | [0.55–1.39] | 0.730 |  |
| 2009 | 327 | 5.2 | [3.3–8.2] | 0.44 | [0.24–0.8] | 0.132 |  |
| 2010 | 365 | 7.7 | [5.4–10.9] | 1.43 | [0.77–2.67] | 0.616 |  |
| 2011 | 409 | 9.3 | [6.8–12.5] | 1.27 | [0.76–2.12] | 0.620 |  |
| 2012 | 378 | 7.7 | [5.4–10.8] | 0.79 | [0.48–1.31] | 0.620 |  |
| 2013 | 320 | 10.6 | [7.7–14.5] | 1.44 | [0.86–2.43] | 0.472 |  |
| 2014 | 333 | 7.5 | [5.1–10.8] | 0.68 | [0.39–1.17] | 0.472 |  |
| 2015 | 271 | 12.2 | [8.8–16.6] | 1.68 | [0.97–2.9] | 0.405 |  |
| 2016 | 324 | 9.9 | [7.1–13.6] | 0.83 | [0.49–1.39] | 0.640 |  |
| 2017 | 280 | 11.8 | [8.5–16.1] | 1.22 | [0.73–2.05] | 0.640 |  |
| 2018 | 299 | 11.0 | [8–15.1] | 0.92 | [0.55–1.53] | 0.878 |  |
| 2019 | 246 | 11.4 | [8–16] | 0.95 | [0.56–1.63] | 0.889 |  |
| 2020 | 234 | 10.3 | [7–14.8] | 0.96 | [0.54–1.71] | 0.889 |  |
| 2021 | 309 | 7.8 | [5.3–11.3] | 0.73 | [0.4–1.33] | 0.620 |  |
| 2022 | 329 | 6.4 | [4.2–9.6] | 0.78 | [0.42–1.43] | 0.640 |  |
| 2023 | 211 | 6.6 | [4–10.8] | 1.08 | [0.54–2.18] | 0.889 |  |
| 2024 | 198 | 3.5 | [1.7–7.1] | 0.52 | [0.21–1.33] | 0.472 |  |

### Table S146: Annual percentage of *Pseudomonas aeruginosa* resistance to Gentamicin from 2005 to 2024.

|  | Tested | Resistance | | Logistic model | |  | |
| --- | --- | --- | --- | --- | --- | --- | --- |
| Year | N | % | [95% CI] | OR | [95% CI] | P-value |  |
| 2005 | 308 | 21.8 | [17.5–26.7] | – | – | – |  |
| 2006 | 335 | 9.6 | [6.8–13.2] | 0.36 | [0.23–0.57] | 0.000 | *** |
| 2007 | 340 | 13.8 | [10.6–17.9] | 1.54 | [0.95–2.48] | 0.212 |  |
| 2008 | 343 | 11.4 | [8.4–15.2] | 0.81 | [0.51–1.27] | 0.609 |  |
| 2009 | 327 | 11.6 | [8.6–15.5] | 1.06 | [0.66–1.71] | 0.913 |  |
| 2010 | 365 | 8.2 | [5.8–11.5] | 0.64 | [0.39–1.07] | 0.212 |  |
| 2011 | 407 | 16.5 | [13.2–20.4] | 2.28 | [1.44–3.6] | 0.003 | ** |
| 2012 | 376 | 12.0 | [9.1–15.6] | 0.67 | [0.45–1.01] | 0.198 |  |
| 2013 | 320 | 9.7 | [6.9–13.4] | 0.79 | [0.49–1.29] | 0.609 |  |
| 2014 | 332 | 9.3 | [6.7–12.9] | 0.95 | [0.57–1.61] | 0.913 |  |
| 2015 | 271 | 8.9 | [6–12.8] | 0.93 | [0.53–1.62] | 0.913 |  |
| 2016 | 322 | 13.4 | [10.1–17.5] | 1.67 | [0.98–2.83] | 0.198 |  |
| 2017 | 280 | 14.3 | [10.7–18.9] | 1.07 | [0.67–1.71] | 0.913 |  |
| 2018 | 299 | 12.7 | [9.4–17] | 0.87 | [0.54–1.4] | 0.878 |  |
| 2019 | 228 | 13.2 | [9.4–18.2] | 0.97 | [0.58–1.62] | 0.913 |  |
| 2020 | 203 | 8.4 | [5.3–13] | 0.64 | [0.34–1.19] | 0.340 |  |
| 2021 | 290 | 3.8 | [2.1–6.7] | 0.43 | [0.2–0.93] | 0.188 |  |
| 2022 | 238 | 4.6 | [2.6–8.1] | 1.19 | [0.51–2.78] | 0.913 |  |
| 2023 | 0 | 0.0 | [NaN–NaN] | – | – | – |  |
| 2024 | 0 | 0.0 | [NaN–NaN] | – | – | – |  |

### Table S147: Annual percentage of *Pseudomonas aeruginosa* resistance to Imipenem from 2005 to 2024.

|  | Tested | Resistance | | Logistic model | |  | |
| --- | --- | --- | --- | --- | --- | --- | --- |
| Year | N | % | [95% CI] | OR | [95% CI] | P-value |  |
| 2005 | 308 | 4.9 | [3–7.9] | – | – | – |  |
| 2006 | 335 | 2.7 | [1.4–5] | 0.53 | [0.23–1.23] | 0.582 |  |
| 2007 | 340 | 4.1 | [2.5–6.8] | 1.56 | [0.66–3.68] | 0.582 |  |
| 2008 | 343 | 5.2 | [3.3–8.1] | 1.3 | [0.63–2.67] | 0.692 |  |
| 2009 | 327 | 3.7 | [2.1–6.3] | 0.7 | [0.33–1.49] | 0.618 |  |
| 2010 | 365 | 3.6 | [2.1–6] | 0.94 | [0.42–2.09] | 0.924 |  |
| 2011 | 408 | 5.1 | [3.4–7.7] | 1.49 | [0.73–3.03] | 0.582 |  |
| 2012 | 377 | 7.4 | [5.2–10.5] | 1.46 | [0.81–2.63] | 0.582 |  |
| 2013 | 319 | 7.5 | [5.1–11] | 1.01 | [0.57–1.79] | 0.961 |  |
| 2014 | 333 | 5.1 | [3.2–8] | 0.66 | [0.35–1.26] | 0.582 |  |
| 2015 | 271 | 10.3 | [7.2–14.5] | 2.12 | [1.13–3.97] | 0.367 |  |
| 2016 | 320 | 9.7 | [6.9–13.4] | 0.95 | [0.55–1.64] | 0.924 |  |
| 2017 | 280 | 7.9 | [5.2–11.6] | 0.8 | [0.45–1.42] | 0.692 |  |
| 2018 | 297 | 9.1 | [6.3–12.9] | 1.16 | [0.64–2.1] | 0.785 |  |
| 2019 | 246 | 6.9 | [4.4–10.8] | 0.71 | [0.37–1.34] | 0.582 |  |
| 2020 | 231 | 7.8 | [5–12] | 1.19 | [0.6–2.39] | 0.785 |  |
| 2021 | 306 | 8.5 | [5.9–12.2] | 1.09 | [0.58–2.05] | 0.924 |  |
| 2022 | 330 | 5.5 | [3.5–8.5] | 0.61 | [0.33–1.14] | 0.582 |  |
| 2023 | 212 | 2.4 | [1–5.4] | 0.43 | [0.16–1.18] | 0.582 |  |
| 2024 | 198 | 4.5 | [2.4–8.4] | 1.99 | [0.65–6.09] | 0.582 |  |

### Table S148: Annual percentage of *Pseudomonas aeruginosa* resistance to Piperacillin/Tazobactam from 2005 to 2024.

|  | Tested | Resistance | | Logistic model | |  | |
| --- | --- | --- | --- | --- | --- | --- | --- |
| Year | N | % | [95% CI] | OR | [95% CI] | P-value |  |
| 2005 | 308 | 3.6 | [2–6.3] | – | – | – |  |
| 2006 | 333 | 5.4 | [3.4–8.4] | 1.53 | [0.71–3.3] | 0.593 |  |
| 2007 | 340 | 4.4 | [2.7–7.2] | 0.81 | [0.4–1.64] | 0.761 |  |
| 2008 | 342 | 3.2 | [1.8–5.7] | 0.73 | [0.33–1.61] | 0.679 |  |
| 2009 | 327 | 6.1 | [4–9.3] | 2 | [0.94–4.26] | 0.333 |  |
| 2010 | 365 | 11.2 | [8.4–14.9] | 1.9 | [1.08–3.32] | 0.237 |  |
| 2011 | 212 | 15.6 | [11.3–21.1] | 1.47 | [0.9–2.42] | 0.343 |  |
| 2012 | 194 | 16.5 | [11.9–22.4] | 1.04 | [0.61–1.78] | 0.934 |  |
| 2013 | 317 | 18.3 | [14.4–22.9] | 1.15 | [0.72–1.86] | 0.761 |  |
| 2014 | 331 | 19.6 | [15.7–24.3] | 1.1 | [0.74–1.63] | 0.825 |  |
| 2015 | 270 | 15.9 | [12–20.8] | 0.76 | [0.5–1.17] | 0.508 |  |
| 2016 | 320 | 24.4 | [20–29.4] | 1.73 | [1.14–2.63] | 0.186 |  |
| 2017 | 279 | 18.6 | [14.5–23.6] | 0.72 | [0.48–1.07] | 0.333 |  |
| 2018 | 298 | 16.1 | [12.4–20.7] | 0.82 | [0.53–1.27] | 0.679 |  |
| 2019 | 243 | 22.2 | [17.5–27.9] | 1.44 | [0.93–2.23] | 0.333 |  |
| 2020 | 230 | 20.4 | [15.7–26.1] | 0.94 | [0.61–1.47] | 0.934 |  |
| 2021 | 306 | 20.6 | [16.4–25.5] | 0.99 | [0.65–1.52] | 0.981 |  |
| 2022 | 330 | 15.8 | [12.2–20.1] | 0.71 | [0.47–1.07] | 0.333 |  |
| 2023 | 211 | 12.8 | [8.9–18] | 0.81 | [0.49–1.34] | 0.679 |  |
| 2024 | 196 | 12.2 | [8.4–17.6] | 0.96 | [0.53–1.73] | 0.934 |  |

## Other Gram-Negatives

### Figure S17: Annual percentage of *Haemophilus influenzae* resistance from 2005 to 2024.


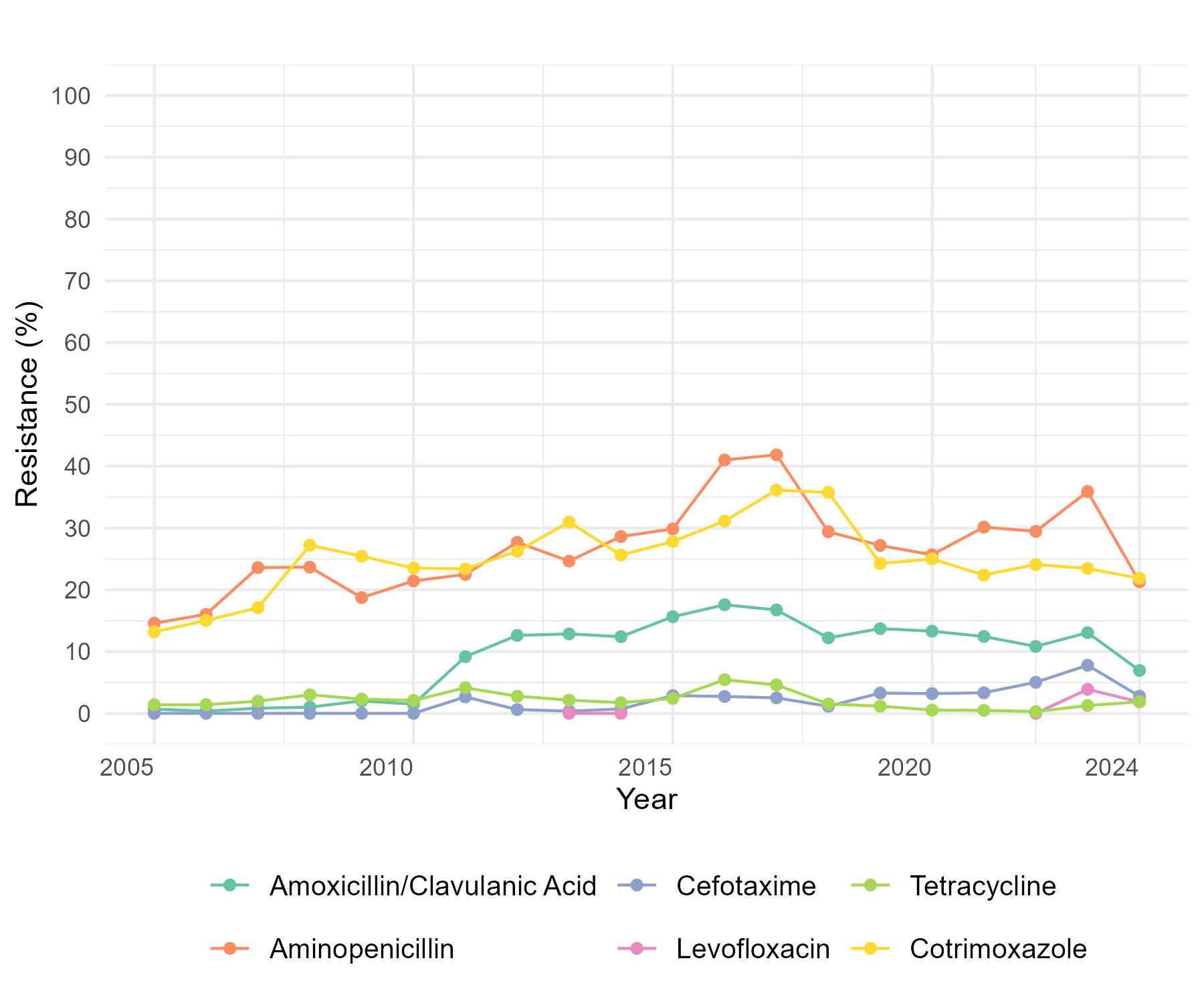


### Table S149: Logistic regression analysis of the effect of continuous time on the antibiotic resistance of *Haemophilus influenzae* from 2005 to 2024.

| Antibiotic | β | OR | [95%CI] | P-value |  |
| --- | --- | --- | --- | --- | --- |
| Amoxicillin/Clavulanic Acid | 0.093 | 1.10 | [1.08–1.12] | 0.000 | *** |
| Aminopenicillin | 0.039 | 1.04 | [1.03–1.05] | 0.000 | *** |
| Cefotaxime | 0.154 | 1.17 | [1.11–1.22] | 0.000 | *** |
| Levofloxacin | 0.004 | 1.00 | [0.91–1.1] | 0.927 |  |
| Tetracycline | -0.027 | 0.97 | [0.94–1.01] | 0.100 | . |
| Cotrimoxazole | 0.022 | 1.02 | [1.01–1.03] | 0.000 | *** |

### Table S150: Annual percentage of *Haemophilus influenzae* resistance to Aminopenicillin from 2005 to 2024.

|  | Tested | Resistance | | Logistic model | |  | |
| --- | --- | --- | --- | --- | --- | --- | --- |
| Year | N | % | [95% CI] | OR | [95% CI] | P-value |  |
| 2005 | 288 | 14.6 | [11–19.1] | – | – | – |  |
| 2006 | 287 | 16.0 | [12.2–20.7] | 1.13 | [0.71–1.78] | 0.889 |  |
| 2007 | 356 | 23.6 | [19.5–28.3] | 1.61 | [1.08–2.4] | 0.097 | . |
| 2008 | 300 | 23.7 | [19.2–28.8] | 1 | [0.7–1.44] | 0.994 |  |
| 2009 | 347 | 18.7 | [15–23.2] | 0.74 | [0.51–1.08] | 0.353 |  |
| 2010 | 336 | 21.4 | [17.4–26.1] | 1.18 | [0.81–1.72] | 0.674 |  |
| 2011 | 338 | 22.5 | [18.4–27.2] | 1.07 | [0.74–1.54] | 0.910 |  |
| 2012 | 325 | 27.7 | [23.1–32.8] | 1.31 | [0.92–1.87] | 0.353 |  |
| 2013 | 280 | 24.6 | [20–30] | 0.85 | [0.59–1.23] | 0.674 |  |
| 2014 | 290 | 28.6 | [23.7–34.1] | 1.23 | [0.85–1.79] | 0.639 |  |
| 2015 | 211 | 29.9 | [24.1–36.3] | 1.06 | [0.72–1.57] | 0.910 |  |
| 2016 | 256 | 41.0 | [35.2–47.1] | 1.63 | [1.11–2.4] | 0.083 | . |
| 2017 | 239 | 41.8 | [35.8–48.2] | 1.03 | [0.72–1.48] | 0.913 |  |
| 2018 | 262 | 29.4 | [24.2–35.2] | 0.58 | [0.4–0.84] | 0.041 | * |
| 2019 | 276 | 27.2 | [22.3–32.7] | 0.9 | [0.62–1.31] | 0.889 |  |
| 2020 | 187 | 25.7 | [19.9–32.4] | 0.93 | [0.61–1.41] | 0.910 |  |
| 2021 | 209 | 30.1 | [24.3–36.7] | 1.26 | [0.81–1.97] | 0.639 |  |
| 2022 | 360 | 29.4 | [25–34.3] | 0.96 | [0.66–1.39] | 0.913 |  |
| 2023 | 337 | 35.9 | [31–41.2] | 1.34 | [0.98–1.85] | 0.267 |  |
| 2024 | 216 | 21.3 | [16.4–27.2] | 0.49 | [0.33–0.72] | 0.007 | ** |

### Table S151: Annual percentage of *Haemophilus influenzae* resistance to Amoxicillin/Clavulanic Acid from 2005 to 2024.

|  | Tested | Resistance | | Logistic model | |  | |
| --- | --- | --- | --- | --- | --- | --- | --- |
| Year | N | % | [95% CI] | OR | [95% CI] | P-value |  |
| 2005 | 288 | 0.7 | [0.2–2.5] | – | – | – |  |
| 2006 | 287 | 0.3 | [0.1–1.9] | 0.5 | [0.04–5.62] | 0.864 |  |
| 2007 | 356 | 0.8 | [0.3–2.4] | 2.42 | [0.25–23.64] | 0.864 |  |
| 2008 | 300 | 1.0 | [0.3–2.9] | 1.18 | [0.24–5.95] | 0.935 |  |
| 2009 | 347 | 2.0 | [1–4.1] | 2.04 | [0.52–8.02] | 0.864 |  |
| 2010 | 336 | 1.5 | [0.6–3.4] | 0.73 | [0.23–2.33] | 0.864 |  |
| 2011 | 338 | 9.2 | [6.5–12.7] | 6.74 | [2.58–17.63] | 0.002 | ** |
| 2012 | 325 | 12.6 | [9.4–16.7] | 1.43 | [0.87–2.34] | 0.759 |  |
| 2013 | 280 | 12.9 | [9.4–17.3] | 1.02 | [0.63–1.65] | 0.942 |  |
| 2014 | 290 | 12.4 | [9.1–16.7] | 0.96 | [0.58–1.58] | 0.935 |  |
| 2015 | 211 | 15.6 | [11.4–21.1] | 1.3 | [0.78–2.18] | 0.864 |  |
| 2016 | 256 | 17.6 | [13.4–22.7] | 1.15 | [0.7–1.89] | 0.864 |  |
| 2017 | 239 | 16.7 | [12.5–22] | 0.95 | [0.59–1.51] | 0.935 |  |
| 2018 | 262 | 12.2 | [8.8–16.7] | 0.69 | [0.42–1.14] | 0.759 |  |
| 2019 | 277 | 13.7 | [10.2–18.3] | 1.15 | [0.7–1.92] | 0.864 |  |
| 2020 | 188 | 13.3 | [9.2–18.9] | 0.96 | [0.56–1.66] | 0.935 |  |
| 2021 | 209 | 12.4 | [8.6–17.6] | 0.93 | [0.51–1.68] | 0.935 |  |
| 2022 | 360 | 10.8 | [8–14.5] | 0.85 | [0.5–1.45] | 0.864 |  |
| 2023 | 337 | 13.1 | [9.9–17.1] | 1.25 | [0.79–1.98] | 0.864 |  |
| 2024 | 216 | 6.9 | [4.3–11.1] | 0.49 | [0.27–0.91] | 0.233 |  |

### Table S152: Annual percentage of *Haemophilus influenzae* resistance to Cefotaxime from 2005 to 2024.

|  | Tested | Resistance | | Logistic model | |  | |
| --- | --- | --- | --- | --- | --- | --- | --- |
| Year | N | % | [95% CI] | OR | [95% CI] | P-value |  |
| 2005 | 158 | 0.0 | [0–2.4] | – | – | – |  |
| 2006 | 43 | 0.0 | [0–8.2] | – | – | – |  |
| 2007 | 33 | 0.0 | [0–10.4] | – | – | – |  |
| 2008 | 54 | 0.0 | [0–6.6] | – | – | – |  |
| 2009 | 29 | 0.0 | [0–11.7] | – | – | – |  |
| 2010 | 97 | 0.0 | [0–3.8] | – | – | – |  |
| 2011 | 338 | 2.7 | [1.4–5] | – | – | – |  |
| 2012 | 323 | 0.6 | [0.2–2.2] | 0.23 | [0.05–0.97] | 0.416 |  |
| 2013 | 279 | 0.4 | [0.1–2] | 0.56 | [0.06–5.48] | 1.000 |  |
| 2014 | 281 | 0.7 | [0.2–2.6] | 2.06 | [0.21–20.25] | 1.000 |  |
| 2015 | 209 | 2.9 | [1.3–6.1] | 4.08 | [0.88–18.82] | 0.416 |  |
| 2016 | 256 | 2.7 | [1.3–5.5] | 0.95 | [0.33–2.72] | 1.000 |  |
| 2017 | 239 | 2.5 | [1.2–5.4] | 0.91 | [0.32–2.6] | 1.000 |  |
| 2018 | 262 | 1.1 | [0.4–3.3] | 0.46 | [0.12–1.74] | 0.806 |  |
| 2019 | 274 | 3.3 | [1.7–6.1] | 2.98 | [0.85–10.42] | 0.416 |  |
| 2020 | 188 | 3.2 | [1.5–6.8] | 0.97 | [0.36–2.62] | 1.000 |  |
| 2021 | 210 | 3.3 | [1.6–6.7] | 1.1 | [0.38–3.15] | 1.000 |  |
| 2022 | 358 | 5.0 | [3.2–7.8] | 1.46 | [0.63–3.41] | 1.000 |  |
| 2023 | 334 | 7.8 | [5.4–11.2] | 1.61 | [0.89–2.91] | 0.430 |  |
| 2024 | 216 | 2.8 | [1.3–5.9] | 0.35 | [0.15–0.82] | 0.299 |  |

### Table S153: Annual percentage of *Haemophilus influenzae* resistance to Levofloxacin from 2005 to 2024.

|  | Tested | Resistance | | Logistic model | |  | |
| --- | --- | --- | --- | --- | --- | --- | --- |
| Year | N | % | [95% CI] | OR | [95% CI] | P-value |  |
| 2005 | 10 | 0.0 | [0–27.8] | – | – | – |  |
| 2006 | 17 | 0.0 | [0–18.4] | – | – | – |  |
| 2007 | 14 | 0.0 | [0–21.5] | – | – | – |  |
| 2008 | 23 | 0.0 | [0–14.3] | – | – | – |  |
| 2009 | 11 | 0.0 | [0–25.9] | – | – | – |  |
| 2010 | 21 | 4.8 | [0.8–22.7] | – | – | – |  |
| 2011 | 19 | 5.3 | [0.9–24.6] | 1.12 | [0.07–17.37] | 1.000 |  |
| 2012 | 19 | 0.0 | [0–16.8] | – | – | – |  |
| 2013 | 10 | 20.0 | [5.7–51] | – | – | – |  |
| 2014 | 9 | 0.0 | [0–29.9] | – | – | – |  |
| 2015 | 3 | 0.0 | [0–56.1] | – | – | – |  |
| 2016 | 7 | 14.3 | [2.6–51.3] | – | – | – |  |
| 2017 | 8 | 0.0 | [0–32.4] | – | – | – |  |
| 2018 | 3 | 0.0 | [0–56.1] | – | – | – |  |
| 2019 | 223 | 1.8 | [0.7–4.5] | – | – | – |  |
| 2020 | 188 | 1.1 | [0.3–3.8] | 0.61 | [0.12–3.15] | 1.000 |  |
| 2021 | 210 | 1.9 | [0.7–4.8] | 1.77 | [0.34–9.16] | 1.000 |  |
| 2022 | 357 | 2.0 | [1–4] | 1.04 | [0.32–3.43] | 1.000 |  |
| 2023 | 333 | 1.5 | [0.6–3.5] | 0.7 | [0.23–2.14] | 1.000 |  |
| 2024 | 215 | 1.9 | [0.7–4.7] | 1.33 | [0.37–4.76] | 1.000 |  |

### Table S154: Annual percentage of *Haemophilus influenzae* resistance to Tetracycline from 2005 to 2024.

|  | Tested | Resistance | | Logistic model | |  | |
| --- | --- | --- | --- | --- | --- | --- | --- |
| Year | N | % | [95% CI] | OR | [95% CI] | P-value |  |
| 2005 | 287 | 1.4 | [0.5–3.5] | – | – | – |  |
| 2006 | 287 | 1.4 | [0.5–3.5] | 1.01 | [0.25–4.12] | 0.989 |  |
| 2007 | 356 | 2.0 | [1–4] | 1.41 | [0.4–4.91] | 0.865 |  |
| 2008 | 300 | 3.0 | [1.6–5.6] | 1.54 | [0.56–4.21] | 0.865 |  |
| 2009 | 347 | 2.3 | [1.2–4.5] | 0.76 | [0.29–2.01] | 0.865 |  |
| 2010 | 336 | 2.1 | [1–4.2] | 0.9 | [0.32–2.53] | 0.942 |  |
| 2011 | 338 | 4.1 | [2.5–6.8] | 2.03 | [0.8–5.13] | 0.854 |  |
| 2012 | 324 | 2.8 | [1.5–5.2] | 0.66 | [0.28–1.55] | 0.865 |  |
| 2013 | 280 | 2.1 | [1–4.6] | 0.76 | [0.27–2.18] | 0.865 |  |
| 2014 | 290 | 1.7 | [0.7–4] | 0.81 | [0.24–2.7] | 0.865 |  |
| 2015 | 207 | 2.4 | [1–5.5] | 1.41 | [0.4–4.99] | 0.865 |  |
| 2016 | 257 | 5.4 | [3.3–8.9] | 2.31 | [0.81–6.59] | 0.854 |  |
| 2017 | 239 | 4.6 | [2.6–8.1] | 0.83 | [0.37–1.88] | 0.865 |  |
| 2018 | 262 | 1.5 | [0.6–3.9] | 0.33 | [0.1–1.05] | 0.854 |  |
| 2019 | 258 | 1.2 | [0.4–3.4] | 0.76 | [0.17–3.47] | 0.865 |  |
| 2020 | 188 | 0.5 | [0.1–3] | 0.45 | [0.05–4.47] | 0.865 |  |
| 2021 | 205 | 0.5 | [0.1–2.7] | 0.93 | [0.06–15.33] | 0.989 |  |
| 2022 | 354 | 0.3 | [0–1.6] | 0.57 | [0.03–9.32] | 0.865 |  |
| 2023 | 318 | 1.3 | [0.5–3.2] | 4.49 | [0.49–41.06] | 0.865 |  |
| 2024 | 214 | 1.9 | [0.7–4.7] | 1.52 | [0.37–6.2] | 0.865 |  |

### Table S155: Annual percentage of *Haemophilus influenzae* resistance to Cotrimoxazole from 2005 to 2024.

|  | Tested | Resistance | | Logistic model | |  | |
| --- | --- | --- | --- | --- | --- | --- | --- |
| Year | N | % | [95% CI] | OR | [95% CI] | P-value |  |
| 2005 | 288 | 13.2 | [9.8–17.6] | – | – | – |  |
| 2006 | 286 | 15.0 | [11.4–19.6] | 1.18 | [0.73–1.89] | 0.890 |  |
| 2007 | 351 | 17.1 | [13.5–21.4] | 1.16 | [0.75–1.78] | 0.890 |  |
| 2008 | 283 | 27.2 | [22.4–32.7] | 1.8 | [1.23–2.65] | 0.040 | * |
| 2009 | 346 | 25.4 | [21.1–30.3] | 0.91 | [0.64–1.3] | 0.890 |  |
| 2010 | 336 | 23.5 | [19.3–28.3] | 0.9 | [0.63–1.27] | 0.890 |  |
| 2011 | 325 | 23.4 | [19.1–28.3] | 0.99 | [0.69–1.43] | 1.000 |  |
| 2012 | 305 | 26.2 | [21.6–31.4] | 1.16 | [0.81–1.67] | 0.890 |  |
| 2013 | 268 | 31.0 | [25.7–36.7] | 1.23 | [0.85–1.77] | 0.890 |  |
| 2014 | 281 | 25.6 | [20.9–31] | 0.79 | [0.54–1.15] | 0.890 |  |
| 2015 | 205 | 27.8 | [22.1–34.3] | 1.12 | [0.74–1.68] | 0.890 |  |
| 2016 | 257 | 31.1 | [25.8–37] | 1.17 | [0.78–1.75] | 0.890 |  |
| 2017 | 238 | 36.1 | [30.3–42.4] | 1.25 | [0.86–1.81] | 0.890 |  |
| 2018 | 260 | 35.8 | [30.2–41.8] | 1 | [0.69–1.44] | 1.000 |  |
| 2019 | 268 | 24.3 | [19.5–29.7] | 0.58 | [0.39–0.84] | 0.040 | * |
| 2020 | 188 | 25.0 | [19.4–31.6] | 1.04 | [0.67–1.61] | 0.958 |  |
| 2021 | 201 | 22.4 | [17.2–28.6] | 0.88 | [0.55–1.41] | 0.890 |  |
| 2022 | 357 | 24.1 | [19.9–28.8] | 1.08 | [0.71–1.63] | 0.922 |  |
| 2023 | 307 | 23.5 | [19.1–28.5] | 0.97 | [0.67–1.38] | 0.958 |  |
| 2024 | 215 | 21.9 | [16.9–27.9] | 0.93 | [0.61–1.41] | 0.922 |  |

### Figure S18: Annual percentage of *Neisseria gonorrhoeae* resistance from 2005 to 2024.


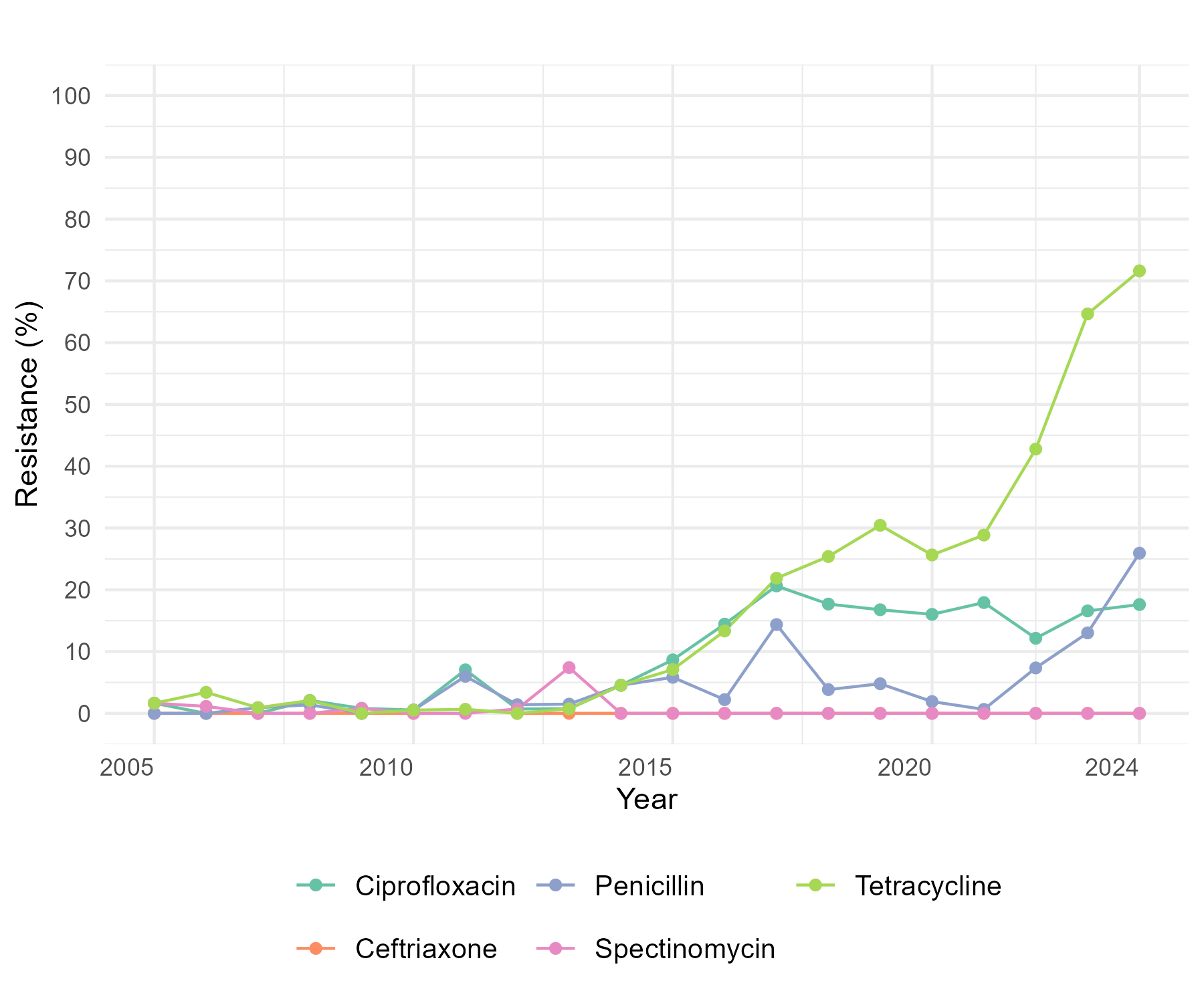


### Table S156: Logistic regression analysis of the effect of continuous time on the antibiotic resistance of *Neisseria gonorrhoeae* from 2005 to 2024.

| Antibiotic | β | OR | [95%CI] | P-value |  |
| --- | --- | --- | --- | --- | --- |
| Ciprofloxacin | 0.153 | 1.16 | [1.13–1.2] | 0.000 | *** |
| Cefotaxime | 0.000 | 1.00 | [0.99–1.01] | 1.000 |  |
| Penicillin | 0.131 | 1.14 | [1.09–1.19] | 0.000 | *** |
| Spectinomycin | -0.117 | 0.89 | [0.8–0.99] | 0.025 | * |
| Tetracycline | 0.351 | 1.42 | [1.37–1.48] | 0.000 | *** |

### Table S157: Annual percentage of *Neisseria gonorrhoeae* resistance to Ciprofloxacin from 2005 to 2024.

|  | Tested | Resistance | | Logistic model | |  | |
| --- | --- | --- | --- | --- | --- | --- | --- |
| Year | N | % | [95% CI] | OR | [95% CI] | P-value |  |
| 2005 | 61 | 1.6 | [0.3–8.7] | – | – | – |  |
| 2006 | 88 | 0.0 | [0–4.2] | – | – | – |  |
| 2007 | 109 | 0.0 | [0–3.4] | – | – | – |  |
| 2008 | 143 | 2.1 | [0.7–6] | – | – | – |  |
| 2009 | 124 | 0.8 | [0.1–4.4] | 0.37 | [0.04–3.08] | 0.749 |  |
| 2010 | 185 | 0.5 | [0.1–3] | 0.69 | [0.05–9.16] | 1.000 |  |
| 2011 | 142 | 7.0 | [3.9–12.5] | 13.99 | [2.04–95.83] | 0.137 |  |
| 2012 | 140 | 0.7 | [0.1–3.9] | 0.09 | [0.01–0.65] | 0.155 |  |
| 2013 | 134 | 0.7 | [0.1–4.1] | 1.08 | [0.08–14.35] | 1.000 |  |
| 2014 | 132 | 4.5 | [2.1–9.6] | 6.1 | [0.84–44.33] | 0.350 |  |
| 2015 | 127 | 8.7 | [4.9–14.8] | 1.81 | [0.69–4.79] | 0.548 |  |
| 2016 | 90 | 14.4 | [8.6–23.2] | 2.02 | [0.89–4.55] | 0.350 |  |
| 2017 | 160 | 20.6 | [15.1–27.5] | 1.57 | [0.82–3.04] | 0.476 |  |
| 2018 | 130 | 17.7 | [12.1–25.2] | 0.79 | [0.45–1.37] | 0.749 |  |
| 2019 | 167 | 16.8 | [11.9–23.2] | 0.9 | [0.51–1.59] | 1.000 |  |
| 2020 | 156 | 16.0 | [11.1–22.6] | 1 | [0.57–1.75] | 1.000 |  |
| 2021 | 145 | 17.9 | [12.5–25] | 1.09 | [0.61–1.97] | 1.000 |  |
| 2022 | 181 | 12.2 | [8.2–17.7] | 0.57 | [0.3–1.08] | 0.350 |  |
| 2023 | 199 | 16.6 | [12.1–22.4] | 1.6 | [0.89–2.89] | 0.372 |  |
| 2024 | 159 | 17.6 | [12.5–24.3] | 1.07 | [0.63–1.79] | 1.000 |  |

### Table S158: Annual percentage of *Neisseria gonorrhoeae* resistance to Ceftriaxone from 2005 to 2024.

|  | Tested | Resistance | | Logistic model | |  | |
| --- | --- | --- | --- | --- | --- | --- | --- |
| Year | N | % | [95% CI] | OR | [95% CI] | P-value |  |
| 2005 | 61 | 0 | [0–5.9] | – | – | – |  |
| 2006 | 88 | 0 | [0–4.2] | 1 | [0.72–1.39] | 1.000 |  |
| 2007 | 109 | 0 | [0–3.4] | 1 | [0.75–1.33] | 1.000 |  |
| 2008 | 140 | 0 | [0–2.7] | 1 | [0.78–1.29] | 1.000 |  |
| 2009 | 75 | 0 | [0–4.9] | 1 | [0.75–1.33] | 1.000 |  |
| 2010 | 3 | 0 | [0–56.1] | 1 | [0.31–3.19] | 1.000 |  |
| 2011 | 0 | 0 | – | – | – | – |  |
| 2012 | 1 | 0 | [0–79.3] | 1 | [0.1–9.73] | 1.000 |  |
| 2013 | 18 | 0 | [0–17.6] | 1 | [0.13–7.57] | 1.000 |  |
| 2014 | 129 | 0 | [0–2.9] | 1 | [0.61–1.64] | 1.000 |  |
| 2015 | 125 | 0 | [0–3] | 1 | [0.78–1.28] | 1.000 |  |
| 2016 | 88 | 0 | [0–4.2] | 1 | [0.76–1.32] | 1.000 |  |
| 2017 | 159 | 0 | [0–2.4] | 1 | [0.77–1.3] | 1.000 |  |
| 2018 | 130 | 0 | [0–2.9] | 1 | [0.79–1.26] | 1.000 |  |
| 2019 | 167 | 0 | [0–2.2] | 1 | [0.79–1.26] | 1.000 |  |
| 2020 | 157 | 0 | [0–2.4] | 1 | [0.8–1.25] | 1.000 |  |
| 2021 | 156 | 0 | [0–2.4] | 1 | [0.79–1.26] | 1.000 |  |
| 2022 | 205 | 0 | [0–1.8] | 1 | [0.8–1.25] | 1.000 |  |
| 2023 | 219 | 0 | [0–1.7] | 1 | [0.82–1.22] | 1.000 |  |
| 2024 | 161 | 0 | [0–2.3] | 1 | [0.81–1.23] | 1.000 |  |

### Table S159: Annual percentage of *Neisseria gonorrhoeae* resistance to Penicillin from 2005 to 2024.

|  | Tested | Resistance | | Logistic model | |  | |
| --- | --- | --- | --- | --- | --- | --- | --- |
| Year | N | % | [95% CI] | OR | [95% CI] | P-value |  |
| 2005 | 61 | 0.0 | [0–5.9] | – | – | – |  |
| 2006 | 88 | 0.0 | [0–4.2] | – | – | – |  |
| 2007 | 109 | 0.9 | [0.2–5] | – | – | – |  |
| 2008 | 144 | 1.4 | [0.4–4.9] | 1.5 | [0.16–14.05] | 1.000 |  |
| 2009 | 124 | 0.0 | [0–3] | – | – | – |  |
| 2010 | 191 | 0.5 | [0.1–2.9] | – | – | – |  |
| 2011 | 150 | 6.0 | [3.2–11] | 12.2 | [1.78–83.73] | 0.069 | . |
| 2012 | 142 | 1.4 | [0.4–5] | 0.22 | [0.05–0.93] | 0.127 |  |
| 2013 | 134 | 1.5 | [0.4–5.3] | 1.1 | [0.18–6.88] | 1.000 |  |
| 2014 | 132 | 4.5 | [2.1–9.6] | 3.06 | [0.68–13.76] | 0.390 |  |
| 2015 | 120 | 5.8 | [2.9–11.6] | 1.09 | [0.37–3.21] | 1.000 |  |
| 2016 | 90 | 2.2 | [0.6–7.7] | 0.45 | [0.1–2.01] | 0.620 |  |
| 2017 | 160 | 14.4 | [9.8–20.6] | 7.86 | [2.01–30.76] | 0.029 | * |
| 2018 | 130 | 3.8 | [1.7–8.7] | 0.23 | [0.09–0.57] | 0.029 | * |
| 2019 | 167 | 4.8 | [2.4–9.2] | 1.19 | [0.41–3.44] | 1.000 |  |
| 2020 | 157 | 1.9 | [0.7–5.5] | 0.43 | [0.12–1.5] | 0.437 |  |
| 2021 | 156 | 0.6 | [0.1–3.5] | – | – | – |  |
| 2022 | 204 | 7.4 | [4.5–11.8] | – | – | – |  |
| 2023 | 215 | 13.0 | [9.2–18.2] | 2.18 | [1.1–4.33] | 0.117 |  |
| 2024 | 27 | 25.9 | [13.2–44.7] | 2.68 | [1.1–6.54] | 0.117 |  |

### Table S160: Annual percentage of *Neisseria gonorrhoeae* resistance to Spectinomycin from 2005 to 2024.

|  | Tested | Resistance | | Logistic model | |  | |
| --- | --- | --- | --- | --- | --- | --- | --- |
| Year | N | % | [95% CI] | OR | [95% CI] | P-value |  |
| 2005 | 61 | 1.6 | [0.3–8.7] | – | – | – |  |
| 2006 | 88 | 1.1 | [0.2–6.2] | 0.78 | [0.24–2.5] | 1.000 |  |
| 2007 | 109 | 0.0 | [0–3.4] | – | – | – |  |
| 2008 | 144 | 0.0 | [0–2.6] | – | – | – |  |
| 2009 | 123 | 0.8 | [0.1–4.5] | – | – | – |  |
| 2010 | 191 | 0.0 | [0–2] | – | – | – |  |
| 2011 | 152 | 0.0 | [0–2.5] | – | – | – |  |
| 2012 | 142 | 0.7 | [0.1–3.9] | – | – | – |  |
| 2013 | 135 | 7.4 | [4.1–13.1] | 12.02 | [5.08–28.46] | 0.000 | *** |
| 2014 | 132 | 0.0 | [0–2.8] | – | – | – |  |
| 2015 | 127 | 0.0 | [0–2.9] | – | – | – |  |
| 2016 | 90 | 0.0 | [0–4.1] | – | – | – |  |
| 2017 | 147 | 0.0 | [0–2.5] | – | – | – |  |
| 2018 | 130 | 0.0 | [0–2.9] | – | – | – |  |
| 2019 | 167 | 0.0 | [0–2.2] | – | – | – |  |
| 2020 | 155 | 0.0 | [0–2.4] | – | – | – |  |
| 2021 | 151 | 0.0 | [0–2.5] | – | – | – |  |
| 2022 | 198 | 0.0 | [0–1.9] | – | – | – |  |
| 2023 | 209 | 0.0 | [0–1.8] | – | – | – |  |
| 2024 | 46 | 0.0 | [0–7.7] | – | – | – |  |

### Table S161: Annual percentage of *Neisseria gonorrhoeae* resistance to Tetracycline from 2005 to 2024.

|  | Tested | Resistance | | Logistic model | |  | |
| --- | --- | --- | --- | --- | --- | --- | --- |
| Year | N | % | [95% CI] | OR | [95% CI] | P-value |  |
| 2005 | 61 | 1.6 | [0.3–8.7] | – | – | – |  |
| 2006 | 88 | 3.4 | [1.2–9.5] | 2.18 | [0.26–18.44] | 0.722 |  |
| 2007 | 109 | 0.9 | [0.2–5] | 0.25 | [0.03–2.14] | 0.564 |  |
| 2008 | 144 | 2.1 | [0.7–5.9] | 2.31 | [0.27–19.34] | 0.722 |  |
| 2009 | 124 | 0.0 | [0–3] | – | – | – |  |
| 2010 | 191 | 0.5 | [0.1–2.9] | – | – | – |  |
| 2011 | 152 | 0.7 | [0.1–3.6] | 1.25 | [0.09–16.8] | 0.980 |  |
| 2012 | 142 | 0.0 | [0–2.6] | – | – | – |  |
| 2013 | 135 | 0.7 | [0.1–4.1] | – | – | – |  |
| 2014 | 132 | 4.5 | [2.1–9.6] | 6.22 | [0.85–45.49] | 0.278 |  |
| 2015 | 127 | 7.1 | [3.8–12.9] | 1.43 | [0.52–3.95] | 0.722 |  |
| 2016 | 90 | 13.3 | [7.8–21.9] | 2.31 | [0.96–5.55] | 0.278 |  |
| 2017 | 160 | 21.9 | [16.2–28.9] | 1.8 | [0.92–3.53] | 0.278 |  |
| 2018 | 130 | 25.4 | [18.7–33.5] | 1.21 | [0.72–2.02] | 0.722 |  |
| 2019 | 161 | 30.4 | [23.9–37.9] | 1.26 | [0.77–2.05] | 0.722 |  |
| 2020 | 156 | 25.6 | [19.4–33] | 0.83 | [0.52–1.32] | 0.722 |  |
| 2021 | 149 | 28.9 | [22.2–36.6] | 1.15 | [0.71–1.88] | 0.776 |  |
| 2022 | 187 | 42.8 | [35.9–49.9] | 1.94 | [1.23–3.06] | 0.044 | * |
| 2023 | 215 | 64.7 | [58.1–70.7] | 2.18 | [1.47–3.24] | 0.002 | ** |
| 2024 | 148 | 71.6 | [63.9–78.3] | 1.45 | [0.95–2.23] | 0.278 |  |

## Staphylococci

### Figure S19: Annual percentage of *Staphylococcus aureus* resistance from 2005 to 2024.


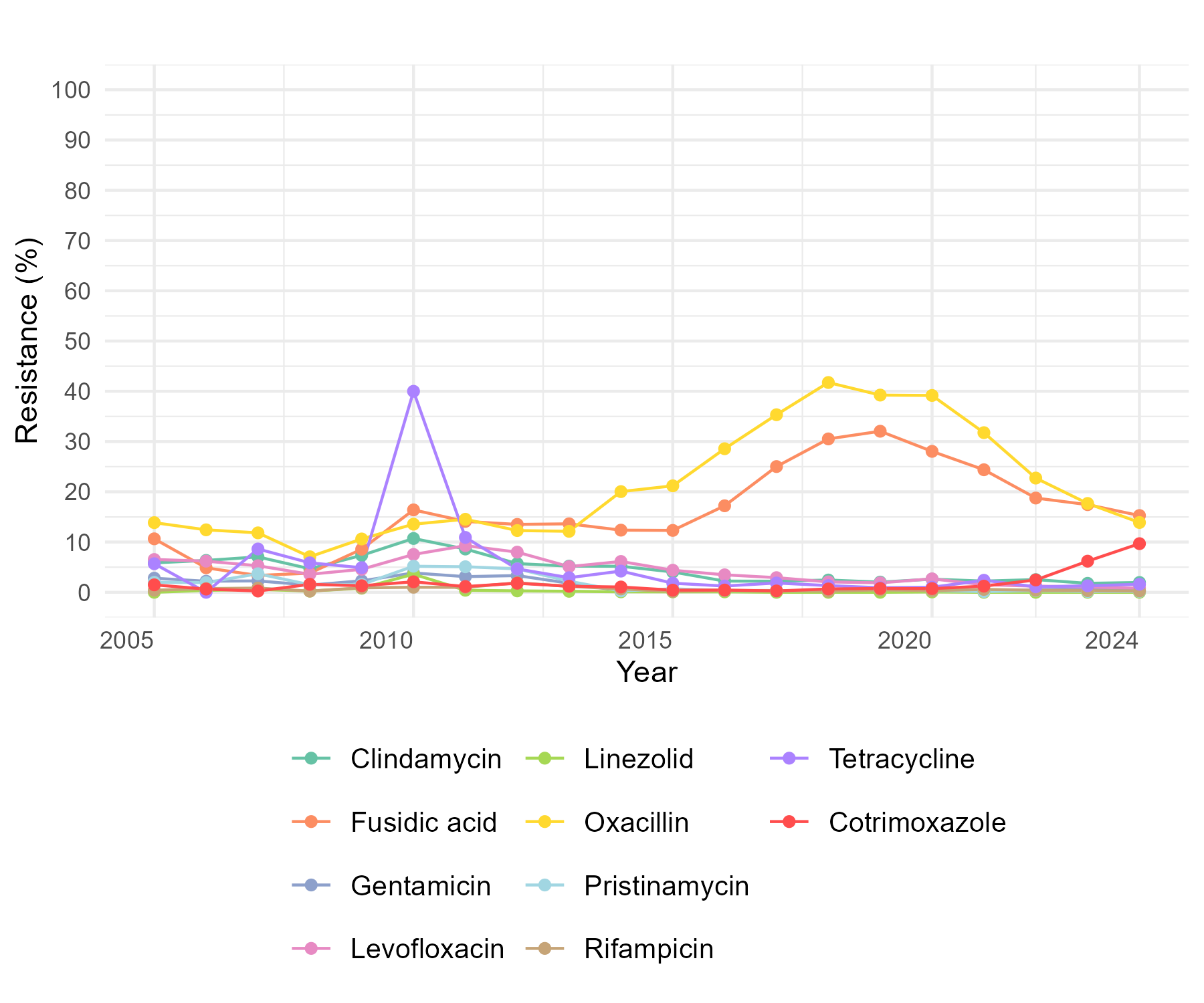


### Table S162: Logistic regression analysis of the effect of continuous time on the antibiotic resistance of *Staphylococcus aureus* from 2005 to 2024.

| Antibiotic | β | OR | [95%CI] | P-value |  |
| --- | --- | --- | --- | --- | --- |
| Clindamycin | -0.086 | 0.92 | [0.91–0.93] | 0.000 | *** |
| Fusidic acid | 0.078 | 1.08 | [1.07–1.09] | 0.000 | *** |
| Gentamicin | -0.126 | 0.88 | [0.86–0.9] | 0.000 | *** |
| Levofloxacin | -0.097 | 0.91 | [0.9–0.92] | 0.000 | *** |
| Linezolid | -0.194 | 0.82 | [0.79–0.86] | 0.000 | *** |
| Oxacillin | 0.071 | 1.07 | [1.07–1.08] | 0.000 | *** |
| Pristinamycin | -0.138 | 0.87 | [0.85–0.89] | 0.000 | *** |
| Rifampin | -0.044 | 0.96 | [0.93–0.98] | 0.003 | ** |
| Tetracycline | -0.156 | 0.86 | [0.85–0.87] | 0.000 | *** |
| Cotrimoxazole | 0.111 | 1.12 | [1.1–1.14] | 0.000 | *** |

### Table S163: Annual percentage of *Staphylococcus aureus* resistance to Clindamycin from 2005 to 2024.

|  | Tested | Resistance | | Logistic model | |  | |
| --- | --- | --- | --- | --- | --- | --- | --- |
| Year | N | % | [95% CI] | OR | [95% CI] | P-value |  |
| 2005 | 1212 | 5.9 | [4.7–7.3] | – | – | – |  |
| 2006 | 1088 | 6.3 | [5–7.9] | 1.08 | [0.76–1.53] | 0.793 |  |
| 2007 | 1192 | 7.0 | [5.7–8.6] | 1.13 | [0.8–1.58] | 0.773 |  |
| 2008 | 1259 | 4.7 | [3.7–6] | 0.68 | [0.48–0.97] | 0.127 |  |
| 2009 | 1225 | 7.3 | [6–8.9] | 1.59 | [1.12–2.24] | 0.094 | . |
| 2010 | 1195 | 10.7 | [9.1–12.6] | 1.41 | [1.06–1.89] | 0.094 | . |
| 2011 | 1219 | 8.6 | [7.2–10.3] | 0.75 | [0.57–0.99] | 0.139 |  |
| 2012 | 1051 | 5.7 | [4.5–7.3] | 0.66 | [0.47–0.92] | 0.094 | . |
| 2013 | 998 | 5.2 | [4–6.8] | 0.89 | [0.6–1.31] | 0.793 |  |
| 2014 | 1042 | 5.2 | [4–6.7] | 0.96 | [0.64–1.43] | 0.873 |  |
| 2015 | 1161 | 4.0 | [3.1–5.3] | 0.85 | [0.56–1.28] | 0.740 |  |
| 2016 | 1121 | 2.2 | [1.5–3.3] | 0.55 | [0.33–0.9] | 0.094 | . |
| 2017 | 1132 | 2.2 | [1.5–3.2] | 0.96 | [0.54–1.69] | 0.878 |  |
| 2018 | 1350 | 2.4 | [1.7–3.4] | 1.15 | [0.68–1.96] | 0.793 |  |
| 2019 | 1428 | 2.0 | [1.4–2.9] | 0.81 | [0.49–1.35] | 0.740 |  |
| 2020 | 1491 | 2.6 | [1.9–3.6] | 1.32 | [0.8–2.15] | 0.652 |  |
| 2021 | 1389 | 2.2 | [1.6–3.2] | 0.81 | [0.5–1.32] | 0.740 |  |
| 2022 | 1464 | 2.5 | [1.8–3.5] | 1.13 | [0.69–1.84] | 0.793 |  |
| 2023 | 1232 | 1.8 | [1.2–2.7] | 0.71 | [0.41–1.21] | 0.560 |  |
| 2024 | 1126 | 2.0 | [1.3–2.9] | 1.07 | [0.59–1.96] | 0.873 |  |

### Table S164: Annual percentage of *Staphylococcus aureus* resistance to Fusidic acid from 2005 to 2024.

|  | Tested | Resistance | | Logistic model | |  | |
| --- | --- | --- | --- | --- | --- | --- | --- |
| Year | N | % | [95% CI] | OR | [95% CI] | P-value |  |
| 2005 | 1212 | 10.6 | [9–12.5] | – | – | – |  |
| 2006 | 1088 | 4.9 | [3.7–6.3] | 0.43 | [0.31–0.6] | 0.000 | *** |
| 2007 | 1192 | 3.4 | [2.5–4.5] | 0.68 | [0.45–1.03] | 0.130 |  |
| 2008 | 1259 | 3.8 | [2.9–5] | 1.13 | [0.74–1.73] | 0.681 |  |
| 2009 | 1225 | 8.6 | [7.1–10.3] | 2.36 | [1.66–3.35] | 0.000 | *** |
| 2010 | 1195 | 16.4 | [14.4–18.6] | 2.12 | [1.65–2.73] | 0.000 | *** |
| 2011 | 1219 | 14.1 | [12.3–16.2] | 0.85 | [0.68–1.06] | 0.246 |  |
| 2012 | 1051 | 13.5 | [11.6–15.7] | 0.94 | [0.74–1.2] | 0.712 |  |
| 2013 | 998 | 13.6 | [11.6–15.9] | 1.02 | [0.79–1.31] | 0.910 |  |
| 2014 | 1043 | 12.4 | [10.5–14.5] | 0.9 | [0.69–1.16] | 0.531 |  |
| 2015 | 1161 | 12.3 | [10.5–14.3] | 0.99 | [0.76–1.27] | 0.910 |  |
| 2016 | 1121 | 17.2 | [15.1–19.5] | 1.47 | [1.16–1.86] | 0.004 | ** |
| 2017 | 1131 | 25.0 | [22.6–27.6] | 1.61 | [1.31–1.98] | 0.000 | *** |
| 2018 | 1353 | 30.5 | [28.1–33] | 1.31 | [1.1–1.56] | 0.008 | ** |
| 2019 | 1429 | 32.1 | [29.7–34.5] | 1.08 | [0.92–1.26] | 0.503 |  |
| 2020 | 1494 | 28.0 | [25.8–30.4] | 0.82 | [0.7–0.96] | 0.037 | * |
| 2021 | 1390 | 24.4 | [22.2–26.7] | 0.83 | [0.71–0.98] | 0.068 | . |
| 2022 | 1466 | 18.8 | [16.8–20.8] | 0.71 | [0.6–0.85] | 0.001 | *** |
| 2023 | 1244 | 17.4 | [15.4–19.7] | 0.91 | [0.75–1.11] | 0.503 |  |
| 2024 | 1126 | 15.3 | [13.3–17.5] | 0.85 | [0.69–1.06] | 0.246 |  |

### Table S165: Annual percentage of *Staphylococcus aureus* resistance to Gentamicin from 2005 to 2024.

|  | Tested | Resistance | | Logistic model | |  | |
| --- | --- | --- | --- | --- | --- | --- | --- |
| Year | N | % | [95% CI] | OR | [95% CI] | P-value |  |
| 2005 | 1212 | 2.8 | [2–3.9] | – | – | – |  |
| 2006 | 1088 | 2.2 | [1.5–3.3] | 0.79 | [0.46–1.37] | 0.704 |  |
| 2007 | 1192 | 2.3 | [1.6–3.3] | 1.03 | [0.58–1.82] | 0.930 |  |
| 2008 | 1258 | 1.4 | [0.9–2.3] | 0.67 | [0.36–1.24] | 0.542 |  |
| 2009 | 1225 | 2.3 | [1.6–3.3] | 1.57 | [0.85–2.89] | 0.474 |  |
| 2010 | 1195 | 3.8 | [2.9–5.1] | 1.58 | [0.96–2.57] | 0.328 |  |
| 2011 | 1218 | 3.1 | [2.3–4.3] | 0.78 | [0.49–1.22] | 0.573 |  |
| 2012 | 1051 | 3.3 | [2.4–4.6] | 1.11 | [0.69–1.8] | 0.847 |  |
| 2013 | 997 | 1.8 | [1.1–2.8] | 0.52 | [0.29–0.94] | 0.283 |  |
| 2014 | 1043 | 0.3 | [0.1–0.8] | 0.15 | [0.04–0.52] | 0.055 | . |
| 2015 | 1161 | 0.3 | [0.1–0.9] | 1.33 | [0.29–6.13] | 0.851 |  |
| 2016 | 1121 | 0.4 | [0.1–0.9] | 1.07 | [0.26–4.39] | 0.930 |  |
| 2017 | 1132 | 0.3 | [0.1–0.8] | 0.72 | [0.16–3.31] | 0.847 |  |
| 2018 | 1353 | 0.4 | [0.2–0.9] | 1.45 | [0.33–6.24] | 0.847 |  |
| 2019 | 1428 | 0.6 | [0.3–1.1] | 1.5 | [0.48–4.71] | 0.770 |  |
| 2020 | 1494 | 0.6 | [0.3–1.1] | 1.08 | [0.41–2.87] | 0.930 |  |
| 2021 | 1392 | 0.1 | [0–0.5] | 0.23 | [0.05–1.09] | 0.328 |  |
| 2022 | 1466 | 0.5 | [0.2–1] | 3.31 | [0.67–16.48] | 0.474 |  |
| 2023 | 1243 | 0.7 | [0.4–1.4] | 1.54 | [0.56–4.23] | 0.704 |  |
| 2024 | 1126 | 0.4 | [0.1–0.9] | 0.48 | [0.14–1.59] | 0.542 |  |

### Table S166: Annual percentage of *Staphylococcus aureus* resistance to Levofloxacin from 2005 to 2024.

|  | Tested | Resistance | | Logistic model | |  | |
| --- | --- | --- | --- | --- | --- | --- | --- |
| Year | N | % | [95% CI] | OR | [95% CI] | P-value |  |
| 2005 | 1026 | 6.5 | [5.2–8.2] | – | – | – |  |
| 2006 | 1081 | 6.2 | [4.9–7.8] | 0.93 | [0.64–1.34] | 0.686 |  |
| 2007 | 1192 | 5.3 | [4.2–6.7] | 0.83 | [0.57–1.21] | 0.545 |  |
| 2008 | 1256 | 3.6 | [2.7–4.8] | 0.74 | [0.49–1.11] | 0.543 |  |
| 2009 | 1224 | 4.6 | [3.5–5.9] | 1.23 | [0.81–1.86] | 0.545 |  |
| 2010 | 1195 | 7.5 | [6.2–9.2] | 1.53 | [1.07–2.2] | 0.126 |  |
| 2011 | 1217 | 9.3 | [7.8–11] | 1.24 | [0.91–1.68] | 0.545 |  |
| 2012 | 1051 | 8.0 | [6.5–9.8] | 0.87 | [0.64–1.2] | 0.587 |  |
| 2013 | 998 | 5.1 | [3.9–6.7] | 0.58 | [0.39–0.84] | 0.081 | . |
| 2014 | 1043 | 6.1 | [4.8–7.8] | 1.15 | [0.77–1.7] | 0.594 |  |
| 2015 | 1161 | 4.4 | [3.4–5.7] | 0.8 | [0.54–1.18] | 0.545 |  |
| 2016 | 1121 | 3.5 | [2.6–4.7] | 0.81 | [0.52–1.26] | 0.545 |  |
| 2017 | 1132 | 2.9 | [2.1–4.1] | 0.78 | [0.48–1.26] | 0.545 |  |
| 2018 | 1353 | 2.1 | [1.4–3] | 0.74 | [0.44–1.25] | 0.545 |  |
| 2019 | 1431 | 1.9 | [1.3–2.7] | 0.88 | [0.51–1.52] | 0.686 |  |
| 2020 | 1494 | 2.7 | [2–3.6] | 1.48 | [0.9–2.45] | 0.543 |  |
| 2021 | 1390 | 1.5 | [1–2.3] | 0.52 | [0.3–0.89] | 0.126 |  |
| 2022 | 1465 | 1.2 | [0.8–1.9] | 0.8 | [0.42–1.52] | 0.594 |  |
| 2023 | 1236 | 1.1 | [0.6–1.8] | 0.86 | [0.42–1.77] | 0.686 |  |
| 2024 | 1125 | 0.8 | [0.4–1.5] | 0.73 | [0.31–1.72] | 0.594 |  |

### Table S167: Annual percentage of *Staphylococcus aureus* resistance to Linezolid from 2005 to 2024.

|  | Tested | Resistance | | Logistic model | |  | |
| --- | --- | --- | --- | --- | --- | --- | --- |
| Year | N | % | [95% CI] | OR | [95% CI] | P-value |  |
| 2005 | 335 | 0.0 | [0–1.1] | – | – | – |  |
| 2006 | 1071 | 0.4 | [0.1–1] | – | – | – |  |
| 2007 | 1188 | 0.6 | [0.3–1.2] | 1.56 | [0.59–4.11] | 1.000 |  |
| 2008 | 1255 | 0.2 | [0.1–0.7] | 0.4 | [0.14–1.16] | 0.431 |  |
| 2009 | 1223 | 0.8 | [0.4–1.5] | 3.35 | [1.21–9.25] | 0.124 |  |
| 2010 | 1192 | 3.6 | [2.7–4.8] | 4.75 | [2.75–8.19] | 0.000 | *** |
| 2011 | 1218 | 0.4 | [0.2–1] | 0.11 | [0.06–0.24] | 0.000 | *** |
| 2012 | 1051 | 0.3 | [0.1–0.8] | 0.7 | [0.23–2.16] | 1.000 |  |
| 2013 | 997 | 0.2 | [0.1–0.7] | 0.71 | [0.17–2.89] | 1.000 |  |
| 2014 | 1042 | 0.1 | [0–0.5] | 0.49 | [0.07–3.21] | 1.000 |  |
| 2015 | 1161 | 0.1 | [0–0.5] | 0.92 | [0.1–8.08] | 1.000 |  |
| 2016 | 1121 | 0.1 | [0–0.5] | 1.03 | [0.12–9.05] | 1.000 |  |
| 2017 | 1132 | 0.0 | [0–0.3] | – | – | – |  |
| 2018 | 1351 | 0.0 | [0–0.3] | – | – | – |  |
| 2019 | 1431 | 0.0 | [0–0.3] | – | – | – |  |
| 2020 | 1493 | 0.1 | [0–0.4] | – | – | – |  |
| 2021 | 1382 | 0.0 | [0–0.3] | – | – | – |  |
| 2022 | 1465 | 0.0 | [0–0.3] | – | – | – |  |
| 2023 | 1243 | 0.0 | [0–0.3] | – | – | – |  |
| 2024 | 1125 | 0.0 | [0–0.3] | – | – | – |  |

### Table S168: Annual percentage of *Staphylococcus aureus* resistance to Oxacillin from 2005 to 2024.

|  | Tested | Resistance | | Logistic model | |  | |
| --- | --- | --- | --- | --- | --- | --- | --- |
| Year | N | % | [95% CI] | OR | [95% CI] | P-value |  |
| 2005 | 1212 | 13.9 | [12–15.9] | – | – | – |  |
| 2006 | 1086 | 12.4 | [10.6–14.5] | 0.88 | [0.69–1.13] | 0.431 |  |
| 2007 | 1192 | 11.8 | [10.1–13.8] | 0.94 | [0.73–1.21] | 0.734 |  |
| 2008 | 1259 | 7.1 | [5.8–8.6] | 0.58 | [0.44–0.76] | 0.000 | *** |
| 2009 | 1225 | 10.6 | [9–12.5] | 1.55 | [1.17–2.05] | 0.004 | ** |
| 2010 | 1195 | 13.6 | [11.7–15.6] | 1.29 | [1.01–1.65] | 0.067 | . |
| 2011 | 1218 | 14.5 | [12.7–16.6] | 1.07 | [0.85–1.35] | 0.656 |  |
| 2012 | 1050 | 12.3 | [10.4–14.4] | 0.83 | [0.65–1.06] | 0.215 |  |
| 2013 | 997 | 12.1 | [10.3–14.3] | 0.98 | [0.75–1.28] | 0.940 |  |
| 2014 | 1043 | 20.0 | [17.7–22.6] | 1.8 | [1.42–2.3] | 0.000 | *** |
| 2015 | 1161 | 21.2 | [18.9–23.6] | 1.09 | [0.89–1.34] | 0.495 |  |
| 2016 | 1120 | 28.6 | [26–31.3] | 1.5 | [1.24–1.82] | 0.000 | *** |
| 2017 | 1132 | 35.3 | [32.6–38.2] | 1.36 | [1.14–1.62] | 0.002 | ** |
| 2018 | 1351 | 41.7 | [39.1–44.4] | 1.33 | [1.13–1.56] | 0.002 | ** |
| 2019 | 1427 | 39.2 | [36.7–41.8] | 0.89 | [0.77–1.04] | 0.215 |  |
| 2020 | 1491 | 39.2 | [36.7–41.7] | 1 | [0.86–1.16] | 0.999 |  |
| 2021 | 1382 | 31.8 | [29.4–34.3] | 0.71 | [0.61–0.83] | 0.000 | *** |
| 2022 | 1465 | 22.7 | [20.7–24.9] | 0.63 | [0.53–0.74] | 0.000 | *** |
| 2023 | 1233 | 17.7 | [15.7–19.9] | 0.73 | [0.61–0.89] | 0.003 | ** |
| 2024 | 1116 | 13.9 | [12–16] | 0.75 | [0.6–0.94] | 0.021 | * |

### Table S169: Annual percentage of *Staphylococcus aureus* resistance to Pristinamycin from 2005 to 2024.

|  | Tested | Resistance | | Logistic model | |  | |
| --- | --- | --- | --- | --- | --- | --- | --- |
| Year | N | % | [95% CI] | OR | [95% CI] | P-value |  |
| 2005 | 1212 | 2.0 | [1.3–2.9] | – | – | – |  |
| 2006 | 1088 | 1.9 | [1.3–2.9] | 0.96 | [0.56–1.66] | 0.987 |  |
| 2007 | 1192 | 3.7 | [2.8–4.9] | 1.97 | [1.21–3.2] | 0.024 | * |
| 2008 | 1259 | 1.5 | [1–2.3] | 0.42 | [0.25–0.69] | 0.004 | ** |
| 2009 | 1225 | 1.6 | [1–2.4] | 1 | [0.56–1.81] | 0.994 |  |
| 2010 | 1195 | 5.2 | [4.1–6.6] | 3.27 | [2.03–5.29] | 0.000 | *** |
| 2011 | 1219 | 5.1 | [4–6.5] | 0.96 | [0.68–1.34] | 0.938 |  |
| 2012 | 1051 | 4.7 | [3.5–6.1] | 0.95 | [0.66–1.35] | 0.938 |  |
| 2013 | 998 | 2.2 | [1.5–3.3] | 0.45 | [0.28–0.72] | 0.004 | ** |
| 2014 | 1043 | 0.3 | [0.1–0.8] | 0.12 | [0.04–0.37] | 0.002 | ** |
| 2015 | 1161 | 0.7 | [0.3–1.4] | 2.65 | [0.78–8.98] | 0.373 |  |
| 2016 | 1121 | 0.4 | [0.1–0.9] | 0.53 | [0.17–1.59] | 0.564 |  |
| 2017 | 1131 | 0.3 | [0.1–0.8] | 0.72 | [0.18–2.86] | 0.871 |  |
| 2018 | 1350 | 0.4 | [0.2–0.9] | 1.44 | [0.39–5.36] | 0.861 |  |
| 2019 | 1428 | 0.6 | [0.3–1.1] | 1.5 | [0.54–4.19] | 0.697 |  |
| 2020 | 1490 | 0.3 | [0.1–0.8] | 0.6 | [0.21–1.67] | 0.586 |  |
| 2021 | 1382 | 0.1 | [0–0.5] | 0.41 | [0.09–1.87] | 0.564 |  |
| 2022 | 1461 | 0.3 | [0.1–0.8] | 2.35 | [0.52–10.58] | 0.564 |  |
| 2023 | 1231 | 0.2 | [0–0.6] | 0.48 | [0.11–2.16] | 0.586 |  |
| 2024 | 1119 | 0.2 | [0–0.6] | 1.08 | [0.18–6.52] | 0.987 |  |

### Table S170: Annual percentage of *Staphylococcus aureus* resistance to Rifampicin from 2005 to 2024.

|  | Tested | Resistance | | Logistic model | |  | |
| --- | --- | --- | --- | --- | --- | --- | --- |
| Year | N | % | [95% CI] | OR | [95% CI] | P-value |  |
| 2005 | 1211 | 0.4 | [0.2–1] | – | – | – |  |
| 2006 | 1086 | 0.7 | [0.4–1.4] | 1.77 | [0.58–5.36] | 0.728 |  |
| 2007 | 1192 | 0.8 | [0.5–1.5] | 1.14 | [0.45–2.87] | 0.890 |  |
| 2008 | 1259 | 0.2 | [0.1–0.7] | 0.28 | [0.08–1.02] | 0.512 |  |
| 2009 | 1225 | 0.9 | [0.5–1.6] | 3.74 | [1.05–13.29] | 0.512 |  |
| 2010 | 1195 | 1.0 | [0.6–1.7] | 1.11 | [0.49–2.52] | 0.890 |  |
| 2011 | 1218 | 1.0 | [0.6–1.7] | 0.99 | [0.45–2.2] | 0.981 |  |
| 2012 | 530 | 1.9 | [1–3.4] | 1.98 | [0.86–4.58] | 0.524 |  |
| 2013 | 675 | 1.2 | [0.6–2.3] | 0.61 | [0.24–1.55] | 0.728 |  |
| 2014 | 1043 | 0.7 | [0.3–1.4] | 0.57 | [0.21–1.57] | 0.728 |  |
| 2015 | 1161 | 0.2 | [0–0.6] | 0.26 | [0.06–1.25] | 0.524 |  |
| 2016 | 1121 | 0.4 | [0.1–0.9] | 2.08 | [0.39–11.21] | 0.746 |  |
| 2017 | 1131 | 0.4 | [0.1–0.9] | 0.98 | [0.25–3.88] | 0.981 |  |
| 2018 | 1353 | 0.2 | [0.1–0.6] | 0.63 | [0.14–2.78] | 0.789 |  |
| 2019 | 1429 | 0.5 | [0.2–1] | 2.2 | [0.57–8.41] | 0.728 |  |
| 2020 | 1491 | 0.3 | [0.1–0.8] | 0.68 | [0.22–2.11] | 0.789 |  |
| 2021 | 1392 | 0.6 | [0.3–1.1] | 1.71 | [0.56–5.18] | 0.728 |  |
| 2022 | 1466 | 0.4 | [0.2–0.9] | 0.7 | [0.24–2] | 0.789 |  |
| 2023 | 1243 | 0.3 | [0.1–0.8] | 0.79 | [0.23–2.79] | 0.890 |  |
| 2024 | 1126 | 0.3 | [0.1–0.8] | 0.82 | [0.18–3.61] | 0.890 |  |

### Table S171: Annual percentage of *Staphylococcus aureus* resistance to Tetracycline from 2005 to 2024.

|  | Tested | Resistance | | Logistic model | |  | |
| --- | --- | --- | --- | --- | --- | --- | --- |
| Year | N | % | [95% CI] | OR | [95% CI] | P-value |  |
| 2005 | 879 | 5.7 | [4.3–7.4] | – | – | – |  |
| 2006 | 20 | 0.0 | [0–16.1] | – | – | – |  |
| 2007 | 58 | 8.6 | [3.7–18.6] | – | – | – |  |
| 2008 | 1194 | 5.9 | [4.7–7.3] | 0.67 | [0.26–1.71] | 0.539 |  |
| 2009 | 1225 | 4.9 | [3.8–6.3] | 0.83 | [0.58–1.18] | 0.453 |  |
| 2010 | 1195 | 40.0 | [37.3–42.8] | 12.9 | [9.73–17.11] | 0.000 | *** |
| 2011 | 1218 | 10.9 | [9.3–12.8] | 0.18 | [0.14–0.22] | 0.000 | *** |
| 2012 | 1052 | 4.7 | [3.5–6.1] | 0.4 | [0.29–0.57] | 0.000 | *** |
| 2013 | 998 | 2.9 | [2–4.1] | 0.61 | [0.38–0.97] | 0.095 | . |
| 2014 | 1042 | 4.2 | [3.2–5.6] | 1.46 | [0.91–2.34] | 0.279 |  |
| 2015 | 1160 | 1.8 | [1.2–2.8] | 0.43 | [0.26–0.73] | 0.008 | ** |
| 2016 | 1121 | 1.2 | [0.7–2.1] | 0.69 | [0.35–1.36] | 0.453 |  |
| 2017 | 1132 | 1.9 | [1.2–2.8] | 1.48 | [0.75–2.9] | 0.453 |  |
| 2018 | 1351 | 1.3 | [0.8–2.1] | 0.72 | [0.39–1.35] | 0.453 |  |
| 2019 | 1427 | 0.9 | [0.5–1.6] | 0.68 | [0.33–1.38] | 0.453 |  |
| 2020 | 1491 | 1.0 | [0.6–1.7] | 1.11 | [0.53–2.32] | 0.881 |  |
| 2021 | 1377 | 2.4 | [1.7–3.3] | 2.38 | [1.3–4.38] | 0.020 | * |
| 2022 | 1466 | 1.1 | [0.7–1.8] | 0.45 | [0.25–0.81] | 0.026 | * |
| 2023 | 1239 | 1.3 | [0.8–2.1] | 1.19 | [0.6–2.38] | 0.735 |  |
| 2024 | 1126 | 1.6 | [1–2.5] | 1.23 | [0.63–2.41] | 0.687 |  |

### Table S172: Annual percentage of *Staphylococcus aureus* resistance to Cotrimoxazole from 2005 to 2024.

|  | Tested | Resistance | | Logistic model | |  | |
| --- | --- | --- | --- | --- | --- | --- | --- |
| Year | N | % | [95% CI] | OR | [95% CI] | P-value |  |
| 2005 | 1211 | 1.5 | [0.9–2.3] | – | – | – |  |
| 2006 | 1088 | 0.6 | [0.3–1.3] | 0.46 | [0.19–1.09] | 0.246 |  |
| 2007 | 1192 | 0.3 | [0.1–0.7] | 0.39 | [0.1–1.48] | 0.294 |  |
| 2008 | 1258 | 1.6 | [1–2.4] | 6.48 | [1.95–21.49] | 0.014 | * |
| 2009 | 1225 | 1.3 | [0.8–2.1] | 0.82 | [0.43–1.58] | 0.699 |  |
| 2010 | 1195 | 2.1 | [1.4–3.1] | 1.58 | [0.85–2.95] | 0.294 |  |
| 2011 | 1219 | 1.1 | [0.7–1.9] | 0.53 | [0.28–1.02] | 0.222 |  |
| 2012 | 1052 | 1.8 | [1.2–2.8] | 1.59 | [0.8–3.15] | 0.294 |  |
| 2013 | 996 | 1.2 | [0.7–2.1] | 0.66 | [0.32–1.35] | 0.375 |  |
| 2014 | 1043 | 1.1 | [0.6–1.9] | 0.87 | [0.38–1.95] | 0.833 |  |
| 2015 | 1161 | 0.4 | [0.2–1] | 0.41 | [0.14–1.16] | 0.251 |  |
| 2016 | 1121 | 0.4 | [0.2–1] | 1.04 | [0.31–3.55] | 0.946 |  |
| 2017 | 1132 | 0.3 | [0.1–0.8] | 0.59 | [0.14–2.43] | 0.635 |  |
| 2018 | 1353 | 0.7 | [0.4–1.3] | 2.54 | [0.7–9.24] | 0.294 |  |
| 2019 | 1430 | 0.8 | [0.4–1.4] | 1.16 | [0.48–2.76] | 0.833 |  |
| 2020 | 1494 | 0.7 | [0.4–1.3] | 0.97 | [0.42–2.21] | 0.946 |  |
| 2021 | 1387 | 1.2 | [0.8–2] | 1.66 | [0.78–3.52] | 0.294 |  |
| 2022 | 1466 | 2.5 | [1.8–3.4] | 2.05 | [1.16–3.64] | 0.066 | . |
| 2023 | 1243 | 6.2 | [5–7.7] | 2.62 | [1.76–3.9] | 0.000 | *** |
| 2024 | 1126 | 9.7 | [8.1–11.5] | 1.63 | [1.21–2.2] | 0.013 | * |

## Streptococci

### Figure S20: Annual percentage of *Streptococcus pneumoniae* resistance from 2005 to 2024.


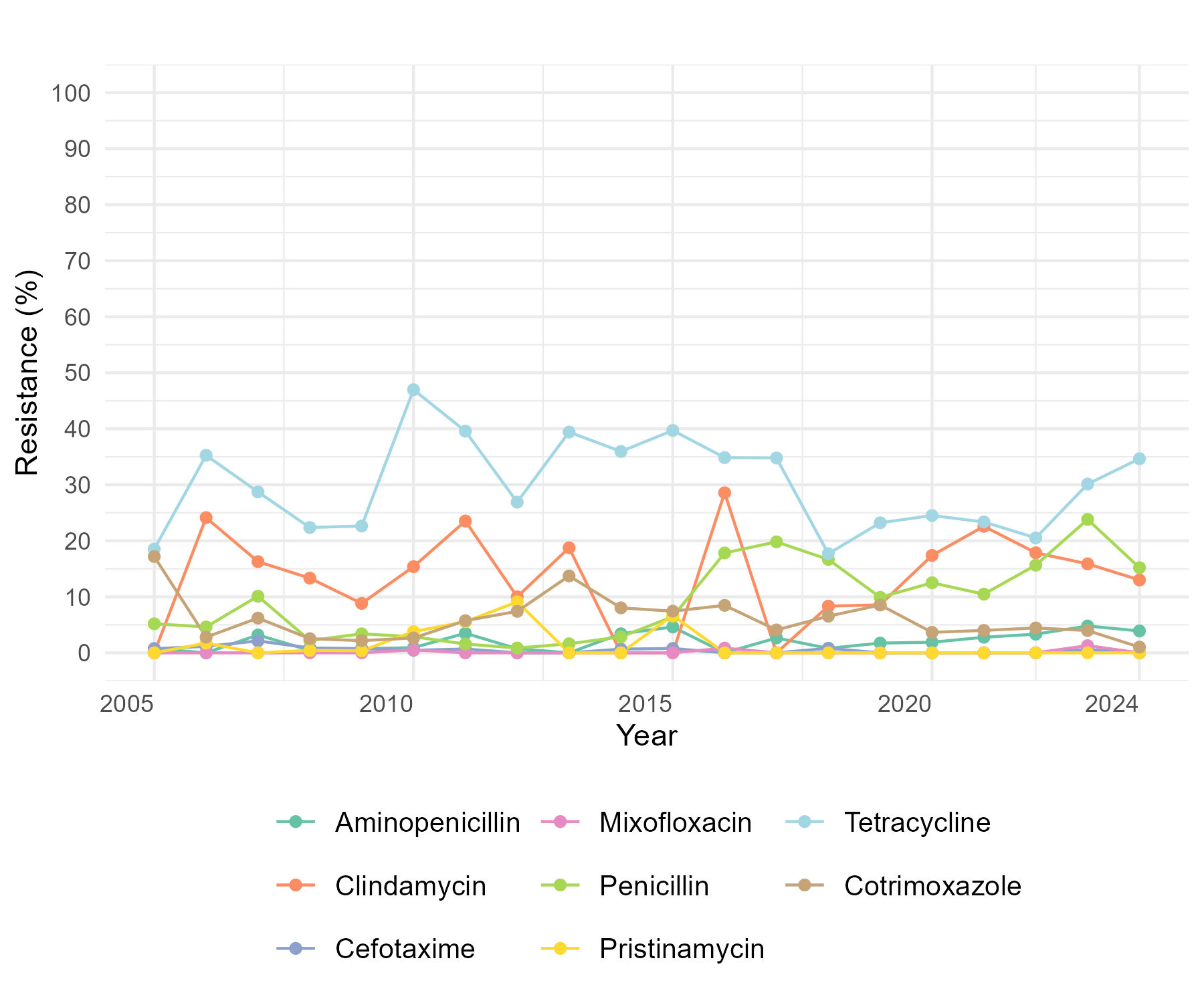


### Table S173: Logistic regression analysis of the effect of continuous time on the antibiotic resistance of *Streptococcus pneumoniae* from 2005 to 2024.

| Antibiotic | β | OR | [95%CI] | P-value |  |
| --- | --- | --- | --- | --- | --- |
| Aminopenicillin | 0.083 | 1.09 | [1.04–1.14] | 0.000 | *** |
| Cefotaxime | -0.131 | 0.88 | [0.79–0.98] | 0.020 | * |
| Clindamycin | 0.001 | 1.00 | [0.97–1.03] | 0.947 |  |
| MXF | 0.146 | 1.16 | [0.96–1.4] | 0.127 |  |
| Penicillin | 0.114 | 1.12 | [1.09–1.15] | 0.000 | *** |
| Pristinamycin | -0.030 | 0.97 | [0.86–1.1] | 0.632 |  |
| Tetracycline | 0.000 | 1.00 | [0.99–1.01] | 0.946 |  |
| Cotrimoxazole | -0.016 | 0.98 | [0.95–1.01] | 0.295 |  |

### Table S174: Annual percentage of *Streptococcus pneumoniae* resistance to Aminopenicillin from 2005 to 2024.

|  | Tested | Resistance | | Logistic model | |  | |
| --- | --- | --- | --- | --- | --- | --- | --- |
| Year | N | % | [95% CI] | OR | [95% CI] | P-value |  |
| 2005 | 135 | 0.7 | [0.1–4.1] | – | – | – |  |
| 2006 | 173 | 0.0 | [0–2.2] | – | – | – |  |
| 2007 | 188 | 3.2 | [1.5–6.8] | – | – | – |  |
| 2008 | 227 | 0.4 | [0.1–2.5] | 0.13 | [0.02–0.91] | 0.622 |  |
| 2009 | 261 | 0.8 | [0.2–2.8] | 1.77 | [0.19–16.18] | 0.990 |  |
| 2010 | 219 | 0.9 | [0.3–3.3] | 1.16 | [0.19–7.1] | 0.990 |  |
| 2011 | 143 | 3.5 | [1.5–7.9] | 4.19 | [0.91–19.22] | 0.622 |  |
| 2012 | 136 | 0.7 | [0.1–4] | 0.2 | [0.03–1.43] | 0.685 |  |
| 2013 | 157 | 0.0 | [0–2.4] | – | – | – |  |
| 2014 | 147 | 3.4 | [1.5–7.7] | – | – | – |  |
| 2015 | 130 | 4.6 | [2.1–9.7] | 1.33 | [0.44–4.06] | 0.990 |  |
| 2016 | 133 | 0.0 | [0–2.8] | – | – | – |  |
| 2017 | 113 | 2.7 | [0.9–7.5] | – | – | – |  |
| 2018 | 125 | 0.8 | [0.1–4.4] | 0.31 | [0.04–2.49] | 0.990 |  |
| 2019 | 115 | 1.7 | [0.5–6.1] | 2.2 | [0.24–20.24] | 0.990 |  |
| 2020 | 106 | 1.9 | [0.5–6.6] | 1.17 | [0.19–7.23] | 0.990 |  |
| 2021 | 108 | 2.8 | [0.9–7.9] | 1.43 | [0.27–7.59] | 0.990 |  |
| 2022 | 120 | 3.3 | [1.3–8.3] | 1.17 | [0.29–4.76] | 0.990 |  |
| 2023 | 167 | 4.8 | [2.4–9.2] | 1.5 | [0.49–4.64] | 0.990 |  |
| 2024 | 128 | 3.9 | [1.7–8.8] | 0.79 | [0.27–2.25] | 0.990 |  |

### Table S175: Annual percentage of *Streptococcus pneumoniae* resistance to Cefoxtaxime from 2005 to 2024.

|  | Tested | Resistance | | Logistic model | |  | |
| --- | --- | --- | --- | --- | --- | --- | --- |
| Year | N | % | [95% CI] | OR | [95% CI] | P-value |  |
| 2005 | 134 | 0.7 | [0.1–4.1] | – | – | – |  |
| 2006 | 173 | 1.2 | [0.3–4.1] | 1.56 | [0.23–10.4] | 1.000 |  |
| 2007 | 188 | 2.1 | [0.8–5.3] | 1.87 | [0.49–7.2] | 1.000 |  |
| 2008 | 228 | 0.9 | [0.2–3.1] | 0.39 | [0.1–1.51] | 1.000 |  |
| 2009 | 265 | 0.8 | [0.2–2.7] | 0.86 | [0.18–4.07] | 1.000 |  |
| 2010 | 219 | 0.5 | [0.1–2.5] | 0.59 | [0.09–3.94] | 1.000 |  |
| 2011 | 146 | 0.7 | [0.1–3.8] | 1.57 | [0.18–14.06] | 1.000 |  |
| 2012 | 140 | 0.0 | [0–2.7] | – | – | – |  |
| 2013 | 166 | 0.0 | [0–2.3] | – | – | – |  |
| 2014 | 150 | 0.7 | [0.1–3.7] | – | – | – |  |
| 2015 | 129 | 0.8 | [0.1–4.3] | 1.12 | [0.13–10.03] | 1.000 |  |
| 2016 | 134 | 0.0 | [0–2.8] | – | – | – |  |
| 2017 | 115 | 0.0 | [0–3.2] | – | – | – |  |
| 2018 | 125 | 0.8 | [0.1–4.4] | – | – | – |  |
| 2019 | 117 | 0.0 | [0–3.2] | – | – | – |  |
| 2020 | 105 | 0.0 | [0–3.5] | – | – | – |  |
| 2021 | 108 | 0.0 | [0–3.4] | – | – | – |  |
| 2022 | 120 | 0.0 | [0–3.1] | – | – | – |  |
| 2023 | 170 | 0.6 | [0.1–3.3] | – | – | – |  |
| 2024 | 124 | 0.0 | [0–3] | – | – | – |  |

### Table S176: Annual percentage of *Streptococcus pneumoniae* resistance to Clindamycin from 2005 to 2024.

|  | Tested | Resistance | | Logistic model | |  | |
| --- | --- | --- | --- | --- | --- | --- | --- |
| Year | N | % | [95% CI] | OR | [95% CI] | P-value |  |
| 2005 | 7 | 0.0 | [0–35.4] | – | – | – |  |
| 2006 | 29 | 24.1 | [12.2–42.1] | – | – | – |  |
| 2007 | 43 | 16.3 | [8.1–30] | 0.59 | [0.19–1.87] | 1.000 |  |
| 2008 | 30 | 13.3 | [5.3–29.7] | 0.8 | [0.22–2.94] | 1.000 |  |
| 2009 | 34 | 8.8 | [3–23] | 0.65 | [0.14–3.09] | 1.000 |  |
| 2010 | 26 | 15.4 | [6.2–33.5] | 1.78 | [0.37–8.51] | 1.000 |  |
| 2011 | 17 | 23.5 | [9.6–47.3] | 1.72 | [0.38–7.85] | 1.000 |  |
| 2012 | 10 | 10.0 | [1.8–40.4] | 0.36 | [0.04–3.65] | 1.000 |  |
| 2013 | 16 | 18.8 | [6.6–43] | 2.09 | [0.2–22.41] | 1.000 |  |
| 2014 | 16 | 0.0 | [0–19.4] | – | – | – |  |
| 2015 | 12 | 0.0 | [0–24.2] | – | – | – |  |
| 2016 | 14 | 28.6 | [11.7–54.6] | – | – | – |  |
| 2017 | 18 | 0.0 | [0–17.6] | – | – | – |  |
| 2018 | 24 | 8.3 | [2.3–25.8] | – | – | – |  |
| 2019 | 35 | 8.6 | [3–22.4] | 1.08 | [0.17–6.76] | 1.000 |  |
| 2020 | 23 | 17.4 | [7–37.1] | 2.25 | [0.47–10.9] | 1.000 |  |
| 2021 | 31 | 22.6 | [11.4–39.8] | 1.36 | [0.35–5.22] | 1.000 |  |
| 2022 | 28 | 17.9 | [7.9–35.6] | 0.77 | [0.22–2.71] | 1.000 |  |
| 2023 | 170 | 15.9 | [11.2–22.1] | 0.85 | [0.3–2.39] | 1.000 |  |
| 2024 | 123 | 13.0 | [8.2–20.1] | 0.81 | [0.42–1.55] | 1.000 |  |

### Table S177: Annual percentage of *Streptococcus pneumoniae* resistance to Mixofloxacin from 2005 to 2024.

|  | Tested | Resistance | | Logistic model | |  | |
| --- | --- | --- | --- | --- | --- | --- | --- |
| Year | N | % | [95% CI] | OR | [95% CI] | P-value |  |
| 2005 | 25 | 0.0 | [0–13.3] | – | – | – |  |
| 2006 | 137 | 0.0 | [0–2.7] | – | – | – |  |
| 2007 | 143 | 0.0 | [0–2.6] | – | – | – |  |
| 2008 | 198 | 0.0 | [0–1.9] | – | – | – |  |
| 2009 | 228 | 0.0 | [0–1.7] | – | – | – |  |
| 2010 | 189 | 0.5 | [0.1–2.9] | – | – | – |  |
| 2011 | 122 | 0.0 | [0–3.1] | – | – | – |  |
| 2012 | 135 | 0.0 | [0–2.8] | – | – | – |  |
| 2013 | 153 | 0.0 | [0–2.4] | – | – | – |  |
| 2014 | 137 | 0.0 | [0–2.7] | – | – | – |  |
| 2015 | 121 | 0.0 | [0–3.1] | – | – | – |  |
| 2016 | 119 | 0.8 | [0.1–4.6] | – | – | – |  |
| 2017 | 99 | 0.0 | [0–3.7] | – | – | – |  |
| 2018 | 108 | 0.0 | [0–3.4] | – | – | – |  |
| 2019 | 82 | 0.0 | [0–4.5] | – | – | – |  |
| 2020 | 82 | 0.0 | [0–4.5] | – | – | – |  |
| 2021 | 77 | 0.0 | [0–4.8] | – | – | – |  |
| 2022 | 92 | 0.0 | [0–4] | – | – | – |  |
| 2023 | 156 | 1.3 | [0.4–4.6] | – | – | – |  |
| 2024 | 126 | 0.0 | [0–3] | – | – | – |  |

### Table S178: Annual percentage of *Streptococcus pneumoniae* resistance to Penicillin from 2005 to 2024.

|  | Tested | Resistance | | Logistic model | |  | |
| --- | --- | --- | --- | --- | --- | --- | --- |
| Year | N | % | [95% CI] | OR | [95% CI] | P-value |  |
| 2005 | 135 | 5.2 | [2.5–10.3] | – | – | – |  |
| 2006 | 173 | 4.6 | [2.4–8.9] | 0.91 | [0.33–2.56] | 0.863 |  |
| 2007 | 188 | 10.1 | [6.6–15.2] | 2.37 | [1.02–5.52] | 0.284 |  |
| 2008 | 228 | 2.2 | [0.9–5] | 0.19 | [0.07–0.51] | 0.019 | * |
| 2009 | 265 | 3.4 | [1.8–6.3] | 1.65 | [0.55–4.95] | 0.703 |  |
| 2010 | 209 | 2.9 | [1.3–6.1] | 0.8 | [0.28–2.25] | 0.703 |  |
| 2011 | 127 | 1.6 | [0.4–5.6] | 0.59 | [0.12–2.89] | 0.703 |  |
| 2012 | 117 | 0.9 | [0.2–4.7] | 0.54 | [0.05–5.84] | 0.703 |  |
| 2013 | 124 | 1.6 | [0.4–5.7] | 1.85 | [0.17–20.07] | 0.703 |  |
| 2014 | 140 | 2.9 | [1.1–7.1] | 1.83 | [0.34–9.96] | 0.703 |  |
| 2015 | 124 | 6.5 | [3.3–12.2] | 2.31 | [0.69–7.78] | 0.475 |  |
| 2016 | 129 | 17.8 | [12.2–25.3] | 3.14 | [1.36–7.26] | 0.071 | . |
| 2017 | 106 | 19.8 | [13.3–28.4] | 1.17 | [0.61–2.25] | 0.703 |  |
| 2018 | 120 | 16.7 | [11.1–24.3] | 0.82 | [0.42–1.61] | 0.703 |  |
| 2019 | 111 | 9.9 | [5.6–16.9] | 0.53 | [0.24–1.15] | 0.347 |  |
| 2020 | 104 | 12.5 | [7.5–20.2] | 1.37 | [0.59–3.2] | 0.703 |  |
| 2021 | 105 | 10.5 | [6–17.8] | 0.8 | [0.34–1.86] | 0.703 |  |
| 2022 | 115 | 15.7 | [10.1–23.4] | 1.62 | [0.73–3.58] | 0.562 |  |
| 2023 | 168 | 23.8 | [18–30.8] | 1.7 | [0.92–3.12] | 0.344 |  |
| 2024 | 125 | 15.2 | [10–22.5] | 0.58 | [0.32–1.06] | 0.344 |  |

### Table S179: Annual percentage of *Streptococcus pneumoniae* resistance to Pristinamycin from 2005 to 2024.

|  | Tested | Resistance | | Logistic model | |  | |
| --- | --- | --- | --- | --- | --- | --- | --- |
| Year | N | % | [95% CI] | OR | [95% CI] | P-value |  |
| 2005 | 134 | 0.0 | [0–2.8] | – | – | – |  |
| 2006 | 173 | 1.7 | [0.6–5] | – | – | – |  |
| 2007 | 188 | 0.0 | [0–2] | – | – | – |  |
| 2008 | 228 | 0.4 | [0.1–2.4] | – | – | – |  |
| 2009 | 265 | 0.4 | [0.1–2.1] | 0.84 | [0.1–7.12] | 1.000 |  |
| 2010 | 132 | 3.8 | [1.6–8.6] | 10.93 | [2.06–57.98] | 0.095 | . |
| 2011 | 18 | 5.6 | [1–25.8] | 1.5 | [0.27–8.28] | 1.000 |  |
| 2012 | 11 | 9.1 | [1.6–37.7] | 1.59 | [0.17–14.81] | 1.000 |  |
| 2013 | 19 | 0.0 | [0–16.8] | – | – | – |  |
| 2014 | 22 | 0.0 | [0–14.9] | – | – | – |  |
| 2015 | 15 | 6.7 | [1.2–29.8] | – | – | – |  |
| 2016 | 14 | 0.0 | [0–21.5] | – | – | – |  |
| 2017 | 19 | 0.0 | [0–16.8] | – | – | – |  |
| 2018 | 23 | 0.0 | [0–14.3] | – | – | – |  |
| 2019 | 37 | 0.0 | [0–9.4] | – | – | – |  |
| 2020 | 24 | 0.0 | [0–13.8] | – | – | – |  |
| 2021 | 32 | 0.0 | [0–10.7] | – | – | – |  |
| 2022 | 26 | 0.0 | [0–12.9] | – | – | – |  |
| 2023 | 19 | 0.0 | [0–16.8] | – | – | – |  |
| 2024 | 28 | 0.0 | [0–12.1] | – | – | – |  |

### Table S180: Annual percentage of *Streptococcus pneumoniae* resistance to Tetracycline from 2005 to 2024.

|  | Tested | Resistance | | Logistic model | |  | |
| --- | --- | --- | --- | --- | --- | --- | --- |
| Year | N | % | [95% CI] | OR | [95% CI] | P-value |  |
| 2005 | 135 | 18.5 | [12.9–25.9] | – | – | – |  |
| 2006 | 173 | 35.3 | [28.5–42.6] | 2.43 | [1.42–4.17] | 0.012 | * |
| 2007 | 188 | 28.7 | [22.7–35.6] | 0.73 | [0.47–1.15] | 0.418 |  |
| 2008 | 228 | 22.4 | [17.4–28.2] | 0.7 | [0.45–1.1] | 0.340 |  |
| 2009 | 265 | 22.6 | [18–28.1] | 1.05 | [0.68–1.6] | 0.879 |  |
| 2010 | 215 | 47.0 | [40.4–53.6] | 2.95 | [1.99–4.39] | 0.000 | *** |
| 2011 | 139 | 39.6 | [31.8–47.9] | 0.77 | [0.5–1.19] | 0.512 |  |
| 2012 | 145 | 26.9 | [20.3–34.6] | 0.56 | [0.34–0.93] | 0.092 | . |
| 2013 | 170 | 39.4 | [32.4–46.9] | 1.75 | [1.08–2.83] | 0.092 | . |
| 2014 | 153 | 35.9 | [28.8–43.8] | 0.87 | [0.55–1.38] | 0.758 |  |
| 2015 | 131 | 39.7 | [31.7–48.3] | 1.16 | [0.72–1.89] | 0.758 |  |
| 2016 | 132 | 34.8 | [27.3–43.3] | 0.8 | [0.48–1.33] | 0.627 |  |
| 2017 | 115 | 34.8 | [26.7–43.9] | 1.02 | [0.6–1.73] | 0.950 |  |
| 2018 | 130 | 17.7 | [12.1–25.2] | 0.4 | [0.22–0.73] | 0.018 | * |
| 2019 | 112 | 23.2 | [16.4–31.8] | 1.4 | [0.74–2.63] | 0.574 |  |
| 2020 | 102 | 24.5 | [17.2–33.7] | 1.11 | [0.59–2.09] | 0.879 |  |
| 2021 | 107 | 23.4 | [16.4–32.2] | 0.92 | [0.49–1.75] | 0.879 |  |
| 2022 | 117 | 20.5 | [14.2–28.7] | 0.85 | [0.45–1.6] | 0.768 |  |
| 2023 | 166 | 30.1 | [23.7–37.5] | 1.69 | [0.96–2.96] | 0.212 |  |
| 2024 | 127 | 34.6 | [26.9–43.3] | 1.24 | [0.75–2.04] | 0.627 |  |

### Table S181: Annual percentage of *Streptococcus pneumoniae* resistance to Cotrimoxazole from 2005 to 2024.

|  | Tested | Resistance | | Logistic model | |  | |
| --- | --- | --- | --- | --- | --- | --- | --- |
| Year | N | % | [95% CI] | OR | [95% CI] | P-value |  |
| 2005 | 128 | 17.2 | [11.6–24.7] | – | – | – |  |
| 2006 | 143 | 2.8 | [1.1–7] | 0.15 | [0.05–0.45] | 0.014 | * |
| 2007 | 145 | 6.2 | [3.3–11.4] | 2.31 | [0.69–7.74] | 0.475 |  |
| 2008 | 198 | 2.5 | [1.1–5.8] | 0.37 | [0.12–1.13] | 0.475 |  |
| 2009 | 230 | 2.2 | [0.9–5] | 0.91 | [0.26–3.2] | 0.929 |  |
| 2010 | 189 | 2.6 | [1.1–6] | 1.16 | [0.33–4.12] | 0.929 |  |
| 2011 | 122 | 5.7 | [2.8–11.4] | 2.48 | [0.76–8.07] | 0.475 |  |
| 2012 | 135 | 7.4 | [4.1–13.1] | 1.26 | [0.46–3.46] | 0.929 |  |
| 2013 | 153 | 13.7 | [9.2–20.1] | 1.98 | [0.89–4.41] | 0.475 |  |
| 2014 | 137 | 8.0 | [4.5–13.8] | 0.57 | [0.26–1.24] | 0.475 |  |
| 2015 | 121 | 7.4 | [4–13.5] | 0.88 | [0.35–2.22] | 0.929 |  |
| 2016 | 118 | 8.5 | [4.7–14.9] | 1.16 | [0.45–2.99] | 0.929 |  |
| 2017 | 98 | 4.1 | [1.6–10] | 0.47 | [0.14–1.55] | 0.475 |  |
| 2018 | 107 | 6.5 | [3.2–12.9] | 1.67 | [0.47–5.94] | 0.817 |  |
| 2019 | 82 | 8.5 | [4.2–16.6] | 1.35 | [0.45–4.06] | 0.929 |  |
| 2020 | 82 | 3.7 | [1.3–10.2] | 0.42 | [0.1–1.7] | 0.475 |  |
| 2021 | 75 | 4.0 | [1.4–11.1] | 1.09 | [0.21–5.65] | 0.929 |  |
| 2022 | 90 | 4.4 | [1.7–10.9] | 1.07 | [0.23–5.01] | 0.929 |  |
| 2023 | 151 | 4.0 | [1.8–8.4] | 0.92 | [0.25–3.37] | 0.929 |  |
| 2024 | 98 | 1.0 | [0.2–5.6] | 0.25 | [0.03–2.12] | 0.475 |  |

### Figure S11: Annual percentage of *Streptococcus pyogenes* resistance from 2005 to 2024.


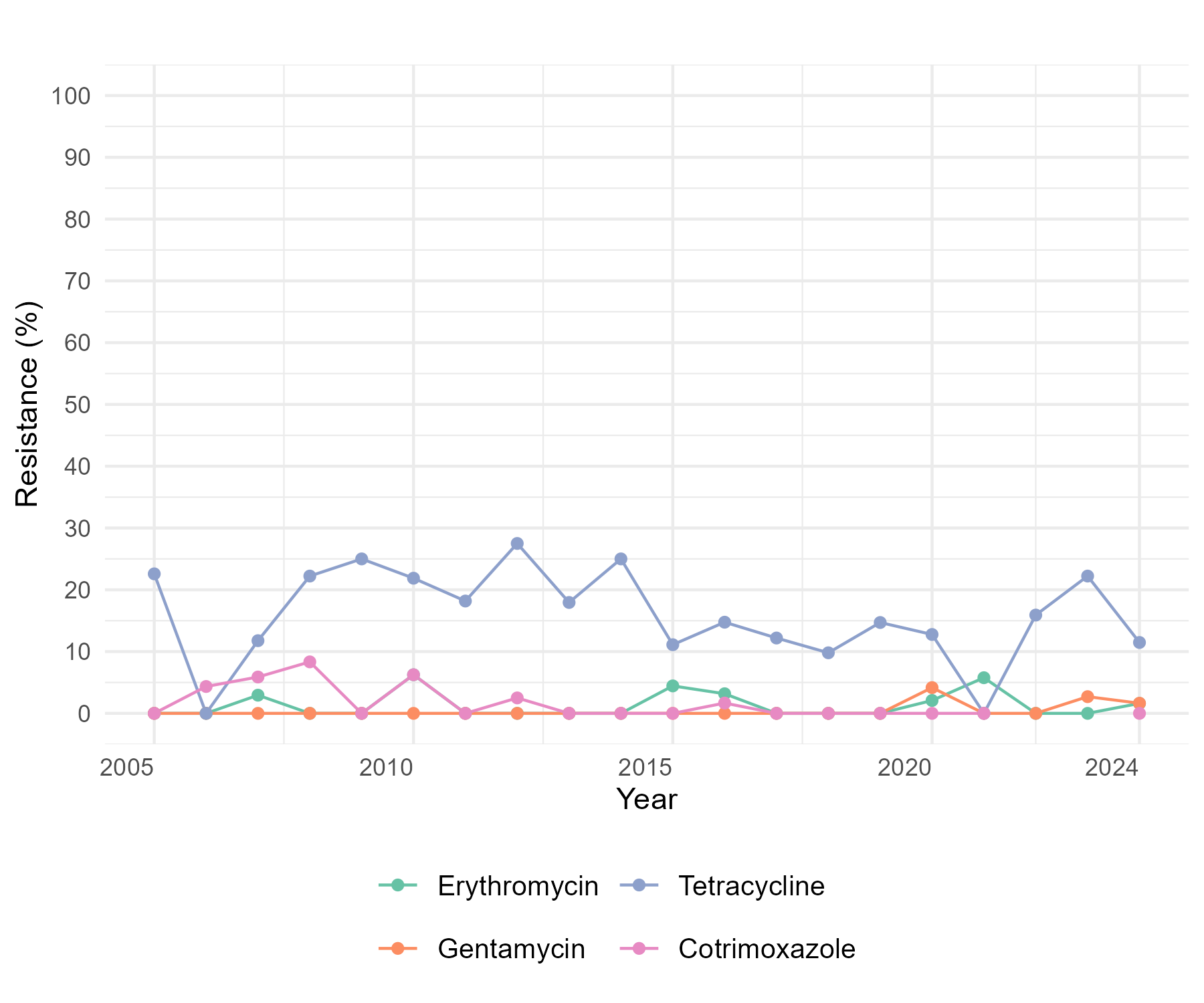


### Table S182: Logistic regression analysis of the effect of continuous time on the antibiotic resistance of *Streptococcus pyogenes* from 2005 to 2024.

| Antibiotic | β | OR | [95%CI] | P-value |  |
| --- | --- | --- | --- | --- | --- |
| Erythromycin | 0.023 | 1.02 | [0.92–1.14] | 0.672 |  |
| Levofloxacin | 0.451 | 1.57 | [1.15–2.14] | 0.005 | ** |
| Cefotaxime | 0.000 | 1.00 | [0.98–1.02] | 1.000 |  |
| Gentamicin | 0.308 | 1.36 | [1.12–1.65] | 0.002 | ** |
| Tetracycline | -0.032 | 0.97 | [0.94–1] | 0.059 | . |
| Cotrimoxazole | -0.213 | 0.81 | [0.69–0.95] | 0.010 | ** |

### Table S183: Annual percentage of *Streptococcus pyogenes* resistance to Erythromycin from 2005 to 2024.

|  | Tested | Resistance | | Logistic model | |  | |
| --- | --- | --- | --- | --- | --- | --- | --- |
| Year | N | % | [95% CI] | OR | [95% CI] | P-value |  |
| 2005 | 31 | 0.0 | [0–11] | – | – | – |  |
| 2006 | 23 | 0.0 | [0–14.3] | – | – | – |  |
| 2007 | 34 | 2.9 | [0.5–14.9] | – | – | – |  |
| 2008 | 36 | 0.0 | [0–9.6] | – | – | – |  |
| 2009 | 36 | 0.0 | [0–9.6] | – | – | – |  |
| 2010 | 32 | 6.2 | [1.7–20.1] | – | – | – |  |
| 2011 | 33 | 0.0 | [0–10.4] | – | – | – |  |
| 2012 | 39 | 0.0 | [0–9] | – | – | – |  |
| 2013 | 39 | 0.0 | [0–9] | – | – | – |  |
| 2014 | 48 | 0.0 | [0–7.4] | – | – | – |  |
| 2015 | 46 | 4.3 | [1.2–14.5] | – | – | – |  |
| 2016 | 63 | 3.2 | [0.9–10.9] | 0.73 | [0.19–2.75] | 1.000 |  |
| 2017 | 42 | 0.0 | [0–8.4] | – | – | – |  |
| 2018 | 52 | 0.0 | [0–6.9] | – | – | – |  |
| 2019 | 36 | 0.0 | [0–9.6] | – | – | – |  |
| 2020 | 48 | 2.1 | [0.4–10.9] | – | – | – |  |
| 2021 | 51 | 5.9 | [2–15.9] | 3.81 | [0.81–18] | 1.000 |  |
| 2022 | 44 | 0.0 | [0–8] | – | – | – |  |
| 2023 | 50 | 0.0 | [0–7.1] | – | – | – |  |
| 2024 | 62 | 1.6 | [0.3–8.6] | – | – | – |  |

### Table S184: Annual percentage of *Streptococcus pyogenes* resistance to Levofloxacin from 2005 to 2024.

|  | Tested | Resistance | | Logistic model | |  | |
| --- | --- | --- | --- | --- | --- | --- | --- |
| Year | N | % | [95% CI] | OR | [95% CI] | P-value |  |
| 2005 | 2 | 0.0 | [0–65.8] | – | – | – |  |
| 2006 | 23 | 0.0 | [0–14.3] | – | – | – |  |
| 2007 | 34 | 0.0 | [0–10.2] | – | – | – |  |
| 2008 | 36 | 0.0 | [0–9.6] | – | – | – |  |
| 2009 | 36 | 0.0 | [0–9.6] | – | – | – |  |
| 2010 | 32 | 0.0 | [0–10.7] | – | – | – |  |
| 2011 | 32 | 0.0 | [0–10.7] | – | – | – |  |
| 2012 | 39 | 0.0 | [0–9] | – | – | – |  |
| 2013 | 39 | 0.0 | [0–9] | – | – | – |  |
| 2014 | 48 | 0.0 | [0–7.4] | – | – | – |  |
| 2015 | 46 | 0.0 | [0–7.7] | – | – | – |  |
| 2016 | 61 | 0.0 | [0–5.9] | – | – | – |  |
| 2017 | 40 | 0.0 | [0–8.8] | – | – | – |  |
| 2018 | 52 | 1.9 | [0.3–10.1] | – | – | – |  |
| 2019 | 25 | 0.0 | [0–13.3] | – | – | – |  |
| 2020 | 1 | 0.0 | [0–79.3] | – | – | – |  |
| 2021 | 0 | 0.0 | [NaN–NaN] | – | – | – |  |
| 2022 | 0 | 0.0 | [NaN–NaN] | – | – | – |  |
| 2023 | 0 | 0.0 | [NaN–NaN] | – | – | – |  |
| 2024 | 1 | 0.0 | [0–79.3] | – | – | – |  |

### Table S185: Annual percentage of *Streptococcus pyogenes* resistance to Cefotaxime from 2005 to 2024.

|  | Tested | Resistance | | Logistic model | |  | |
| --- | --- | --- | --- | --- | --- | --- | --- |
| Year | N | % | [95% CI] | OR | [95% CI] | P-value |  |
| 2005 | 2 | 0.0 | [0–65.8] | – | – | – |  |
| 2006 | 23 | 0.0 | [0–14.3] | 1 | [0.23–4.36] | 1.000 |  |
| 2007 | 34 | 0.0 | [0–10.2] | 1 | [0.58–1.71] | 1.000 |  |
| 2008 | 36 | 0.0 | [0–9.6] | 1 | [0.62–1.61] | 1.000 |  |
| 2009 | 36 | 0.0 | [0–9.6] | 1 | [0.62–1.6] | 1.000 |  |
| 2010 | 32 | 0.0 | [0–10.7] | 1 | [0.62–1.62] | 1.000 |  |
| 2011 | 32 | 0.0 | [0–10.7] | 1 | [0.61–1.65] | 1.000 |  |
| 2012 | 39 | 0.0 | [0–9] | 1 | [0.62–1.61] | 1.000 |  |
| 2013 | 38 | 0.0 | [0–9.2] | 1 | [0.63–1.58] | 1.000 |  |
| 2014 | 48 | 0.0 | [0–7.4] | 1 | [0.65–1.54] | 1.000 |  |
| 2015 | 45 | 0.0 | [0–7.9] | 1 | [0.66–1.51] | 1.000 |  |
| 2016 | 60 | 0.0 | [0–6] | 1 | [0.67–1.48] | 1.000 |  |
| 2017 | 41 | 0.0 | [0–8.6] | 1 | [0.67–1.5] | 1.000 |  |
| 2018 | 52 | 0.0 | [0–6.9] | 1 | [0.66–1.52] | 1.000 |  |
| 2019 | 25 | 0.0 | [0–13.3] | 1 | [0.61–1.63] | 1.000 |  |
| 2020 | 1 | 0.0 | [0–79.3] | 1 | [0.13–7.65] | 1.000 |  |
| 2021 | 0 | 0.0 | [NaN–NaN] | – | – | – |  |
| 2022 | 1 | 0.0 | [0–79.3] | 1 | [0.06–16.78] | 1.000 |  |
| 2023 | 15 | 0.0 | [0–20.4] | 1 | [0.13–7.88] | 1.000 |  |
| 2024 | 61 | 0.0 | [0–5.9] | 1 | [0.56–1.78] | 1.000 |  |

### Table S186: Annual percentage of *Streptococcus pyogenes* resistance to Gentamycin from 2005 to 2024.

|  | Tested | Resistance | | Logistic model | |  | |
| --- | --- | --- | --- | --- | --- | --- | --- |
| Year | N | % | [95% CI] | OR | [95% CI] | P-value |  |
| 2005 | 31 | 0.0 | [0–11] | – | – | – |  |
| 2006 | 23 | 0.0 | [0–14.3] | – | – | – |  |
| 2007 | 34 | 0.0 | [0–10.2] | – | – | – |  |
| 2008 | 36 | 0.0 | [0–9.6] | – | – | – |  |
| 2009 | 36 | 0.0 | [0–9.6] | – | – | – |  |
| 2010 | 32 | 0.0 | [0–10.7] | – | – | – |  |
| 2011 | 33 | 0.0 | [0–10.4] | – | – | – |  |
| 2012 | 39 | 0.0 | [0–9] | – | – | – |  |
| 2013 | 39 | 0.0 | [0–9] | – | – | – |  |
| 2014 | 48 | 0.0 | [0–7.4] | – | – | – |  |
| 2015 | 46 | 0.0 | [0–7.7] | – | – | – |  |
| 2016 | 61 | 0.0 | [0–5.9] | – | – | – |  |
| 2017 | 42 | 0.0 | [0–8.4] | – | – | – |  |
| 2018 | 52 | 0.0 | [0–6.9] | – | – | – |  |
| 2019 | 36 | 0.0 | [0–9.6] | – | – | – |  |
| 2020 | 48 | 4.2 | [1.2–14] | – | – | – |  |
| 2021 | 51 | 0.0 | [0–7] | – | – | – |  |
| 2022 | 44 | 0.0 | [0–8] | – | – | – |  |
| 2023 | 37 | 2.7 | [0.5–13.8] | – | – | – |  |
| 2024 | 61 | 1.6 | [0.3–8.7] | 0.44 | [0.15–1.3] | 1.000 |  |

### Table S187: Annual percentage of *Streptococcus pyogenes* resistance to Tetracycline from 2005 to 2024.

|  | Tested | Resistance | | Logistic model | |  | |
| --- | --- | --- | --- | --- | --- | --- | --- |
| Year | N | % | [95% CI] | OR | [95% CI] | P-value |  |
| 2005 | 31 | 22.6 | [11.4–39.8] | – | – | – |  |
| 2006 | 23 | 0.0 | [0–14.3] | – | – | – |  |
| 2007 | 34 | 11.8 | [4.7–26.6] | – | – | – |  |
| 2008 | 36 | 22.2 | [11.7–38.1] | 2.08 | [0.59–7.35] | 0.960 |  |
| 2009 | 36 | 25.0 | [13.8–41.1] | 1.22 | [0.43–3.53] | 0.960 |  |
| 2010 | 32 | 21.9 | [11–38.8] | 0.8 | [0.27–2.39] | 0.960 |  |
| 2011 | 33 | 18.2 | [8.6–34.4] | 0.77 | [0.24–2.5] | 0.960 |  |
| 2012 | 39 | 28.2 | [16.5–43.8] | 2.01 | [0.67–6] | 0.960 |  |
| 2013 | 39 | 17.9 | [9–32.7] | 0.52 | [0.19–1.49] | 0.960 |  |
| 2014 | 48 | 25.0 | [14.9–38.8] | 1.54 | [0.56–4.25] | 0.960 |  |
| 2015 | 46 | 10.9 | [4.7–23] | 0.36 | [0.12–1.08] | 0.960 |  |
| 2016 | 61 | 14.8 | [8–25.7] | 1.41 | [0.46–4.34] | 0.960 |  |
| 2017 | 41 | 12.2 | [5.3–25.5] | 0.8 | [0.26–2.49] | 0.960 |  |
| 2018 | 52 | 9.6 | [4.2–20.6] | 0.75 | [0.21–2.65] | 0.960 |  |
| 2019 | 34 | 14.7 | [6.4–30.1] | 1.7 | [0.47–6.11] | 0.960 |  |
| 2020 | 47 | 12.8 | [6–25.2] | 0.89 | [0.26–3.04] | 0.984 |  |
| 2021 | 50 | 0.0 | [0–7.1] | – | – | – |  |
| 2022 | 44 | 15.9 | [7.9–29.4] | – | – | – |  |
| 2023 | 45 | 22.2 | [12.5–36.3] | 1.39 | [0.49–3.92] | 0.960 |  |
| 2024 | 61 | 11.5 | [5.7–21.8] | 0.46 | [0.16–1.26] | 0.960 |  |

### Table S188: Annual percentage of *Streptococcus pyogenes* resistance to Cotrimoxazole from 2005 to 2024.

|  | Tested | Resistance | | Logistic model | |  | |
| --- | --- | --- | --- | --- | --- | --- | --- |
| Year | N | % | [95% CI] | OR | [95% CI] | P-value |  |
| 2005 | 31 | 0.0 | [0–11] | – | – | – |  |
| 2006 | 23 | 4.3 | [0.8–21] | – | – | – |  |
| 2007 | 34 | 5.9 | [1.6–19.1] | 1.5 | [0.32–7.08] | 1.000 |  |
| 2008 | 36 | 8.3 | [2.9–21.8] | 1.36 | [0.42–4.39] | 1.000 |  |
| 2009 | 36 | 0.0 | [0–9.6] | – | – | – |  |
| 2010 | 32 | 6.2 | [1.7–20.1] | – | – | – |  |
| 2011 | 33 | 0.0 | [0–10.4] | – | – | – |  |
| 2012 | 39 | 2.6 | [0.5–13.2] | – | – | – |  |
| 2013 | 38 | 0.0 | [0–9.2] | – | – | – |  |
| 2014 | 48 | 0.0 | [0–7.4] | – | – | – |  |
| 2015 | 45 | 0.0 | [0–7.9] | – | – | – |  |
| 2016 | 60 | 1.7 | [0.3–8.9] | – | – | – |  |
| 2017 | 41 | 0.0 | [0–8.6] | – | – | – |  |
| 2018 | 52 | 0.0 | [0–6.9] | – | – | – |  |
| 2019 | 23 | 0.0 | [0–14.3] | – | – | – |  |
| 2020 | 2 | 0.0 | [0–65.8] | – | – | – |  |
| 2021 | 1 | 0.0 | [0–79.3] | – | – | – |  |
| 2022 | 0 | 0.0 | [NaN–NaN] | – | – | – |  |
| 2023 | 0 | 0.0 | [NaN–NaN] | – | – | – |  |
| 2024 | 1 | 0.0 | [0–79.3] | – | – | – |  |

### Figure S22: Annual percentage of *Streptococcus agalactiae* resistance from 2005 to 2024.


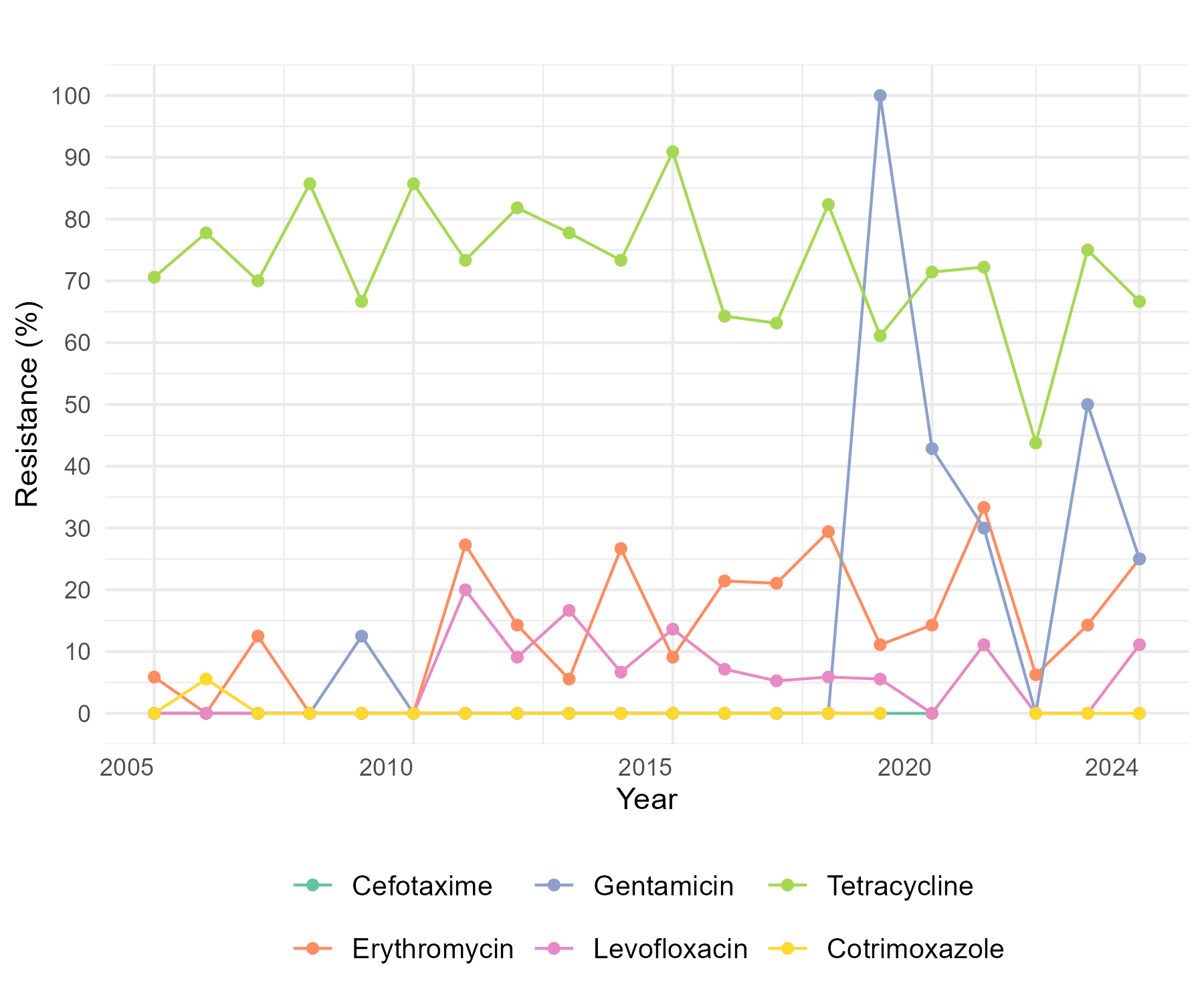


### Table S189: Logistic regression analysis of the effect of continuous time on the antibiotic resistance of *Streptococcus agalactiae* from 2005 to 2024.

| Antibiotic | β | OR | [95%CI] | P-value |  |
| --- | --- | --- | --- | --- | --- |
| Erythromycin | 0.079 | 1.08 | [1.02–1.15] | 0.015 | * |
| Cefotaxime | 0.000 | 1.00 | [0.96–1.05] | 1.000 |  |
| Gentamicin | 0.209 | 1.23 | [1.07–1.42] | 0.004 | ** |
| Levofloxacin | 0.007 | 1.01 | [0.91–1.11] | 0.890 |  |
| Tetracycline | -0.037 | 0.96 | [0.92–1.01] | 0.144 |  |
| Cotrimoxazole | -0.439 | 0.64 | [0.45–0.93] | 0.021 | * |

### Table S190: Annual percentage of *Streptococcus agalactiae* resistance to Erythromycin from 2005 to 2024.

|  | Tested | Resistance | | Logistic model | |  | |
| --- | --- | --- | --- | --- | --- | --- | --- |
| Year | N | % | [95% CI] | OR | [95% CI] | P-value |  |
| 2005 | 17 | 5.9 | [1–27] | – | – | – |  |
| 2006 | 18 | 0.0 | [0–17.6] | – | – | – |  |
| 2007 | 8 | 12.5 | [2.2–47.1] | – | – | – |  |
| 2008 | 1 | 0.0 | [0–79.3] | – | – | – |  |
| 2009 | 5 | 0.0 | [0–43.4] | – | – | – |  |
| 2010 | 8 | 0.0 | [0–32.4] | – | – | – |  |
| 2011 | 11 | 27.3 | [9.7–56.6] | – | – | – |  |
| 2012 | 21 | 14.3 | [5–34.6] | 0.46 | [0.07–2.78] | 0.886 |  |
| 2013 | 18 | 5.6 | [1–25.8] | 0.37 | [0.04–3.87] | 0.886 |  |
| 2014 | 15 | 26.7 | [10.9–52] | 6.04 | [0.6–60.4] | 0.811 |  |
| 2015 | 22 | 9.1 | [2.5–27.8] | 0.29 | [0.05–1.81] | 0.811 |  |
| 2016 | 14 | 21.4 | [7.6–47.6] | 2.66 | [0.39–18.25] | 0.886 |  |
| 2017 | 19 | 21.1 | [8.5–43.3] | 0.96 | [0.18–5.13] | 1.000 |  |
| 2018 | 17 | 29.4 | [13.3–53.1] | 1.57 | [0.35–7.12] | 0.960 |  |
| 2019 | 18 | 11.1 | [3.1–32.8] | 0.31 | [0.05–1.83] | 0.811 |  |
| 2020 | 14 | 14.3 | [4–39.9] | 1.34 | [0.17–10.75] | 1.000 |  |
| 2021 | 18 | 33.3 | [16.3–56.3] | 3.09 | [0.52–18.31] | 0.811 |  |
| 2022 | 16 | 6.2 | [1.1–28.3] | 0.13 | [0.01–1.22] | 0.811 |  |
| 2023 | 14 | 14.3 | [4–39.9] | 2.54 | [0.21–30.9] | 0.886 |  |
| 2024 | 20 | 25.0 | [11.2–46.9] | 1.95 | [0.32–11.74] | 0.886 |  |

### Table S191: Annual percentage of *Streptococcus agalactiae* resistance to Cefotaxime from 2005 to 2024.

|  | Tested | Resistance | | Logistic model | |  | |
| --- | --- | --- | --- | --- | --- | --- | --- |
| Year | N | % | [95% CI] | OR | [95% CI] | P-value |  |
| 2005 | 1 | 0 | [0–79.3] | – | – | – |  |
| 2006 | 4 | 0 | [0–49] | 1 | [0.07–14.77] | 1.000 |  |
| 2007 | 6 | 0 | [0–39] | 1 | [0.19–5.29] | 1.000 |  |
| 2008 | 1 | 0 | [0–79.3] | 1 | [0.08–12.58] | 1.000 |  |
| 2009 | 5 | 0 | [0–43.4] | 1 | [0.07–13.74] | 1.000 |  |
| 2010 | 7 | 0 | [0–35.4] | 1 | [0.25–4.07] | 1.000 |  |
| 2011 | 1 | 0 | [0–79.3] | 1 | [0.08–12.44] | 1.000 |  |
| 2012 | 3 | 0 | [0–56.1] | 1 | [0.06–15.62] | 1.000 |  |
| 2013 | 2 | 0 | [0–65.8] | 1 | [0.11–9.03] | 1.000 |  |
| 2014 | 4 | 0 | [0–49] | 1 | [0.13–7.78] | 1.000 |  |
| 2015 | 7 | 0 | [0–35.4] | 1 | [0.23–4.32] | 1.000 |  |
| 2016 | 3 | 0 | [0–56.1] | 1 | [0.19–5.39] | 1.000 |  |
| 2017 | 4 | 0 | [0–49] | 1 | [0.16–6.21] | 1.000 |  |
| 2018 | 6 | 0 | [0–39] | 1 | [0.22–4.54] | 1.000 |  |
| 2019 | 1 | 0 | [0–79.3] | 1 | [0.08–12.72] | 1.000 |  |
| 2020 | 2 | 0 | [0–65.8] | 1 | [0.06–17.5] | 1.000 |  |
| 2021 | 0 | 0 | [NaN–NaN] | – | – | – |  |
| 2022 | 0 | 0 | [NaN–NaN] | – | – | – |  |
| 2023 | 0 | 0 | [NaN–NaN] | – | – | – |  |
| 2024 | 10 | 0 | [0–27.8] | 1 | [0.17–6.05] | 1.000 |  |

### Table S192: Annual percentage of *Streptococcus agalactiae* resistance to Gentamicin from 2005 to 2024.

|  | Tested | Resistance | | Logistic model | |  | |
| --- | --- | --- | --- | --- | --- | --- | --- |
| Year | N | % | [95% CI] | OR | [95% CI] | P-value |  |
| 2005 | 13 | 0.0 | [0–22.8] | – | – | – |  |
| 2006 | 4 | 0.0 | [0–49] | – | – | – |  |
| 2007 | 6 | 0.0 | [0–39] | – | – | – |  |
| 2008 | 1 | 0.0 | [0–79.3] | – | – | – |  |
| 2009 | 8 | 12.5 | [2.2–47.1] | – | – | – |  |
| 2010 | 7 | 0.0 | [0–35.4] | – | – | – |  |
| 2011 | 1 | 0.0 | [0–79.3] | – | – | – |  |
| 2012 | 4 | 0.0 | [0–49] | – | – | – |  |
| 2013 | 2 | 0.0 | [0–65.8] | – | – | – |  |
| 2014 | 4 | 0.0 | [0–49] | – | – | – |  |
| 2015 | 7 | 0.0 | [0–35.4] | – | – | – |  |
| 2016 | 3 | 0.0 | [0–56.1] | – | – | – |  |
| 2017 | 4 | 0.0 | [0–49] | – | – | – |  |
| 2018 | 6 | 0.0 | [0–39] | – | – | – |  |
| 2019 | 1 | 100.0 | [20.7–100] | – | – | – |  |
| 2020 | 7 | 42.9 | [15.8–75] | – | – | – |  |
| 2021 | 10 | 30.0 | [10.8–60.3] | 0.44 | [0.1–1.94] | 1.000 |  |
| 2022 | 5 | 0.0 | [0–43.4] | – | – | – |  |
| 2023 | 2 | 50.0 | [9.5–90.5] | – | – | – |  |
| 2024 | 12 | 25.0 | [8.9–53.2] | 0.51 | [0.07–3.95] | 1.000 |  |

### Table S193: Annual percentage of *Streptococcus agalactiae* resistance to Levofloxacin from 2005 to 2024.

|  | Tested | Resistance | | Logistic model | |  | |
| --- | --- | --- | --- | --- | --- | --- | --- |
| Year | N | % | [95% CI] | OR | [95% CI] | P-value |  |
| 2005 | 4 | 0.0 | [0–49] | – | – | – |  |
| 2006 | 18 | 0.0 | [0–17.6] | – | – | – |  |
| 2007 | 10 | 0.0 | [0–27.8] | – | – | – |  |
| 2008 | 7 | 0.0 | [0–35.4] | – | – | – |  |
| 2009 | 12 | 0.0 | [0–24.2] | – | – | – |  |
| 2010 | 14 | 0.0 | [0–21.5] | – | – | – |  |
| 2011 | 15 | 20.0 | [7–45.2] | – | – | – |  |
| 2012 | 22 | 9.1 | [2.5–27.8] | 0.38 | [0.07–1.95] | 1.000 |  |
| 2013 | 18 | 16.7 | [5.8–39.2] | 1.78 | [0.35–9.12] | 1.000 |  |
| 2014 | 15 | 6.7 | [1.2–29.8] | 0.38 | [0.05–2.84] | 1.000 |  |
| 2015 | 22 | 13.6 | [4.7–33.3] | 1.95 | [0.26–14.41] | 1.000 |  |
| 2016 | 14 | 7.1 | [1.3–31.5] | 0.53 | [0.07–3.93] | 1.000 |  |
| 2017 | 19 | 5.3 | [0.9–24.6] | 0.76 | [0.07–8.38] | 1.000 |  |
| 2018 | 17 | 5.9 | [1–27] | 1.1 | [0.1–12.11] | 1.000 |  |
| 2019 | 18 | 5.6 | [1–25.8] | 0.91 | [0.08–9.93] | 1.000 |  |
| 2020 | 7 | 0.0 | [0–35.4] | – | – | – |  |
| 2021 | 9 | 11.1 | [2–43.5] | – | – | – |  |
| 2022 | 11 | 0.0 | [0–25.9] | – | – | – |  |
| 2023 | 10 | 0.0 | [0–27.8] | – | – | – |  |
| 2024 | 9 | 11.1 | [2–43.5] | – | – | – |  |

### Table S194: Annual percentage of *Streptococcus agalactiae* resistance to Tetracycline from 2005 to 2024.

|  | Tested | Resistance | | Logistic model | |  | |
| --- | --- | --- | --- | --- | --- | --- | --- |
| Year | N | % | [95% CI] | OR | [95% CI] | P-value |  |
| 2005 | 17 | 70.6 | [46.9–86.7] | – | – | – |  |
| 2006 | 18 | 77.8 | [54.8–91] | 1.66 | [0.3–9.17] | 0.820 |  |
| 2007 | 10 | 70.0 | [39.7–89.2] | 0.63 | [0.09–4.38] | 0.865 |  |
| 2008 | 7 | 85.7 | [48.7–97.4] | 2.31 | [0.17–32.01] | 0.820 |  |
| 2009 | 12 | 66.7 | [39.1–86.2] | 0.34 | [0.03–4.27] | 0.820 |  |
| 2010 | 14 | 85.7 | [60.1–96] | 3.27 | [0.44–24.44] | 0.780 |  |
| 2011 | 15 | 73.3 | [48–89.1] | 0.43 | [0.06–3.06] | 0.820 |  |
| 2012 | 22 | 81.8 | [61.5–92.7] | 1.65 | [0.32–8.62] | 0.820 |  |
| 2013 | 18 | 77.8 | [54.8–91] | 0.82 | [0.16–4.19] | 0.909 |  |
| 2014 | 15 | 73.3 | [48–89.1] | 0.76 | [0.14–4.03] | 0.892 |  |
| 2015 | 22 | 90.9 | [72.2–97.5] | 3.8 | [0.55–26.27] | 0.780 |  |
| 2016 | 14 | 64.3 | [38.8–83.7] | 0.17 | [0.03–1.17] | 0.780 |  |
| 2017 | 19 | 63.2 | [41–80.9] | 0.93 | [0.21–4.17] | 0.932 |  |
| 2018 | 17 | 82.4 | [59–93.8] | 2.75 | [0.54–13.95] | 0.780 |  |
| 2019 | 18 | 61.1 | [38.6–79.7] | 0.34 | [0.07–1.75] | 0.780 |  |
| 2020 | 14 | 71.4 | [45.4–88.3] | 1.6 | [0.34–7.61] | 0.820 |  |
| 2021 | 18 | 72.2 | [49.1–87.5] | 1.07 | [0.21–5.41] | 0.932 |  |
| 2022 | 16 | 43.8 | [23.1–66.8] | 0.29 | [0.07–1.3] | 0.780 |  |
| 2023 | 4 | 75.0 | [30.1–95.4] | 4.05 | [0.31–53.38] | 0.780 |  |
| 2024 | 12 | 66.7 | [39.1–86.2] | 0.63 | [0.04–9.15] | 0.892 |  |

### Table S195: Annual percentage of *Streptococcus agalactiae* resistance to Cotrimoxazole from 2005 to 2024.

|  | Tested | Resistance | | Logistic model | |  | |
| --- | --- | --- | --- | --- | --- | --- | --- |
| Year | N | % | [95% CI] | OR | [95% CI] | P-value |  |
| 2005 | 17 | 0.0 | [0–18.4] | – | – | – |  |
| 2006 | 18 | 5.6 | [1–25.8] | – | – | – |  |
| 2007 | 10 | 0.0 | [0–27.8] | – | – | – |  |
| 2008 | 7 | 0.0 | [0–35.4] | – | – | – |  |
| 2009 | 12 | 0.0 | [0–24.2] | – | – | – |  |
| 2010 | 14 | 0.0 | [0–21.5] | – | – | – |  |
| 2011 | 15 | 0.0 | [0–20.4] | – | – | – |  |
| 2012 | 22 | 0.0 | [0–14.9] | – | – | – |  |
| 2013 | 18 | 0.0 | [0–17.6] | – | – | – |  |
| 2014 | 15 | 0.0 | [0–20.4] | – | – | – |  |
| 2015 | 22 | 0.0 | [0–14.9] | – | – | – |  |
| 2016 | 14 | 0.0 | [0–21.5] | – | – | – |  |
| 2017 | 19 | 0.0 | [0–16.8] | – | – | – |  |
| 2018 | 11 | 0.0 | [0–25.9] | – | – | – |  |
| 2019 | 2 | 0.0 | [0–65.8] | – | – | – |  |
| 2020 | 0 | 0.0 | [NaN–NaN] | – | – | – |  |
| 2021 | 0 | 0.0 | [NaN–NaN] | – | – | – |  |
| 2022 | 2 | 0.0 | [0–65.8] | – | – | – |  |
| 2023 | 10 | 0.0 | [0–27.8] | – | – | – |  |
| 2024 | 10 | 0.0 | [0–27.8] | – | – | – |  |

### Figure S23: Annual percentage of resistance for six pathogen-drug combinations among community-acquired isolates versus among hospital-acquired isolates from 2005 to 2024.


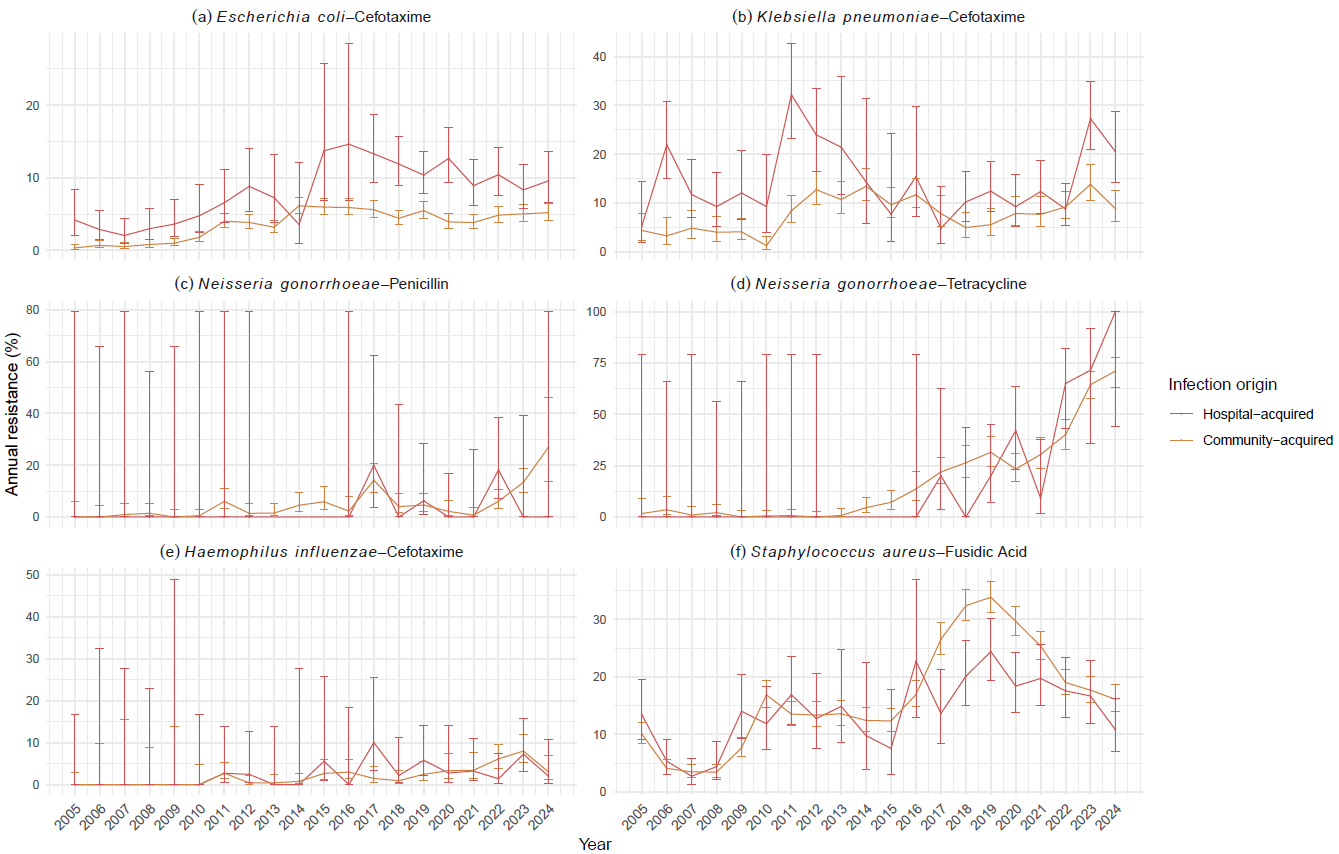


# D) High Priority Pathogens

### Table S196: Distribution of HPP types.

|  | Frequency | Percentage (%) | [95%CI] |
| --- | --- | --- | --- |
| CR-AB | 292 | 3.4 | [3.1–3.9] |
| ESBL-E | 2208 | 26.0 | [25.1–27] |
| CPE | 85 | 1.0 | [0.8–1.2] |
| CAZR-PA | 555 | 6.5 | [6–7.1] |
| MRSA | 5280 | 62.2 | [61.2–63.3] |
| VRE | 62 | 0.7 | [0.6–0.9] |

### Table S197: HPP prevalence within each bacterial species or genera.

| Group | Pathogen | Frequency | Percentage (%) | [95%CI] |
| --- | --- | --- | --- | --- |
| Enterobacterales | *Citrobacter freundii* | 53 | 7.1 | [5.5–9.2] |
| Enterobacterales | *Citrobacter koseri* | 28 | 1.3 | [0.9–1.8] |
| Enterobacterales | *Escherichia coli* | 1082 | 3 | [2.8–3.2] |
| Enterobacterales | *Enterobacter cloacae* complex | 298 | 6.7 | [6–7.5] |
| Enterobacterales | *Klebsiella pneumoniae* | 740 | 8.6 | [8.1–9.3] |
| Enterobacterales | *Morganella morganii* | 3 | 0.3 | [0.1–0.9] |
| Enterobacterales | *Proteus mirabilis* | 21 | 0.4 | [0.3–0.7] |
| Enterobacterales | *Salmonella spp.* | 8 | 0.9 | [0.5–1.8] |
| Enterobacterales | *Shigella spp.* | 0 | 0 | [0–2.4] |
| Enterobacterales | *Serratia marcescens* | 60 | 4.8 | [3.8–6.1] |
| Enterococcus | *Enterococcus faecium* | 62 | 16 | [12.7–20] |
| Non-fermenter | *Acinetobacter baumannii* | 292 | 21.8 | [19.6–24.1] |
| Non-fermenter | *Pseudomonas aeruginosa* | 555 | 9 | [8.3–9.7] |
| Staphylococcus | *Staphylococcus aureus* | 5280 | 21.6 | [21.1–22.2] |

### Table S198: ESBL prevalence by bacterial species or genera.

|  | Frequency | Percentage (%) | [95% CI] |
| --- | --- | --- | --- |
| *Citrobacter freundii* | 39 | 5.2 | [3.8–7.1] |
| *Citrobacter koseri* | 27 | 1.2 | [0.8–1.8] |
| *Escherichia coli* | 1076 | 3 | [2.8–3.2] |
| *Enterobacter cloacae* complex | 267 | 6 | [5.4–6.8] |
| *Klebsiella pneumoniae* | 721 | 8.4 | [7.9–9] |
| *Morganella morganii* | 3 | 0.3 | [0.1–0.9] |
| *Proteus mirabilis* | 20 | 0.4 | [0.3–0.6] |
| *Salmonella spp.* | 8 | 0.9 | [0.5–1.8] |
| *Serratia marcescens* | 47 | 3.8 | [2.8–5] |

### Table S199: Annual percentage of ESBL-E among Enterobacterales from 2005 to 2024.

|  | Tested | Resistance | | Logistic model | |  | |
| --- | --- | --- | --- | --- | --- | --- | --- |
| Year | N | % | [95% CI] | OR | [95% CI] | P-value |  |
| 2005 | 2451 | 0.0 | [0–0.2] | – | – | – |  |
| 2006 | 2469 | 0.0 | [0–0.2] | – | – | – |  |
| 2007 | 2829 | 1.6 | [1.2–2.1] | 38.08 | [5.79–250.38] | 0.001 | ** |
| 2008 | 2917 | 1.7 | [1.3–2.2] | 1.07 | [0.72–1.58] | 0.916 |  |
| 2009 | 3168 | 1.5 | [1.1–2] | 0.94 | [0.64–1.37] | 0.916 |  |
| 2010 | 3163 | 1.6 | [1.2–2.1] | 1.01 | [0.69–1.48] | 0.960 |  |
| 2011 | 3289 | 4.2 | [3.5–4.9] | 2.59 | [1.9–3.55] | 0.000 | *** |
| 2012 | 3105 | 4.5 | [3.8–5.3] | 1.11 | [0.88–1.4] | 0.639 |  |
| 2013 | 2939 | 4.4 | [3.7–5.2] | 0.97 | [0.76–1.22] | 0.916 |  |
| 2014 | 3174 | 5.2 | [4.5–6.1] | 1.21 | [0.96–1.51] | 0.349 |  |
| 2015 | 2948 | 4.5 | [3.9–5.4] | 0.83 | [0.67–1.04] | 0.349 |  |
| 2016 | 3072 | 5.2 | [4.4–6] | 1.13 | [0.9–1.41] | 0.581 |  |
| 2017 | 2805 | 4.8 | [4.1–5.7] | 0.91 | [0.72–1.14] | 0.639 |  |
| 2018 | 3026 | 4.3 | [3.6–5] | 0.85 | [0.67–1.08] | 0.418 |  |
| 2019 | 3066 | 5.1 | [4.3–5.9] | 1.13 | [0.9–1.42] | 0.581 |  |
| 2020 | 3036 | 4.7 | [4–5.5] | 0.93 | [0.74–1.16] | 0.759 |  |
| 2021 | 3070 | 4.0 | [3.4–4.8] | 0.84 | [0.66–1.06] | 0.370 |  |
| 2022 | 3390 | 4.1 | [3.5–4.9] | 1.02 | [0.81–1.3] | 0.951 |  |
| 2023 | 3150 | 6.2 | [5.4–7.1] | 1.49 | [1.21–1.85] | 0.001 | ** |
| 2024 | 2733 | 4.9 | [4.2–5.8] | 0.78 | [0.63–0.97] | 0.123 |  |

### Table S200: CPE prevalence by bacterial species or genera.

|  | Frequency | Percentage (%) | [95CI] |
| --- | --- | --- | --- |
| *Citrobacter freundii* | 14 | 1.9 | [1.1–3.1] |
| *Citrobacter koseri* | 1 | 0 | [0–0.3] |
| *Escherichia coli* | 6 | 0 | [0–0] |
| *Enterobacter cloacae complex* | 31 | 0.7 | [0.5–1] |
| *Klebsiella pneumoniae* | 19 | 0.2 | [0.1–0.3] |
| *Morganella morganii* | 0 | 0 | [0–0.4] |
| *Proteus mirabilis* | 1 | 0 | [0–0.1] |
| *Salmonella spp.* | 0 | 0 | [0–0.4] |
| *Serratia marcescens* | 13 | 1 | [0.6–1.8] |

### Table S201: Annual percentage of CPE among Enterobacterales from 2005 to 2024.

|  | Tested | Resistance | | Logistic model | |  | |
| --- | --- | --- | --- | --- | --- | --- | --- |
| Year | N | % | [95% CI] | OR | [95% CI] | P-value |  |
| 2005 | 2451 | 0.0 | [0–0.2] | – | – | – |  |
| 2006 | 2469 | 0.0 | [0–0.2] | – | – | – |  |
| 2007 | 2829 | 0.0 | [0–0.1] | – | – | – |  |
| 2008 | 2917 | 0.0 | [0–0.1] | – | – | – |  |
| 2009 | 3168 | 0.0 | [0–0.1] | – | – | – |  |
| 2010 | 3163 | 0.0 | [0–0.1] | – | – | – |  |
| 2011 | 3289 | 0.0 | [0–0.1] | – | – | – |  |
| 2012 | 3105 | 0.0 | [0–0.1] | – | – | – |  |
| 2013 | 2939 | 0.1 | [0–0.3] | – | – | – |  |
| 2014 | 3174 | 0.0 | [0–0.2] | 0.31 | [0.06–1.72] | 0.490 |  |
| 2015 | 2948 | 0.1 | [0–0.3] | 2.12 | [0.35–12.97] | 0.987 |  |
| 2016 | 3072 | 0.2 | [0.1–0.4] | 2.34 | [0.68–8.06] | 0.490 |  |
| 2017 | 2805 | 0.2 | [0.1–0.5] | 1.29 | [0.53–3.16] | 1.000 |  |
| 2018 | 3026 | 0.5 | [0.3–0.9] | 2.51 | [1.23–5.09] | 0.135 |  |
| 2019 | 3066 | 0.3 | [0.2–0.6] | 0.56 | [0.3–1.04] | 0.325 |  |
| 2020 | 3036 | 0.3 | [0.2–0.6] | 1.01 | [0.5–2.03] | 1.000 |  |
| 2021 | 3070 | 0.2 | [0.1–0.4] | 0.54 | [0.24–1.24] | 0.490 |  |
| 2022 | 3390 | 0.2 | [0.1–0.4] | 1.25 | [0.52–2.96] | 1.000 |  |
| 2023 | 3150 | 0.5 | [0.3–0.8] | 2.33 | [1.19–4.6] | 0.135 |  |
| 2024 | 2733 | 0.2 | [0.1–0.5] | 0.45 | [0.22–0.93] | 0.192 |  |

### Figure S24: Annual percentage of resistance for HPP isolates among community-acquired isolates versus among hospital-acquired isolates from 2005 to 2024.


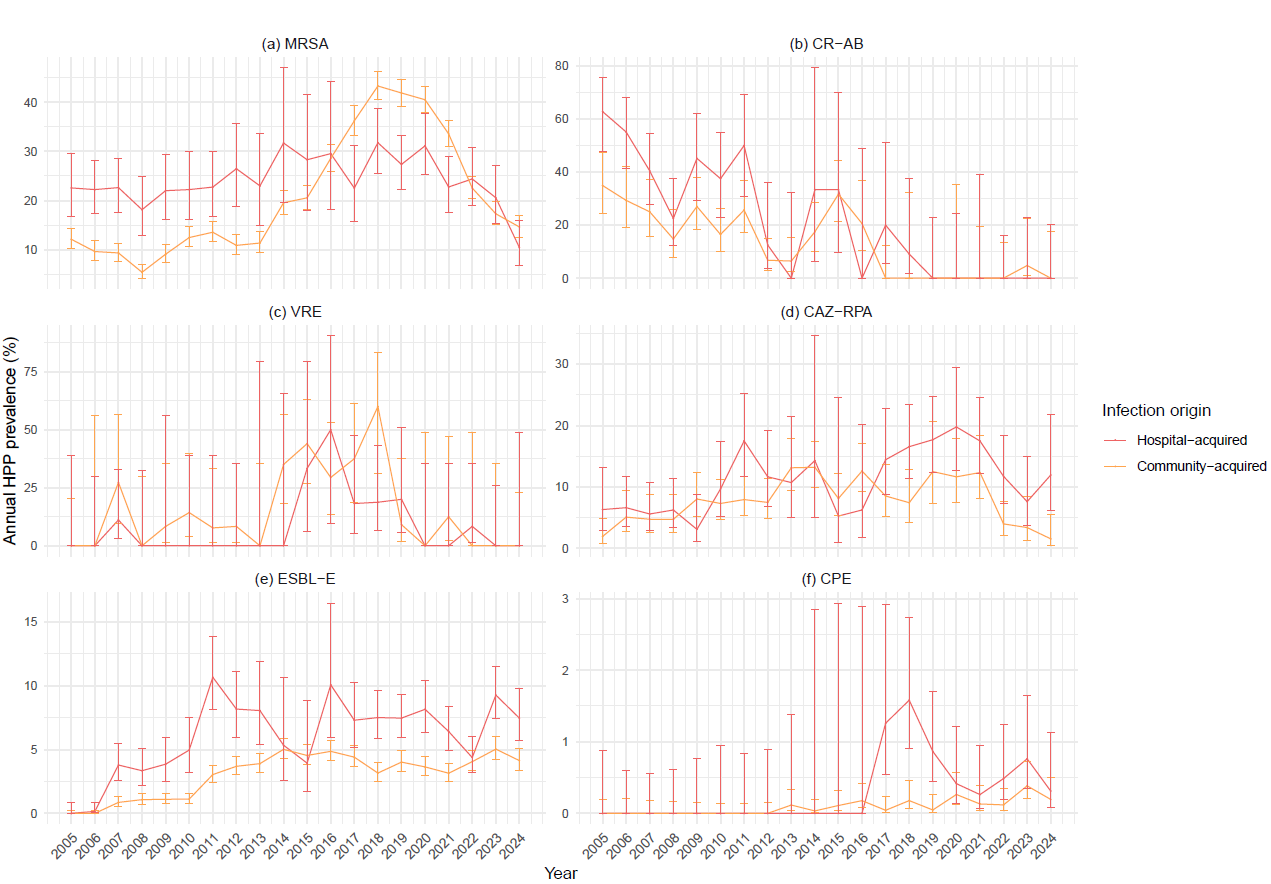


### Table S202: Logistic regression analyses for each of the six HPP types–from 2005 to 2024.

| Outcome | Variable | β | OR | [95% CI] | P-value | stars |
| --- | --- | --- | --- | --- | --- | --- |
| CR-AB | Male | 0.544 | 1.72 | [1.29–2.3] | <0.001 | *** |
| I | Age | 0.686 | 1.99 | [1.69–2.33] | <0.001 | *** |
| ACIBMI | Hemoculture | -0.975 | 0.38 | [0.24–0.58] | <0.001 | *** |
| I | Hospital-acquired | 0.585 | 1.79 | [1.35–2.39] | <0.001 | *** |
| MRSA | Male | 0.108 | 1.11 | [1.05–1.19] | <0.001 | *** |
|  | Age | 0.146 | 1.16 | [1.12–1.19] | <0.001 | *** |
| SA A | Hemoculture | -0.204 | 0.82 | [0.73–0.91] | <0.001 | *** |
| XA | Hospital-acquired | 0.072 | 1.08 | [0.98–1.18] | 0,124 |  |
| VRE | Male | 0.131 | 1.14 | [0.64–2.04] | 0,659 |  |
|  | Age | 0.392 | 1.48 | [1.07–2.05] | 0,019 | * |
|  | Hemoculture | -0.669 | 0.51 | [0.17–1.51] | 0,227 |  |
|  | Hospital-acquired | -1.075 | 0.34 | [0.17–0.67] | 0,002 | ** |
| CAZR-PA | Male | 0.253 | 1.29 | [1.07–1.55] | 0,008 | ** |
|  | Age | 0.090 | 1.09 | [1–1.2] | 0,054 | . |
|  | Hemoculture | -0.084 | 0.92 | [0.68–1.25] | 0,59 |  |
|  | Hospital-acquired | 0.359 | 1.43 | [1.2–1.71] | 0,0001 | *** |
| ESBL-E | Male | 0.506 | 1.66 | [1.52–1.81] | <0.001 | *** |
|  | Age | 0.540 | 1.72 | [1.64–1.8] | <0.001 | *** |
|  | Hemoculture | 0.424 | 1.53 | [1.35–1.73] | <0.001 | *** |
|  | Hospital-acquired | 0.381 | 1.47 | [1.33–1.61] | <0.001 | *** |
| CPE | Male | 0.914 | 2.49 | [1.62–3.84] | <0.001 | *** |
|  | Age | 0.145 | 1.16 | [0.93–1.43] | 0,181 |  |
|  | Hemoculture | 0.576 | 1.78 | [1.05–3.02] | 0,033 | * |
|  | Hospital-acquired | 1.266 | 3.55 | [2.33–5.4] | <0.001 | *** |

# E) MDR and pXDR

### Table S203: MDR prevalence within each HPP type.

|  | Overall | Frequency | Percentage (%) | [95% CI] |
| --- | --- | --- | --- | --- |
| CR-AB | 292 | 289 | 99 | [97–99.6] |
| ESBL-E | 2,206 | 1991 | 90,3 | [88.9–91.4] |
| CPE | 81 | 81 | 100 | [95.5–100] |
| CAZR-PA | 553 | 367 | 66,4 | [62.3–70.2] |
| MRSA | 5,280 | 2870 | 54,4 | [53–55.7] |
| VRE | 61 | 60 | 98,4 | [91.3–99.7] |

### Table S204: MDR prevalence within each bacterial pathogen.

| Group | Pathogen | Overall | Frequency | Percentage (%) | [95% CI] |
| --- | --- | --- | --- | --- | --- |
| Enterobacterales | *Citrobacter freundii* | 745 | 203 | 27,2 | [24.2–30.6] |
| Enterobacterales | *Citrobacter koseri* | 2,229 | 43 | 1,9 | [1.4–2.6] |
| Enterobacterales | *Escherichia coli* | 35,929 | 3648 | 10,2 | [9.8–10.5] |
| Enterobacterales | *Enterococcus cloacae* complex | 4,42 | 1050 | 23,8 | [22.5–25] |
| Enterobacterales | *Klebsiella pneumoniae* | 8,559 | 1022 | 11,9 | [11.3–12.6] |
| Enterobacterales | *Morganella morganii* | 968 | 45 | 4,6 | [3.5–6.2] |
| Enterobacterales | *Proteus mirabilis* | 4,821 | 233 | 4,8 | [4.3–5.5] |
| Enterobacterales | *Salmonella spp.* | 857 | 28 | 3,3 | [2.3–4.7] |
| Enterobacterales | *Shigella spp.* | 154 | 9 | 5,8 | [3.1–10.7] |
| Enterobacterales | *Serratia marcescens* | 1,246 | 56 | 4,5 | [3.5–5.8] |
| Enterococci | *Enterococcus faecium* | 387 | 94 | 24,3 | [20.3–28.8] |
| Enterococcus | *Enterococcus faecalis* | 4,612 | 26 | 0,6 | [0.4–0.8] |
| Non-fermenters | *Acinetobacter baumannii* | 1,339 | 336 | 25,1 | [22.8–27.5] |
| Non-fermenter | *Stenotrophomonas maltophilia* | 588 | 8 | 1,4 | [0.7–2.7] |
| Non-fermenter | *Pseudomonas aeruginosa* | 6,154 | 733 | 11,9 | [11.1–12.7] |
| Other Gram-negatives | *Haemophilus influenzae* | 5,704 | 307 | 5,4 | [4.8–6] |
| Other Gram-negative | *Neisseria gonorrhoeae* | 2,832 | 30 | 1,1 | [0.7–1.5] |
| Staphylococcus | *Staphylococcus aureus* | 24,402 | 3054 | 12,5 | [12.1–12.9] |
| Streptococcus | *Streptococcus pneumoniae* | 3,071 | 86 | 2,8 | [2.3–3.4] |
| Streptococcus | *Streptococcus agalactiae* | 837 | 5 | 1,6 | [0.7–3.6] |
| Streptococcus | *Streptococcus pyogenes* | 321 | 1 | 0,1 | [0–0.7] |

### Table S205: Hospital-acquired prevalence among MDR isolates per year.

| Year | Community-acquired | | | Hospital-acquired | | |
| --- | --- | --- | --- | --- | --- | --- |
|  | N | % | [95% CI] | N | % | [95% CI] |
| 2005 | 298 | 7.4 | [6.7–8.3] | 155 | 17.8 | [15.4–20.5] |
| 2006 | 225 | 6.3 | [5.5–7.1] | 218 | 16.8 | [14.9–19] |
| 2007 | 292 | 6.9 | [6.2–7.7] | 217 | 16.3 | [14.4–18.4] |
| 2008 | 255 | 5.6 | [5–6.3] | 171 | 14 | [12.2–16.1] |
| 2009 | 381 | 7.5 | [6.8–8.3] | 161 | 17 | [14.7–19.5] |
| 2010 | 493 | 9.6 | [8.8–10.5] | 146 | 17.3 | [14.9–20] |
| 2011 | 566 | 11.4 | [10.5–12.3] | 198 | 22.3 | [19.7–25.1] |
| 2012 | 508 | 10.8 | [10–11.7] | 148 | 19.3 | [16.6–22.2] |
| 2013 | 515 | 11.2 | [10.3–12.2] | 95 | 19 | [15.8–22.7] |
| 2014 | 635 | 12.1 | [11.2–13] | 37 | 15.5 | [11.5–20.7] |
| 2015 | 549 | 11.2 | [10.3–12.1] | 45 | 17.8 | [13.6–23] |
| 2016 | 523 | 10.4 | [9.6–11.3] | 37 | 13.8 | [10.1–18.4] |
| 2017 | 466 | 11.1 | [10.2–12.1] | 115 | 16.1 | [13.6–19] |
| 2018 | 525 | 12.8 | [11.8–13.8] | 199 | 15.6 | [13.8–17.7] |
| 2019 | 539 | 13.7 | [12.6–14.8] | 256 | 17.2 | [15.4–19.2] |
| 2020 | 567 | 13.6 | [12.6–14.7] | 214 | 18.6 | [16.5–21] |
| 2021 | 470 | 11.4 | [10.5–12.4] | 191 | 15 | [13.1–17.1] |
| 2022 | 343 | 7.4 | [6.7–8.2] | 176 | 13.2 | [11.5–15.1] |
| 2023 | 334 | 7.9 | [7.1–8.7] | 177 | 14.7 | [12.8–16.8] |
| 2024 | 238 | 6.4 | [5.7–7.2] | 147 | 14.4 | [12.4–16.7] |

### Figure S25: Annual MDR prevalence with annual proportions of hospital-acquired and community-acquired, from 2005 to 2024.


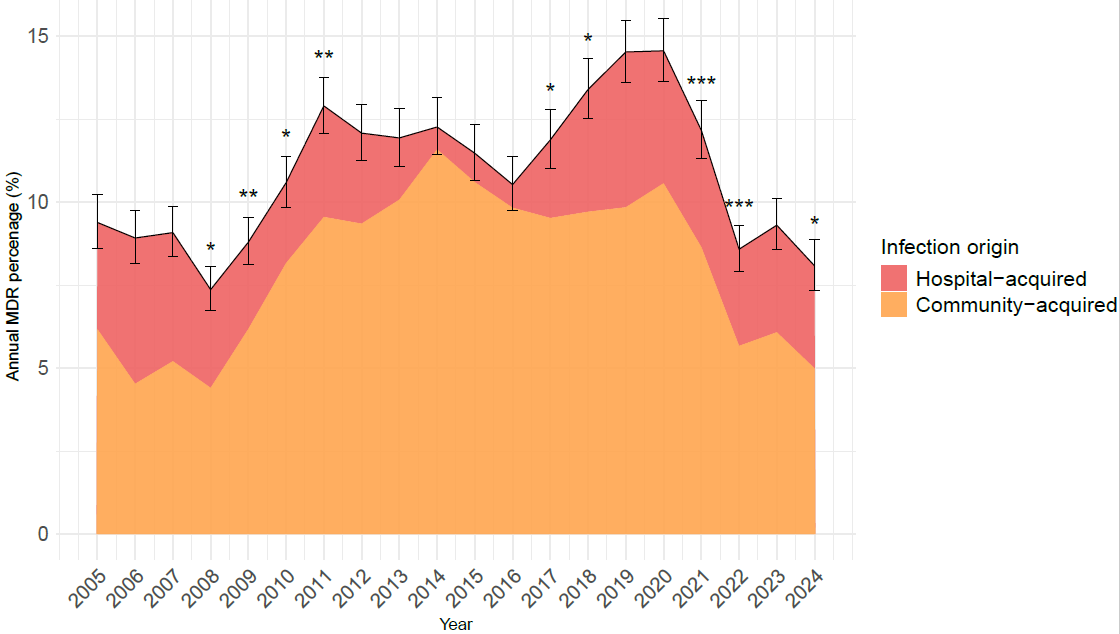


### Table S206: Annual MDR percentage among 110,205 isolates.

|  | Tested | Resistance | | Logistic model | |  | |
| --- | --- | --- | --- | --- | --- | --- | --- |
| Year | N | % | [95% CI] | OR | [95% CI] | P-value |  |
| 2005 | 4 978 | 9,4 | [8,6–10,2] | – | – | – |  |
| 2006 | 4 975 | 9,0 | [8,2–9,8] | 0,97 | [0,84–1,11] | 0,834 |  |
| 2007 | 5 672 | 9,1 | [8,4–9,9] | 1 | [0,87–1,15] | 0,996 |  |
| 2008 | 5 896 | 7,4 | [6,8–8,1] | 0,82 | [0,72–0,94] | 0,016 | * |
| 2009 | 6 134 | 8,9 | [8,2–9,7] | 1,25 | [1,1–1,43] | 0,005 | ** |
| 2010 | 6 140 | 10,7 | [9,9–11,5] | 1,17 | [1,04–1,32] | 0,027 | * |
| 2011 | 6 068 | 13,0 | [12,2–13,9] | 1,21 | [1,08–1,35] | 0,005 | ** |
| 2012 | 5 638 | 12,2 | [11,3–13] | 0,95 | [0,85–1,07] | 0,562 |  |
| 2013 | 5 249 | 12,0 | [11,2–12,9] | 1 | [0,89–1,13] | 0,996 |  |
| 2014 | 5 651 | 12,3 | [11,5–13,2] | 1 | [0,89–1,13] | 0,996 |  |
| 2015 | 5 336 | 11,6 | [10,8–12,5] | 0,91 | [0,81–1,02] | 0,179 |  |
| 2016 | 5 489 | 10,6 | [9,8–11,5] | 0,92 | [0,82–1,05] | 0,330 |  |
| 2017 | 5 030 | 11,9 | [11,1–12,9] | 1,17 | [1,03–1,32] | 0,029 | * |
| 2018 | 5 421 | 13,5 | [12,6–14,4] | 1,17 | [1,04–1,31] | 0,027 | * |
| 2019 | 5 471 | 14,6 | [13,7–15,6] | 1,05 | [0,94–1,17] | 0,562 |  |
| 2020 | 5 368 | 14,6 | [13,7–15,6] | 1 | [0,9–1,12] | 0,996 |  |
| 2021 | 5 415 | 12,2 | [11,4–13,1] | 0,8 | [0,71–0,89] | 0,001 | *** |
| 2022 | 6 026 | 8,6 | [8–9,4] | 0,68 | [0,6–0,77] | 0,000 | *** |
| 2023 | 5 465 | 9,4 | [8,7–10,2] | 1,13 | [0,99–1,29] | 0,119 |  |
| 2024 | 4 783 | 8,1 | [7,4–8,9] | 0,81 | [0,71–0,94] | 0,016 | * |

### Table S207: Mixed-Effect logistic regression analysis of the effect continuous time–blood infection and hospital-acquired infection on the presence of MDR isolates across all isolates–from 2005 to 2024.

| Variable | β | OR | [95% CI] | P-value |  |
| --- | --- | --- | --- | --- | --- |
| Time | 0.008 | 1.01 | [1.00–1.01] | <0.001 | *** |
| Male | 0.255 | 1.29 | [1.24–1.34] | <0.001 | *** |
| Age | 0.342 | 1.41 | [1.38–1.44] | <0.001 | *** |
| Hemoculture | -0.165 | 0.85 | [0.79–0.91] | <0.001 | *** |
| Hospital-acquired | 0.364 | 1.44 | [1.37–1.51] | <0.001 | *** |

### Table S208: Mixed-Effect logistic regression analysis of the effect service on the presence of MDR isolates across CHT isolates–from 2005 to 2024.

| Variable | β | OR | [95% CI] | P-value |  |
| --- | --- | --- | --- | --- | --- |
| Mental Health | 0.831 | 2.30 | [2.01–2.62] | <0.001 | *** |
| Genecology | -0.470 | 0.63 | [0.55–0.71] | <0.001 | *** |
| Medicine | 0.275 | 1.32 | [1.24–1.40] | <0.001 | *** |
| Pediatrics | 0.277 | 1.32 | [1.18–1.47] | <0.001 | *** |
| Intensive Care | 0.383 | 1.47 | [1.36–1.58] | <0.001 | *** |
| Male | 0.146 | 1.16 | [1.10–1.22] | <0.001 | * |
| Age | 0.304 | 1.35 | [1.31–1.40] | <0.001 | *** |

### Table S209: Annual pXDR percentage among 33,75 isolates.

|  | Tested | Resistance | | Logistic model | |  | |
| --- | --- | --- | --- | --- | --- | --- | --- |
| Year | N | % | [95% CI] | OR | [95% CI] | P-value |  |
| 2005 | 1570 | 1.3 | [0.8–2] | – | – | – |  |
| 2006 | 1843 | 0.7 | [0.4–1.1] | 0.66 | [0.32–1.36] | 0.611 |  |
| 2007 | 1995 | 0.3 | [0.1–0.7] | 0.48 | [0.18–1.28] | 0.545 |  |
| 2008 | 2099 | 0.3 | [0.2–0.7] | 1.18 | [0.39–3.54] | 0.860 |  |
| 2009 | 1983 | 0.8 | [0.5–1.2] | 2.24 | [0.91–5.56] | 0.385 |  |
| 2010 | 2075 | 0.3 | [0.2–0.7] | 0.41 | [0.17–1.03] | 0.385 |  |
| 2011 | 1816 | 0.6 | [0.3–1.1] | 1.83 | [0.7–4.78] | 0.586 |  |
| 2012 | 1738 | 0.4 | [0.2–0.8] | 0.75 | [0.29–1.95] | 0.851 |  |
| 2013 | 1551 | 0.9 | [0.5–1.5] | 1.88 | [0.75–4.73] | 0.562 |  |
| 2014 | 1206 | 0.7 | [0.4–1.4] | 0.85 | [0.36–1.98] | 0.851 |  |
| 2015 | 1065 | 0.8 | [0.4–1.5] | 1.2 | [0.46–3.14] | 0.851 |  |
| 2016 | 1587 | 0.5 | [0.3–1] | 1.07 | [0.39–2.88] | 0.951 |  |
| 2017 | 1649 | 0.5 | [0.3–1] | 1.3 | [0.49–3.41] | 0.851 |  |
| 2018 | 1080 | 0.7 | [0.4–1.5] | 0.83 | [0.32–2.19] | 0.851 |  |
| 2019 | 980 | 1.0 | [0.6–1.9] | 1.57 | [0.61–4.05] | 0.670 |  |
| 2020 | 1691 | 0.6 | [0.3–1.1] | 1.02 | [0.41–2.49] | 0.974 |  |
| 2021 | 1758 | 0.4 | [0.2–0.8] | 0.59 | [0.22–1.58] | 0.620 |  |
| 2022 | 2017 | 0.1 | [0–0.4] | 0.24 | [0.05–1.17] | 0.385 |  |
| 2023 | 1959 | 0.3 | [0.1–0.7] | 4.19 | [0.84–20.94] | 0.385 |  |
| 2024 | 1513 | 0.8 | [0.5–1.4] | 1.44 | [0.53–3.94] | 0.815 |  |

### Table S210: pXDR prevalence within each bacterial pathogen.

| Group | Pathogen | Overall | Frequency | Percentage (%) | [95% CI] |
| --- | --- | --- | --- | --- | --- |
| Enterobacterales | *Citrobacter freundii* | 126 | 2 | 1,6 | [0.4–5.6] |
| Enterobacterales | *Citrobacter koseri* | 474 | 0 | 0 | [0–0.8] |
| Enterobacterales | *Escherichia coli* | 17 261 | 5 | 0 | [0–0.1] |
| Enterobacterales | *Enterococcus cloacae* complex | 571 | 29 | 5,1 | [3.6–7.2] |
| Enterobacterales | *Klebsiella pneumoniae* | 1 507 | 32 | 2,1 | [1.5–3] |
| Enterobacterales | *Morganella morganii* | 235 | 2 | 0,9 | [0.2–3] |
| Enterobacterales | *Proteus mirabilis* | 949 | 0 | 0 | [0–0.4] |
| Enterobacterales | *Salmonella spp.* | 91 | 0 | 0 | [0–4.1] |
| Enterobacterales | *Serratia marcescens* | 107 | 0 | 0 | [0–3.5] |
| Non-fermenters | *Acinetobacter baumannii* | 1 253 | 23 | 1,8 | [1.2–2.7] |
| Non-fermenter | *Stenotrophomonas maltophilia* | 580 | 15 | 2,6 | [1.6–4.2] |
| Non-fermenter | *Pseudomonas aeruginosa* | 5 747 | 80 | 1,4 | [1.1–1.7] |
| Other Gram-negative | *Haemophilus influenzae* | 1 632 | 0 | 0 | [0–0.2] |
| Other Gram-negative | *Neisseria gonorrhoeae* | 1 955 | 0 | 0 | [0–0.2] |
| Streptococci | *Streptococcus agalactiae* | 618 | 0 | 0 | [0–5.3] |
| Streptococcus | *Streptococcus pyogenes* | 69 | 0 | 0 | [0–0.6] |
